# Synthesis of Cannabinoids: “In Water” and “On Water” Approaches: Influence of SDS Micelles

**DOI:** 10.1021/acs.joc.0c02698

**Published:** 2021-02-03

**Authors:** José F. Quílez del Moral, Cristina Ruiz Martínez, Helena Pérez del Pulgar, Juan Eduardo Martín González, Ignacio Fernández, José Luis López-Pérez, Alejandro Fernández-Arteaga, Alejandro F. Barrero

**Affiliations:** † Department of Organic Chemistry, Institute of Biotechnology, University of Granada, 18071 Granada, Spain; ‡ Department of Pharmaceutical Sciences, IBSAL-CIETUS, University of Salamanca, 37007 Salamanca, Spain; § Department of Chemistry and Physics, Research Centre CIAIMBITAL, University of Almería, 04120 Almería, Spain

## Abstract

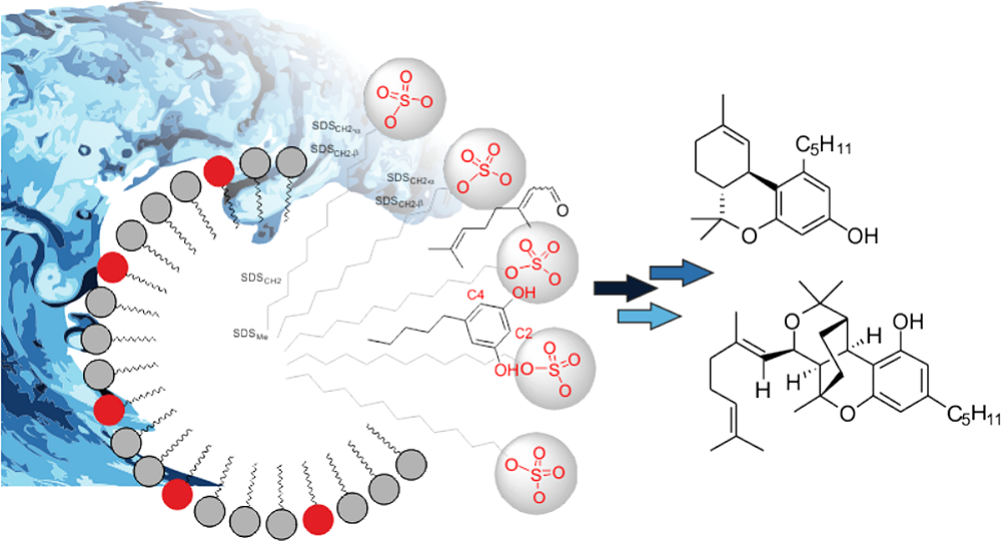

We have proven that the biomimetic-like synthesis of cannabinoids from citral and the corresponding phenolic counterpart may well be carried out using water as a solvent. The influence of different additives such as surfactants was also analyzed. Rationalization of the reaction mode and regiochemistry of the processes were provided in terms of “on water” and “in water” reactions. The same reactions were conducted in organic media using Ga(III) salts as catalysts. Worthy of being underlined, an unprecedented formal [2+2+2] process was found to occur between two citral molecules and the corresponding phenolic species in both aqueous and organic environments. Computational studies were performed in order to gain a comprehensive mechanistic and energetic understanding of the different steps of this singular process. Finally, the influence of SDS micelles in the chemical behavior of olivetol and citral was also pursued using PGSE diffusion and NOESY NMR studies. These data permitted to tentatively propose the existence of a mixed micelle between olivetol and SDS assemblies.

## Introduction

The quest for more sustainable and green chemistry has caused an increasing interest in the use of water as a solvent in organic processes.^1^ Apart from the obvious environmental benefits, many reactions also experienced improved or unexpected reactivities when performed in water.

Cannabinoids are a group of terpenophenolic compounds naturally existing in the Indian plant *Cannabis sativa*, consumed for centuries due to the euphoric sensations experienced after the plant is smoked.^2^ These natural products are biosynthesized from geranyl diphosphate and olivetolic acid.^2^ Thus, different biomimetic-like approaches toward the synthesis of cannabinoids such as tetrahydrocannabinol (THC) or cannabicromene (CBC) involve the reaction between olivetol (or other resorcinols) and citral.^3^ Multiple pharmacological studies on these substances, including their interaction with the G protein-coupled receptors, CB1 and CB2, the ion channel TRPV1, have been carried out due to their growing therapeutic interest.^4^ In fact, several states in the US, Canada, and other countries worldwide have approved their therapeutic use of marijuana.

**Scheme 1:**
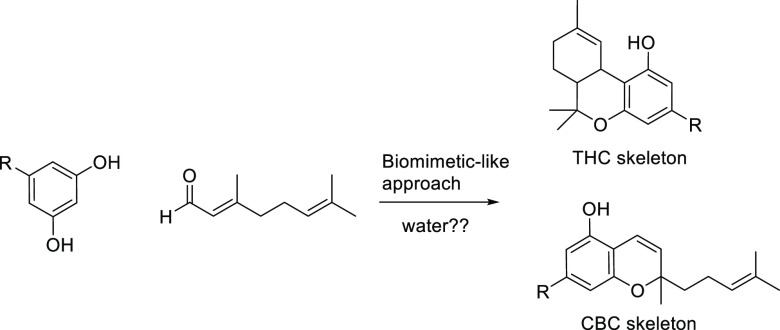
Biomimmetic-Like Approach to Cannabinoids

Encouraged by that interest and taking into consideration both that some of the synthetic procedures leading to these cannabinoids involve cycloaddition reactions,^3c,5^ and the fact that a number of pericyclic reactions are reported to efficiently proceed using water as a solvent, we envisaged that the cannabinoid skeleton of both THC and CBS could also be built on performing the reaction of citral and resorcinol derivatives using water as the solvent of choice (Scheme 1).^6^

To perform the reaction in water as a solvent, it could be convenient to use either pure water, or a microemulsion, or a related type of organized colloidal system able to mix the hydrophobic organic reagents. Being macroscopically homogeneous but microscopically dispersed, they can be regarded as something between the solvent-based one-phase systems and the true two-phase systems. There are some surfactants, not expensive, that allow the promotion of the existence of a certain region of microemulsion in systems containing both hydrophobic and hydrophilic organic compounds. In this work, sodium dodecyl sulfate (SDS) and *p*-dodecyl benzene sulfonic acid (DBSA) are used to formulate these microemulsions.^7^

Additionally, we also analyzed the outcome of these reactions in the presence of different indium and gallium halides as catalysts but using conventional organic solvents such as dichloromethane (DCM). By doing so, we intended not only to expand the limited-existing studies on these groups of Lewis acids^8^ but also to check whether the processes performed in water parallels or differ from those employing organic solvents.

## Results and Discussion

### Reaction of Citral with Resorcinol

We started our study by choosing resorcinol (**1**) as the phenolic component. After some experimentation, including variations on temperature, concentration, and quantities of both citral and resorcinol, we found that when 2 equiv of commercial citral (**2**) (a 4:1 mixture of the *E*-isomer geranial and the *Z*-isomer neral) reacts with 1 equiv of resorcinol in refluxing water for 42 h, the *ortho*-THC analogue **3** was generated as the only detected reaction product.^9^ One- and two-dimensional NMR experiments allowed unraveling the *ortho*-THC skeleton and the *trans* configuration at the interannular junction, with no traces of the corresponding *cis* diastereoisomer. The solubility of resorcinol in water would suggest that this reaction would take place “in water”.^10^ The “in water” nature of this process may also be argued to rationalize the selectivity of the condensation step (which takes place exclusively at the C4 position of resorcinol), since the C2 position is blocked by the existence of hydrogen bonds between the hydroxyl groups at C-1 and C-3 of orcinol and water.

The addition of the Bronsted acid surfactant, *p*-dodecylbenzenesulfonic acid (DBSA), did not improve the efficiency of the reaction (Table 1, entry 2). Interestingly, the nonpreviously reported *cis*-THC derivative **4** was obtained in a similar yield to that of its stereoisomer **3**. On the other hand, the addition of a strong acid or a strong base such as HCl and NaOH, respectively, did not lead to the formation of any reaction product (Table 1, entries 3 and 4).

**Table 1:**
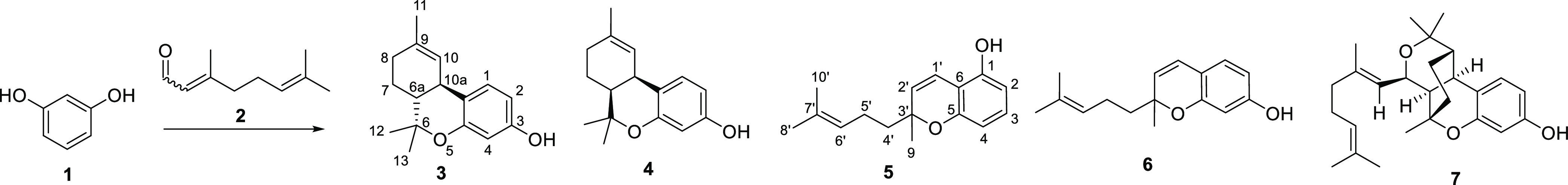
Reaction of Citral and Resorcinol

entry	**1** (equiv)	**2** (equiv)	solvent	temperature (°C)	time (h)	catalyst (equiv)	**3** (%)	**4** (%)	**5** (%)	**6** (%)	**7** (%)
1	1	2	water	reflux	42		37				
2	1	1.5	water	40	1.5	DBSA (0.1)	17	14			
3	1	2	water	reflux	62	HCl (1)					
4	1	2	water	reflux	62	NaOH (1)					
5	1.5	1	toluene	reflux	3	pyrrolidine (2)			6	8	
6	1	1.1	DCM	rt	1	GaCl_3_ (0.1)	24	25			20
7	1	1.1	DCM	rt	2	GaBr_3_ (0.1)	26	24			22
8	1	1.1	DCM	rt	0.5	GaI_3_ (0.1)	26	27			18
9	1	1.1	DCM	rt	14.5	InI_3_ (0.1)	28	28			27

When the reaction is performed following “standard” organic conditions, that is, using toluene as a solvent in the presence of pyrrolidine as a catalyst (Table 1, entry 5), the obtained products, although in marginal yields, are derived from the CBC skeletons **5** and **6**, in agreement with previous reports.^11^

**Figure 1:**
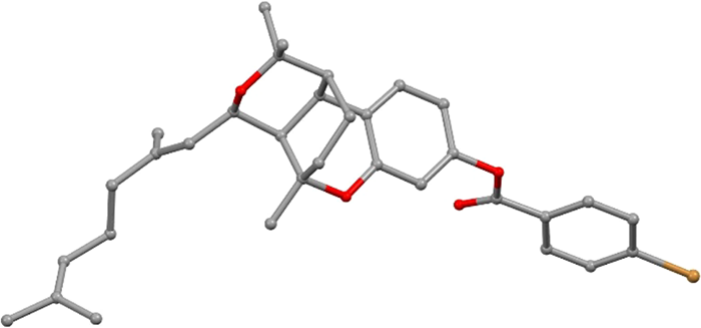
X-ray diffraction structure of compound **7a**.

Finally, the use of gallium and indium catalysts in DCM (Table 1, entries 6–9) led to similar results to those obtained using water and DBSA (Table 1, entry 2), where compounds **3** and **4** were again the major products, but with the significant presence of a third product that was identified as **7**. The structure of this tetracyclic adduct was elucidated after extensive 1D and 2D NMR analysis, including a 2D ADEQUATE experience, and unambiguously determined by X-ray crystallography (Figure 1) of the *p*-bromobenzoate derivative of **7** (**7a**) obtained via derivatization of the phenol moiety.

### Reaction of Citral with Orcinol

We continued our study using a less polar aromatic counterpart, orcinol (**8**). From the data given in Table 2, it can be concluded that the behavior of orcinol when reacting in water with citral was quite similar to that shown by resorcinol. Thus, the reaction using water with or without the presence of SDS and NH_4_Cl led to the exclusive formation of *ortho*-THC derivatives (**9**–**10**)^12^ (Table 2, entries 1–3), whereas the reaction performed in toluene originated the CBC analogue **11**.^13^ The only difference with respect to the use of orcinol was that now the presence of additives such as NH_4_Cl or the surfactant sodium dodecyl sulfate (SDS) improved the efficiency of the corresponding reactions increasing the final yield to 30% in the presence of the former and an increase of 10% in the presence of the latter. The lower polarity of orcinol can be argued to rationalize these results.

**Table 2:**
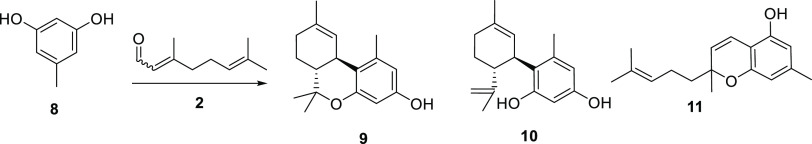
Reaction of Citral and Orcinol

entry	**8** (equiv)	**2** (equiv)	solvent	temperature (°C)	time (h)	catalyst (equiv)	**9** (%)	**10** (%)	**11** (%)
1	1	2	water	reflux	72		38		
2	1	2	water	reflux	36	SDS	48		
3	1	1	water	reflux	21	NH_4_Cl (0.2)	54	14	
4	1.5	1	toluene	reflux	13	pyrrolidine (1.3)			68

**Scheme 2:**
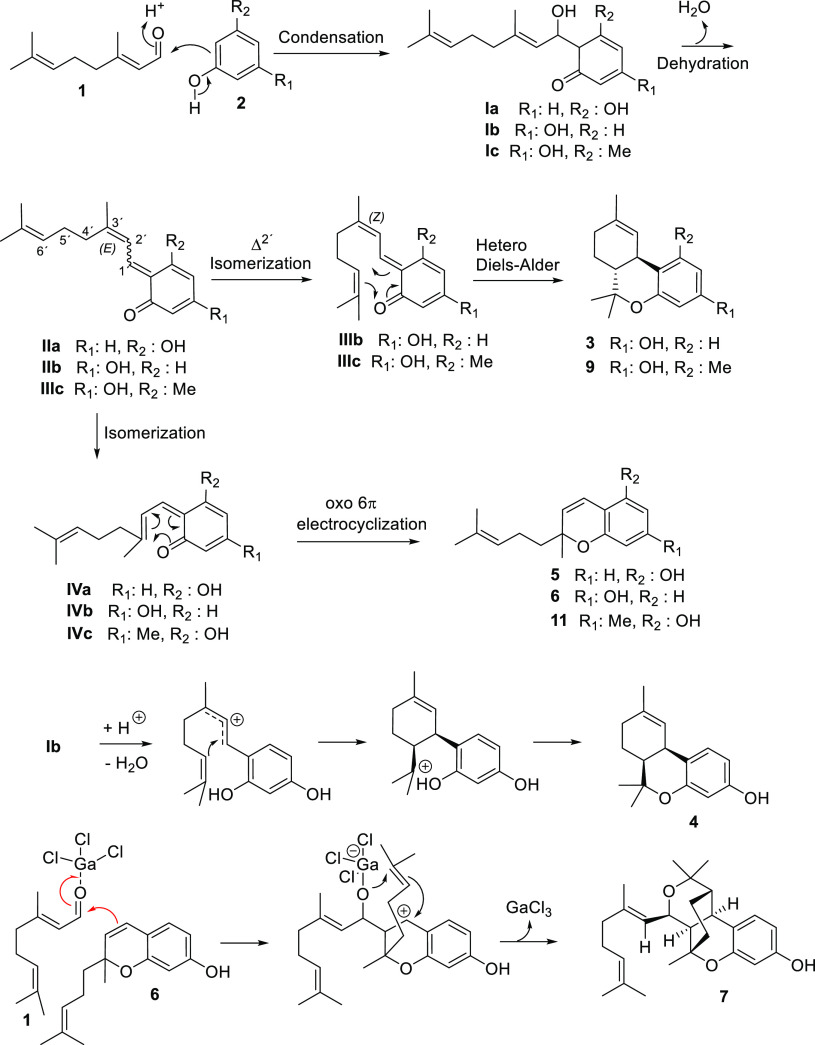
Mechanistic Proposals for the Formation of **3–11**

The mechanistic proposals for the formation of **3**–**11** are shown in Scheme 2. The process toward all of them would start with a condensation step by the electrophilic attack of resorcinol or orcinol to the carbonyl group of citral to produce after dehydration, the common intermediates **IIa**–**c**. From **IIb**,**c**, a partial *E*/*Z* isomerization of the double bond at C2′ would generate intermediates **IIIb**,**c**, which would suffer a completely stereospecific intramolecular hetero-Diels–Alder process to give the THC analogues **3** and **9** via *exo* transition states. The stereochemistry in **3** and **9** supports this mechanistic proposal. To gain a comprehensive mechanistic and energetic understanding of the exclusively generation of *trans*-diastereomers **3** or **9**, the energies of the intramolecular cyclization reactions leading to both *cis* and *trans* diastereomers were calculated via quantum chemical calculations.^14^ The results of these studies showed that the energy of the barrier conducted to the *trans* diastereomer is 10.5 kcal/mol below the barrier that would lead to the *cis* diastereomer (see Schemes S1 and S2), which supported the experimental results.

Intermediates **IIa**–**c** may also suffer an *E*/*Z* isomerization of the C4–C1′ double bond to generate intermediates **IVa**–**c**, which would evolve to the CBC analogues **5**, **6**, and **11** after an oxo 6π electrocyclization. A computational study of the evolution of intermediates **IV** toward the CBC analogues confirmed its feasibility perfectly. Thus, the generation of compound **6** was predicted to have an energetic barrier of only 6 kcal/mol (Scheme S6).

For the generation of **4**, an ionic cyclization mechanism is postulated, triggered by the acid nature of DBSA. The cationic nature of this cyclization would explain the poor stereoselectivity of the process (a mixture of **3** and **4** is generated). In this regard, it has been reported that, while the action of Brønsted acids provoked the generation of the *cis*-isomer, the presence of a Lewis acid led to the selective production of the *trans*-isomer.^15^ Computational studies were undertaken, where the acid medium was emulated by incorporating an H_3_O^+^ molecule into the calculation. In the presence of H_3_O^+^, the ketone intermediate **Ib** evolves into the more stable enolic form whose intramolecular cyclization leads to both stereoisomers **3** and **4** (Schemes S3 and S4). The energies of both transition states toward **3** (*trans* cyclization) and toward **4** (*cis* cyclization) show a very close energy value with a difference of only 0.7 kcal/mol.

**Scheme 3:**
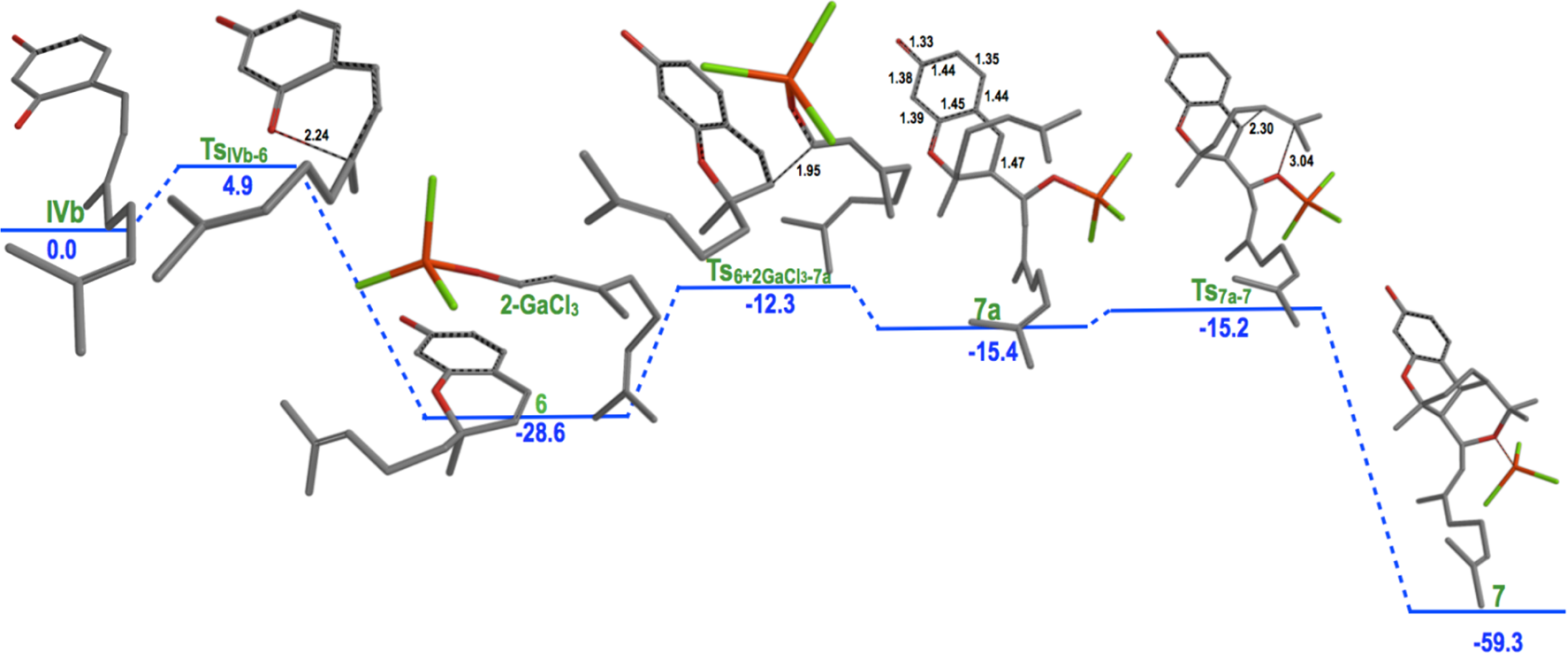
Energy Diagram for the Formation of Species Leading to Compound **7** from Condensation Intermediate **IVb** Relative energies [kcal/mol, Minnesota Functional MN15/6-31+G(d,p)]. Selected distances (Å) in TSs.

Finally, compound **6** evolves via a formal [2+2+2] heterocycloaddition, initiated by condensation of a second molecule of citral, to produce the tetracyclic structure **7**. Although some recent examples of this reaction were reported, mainly via photochemical or radical processes,^16^ no precedents of such a reaction involving two alkenes and a carbonyl group are found when the literature was revised to the best of our knowledge. Again, the production of compound **7** can be perfectly justified from the computational point of view with energetic barriers surmountable (Scheme 3). The process is predicted to involve the initial coupling of **6** to a second molecule of citral to generate the corresponding benzylic carbocation, which evolves to the final compound via concerted two carbon–carbon and carbon–oxygen forming processes to generate two new cycles. Up to 45 kcal/mol is predicted to be liberated in this step.

### Reaction of Citral with Olivetol

Finally, we chose olivetol **12** as the phenolic moiety. Olivetol is practically insoluble in water and contains a five carbon-atom chain, which is also present in both THC and CBC compounds. When olivetol reacted with citral using water as a reaction medium, and at reflux, CBC (**13**) was obtained as the major reaction product (Table 3, entry 1), together with a minor amount of the also natural cannabinoid cannabicitran (**14**).^17^ The selectivity of the pericyclic reaction, leading to cannabicitran, which possesses a *cis* transannular union, was rationalized by computational calculations. Thus, a significant difference between the energetic barriers of the processes leading to both *cis* and *trans* stereoisomers was noticed on these calculations (Schemes S8 and S9).

**Table 3:**
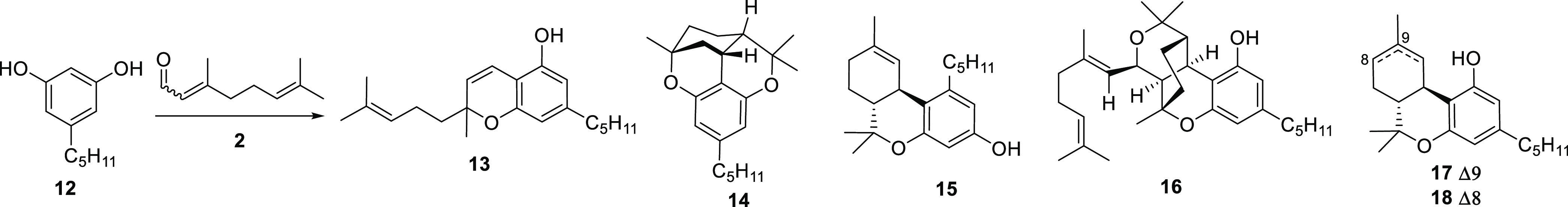
Reaction of Citral and Olivetol

entry	**12** (equiv)	**2** (equiv)	solvent	temperature (°C)	time (h)	catalyst (equiv)	**13** (%)	**14** (%)	**15** (%)	**16** (%)	**17**–**18**^a^ (%)
1	1	1	water	reflux	51		45	5			
2	1	1	water	reflux	6	SDS			47	11	
3	1	1	water	reflux	24	NH_4_Cl (0.2)	75				
4	1	0.6	toluene	reflux	1.5	pyrrolidine (1.3)	59				
5	1	1.1	DCM	rt	1	GaI_3_ (0.1)			25	8	23

a Obtained in variable proportions.

Contrary to what occurred with orcinol and resorcinol, the reactivity of olivetol in water (regioselectivity of condensation and type of cannabinoid obtained) is the same as that obtained with orcinol and resorcinol but in toluene. In this case, the addition of sub stoichiometric NH_4_Cl^18^ increased the yield up to 75% of CBC (Table 3, entry 3). This result clearly indicates a change in the reaction conditions; namely, the process no longer takes place “in water” but “on water”, as expected after the addition of the surfactant. In this scenario, the reagents should be surrounded by an organic microenvironment that defines the “on water” structuring.^19^ Additionally, the addition of SDS completely changed the reactivity of the process (Table 3, entry 2), with the *ortho*-THC **15** being obtained as the main product.^12^

Together with compound **15**, the reaction performed in water with the presence of SDS also produced tetracyclic **16**. Similar to **7**, the generation of this product involved a formal [2+2+2] cycloaddition process between now CBC (**13**) and a second molecule of citral. If the uniqueness of this process was worthy of being remarked when **7** was produced (a Ga(III)-mediated process requiring organic solvent), the fact that this formal cycloaddition takes place also in water with the only addition of a surfactant increases its singularity. This supposes a nice example of the possible complementarity of the reactions performed in water with standard organic processes.

The results obtained when gallium iodide was used as catalysts (Table 3, entry 5) also deserved to be mentioned. Thus, racemic Δ9-THC (**17**)^20^ and its isomer Δ8-THC (**18**),^20b,21^ the major (psycho-)active compounds encountered in *C. sativa*, were obtained as well as tetracyclic **16**. Considering that no CBC is obtained in these cases, the generation of THC (**17**) by the Lewis acid-promoted isomerization of the initially generated CBC (**13**) should not be completely discarded. In this regard, the thermal isomerization of analogues of CBC to analogues of THC was recently reported.^5a^

**Scheme 4:**
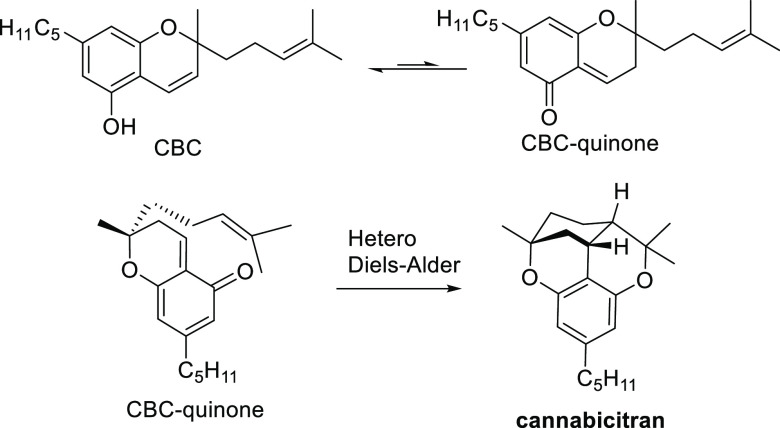
Mechanistic Proposals for the Formation of **14**

Quantum chemical calculations on the mechanism for the generation of natural cannabicitran (**14**) were supportive of the previously reported proposals suggesting a concerted hetero-Diels–Alder reaction for the production of **14** (Scheme 4).^22^

### Study of the Influence of SDS Micelles

**Scheme 5:**
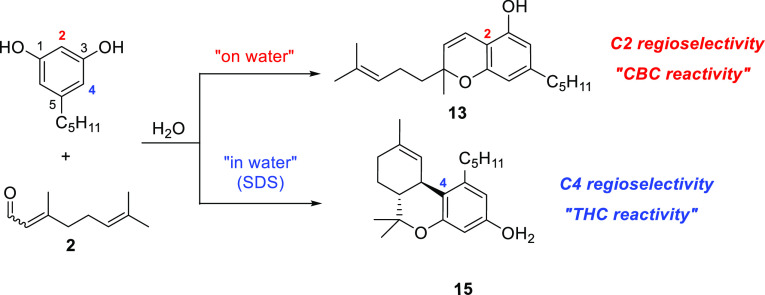
Regiochemistry in the Reaction of Olivetol and Citral Using Water as a Solvent

At this point, we turned our attention to propose a rationalization of the remarkable difference in reaction mode and regiochemistry found in the reaction of olivetol and citral using water as a solvent (Scheme 5). When the reaction is performed “on water”, the condensation with citral takes place only at C2, the most electron-rich position of the diphenol and, therefore, the kinetically favored product. The steric hindrance caused by the side carbon chain may also be argued to support this regioselectivity.

The specific generation of the *ortho* derivative of THC^12^ via C4 regioselectivity when the reaction proceeds in the presence of SDS can be rationalized considering the specific disposition of the reactants in the presence of the created micelles after the addition of the surfactant. SDS micelles^23^ include olivetol and/or citral and therefore promote their spatial proximity in such a disposition that the phenolic hydroxyls are faced toward the aqueous phase (the outer region of the supramolecular assembly) and the hydrophobic chains oriented toward the core of the micelles. In such a situation, it is plausible to assume that the carbonyl group of citral is oriented toward the outer side of the micelle, and the hydrophobic chain is disposed parallel to that of olivetol and SDS. This disposition blocked the C-2 position of olivetol for reacting, forcing the condensation with citral to take place at C-4.

To support this hypothesis, we focused our effort to cast some light on the behavior of citral, resorcinol, and olivetol in SDS micelles. It is well-known that surfactants in water spontaneously self-assemble to form micelles, where the factors influencing their formation in terms of size and shape have been extensively studied over the last decades.^24^ The presence of SDS as well-defined micelles in water has also been confirmed by investigating the dependence of their diffusion coefficients as a function of surfactant concentration, where, for instance, in the case of SDS, the critical micelle concentration (CMC) was established at 7 mM in pure D_2_O,^25^ consistent with other literature.^26^ With these antecedents in mind, we were interested in the study of the diffusion properties of the two frontier conditions, i.e., resorcinol vs olivetol, in order to explain the change in reactivity when SDS micelles are formed in the reaction crude.

Figures S1–S4 show conventional Stejskal–Tanner plots from the ^1^H PGSE NMR diffusion measurements for 2 mM samples of resorcinol (**1**), citral (**2**), and olivetol (**12**), with and without SDS at 40 mM. As the observed resonances in the ^1^H NMR spectra of the reactants in the presence of SDS are an average of free and bound (to SDS micelles) species, when the slope of the curves is smaller, the *D* value is smaller, and the hydrodynamic radius of the effective diffusing species would be larger, due to its interaction with the supramolecular entity. When this situation occurs, the obtained *D* value is referred to as the apparent diffusion coefficient. Often, the Stokes–Einstein equation and its modifications are useful and enable a molecular size estimation of large particles that are much larger than the solvent.^26^ These calculated hydrodynamic radii, *r*_H_, assume spherical shapes; hence, they do not represent the real shape of the molecules. Nevertheless, their use is well established for comparisons because they offer a rapid and easy method to recognize molecular size. Although the viscosity of the prepared solutions varies, the viscosity of the pure solvents for radii calculation in the Stokes–Einstein equation is well established.^27^

As mentioned above, several diffusion experiments were performed using D_2_O samples of SDS micelles at 40 mM; results are given in Table 4. For the diffusion studies presented, the partially deuterated chloroform and water signals were used as internal standards, ensuring good quality measurements in the whole set of measurements, obtaining averaged values of (25.9 ± 0.2) 10^–9^ m^2^ s^–1^ and (18.4 ± 0.3) 10^–9^ m^2^ s^–1^, respectively.

**Table 4:** Diffusion Coefficient (*D*) and Stokes–Einstein Hydrodynamic Radius (*r*_H_) Values for Compounds **1**, **2**, and **12** and SDS at 294 K^a^

entry	solvent	compound	*D* × 10^9^ m^2^ s^–1^	*r*_H_ (Å)^b^
1	D_2_O	SDS (40 mM)	0.1213	18.3
		HDO	1.8838	1.2
2	D_2_O	resorcinol (40 mM)	0.6951	3.1
		HDO	1.8288	1.2
3	CDCl_3_	citral (40 mM)	1.3875	2.8
		CHCl_3_	2.5789	1.5
4	CDCl_3_	olivetol (40 mM)	1.2824	3.0
		CHCl_3_	2.6072	1.5
5	D_2_O	resorcinol (2 mM)	0.6067	3.6
		SDS (40 mM)	0.1174	18.4
		HDO	1.8380	1.2
6	D_2_O	citral (2 mM)	0.3396	6.4
		SDS (40 mM)	0.1178	18.3
		HDO	1.8073	1.2
7	D_2_O	olivetol (2 mM)	0.0775	27.9
		SDS (40 mM)	0.0991	21.8
		HDO	1.8617	1.2
8	D_2_O	olivetol (2 mM)	0.0768	28.1
		citral (2 mM)	0.0939	22.9
		SDS (40 mM)	0.1051	20.5
		HDO	1.8040	1.2

a The experimental error in the *D* values is ±2%.

b The viscosity, η, used in the Stokes–Einstein equation was taken from Perry’s Chemical Engineers’ Handbook eighth Edition (www.knovel.com) for chloroform (entries 3 and 4) and water (entries 1 and 2 and 5–8).

**Figure 2:**
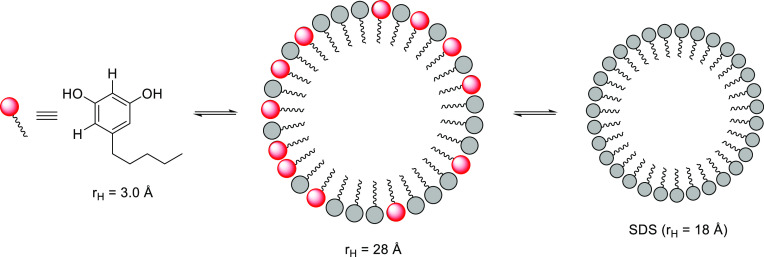
Tentative equilibrium suggested by diffusion measurements where a mixed micelle is established between olivetol and SDS assemblies.

When there are no reactants present (Table 4, entry 1), the diffusion coefficient of the micelles is 0.1213 × 10^–9^ m^2^ s^–1^ with a hydrodynamic radius of 18.3 Å, consistent with previous studies.^27,28^ When the reactants are added, their different interactions with the micelles alter their own diffusive properties. Resorcinol, for instance (Table 4, entries 2 and 5), persists almost unaffected with respect to its behavior in the absence of micelles with a slight reduction of its diffusion coefficient of Δ*D* 0.0884 × 10^–9^ m^2^ s^–1^. Contrarily, citral (Table 4, entries 3 and 6) significantly reduces its diffusion coefficient (Δ*D* of 1.0479 × 10^–9^ m^2^ s^–1^) parallel to an increase in its radius (Δ*r*_H_ 3.6 Å). The third screened reactant, olivetol (Table 4, entries 4 and 7), experiences a dramatic reduction of its *D* value of Δ*D* 1.20491 × 10^–9^ m^2^ s^–1^ and a remarkable increase in its size of Δ*r*_H_ 24.9 Å, suggesting the formation of a new type of mixed micelle between olivetol and SDS (Figure 2), inherently promoting the insertion of the olivetol entity into the matrix of SDS and justifying the distinct regioselectivity obtained.

In order to corroborate that, under similar experimental conditions, the same micellar media was created in samples containing SDS with olivetol, we performed dynamic light scattering (DLS) experiments on a Malvern Zetasizer (backscattering) instrument. We found that the size distribution by intensity shows a bimodal distribution centered at hydrodynamic volume diameters of 2.0 and 345.5 nm, which unequivocally shows a reproducible equilibrium between homo- and heterocomponent micelles previously deduced by NMR.

Finally, a sample containing olivetol, citral, and SDS micelles altogether was prepared in order to ascertain the different degrees of interaction with the micelles that both reactants are able to establish in an aqueous solution. In this context, olivetol showed the highest hydrodynamic radius of 28.1 Å, whereas SDS and citral showed sizes of 22.9 and 20.5 Å, respectively (Table 4, entry 8). In this situation, a mixed micelle between olivetol and SDS is proposed, where citral is in equilibrium between its free state and bound to this mixed micelle. The observed averaged diffusion coefficient for citral (D = 0.0939; rH = 22.9 A) suggests that it mainly exists in its bound state.

**Figure 3:**
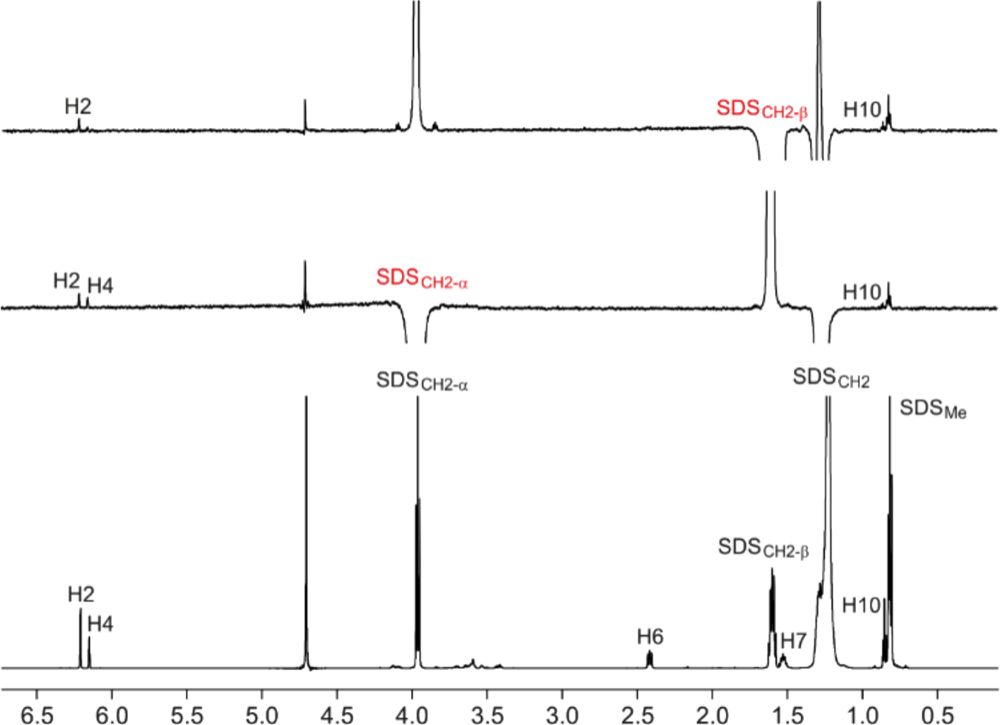
Comparison of the ^1^H NMR spectrum of olivetol (2 mM) in the presence of SDS (40 mM) and the ^1^H-DPFGSE NOESY spectrum (tm 1.0 s) with selective excitation of the α (bottom) and β (top) methylene SDS protons.

Together with the quantitative analysis of the diffusive properties of the reactants at 2 mM in the presence of SDS at a concentration above the CMC, we performed a qualitative examination of one-dimensional and two-dimensional NOESY performed on samples constituted ex professo as previously mentioned. Under these experimental conditions, the corresponding integration of the NOE enhancements provided us with an intensity map for the interaction of each component within the SDS micelle. It is remarkable that olivetol interacts with the methylenic groups of the SDS micelles (Figure 3), exhibiting a clear insertion into the inner part of the micelle and explaining its likely role as an efficient scaffold in the mixed supramolecular assembly. Unfortunately, the severe overlap of the signals produced by the pentyl chain of olivetol with the SDS resonances inhibits the observation of any other cross-peaks, limiting the discussion to only the aromatic protons H2 and H4 and H10 as indicated in Figure 3.

**Figure 4:**
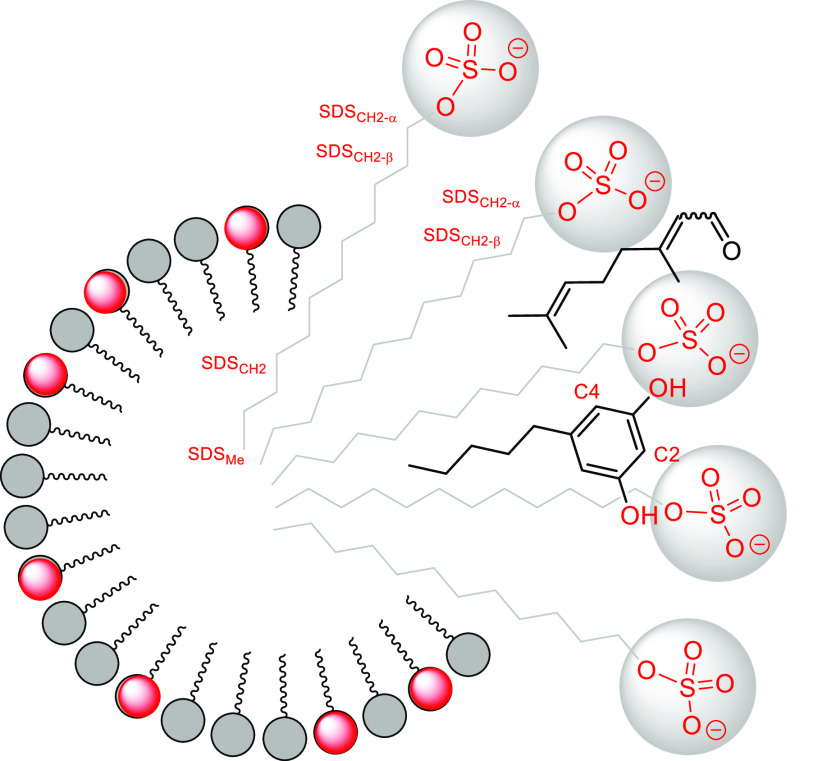
Schematic representation of the location of the reactants within the micelle based on NOE enhancements detected in the NOESY experiments.

On the other hand, the terminal methyls of citral only gave NOE enhancements with the α-methylenic SDS protons, describing a picture where the aldehydic species is not so deeply inserted into the micelle (Figure 4), which matches well with the diffusion data. The analysis of the 2D NOESY map of the sample constituted by resorcinol (**1**) and SDS did not show any dipolar intermolecular interaction, suggesting an almost null inclusion of the diphenol into the supramolecular assembly, as was already suggested by diffusion measurements.

Figure 4 depicts a reasonable solution picture that is built based on the NOE interactions and the diffusive properties that result in a compromise between a strongly inserted olivetol and a more labile-bound citral.

T1 measurements performed on both systems, i.e., resorcinol and olivetol with and without the presence of SDS micelles, corroborated the different behavior in terms of averaged interactions within the micelles. Figure S5 shows the relaxation times determined using the inversion–recovery sequence. In the case of resorcinol, all of the protons suffer a reduction in their relaxation times of 1.3, 1.1, and 0.8 s for H2, H4, and H5, respectively, whereas, in olivetol, the reduction is even more pronounced, especially for H2 with a reduction of 2.3 s, giving an idea that their magnetic environment and probably its tumbling rate have substantially changed when interacting with the micelles due to its rather larger inclusion into the micelle.

**Scheme 6:**
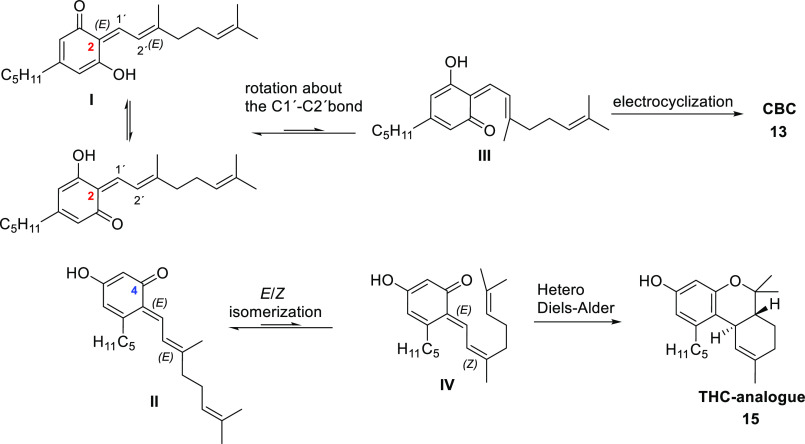
Proposal for the Diverse Reactivity Found after the C2 or C4 Condensation of Olivetol and Citral

Finally, once we found experimental support for the existence of mixed SDS–olivetol micelles and their influence in the regiochemistry of the condensation of citral and olivetol, we proposed the following explanation for the different reactivity found when SDS is present or not (Scheme 6). Thus, when the condensation occurs at C2, only a tautomeric equilibrium change would be required to reach intermediate **III**, prone to suffer an electrocylization, leading to CBC. In the case of the C4 condensation adduct (**II**), no carbonyl group would be then easily available for the electrocyclization to take place, and so the system would evolve via isomerization of the Δ2′ double bond to give intermediate **IV**, the precursor of the THC analogue via a hetero-Diels–Alder cycloaddition (Scheme 6).

## Conclusions

We have proven how citral and resorcinol derivatives react, making use of water as a solvent in a biomimetic-like approach to afford different cannabinoid derivatives. Remarkably, the reactivity and regioselectivity of the corresponding reaction performed “in water” and “on water” were different and highly selective, giving rise to THC analogues, in one case, and to CBC derivatives, in the other. PGSE diffusion and NOESY NMR studies have been applied in order to unravel the solution picture of all of the reactants in the presence of micelles, suggesting a clear insertion of olivetol (**12**), a small to medium interaction of citral (**2**), and almost no interaction of resorcinol (**1**), what drives the regioselectivity of the processes. Another remarkable result unprecedented in water was the generation of the tricyclic derivative **17**, which involves a formal [2+2+2] heterocycloaddition. Additionally, we found that the results derived from the employment of catalytic gallium and indium salts present certain similarities to those obtained “in water” such as the one-step synthesis of Δ8-THC and Δ9-THC, the psychotropic active principles of cannabis.

## Experimental Section

All air- and water-sensitive reactions were performed in flame-dried flasks under a positive flow of argon and conducted under an argon atmosphere. The solvents used were purified according to standard literature techniques and stored under argon. Anhydrous dichloromethane was distilled from calcium hydride (5% w/v) under a positive pressure of nitrogen. THF was freshly distilled immediately prior to use from sodium/benzophenone and strictly deoxygenated for 30 min under argon. Reagents were purchased at a higher commercial quality and used without further purification, unless otherwise stated. Compounds **12** and **13** are commercially available. Silica gel SDS 60 (35–70 μm) was used for flash column chromatography. Reactions were monitored by thin-layer chromatography (TLC) carried out on 0.25 mm E. Merck silica gel plates (60F-254) using UV light as the visualizing agent and solutions of phosphomolybdic acid in ethanol. HPLC with UV detection was used. Semipreparative HPLC separation was carried out on a column (5 μm Silica, 10 × 250 mm) at a flow rate of 2.0 mL/min in an Agilent Series 1100 instrument. NMR spectra were performed with Varian Direct Drive 600 (^1^H 600 MHz/^13^C 151 MHz), Varian Direct Drive 500 (^1^H 500 MHz/^13^C 126 MHz), Varian Direct Drive 400 (^1^H 400 MHz/^13^C 100 MHz), and Varian Inova Unity (^1^H 300 MHz/^13^C 75 MHz) spectrometers. High-resolution MS was determined on an Autospec-Q VG-Analytical (FISONS) mass spectrometer. DEPT 135 and two-dimensional (COSY, HSQC, HMBC, NOESY) NMR spectroscopy were used where appropriate to assist the assignment of signals in the ^1^H and ^13^C NMR spectra.

### Reaction of Citral with Phenolic Compounds in Water: General Procedure

To a round-bottom flask containing citral were added the phenolic compound and water. The mixture was then heated at refluxing temperature (oil bath) until the disappearance of the starting material. Then, brine was added, and the aqueous phase was extracted with EtOAc (×3). The organic layer was dried with Na_2_SO_4_ and concentrated under a vacuum. The residue was purified by column chromatography.

#### Using Resorcinol as a Phenolic Compound

According to the general procedure, the reaction of citral (**2**) (274 mg, 1.8 mmol) with resorcinol (**1**) (100 mg, 0.9 mmol) in refluxing water (5 mL) for 42 h provided after flash chromatography (H/MTBE, 4:1) 37% of compound **3**(9) (82 mg, 0,34 mmol) as a colorless oil.

#### (6a*S*,10a*R*)-6,6,9-Trimethyl-6a,7,8,10a-tetrahydro-6*H*-benzo[*c*]chromen-3-ol (**3**):

^1^H NMR (300 MHz, CDCl_3_) δ 7.18 (d, *J* = 8.4 Hz, 1H), 6.41 (dd, *J* = 8.3, 2.6 Hz, 1H), 6.31 (d, *J* = 2.5 Hz, 1H), 5.92 (bs, 1H), 3.15 (d, *J* = 10.7 Hz, 1H), 2.18–2.08 (m, 2H), 1.90–1.30 (m, 3H), 1.76 (bs, 3H), 1.45 (s, 3H), 1.19 (s, 3H); ^13^C{^1^H} NMR (75 MHz, CDCl_3_) δ 154.9, 154.3, 134.7, 126.2, 122.1, 117.2, 107.0, 103.8, 78.2, 44.7, 33.5, 30.8, 27.9, 26.9, 24.5, 20.8; HRMS (ESI-TOF) *m*/*z* [M + H]^+^ calcd for C_14_H_20_O_2_ 245.1542, found 245.1549.

#### Using Orcinol as a Phenolic Compound

According to the general procedure, the reaction of citral (740 mg, 4,8 mmol) with orcinol (300 mg, 2.4 mmol) in refluxing water (45 mL) for 72 h provided after flash chromatography (H/MTBE, 4:1) 38% of compound **9**(12) as a colorless oil (226 mg, 0.88 mmol).

#### (6a*R*,10a*R*)-1,6,6,9-Tetramethyl-6a,7,8,10a-tetrahydro-6*H*-benzo[*c*]chromen-3-ol (**9**):

^1^H NMR (600 MHz, CDCl_3_) δ 6.26 (d, *J* = 1.8 Hz, 1H), 6.14 (d, *J* = 1.9 Hz, 1H), 5.80 (bs, 1H), 3.12 (bd, *J* = 11.0 Hz, 1H), 2.32 (s, 3H), 2.25–2.10 (m, 2H), 1.92 (dd, *J* = 12.4, 7.5 Hz, 1H), 1.70 (bt, *J* = 11.0 Hz, 1H), 1.66 (bs, 3H), 1.43–1.36 (m, 1H), 1.39 (s, 3H), 1.05 (s, 3H); ^13^C{^1^H} NMR (151 MHz, CDCl_3_) δ 154.9, 154.3, 138.2, 134.0, 125.5, 116.6, 110.1, 101.9, 76.9, 46.8, 35.0, 30.9, 27.4, 25.2, 23.3, 21.2, 18.8; HRMS (ESI-TOF) *m*/*z* [M + H]^+^ calcd for C_17_H_23_O_2_ [M + H]^+^ 259.1698, found 259.1689.

#### Using Olivetol as a Phenolic Compound

According to the general procedure, the reaction of citral (150 mg 0.8 mmol) with olivetol (300 mg, 2.4 mmol) in refluxing water (22.5 mL) for 52 h provided after flash chromatography (H/MTBE, 5:1) 45% of **13** as a colorless oil (113 mg, 0.43 mmol) and 5% of compound **14**(17) as a colorless oil (13 mg, 0. 04 mmol).

#### Cannabicitran (**14**):

^1^H NMR (600 MHz, CDCl_3_) δ 6.32 (s, 1H,), 6.27 (s, 1H,), 2.85 (bs, 1H), 2.50 (t, *J* = 7.7 Hz, 2H), 2.21 (dt, *J* = 13.1, 4.1 Hz, 1H), 2.01 (ddd, *J* = 11.3, 5.0, 2.7 Hz, 1H), 1.82 (d, *J* = 13.1 Hz, 1H), 1.76 (bd, *J* = 15.3 Hz, 1H), 1.59–1.53 (m, 2H), 1.51 (s, 3H,), 1.41 (dt, *J* = 14.5, 7.0 Hz, 1H), 1.37 (s, 3H), 1.34–1.20 (m, 5H), 1.01 (s, 3H), 0.87 (t, *J* = 7.0 Hz, 3H), 0.65–0–56 (m, 1H); ^13^C{^1^H} NMR (151 MHz, CDCl_3_) δ 156.9, 156.5, 142.5, 114.0, 109.7, 108.8, 83.5, 74.4, 46.8, 37.3, 36.1, 35.3, 31.4, 30.9, 29.7, 29.0, 28.1, 23.7, 22.5, 22.1, 14.0; HRMS (ESI-TOF) *m*/*z* [M + H]^+^ calcd for C_21_H_30_O_2_ 315.2324, found 315.2324.

### Reaction of Citral with Phenolic Compounds in Water and SDS: General Procedure

To a round-bottom flask containing citral was added an aqueous emulsion of SDS (0.4 mM). The mixture was then heated at refluxing temperature (oil bath) until the disappearance of the starting material. Then, brine was added, and the aqueous phase was extracted with MTBE (×3). The organic layer was dried with Na_2_SO_4_ and concentrated under a vacuum. The residue was purified by column chromatography.

#### Using Orcinol as a Phenolic Compound

According to the general procedure, the reaction of citral (125 mg, 0.78 mmol) with orcinol (300 mg, 2.4 mmol) in 2.5 mL of an aqueous emulsion of SDS under reflux for 3.5 h provided after flash chromatography (H/MTBE, 4:1) 48% of compound **9** (98 mg, 0.38 mmol).

#### Using Olivetol as a Phenolic Compound

According to a general procedure, the reaction of citral (240 mg, 1.57 mmol) with olivetol (200 mg, 1.61 mmol) in 6 mL of an aqueous emulsion of SDS under reflux for 6 h provided after flash chromatography (H/MTBE, 5:1) mixture of 314 mg of compounds **15** and **16** (58%, 2.3:1 ratio).

#### (6a*R*,10a*R*)-6,6,9-Trimethyl-1-pentyl-6a,7,8,10a-tetrahydro-6*H*-benzo[*c*]chromen-3-ol (**15**)

A fraction enriched in compound **15** was subjected to HPLC (normal phase, H/MTBE, 85:15, *t*_R_ = 10.7 min) to give pure **15**(12) as a colorless oil: ^1^H NMR (300 MHz, CDCl_3_) δ 6.33 (d, *J* = 2.7 Hz, 1H), 6.16 (d, *J* = 2.6 Hz, 1H), 5.73–5.70 (m, 1H), 3.16 (d, *J* = 12.7 Hz, 1H), 2.65 (t, *J* = 15.7 Hz, 2H), 2.23–2.17 (m, 2H), 1.99–1.92 (m, 2H), 1.69 (s, 3H), 1.64–1.59 (m, 1H), 1.40 (s, 3H), 1.39–1.34 (m, 6H), 1.06 (s, 3H), 0.93 (t, *J* = 11.9 Hz, 3H); ^13^C{^1^H} NMR (75 MHz, CDCl_3_) δ 154.8, 154.4, 143.4, 134.7, 126.8, 116.4, 108.7, 101.7, 76.9, 47.0, 34.7, 33.1, 31.8, 31.0, 30.9, 27.4, 25.1, 23.2, 22.5, 18.70, 14.05; HRMS (ESI-TOF) *m*/*z* [M + H]^+^ calcd for C_21_H_30_O_2_ 315.2324, found 315.2318.

#### (1*S*,4*R*,4a*R*,5*S*,10b*R*)-4-((*E*)-2,6-Dimethylhepta-1,5-dien-1-yl)-2,2,5-trimethyl-8-pentyl-1,4a,5,10b-tetrahydro-2*H*,4*H*-1,5-ethanopyrano[3,4-*c*]chromen-10-ol (**16**)

A fraction enriched in compound **16** was subjected to HPLC (normal phase, H/MTBE, 3:1, *t*_R_ = 8.5 min) to give pure **16** as a colorless oil: ^1^H NMR (300 MHz, CDCl_3_) δ 6.18 (d, *J* = 0.8 Hz, 1H), 6.02 (d, *J* = 1.2 Hz, 1H), 5.23 (d, *J* = 8.0 Hz, 1H), 5.02 (s, 1H), 4.83 (d, *J* = 8.4 Hz, 1H), 4.39 (s, 1H), 2.38 (t, *J* = 7.9 Hz, 3H), 1.97 (m, 2H), 2.21 (m, 2H), 1.97 (s, 1H), 1.73 (s, 3H), 1.65 (s, 3H), 1.58 (s, 3H), 1.51 (s, 3H), 1.47 (s, 3H), 1.43 (s, 3H), 1.31 (s, 3H), 1.12 (m, 4H), 1.02 (s, 2H), 0.81 (t, *J* = 6.9 Hz, 3H); ^13^C{^1^H} NMR (75 MHz, CDCl_3_) δ 156.7, 151.5, 142.5, 135.5, 131.9, 127.1, 123.9, 107.6, 105.8, 78.5, 77.2, 76.9, 76.7, 75.3, 70.8, 41.5, 39.0, 35.7, 35.6, 32.5, 31.5, 30.7, 28.5, 26.2, 25.6, 23.9, 23.1, 22.5, 20.6, 17.6, 14.0; HRMS (ESI-TOF) *m*/*z* [M + H]^+^ calcd for C_31_H_47_O_3_ 467.3525, found 467.3522.

### Reaction of Citral with Phenolic Compounds in Water and NH_4_Cl: General Procedure

To a round-bottom flask containing citral was added an aqueous solution of NH_4_Cl. The mixture was then heated at refluxing temperature (oil bath) until the disappearance of the starting material. Then, brine was added, and the aqueous phase was extracted with MTBE (×3). The organic layer was dried with Na_2_SO_4_ and concentrated under a vacuum. The residue was purified by column chromatography

#### Using Orcinol as a Phenolic Compound

According to a general procedure, the reaction of citral (150 mg, 0.98 mmol) with orcinol (122 mg, 0.98 mmol) in 22 mL of an aqueous solution of NH_4_Cl (11 mg, 0.20 mmol) under reflux for 24 h provided after flash chromatography (H/MTBE, 4:1) 54% of compound **9** as a colorless oil (139 mg, 0.52 mmol) and 14% (35 mg, 0.14 mol) of compound **10**(12) as a colorless oil.

#### (1′*R*,2′*R*)-5′,6-Dimethyl-2′-(prop-1-en-2-yl)-1′,2′,3′,4′-tetrahydro-[1,1′-biphenyl]-2,4-diol (**10**):

IR (neat) 3414, 2966, 2922, 1620, 1594, 1464, 1376, 1328, 1264, 1149, 1136, 1055, 989, 890, 840, 738 cm^–1^; ^1^H NMR (600 MHz, CDCl_3_) δ 6.22 (bs, 1H), 6.14 (bs, 1H), 5.55 (bs, 1H), 4.66 (bs, 1H), 4.47 (bs, 1H), 3.56 (d, *J* = 8.7 Hz, 1H), 2.49–2.43 (m, 1H), 2.27–2.07 (m, 2H), 2.16 (s, 3H), 1.87–1.72 (m, 2H), 1.80 (s, 3H), 1.58 (s, 3H); ^13^C{^1^H} NMR (151 MHz, CDCl_3_) δ 156.4, 154.4, 147.6, 139.8, 139.0, 124.5, 120.5, 111.5, 109.4, 102.1, 45.1, 40.2, 30.2, 28.0, 23.6, 21.1, 20.9; HRMS (ESI-TOF) *m*/*z* [M + H]^+^ calcd for C_17_H_23_O_2_ 259.1698, found 259.1690.

#### Using Olivetol as a Phenolic Compound

According to the general procedure, the reaction of citral (150 mg, 0.98 mmol) with orcinol (177 mg, 0.98 mmol) in 22 mL of an aqueous solution of NH_4_Cl (11 mg, 0.20 mmol) under reflux for 24 h provided after flash chromatography (H/MTBE, 4:1) 75% of compound **13** (230 mg, 0.73 mmol).

### Reaction of Citral with Resorcinol in Water and DBSA

To a solution of DBSA (0.45 mmol, 148 mg) in 15 mL of water were added resorcinol (100 mg, 0.9 mmol) and then citral (207 mg, 1.5 mmol). The mixture was then heated at refluxing temperature (oil bath) for 1 h. Then, brine was added, and the aqueous later was extracted with MTBE (3 × 50 mL). The combined organic layers were washed with brine (3 × 100 mL), dried with Na_2_SO_4_, and concentrated under a vacuum. The residue was flash chromatographed (H/MTBE, 4:1) to afford 68 mg of a mixture of **3** and **4** (31%, 1.2:1 ratio).

#### (6a*S*,10a*R*)-6,6,9-Trimethyl-6a,7,8,10a-tetrahydro-6*H*-benzo[*c*]chromen-3-ol (**4**)

A fraction enriched in compound **4** was subjected to HPLC (normal phase, H/MTBE 85:15, *t*_R_ = 26.5 min) to give pure **4** as a colorless oil: ^1^H NMR (600 MHz, CDCl_3_) δ 7.08 (d, *J* = 8.3 Hz, 1H), 6.37 (dd, *J* = 8.4, 2.6 Hz, 1H), 6.23 (d, *J* = 2.5 Hz, 1H) 5.88 (d, *J* = 5.3 Hz, 1H), 3.46 (s, 1H), 2.04–1.89 (m, 2H), 1.84 (dd, *J* = 13.0, 5.5 Hz, 1H), 1.69 (s, 3H), 1.60 (ddd, *J* = 12.8, 5.7, 2.8 Hz, 1H), 1.40 (s, 3H), 1.26 (s, 3H), 1.25 (d, *J* = 5.7 Hz, 1H); ^13^C{^1^H} NMR (151 MHz, CDCl_3_) δ 157.1, 155.6, 137.5, 131.8, 124.8, 120.1, 110.5, 106.3, 78.8, 42.1, 34.3, 33.0, 29.1, 28.2, 26.2, 22.3; HRMS (ESI-TOF) *m*/*z* [M + H]^+^ calcd for C_16_H_21_O_2_ 245.1542, found 245.1533.

### Reaction of Citral with Phenolic Compounds in Toluene and Pyrrolidine: General Procedure

To a solution of citral and the corresponding phenolic compound in dry toluene was added pyrrolidine. The mixture was then heated at refluxing temperature until the disappearance of the starting material. Then, the mixture was diluted MTBE and washed with 2 N HCl. The organic layer was then washed with brine, dried with Na_2_SO_4_, and concentrated under a vacuum. The residue was purified by column chromatography.

#### Using Resorcinol as a Phenolic Compound

According to the general procedure, toluene (11 mL), pyrrolidine (213 mg, 3 mmol), citral (230 mg, 1.51 mmol), and resorcinol (300 mg, 2.27 mmol) were heated. After 3 h, 100 mL of MTBE were added, and the resulting solution was washed with a solution of 2 N HCl (2 × 50 mL) and brine (3 × 50 mL). The organic layer was dried with Na_2_SO_4_ and concentrated under a vacuum. Purification with silica gel chromatography yielded 6% of **5**(11) as a colorless oil (H/MTBE, 4:1) (44 mg, 0.18 mmol) and 8% of **6**(11) as a colorless oil (H/MTBE, 4:1) (33 mg, 0.13 mmol).

#### (2-Methyl-2-(4-methylpent-3-en-1-yl)-2*H*-chromen-5-ol (**5**):

^1^H NMR (500 MHz, CDCl_3_) δ 6.95 (t, *J* = 8.1 Hz, 1H), 6.67 (d, *J* = 10.5 Hz, 1H), 6.44–6.40 (td, 1H), 6.30 (dd, *J* = 8.1, 0.9 Hz, 1H), 5.58 (d, *J* = 10.0 Hz, 1H), 5.12 (tt, *J* = 7.1, 1.4 Hz, 1H), 2.18–2.08 (m, 2H), 1.80–1.69 (m, 2H), 1.69–1.67 (m, 3H), 1.60 (d, *J* = 1.3 Hz, 3H), 1.41 (s, 3H); ^13^C{^1^H} NMR (126 MHz, CDCl_3_) δ 154.3, 151.2, 131.71, 128.9, 128.3, 124.1, 116.7, 109.4, 109.2, 107.4, 78.2, 41.1, 26.3, 25.7, 22.7, 17.6.

#### 2-Methyl-2-(4-methylpent-3-en-1-yl)-2*H*-chromen-7-ol (**6**):

^1^H NMR (500 MHz, CDCl_3_) δ 6.84 (d, *J* = 7.9 Hz, 1H), 6.35–6.28 (m, 3H), 5.44 (d, *J* = 9.8 Hz, 1H), 5.12 (tp, *J* = 7.2, 1.5 Hz, 1H), 2.18–2.06 (m, 2H), 1.75 (ddd, *J* = 13.9, 10.6, 6.1 Hz, 1H), 1.68 (bs, 3H), 1.67–1.61 (m, 1H), 1.59 (bs, 3H), 1.40 (s, 3H); ^13^C{^1^H} NMR (126 MHz, CDCl_3_) δ 156.43, 154.65, 131.71, 127.17, 126.82, 124.13, 122.37, 114.75, 107.34, 103.49, 78.78, 41.37, 26.62, 25.68, 22.73, 17.63.

#### Using Orcinol as a Phenolic Compound

According to the general procedure, toluene (4 mL), pyrrolidine (46 mg, 0.66 mmol), citral (82 mg, 0.53 mmol), and orcinol (96 mg, 0.78 mmol) were heated. After 13 h, 50 mL of MTBE was added, and the resulting solution was washed with a solution of HCl (2 N) (2 × 25 mL) and brine (3 × 25 mL). The organic layer was dried with Na_2_SO_4_ and concentrated under a vacuum. Purification with silica gel chromatography yielded 68% of **11**(13) as a colorless oil (H/MTBE, 5:1) (93 mg, 0.36 mmol).

#### 2,7-Dimethyl-2-(4-methylpent-3-en-1-yl)-2*H*-chromen-5-ol (**11**):

^1^H NMR (300 MHz, CDCl_3_) δ 6.64 (dd, *J* = 10.0, 0.8 Hz, 1H), 6.30–6.25 (m, 1H), 6.17–6.13 (m, 1H), 5.52 (d, *J* = 10.0 Hz, 1H), 5.13 (ddq, *J* = 8.9, 6.0, 1.6 Hz, 1H), 2.24 (s, 3H), 2.19–2.09 (m, 2H), 1.79–1.70 (m, 2H), 1.69 (d, *J* = 1.4 Hz, 3H), 1.61 (d, *J* = 1.3 Hz, 3H), 1.40 (s, 3H); ^13^C{^1^H} NMR (75 MHz, CDCl_3_) δ 154.2, 151.43, 139.8, 131.9, 127.4, 124.4, 116.9, 110.1, 108.5, 106.9, 78.4, 41.3, 26.6, 25.9, 23.0, 21.8, 17.9.

### Reaction of Citral with Phenolic Compounds in the Presence of Lewis Acids: General Procedure

To a solution of citral and the corresponding phenolic compound in dry toluene was added the corresponding Lewis acid. The mixture was then stirred at room temperature until the disappearance of the starting material. Then, the mixture was concentrated under a vacuum, and the residue was purified by column chromatography.

#### Using Resorcinol as a Phenolic Compound

According to the general procedure, dichloromethane (10 mL), citral (303 mg, 1.9 mmol), resorcinol (**1**) (200 mg, 1.8 mmol), and Lewis acid (0.18 mmol, 0.1 equiv) were stirred. After 0.5–14.5 h, the mixture was concentrated under a vacuum. The crude product obtained was purified by column chromatography over silica gel (H/MTBE 5:1) to obtain a mixture of compounds **3**, **4**, and **7** (for yields and ratios, see Table 1).

#### (1*S*,4*R*,4a*R*,5*S*,10b*R*)-4-((*E*)-2,6-Dimethylhepta-1,5-dien-1-yl)-2,2,5-trimethyl-1,4a,5,10b-tetrahydro-2*H*,4*H*-1,5-ethanopyrano[3,4-*c*]chromen-8-ol (**7**)

A fraction enriched in compound **7** was subjected to HPLC (normal phase, H/MTBE, 85:15) to give pure **7** (*t*_R_ = 11.8 min): ^1^H NMR (500 MHz, CDCl_3_) δ 6.83 (d, *J* = 8.1 Hz, 1H), 6.32 (dd, *J* = 8.1, 2.5 Hz, 1H), 6.28 (d, *J* = 2.5 Hz, 1H), 5.32 (dd, *J* = 7.3, 1.0 Hz, 1H), 5.09 (bt, *J* = 6.8 Hz, 1H), 4.84 (dd, *J* = 7.2, 2.6 Hz, 1H), 3.32 (s, 1H), 2.29 (td, *J* = 13.9, 6.0 Hz, 1H), 2.15–2.00 (m, 4H), 1.80 (s, 1H), 1.77–1.64 (m, 2H), 1.69 (s, 3H), 1.65 (d, *J* = 1.2 Hz, 3H), 1.61 (d, *J* = 1.2 Hz, 3H), 1.50 (s, 3H), 1.50–1.45 (m, 1H), 1.41–1.38 (m, 1H), 1.40 (s, 3H), 1.33 (s, 3H); ^13^C{^1^H} NMR (126 MHz, CDCl_3_) δ 157.0, 155.1, 135.6, 131.6, 128.2, 126.4, 124.1, 118.8, 106.4, 101.7, 79.0, 75.3, 71.2, 41.9, 41.1, 39.8, 36.1, 35.6, 30.3, 28.5, 26.2, 25.7, 24.0, 19.9, 17.6, 16.8; HRMS (ESI-TOF) *m*/*z* [M + H]^+^ calcd for C_26_H_36_O_3_ 397.2743, found 397.2742.

#### (*E*)-4-(2,6-Dimethylhepta-1,5-dien-1-yl)-2,2,5-trimethyl-1,4a,5,10b-tetrahydro-2*H*,4*H*-1,5-ethanopyrano[3,4-*c*]chromen-8-yl 4-bromobenzoate (**7a**)

To a solution of **7** (213 mg, 0.59 mmol) in dry DCM (6 mL) was added 0.12 mL of Et_3_N (0.89 mmol), DMAP (144 mg, 1.18 mmol), and *p*-bromobenzoyl chloride (258 mg, 1.18 mmol) at room temperature under an argon atmosphere. After the mixture was stirred for 30 min, the reaction diluted with 10 mL of MTBE was quenched with a saturated aqueous solution of NH_4_Cl (5 mL). The resulting mixture was extracted with MTBE (3 × 25 mL). The combined organic layer was washed with brine, dried over Na_2_SO_4_, and concentrated under reduced pressure. The residue was column chromatographed (H/MTBE 99:1) to afford 289 mg of **7a** (84% yield) as a white solid. The solid was dissolved in 10 mL of absolute ethanol, and 4–5 drops of water were added. The mixture was then heated to its boiling point for a few seconds and then let to cool to room temperature for 24 h before the crystals were filtered off: ^1^H NMR (500 MHz, CDCl_3_) δ 8.06 (d, *J* = 8.5 Hz, 2H), 7.65 (d, *J* = 8.5 Hz, 2H), 7.02 (d, *J* = 8.1 Hz, 1H), 6.68 (dd, *J* = 8.1, 2.2 Hz, 1H), 6.66 (d, *J* = 2.2 Hz, 1H), 5.34 (d, *J* = 7.0 Hz, 1H), 5.11 (t, *J* = 6.6 Hz, 1H), 4.84 (dd, *J* = 7.1, 2.6 Hz, 1H), 3.42 (s, 1H), 2.29 (td, *J* = 13.7, 5.8 Hz, 1H), 2.18–2.01 (m, 4H), 1.86 (s, 1H), 1.81–1.69 (m, 2H), 1.70 (s, 3H), 1.67 (s, 3H), 1.63 (s, 3H), 1.55–1.43 (m, 2H), 1.42 (s, 3H), 1.35 (s, 3H); ^13^C{^1^H} NMR (126 MHz, CDCl_3_) δ 165.6, 156.9, 150.1, 131.9, 131.6, 128.7, 128.6, 128.1, 126.3, 124.2, 124.0, 112.2, 108.3, 79.3, 75.3, 71.1, 41.8, 40.8, 39.8, 36.5, 35.6, 30.3, 28.5, 26.2, 25.7, 24.0, 19.9, 17.7, 16.9.

#### Using Olivetol as a Phenolic Compound

According to the general procedure, dichloromethane (10 mL), citral (303 mg, 1.9 mmol), olivetol (**12**) (324 mg, 1.8 mmol), and gallium iodide (0.18 mmol, 0.1 equiv) were stirred. After 1 h, the mixture was concentrated under a vacuum. The crude product obtained was purified by column chromatography over silica gel using mixtures of H and MTBE of increasing polarity as an eluent to obtain a mixture of compounds **15**–**17** (H/MTBE 5:1) (for yields and ratios, see Table 3).

#### Δ9-Tetrahydrocannabinol (**17**)

A fraction containing compound **17** was subjected to HPLC (normal phase, H/MTBE, 9:1) to give pure **17**(21a) (*t*_R_ = 6.8 min): ^1^H NMR (500 MHz, CDCl_3_) δ 6.33–6.31 (m, 1H), 6.29 (d, *J* = 1.4 Hz, 1H), 6.16 (d, *J* = 1.4 Hz, 1H), 4.69 (s, 1H), 3.22 (bd, *J* = 11.1 Hz, 1H), 2.46 (td, *J* = 8.1, 7.3, 2.1 Hz, 2H), 2.21–2.16 (m, 2H), 1.97–1.91 (m, 1H), 1.73–1.68 (m, 1H), 1.70 (s, 3H), 1.62–1.56 (m, 2H), 1.46–1.40 (m, 1H), 1.43 (s, 3H), 1.36–1.29 (m, 4H), 1.12 (s, 3H), 0.90 (t, *J* = 6.9 Hz, 3H).

#### Δ8-Tetrahydrocannabinol (**18**)

A fraction containing compound **18** was subjected to HPLC (normal phase, H/MTBE, 9:1) to give pure **18**(21) (*t*_R_ = 6.3 min): ^1^H NMR (400 MHz, CDCl_3_) δ 6.30 (d, *J* = 1.5 Hz, 1H), 6.13 (d, *J* = 1.6 Hz, 1H), 5.45 (bd, *J* = 4.9 Hz, 1H), 4.62 (s, 1H), 3.21 (dd, *J* = 16.7, 5.9 Hz, 1H), 2.72 (td, *J* = 10.6, 4.2 Hz, 1H), 2.46 (td, *J* = 7.3, 2.3 Hz, 2H), 2.20–2.12 (m, 1H), 1.90–1.80 (m, 2H), 1.73 (s, 3H), 1.70–1.57 (m, 3H), 1.43 (s, 3H), 1.37–1.28 (m, 4H), 1.13 (s, 3H), 0.90 (t, *J* = 7.0 Hz, 3H); HRMS (ESI-TOF) *m*/*z* [M + H]^+^ calcd for C_21_H_30_O_2_ 315.2324, found 315.2315.

## Supporting Information Available

Copies of ^1^H and ^13^C{^1^H} NMR and bidimensional spectra of the obtained compounds, computational data, results of diffusion experiments, and crystallographic data for compound **7a** (PDF)

## Accession Codes

Deposition Number 2021364 contains the supplementary crystallographic data for this paper. These data are provided free of charge by the joint Cambridge Crystallographic Data Centre and Fachinformationszentrum Karlsruhe Access Structures service www.ccdc.cam.ac.uk/structures.

## References

[ref1] aOrganic Synthesis in Water; Grieco P. A., Ed.; Blackie: London, 1998.

[ref2] aMedicinal Natural Products; Dewick P. M., Ed.; John Wiley & Sons Ltd: Chichester, UK, 2009; pp 10–20.

[ref3] aTaylor E. C.; Lenard K.; Shvo Y. Active Constituents of Hashish. Synthesis of Dl-Δ6–3,4-Trans-Tetrahydrocannabinol. J. Am. Chem. Soc. 1966, 88, 367–369. 10.1021/ja00954a039.

[ref4] aBladen C.; McDaniel S. W.; Gadotti V. M.; Petrov R. R.; Berger N. D.; Diaz P.; Zamponi G. W. Characterization of Novel Cannabinoid Based T-Type Calcium Channel Blockers with Analgesic Effects. ACS Chem. Neurosci. 2015, 6, 277–287. 10.1021/cn500206a. 25314588 PMC4372069

[ref5] aGarcia A.; Borchardt D.; Chang C.-E. A.; Marsella M. J. Thermal Isomerization of Cannabinoid Analogues. J. Am. Chem. Soc. 2009, 131 (46), 16640–16641. 10.1021/ja907062v. 19919138

[ref6] Kitanosono T.; Masuda K.; Xu P.; Kobayashi S. Catalytic Organic Reactions in Water toward Sustainable Society. Chem. Rev. 2018, 118, 679–746. 10.1021/acs.chemrev.7b00417. 29218984

[ref7] For an example of previous use of DBA, see: Li J.-T.; Du C.; Xu X.-Y.; Chen G.-F. Synthesis of 2-(1,5-Diaryl-1,4-Pentadien-3-Ylidene)-Hydrazinecarboximidamide Hydrochloride Catalyzed by p-Dodecylbenzenesulfonic Acid in Aqueous Media under Ultrasound Irradiation. Ultrason. Sonochem. 2012, 19, 1033–1038. 10.1016/j.ultsonch.2012.02.009. 22440718

[ref8] aLi H.-J.; Guillot R.; Gandon V. A Gallium-Catalyzed Cycloisomerization/Friedel–Crafts Tandem. J. Org. Chem. 2010, 75, 8435–8449. 10.1021/jo101709n. 21082803

[ref9] Dethe D. H.; Erande R. D.; Mahapatra S.; Das S.; B V. K. Protecting Group Free Enantiospecific Total Syntheses of Structurally Diverse Natural Products of the Tetrahydrocannabinoid Family. Chem. Commun. 2015, 51, 2871–2873. 10.1039/C4CC08562K. 25582920

[ref10] Shapiro N.; Vigalok A. Highly Efficient Organic Reactions “on Water”, “in Water”, and Both. Angew. Chem., Int. Ed. 2008, 47, 2849–2852. 10.1002/anie.200705347. 18318031

[ref11] Elsohly H. N.; Turner C. E.; Clark A. M.; Elsohly M. A. Synthesis and Antimicrobial Activities of Certain Cannabichromene and Cannabigerol Related Compounds. J. Pharm. Sci. 1982, 71, 1319–1323. 10.1002/jps.2600711204. 7153877

[ref12] Giorgi P. D.; Liautard V.; Pucheault M.; Antoniotti S. Biomimetic Cannabinoid Synthesis Revisited: Batch and Flow All-Catalytic Synthesis of (±)-Ortho-Tetrahydrocannabinols and Analogues from Natural Feedstocks. Eur. J. Org. Chem. 2018, 2018, 1307–1311. 10.1002/ejoc.201800064.

[ref13] Caprioglio D.; Mattoteia D.; Minassi A.; Pollastro F.; Lopatriello A.; Muňoz E.; Taglialatela-Scafati O.; Appendino G. One-Pot Total Synthesis of Cannabinol via Iodine-Mediated Deconstructive Annulation. Org. Lett. 2019, 21, 6122–6125. 10.1021/acs.orglett.9b02258. 31339327

[ref14] To gain a mechanistic and energetic understanding of the formation of the products described in this work, we have performed the corresponding computational calculations. For this study, we have used the optimized Minnesota Functional MN15, in order to consider the dispersion forces, together with the base set 6-31+G(d,p), which adds not only polarization but also diffusion, important to improve the results: Yu H. S.; He X.; Li S. L.; Truhlar D. G. MN15: A Kohn–Sham Global-Hybrid Exchange–Correlation Density Functional with Broad Accuracy for Multi-Reference and Single-Reference Systems and Noncovalent Interactions. Chem. Sci. 2016, 7, 5032–5051. 10.1039/C6SC00705H. 30155154 PMC6018516

[ref15] Malkov A. V.; Kocovsky P. Tetrahydrocannabinol Revisited: Synthetic Approaches Utilizing Molybdenum Catalysts. Collect. Czech. Chem. Commun. 2001, 66, 1257–1268. 10.1135/cccc20011257.

[ref16] aDaeppen C.; Kaiser M.; Neuburger M.; Gademann K. Preparation of Antimalarial Endoperoxides by a Formal [2+2+2] Cycloaddition. Org. Lett. 2015, 17, 5420–5423. 10.1021/acs.orglett.5b02773. 26491785

[ref17] Bercht C. A. L.; Lousberg R. J. J. C.; Küppers F. J. E. M.; Salemink C. A. Cannabicitran: A New Naturally Occurring Tetracyclic Diether from Lebanese Cannabis Sativa. Phytochemistry 1974, 13, 619–621. 10.1016/S0031-9422(00)91362-1.

[ref18] Breslow R. Hydrophobic Effects on Simple Organic Reactions in Water. Acc. Chem. Res. 1991, 24, 159–164. 10.1021/ar00006a001.

[ref19] Narayan S.; Muldoon J.; Finn M. G.; Fokin V. V.; Kolb H. C.; Sharpless K. B. On Water”: Unique Reactivity of Organic Compounds in Aqueous Suspension. Angew. Chem., Int. Ed. 2005, 44, 3275–3279. 10.1002/anie.200462883. 15844112

[ref20] aMechoulam R.; Gaoni Y. A Total Synthesis of dl-Δ1-Tetrahydrocannabinol, the Active Constituent of Hashish^1^. J. Am. Chem. Soc. 1965, 87, 3273–3275. 10.1021/ja01092a065. 14324315

[ref21] aHae Choi Y.; Hazekamp A.; Peltenburg-Looman A. M. G.; Frédérich M.; Erkelens C.; Lefeber A. W. M.; Verpoorte R. Phytochem. Anal. 2004, 15, 345–354. 10.1002/pca.787. 15595449

[ref22] Crombie L.; Redshaw S. D.; Whiting D. A. The Mechanism of Intramolecular ‘Citran’ Bicyclisation of Chromens: Stereochemistry of a Forward (H+ Catalysed) and a Related Reverse (Thermal) Process. J. Chem. Soc., Chem. Commun. 1979, 14, 630–631. 10.1039/C39790000630.

[ref23] In order to characterize the mentioned aggregates that modify the reaction, we have carried out dynamic light scattering (DLS) measurements of the samples to determine the micelle size. The RH of the SDS micelle size is 1.8 ± 0.2 nm, whereas that of the mixed micelles was found to rang from 40 to 120 nm. The DLS measurements corroborate the solubilization of olivetol and citral in aqueous micelles, which is a prerequisite for reactions to be influenced by micelles.

[ref24] aPrinciples of Colloid and Surface Chemistry, 3rd ed.; Hiemenz P. C., Rajagopalan R., Eds., Marcel Dekker: New York, 1997.

[ref25] Cassani J.; Nilsson M.; Morris G. A. Flavonoid Mixture Analysis by Matrix-Assisted Diffusion-Ordered Spectroscopy. J. Nat. Prod. 2012, 75, 131–134. 10.1021/np2005264. 22276617

[ref26] Markarian S. A.; Harutyunyan L. R.; Harutyunyan R. S. The Properties of Mixtures of Sodium Dodecylsulfate and Diethylsulfoxide in Water. J. Solution Chem. 2005, 34, 361–368. 10.1007/s10953-005-3056-x.

[ref27] Raya-Barón Á.; Oña-Burgos P.; Fernández I. Diffusion NMR Spectroscopy Applied to Coordination and Organometallic Compounds. In Annual Reports on NMR Spectroscopy; Webb G. A., Ed.; Academic Press, 2019; Vol. 98, pp 125–191; Chapter 3.

[ref28] All diffusion processing and molecular size estimations were performed by using the DiffAtOnce software package available at http://www.diffatonce.com.

