# Supplementary material for: Synthesis of Cannabinoids: “In Water” and “On Water” Approaches: Influence of SDS Micelles
Source: J Org Chem. 2021 Feb 3;86(4):3344–55. doi: 10.1021/acs.joc.0c02698 (PMC9087200; doi:10.1021/acs.joc.0c02698)
Supplement: Supplementary file 1 — jo0c02698_si_001.pdf [file jo0c02698_si_001.pdf]

Supporting information

Synthesis of Cannabinoids: “In Water” and “On Water” Approaches: Influence of SDS Micelles

José F. Quílez del Moral,* Cristina Ruiz Martínez, Helena Pérez del Pulgar, Juan Eduardo Martín González, Ignacio Fernández, José Luis López-Pérez,* Alejandro Fernández-Arteaga, and Alejandro F. Barrero*

Corresponding Authors

José F. Quílez del Moral: *Department of Organic Chemistry, Institute of Biotechnology, University of Granada, 18071 Granada, Spain;* orcid.org/0000-0003-4806-6693; Email: jfquilez@ugr.es

Alejandro F. Barrero: *Department of Organic Chemistry, Institute of Biotechnology, University of Granada, 18071 Granada, Spain;* orcid.org/0000-0002-3058-8760; Email: afbarre@ugr.es

José Luis López-Pérez: *Department of Pharmaceutical Sciences, IBSAL-CIETUS, University of Salamanca, 37007 Salamanca, Spain*; Email: lopez@usal.es

Authors

Helena Pérez del Pulgar and Juan Eduardo Martín González: *Department of Organic Chemistry, Institute of Biotechnology, University of Granada, 18071 Granada, Spain.*

Cristina Ruiz Martínez and Ignacio Fernández: *Department of Chemistry and Physics, Research Centre CIAIMBITAL, University of Almería, 04120, Almería, Spain*

Alejandro Fernández-Arteaga: *Department of Chemical Engineering, University of Granada, 18071 Granada, Spain.*

Content

1. ^{1}H-NMR, ^{13}C-NMR and Bidimensional Spectra S3

2. Diffusion experiments S40

3. Computational Details S45

4. Single Crystal X-ray Diffraction S89

5. References S92

1. ^{1}H-NMR, ^{13}C-NMR and Bidimensional Spectra

^{1}H NMR of **3** (300 MHz, $CDCl_3$)

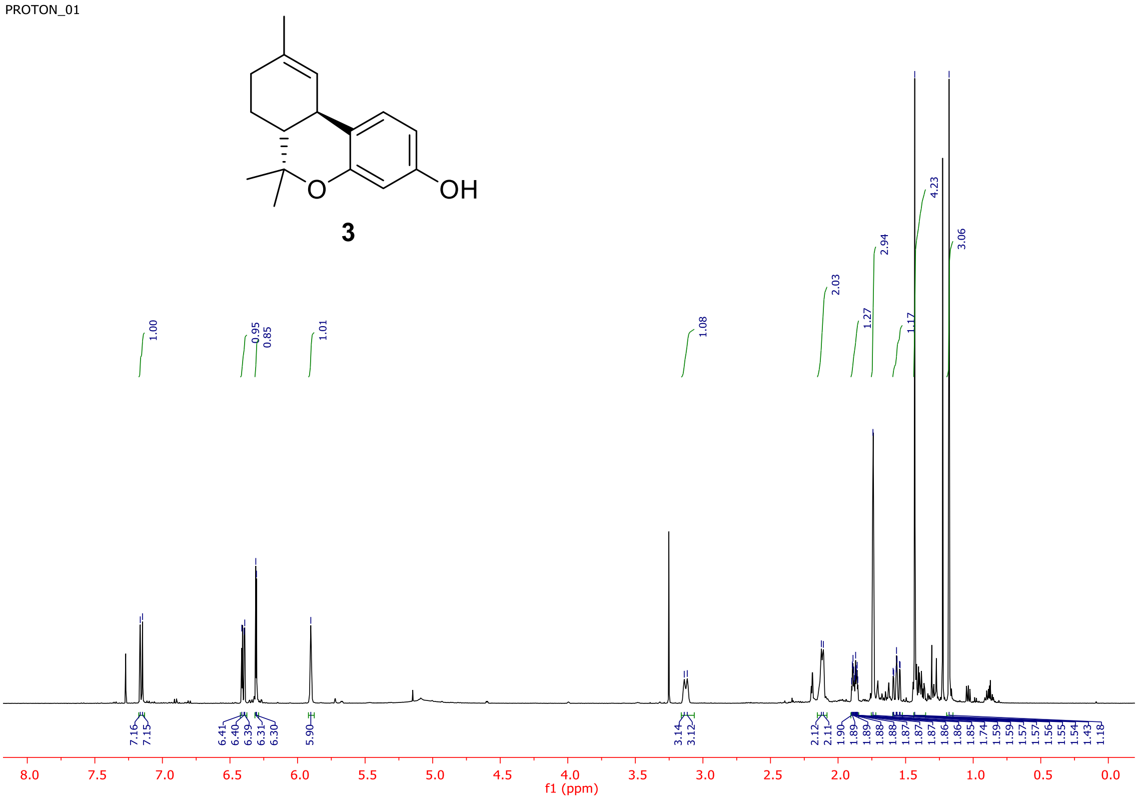

^{13}C{^{1}H} NMR of **3** (75 MHz, $CDCl_3$)

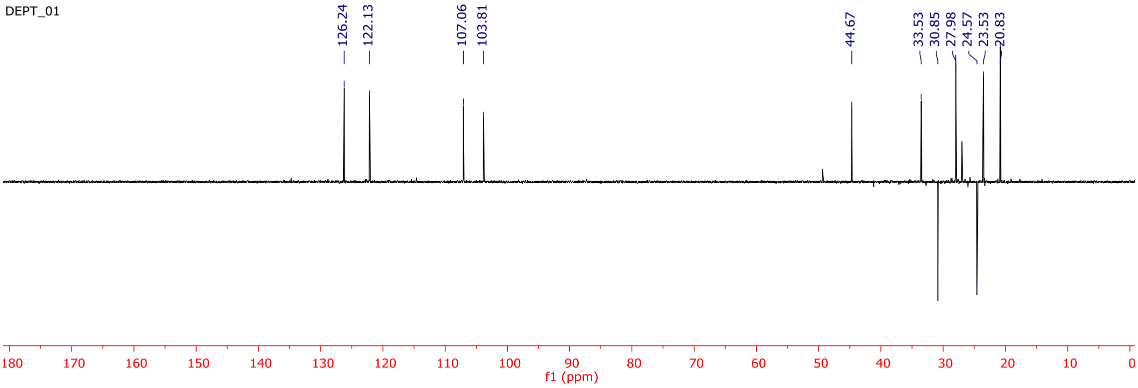

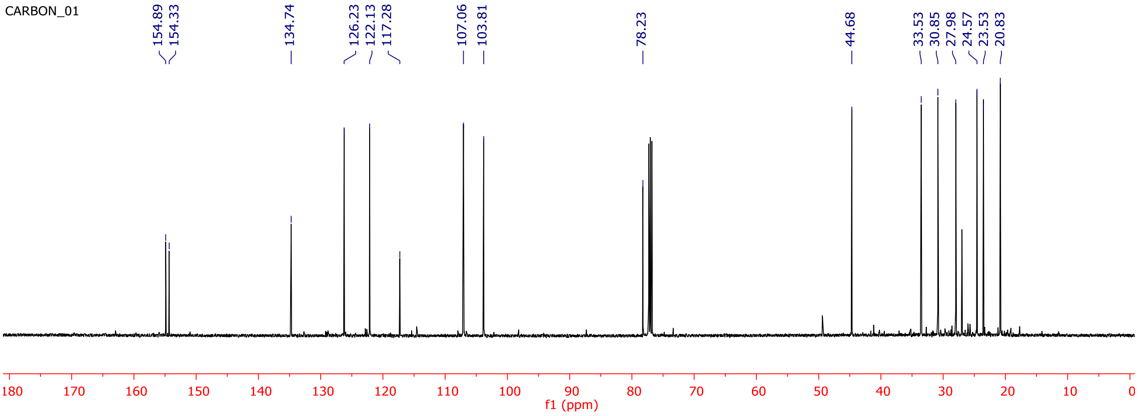

COSY of 3 (300 MHz, $CDCl_3$)

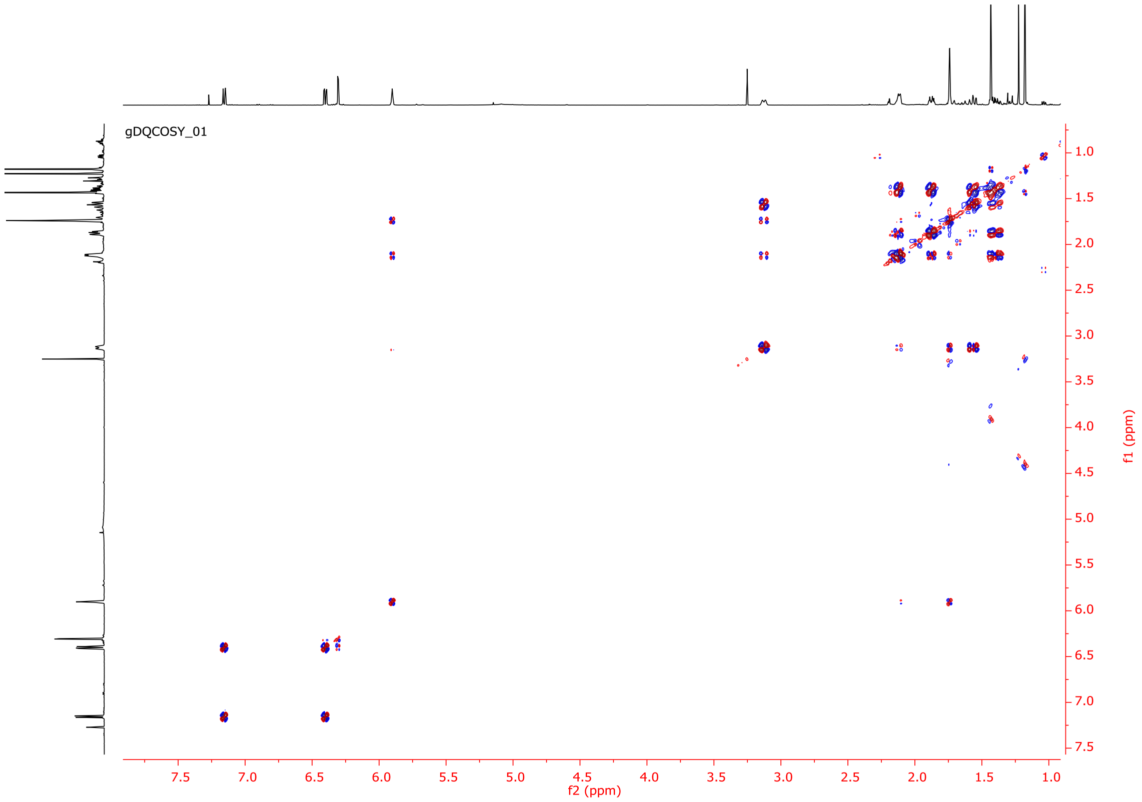

HSQC of 3 (300/75 MHz, $CDCl_3$)

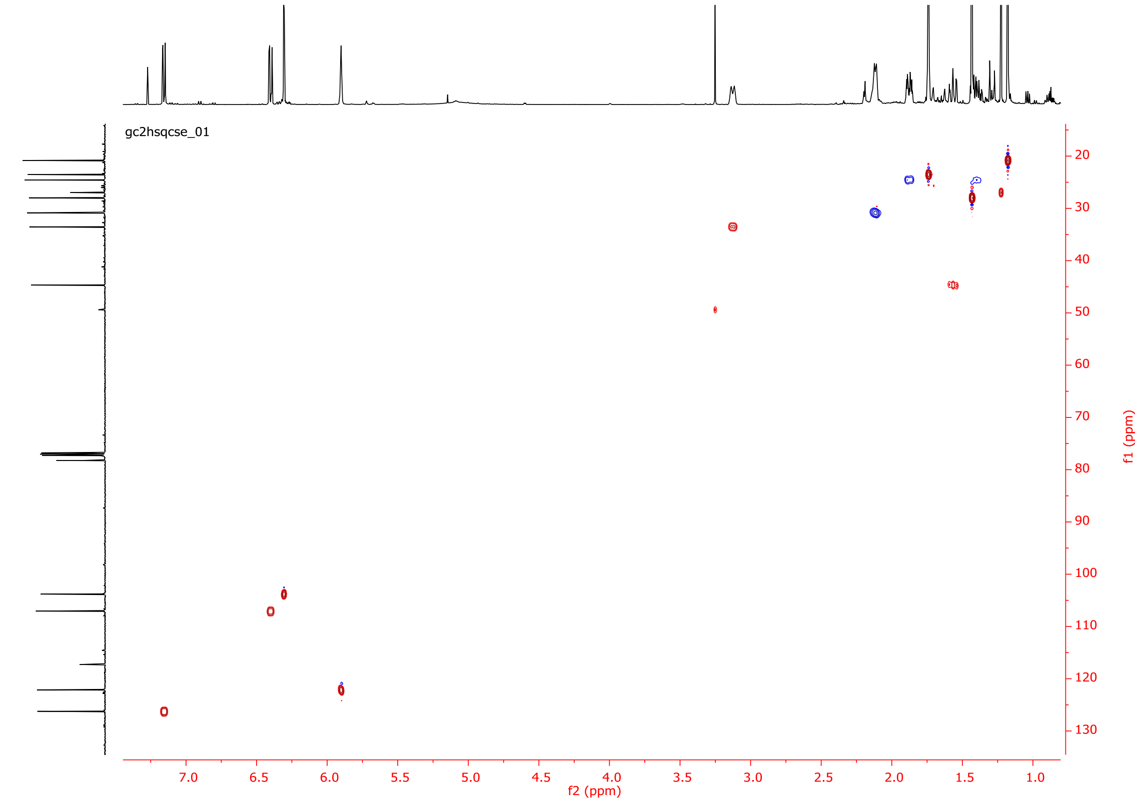

HMBC of 3 (300/75 MHz, $CDCl_3$)

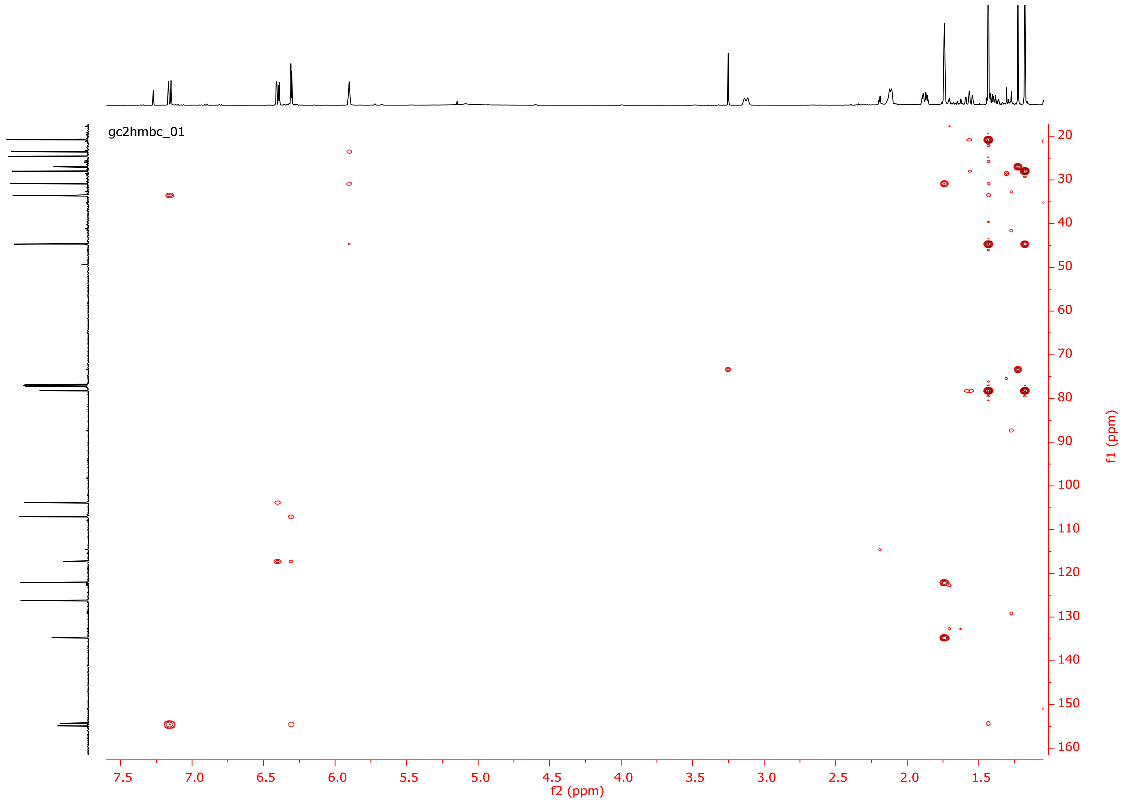

^{1}H NMR of **4** (600 MHz, $CDCl_3$)

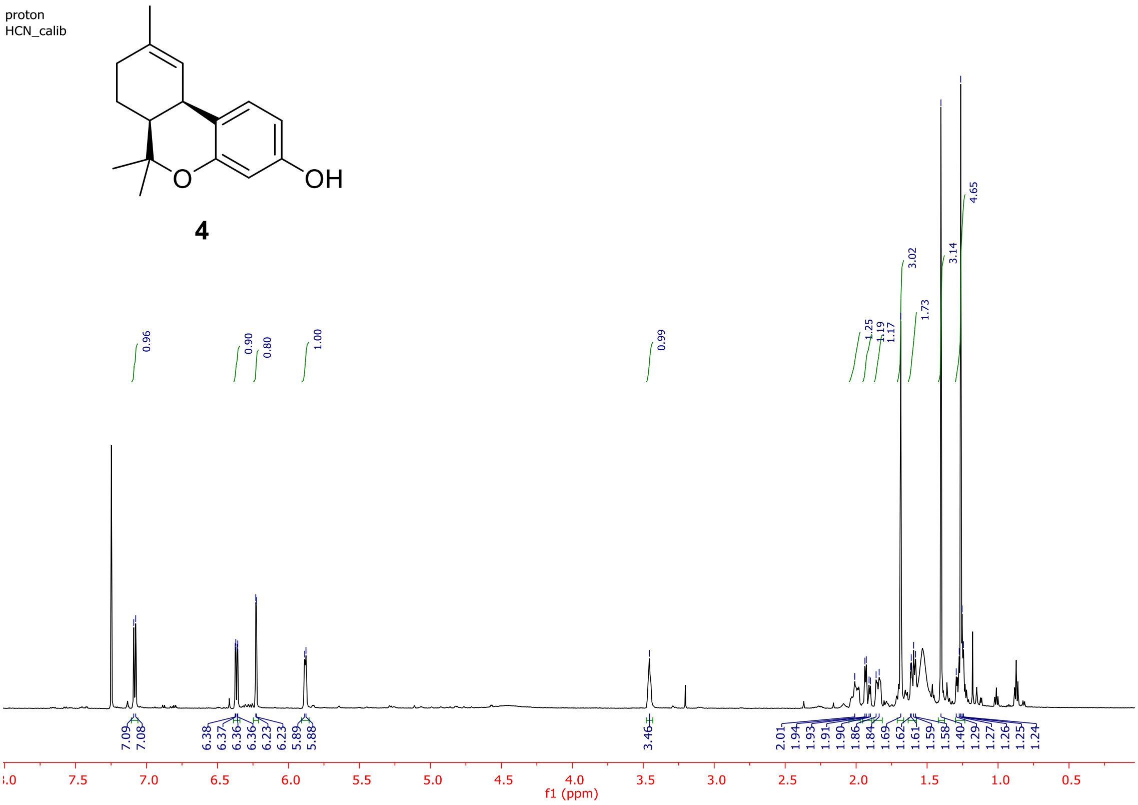

$^{13}C\{^{1}H\}$ NMR of **4** (151 MHz, $CDCl_3$)

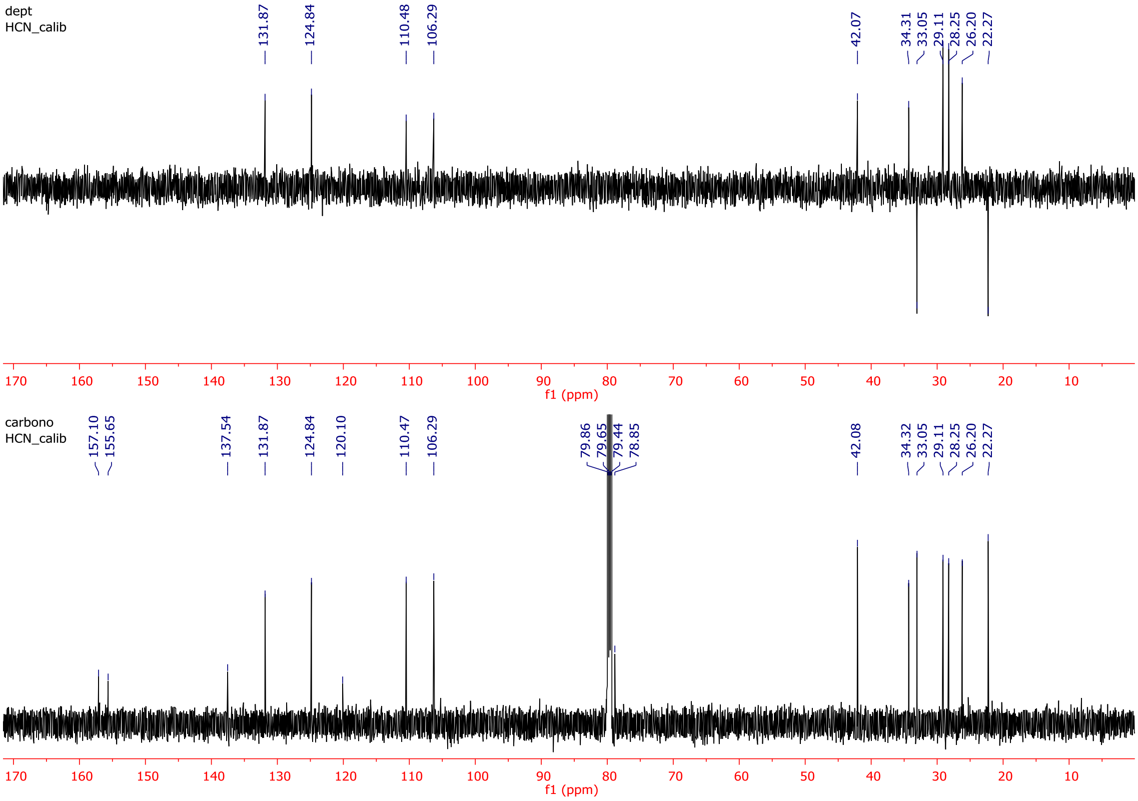

^{1}H NMR of **5** (500 MHz, $CDCl_3$)

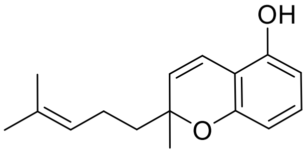

5

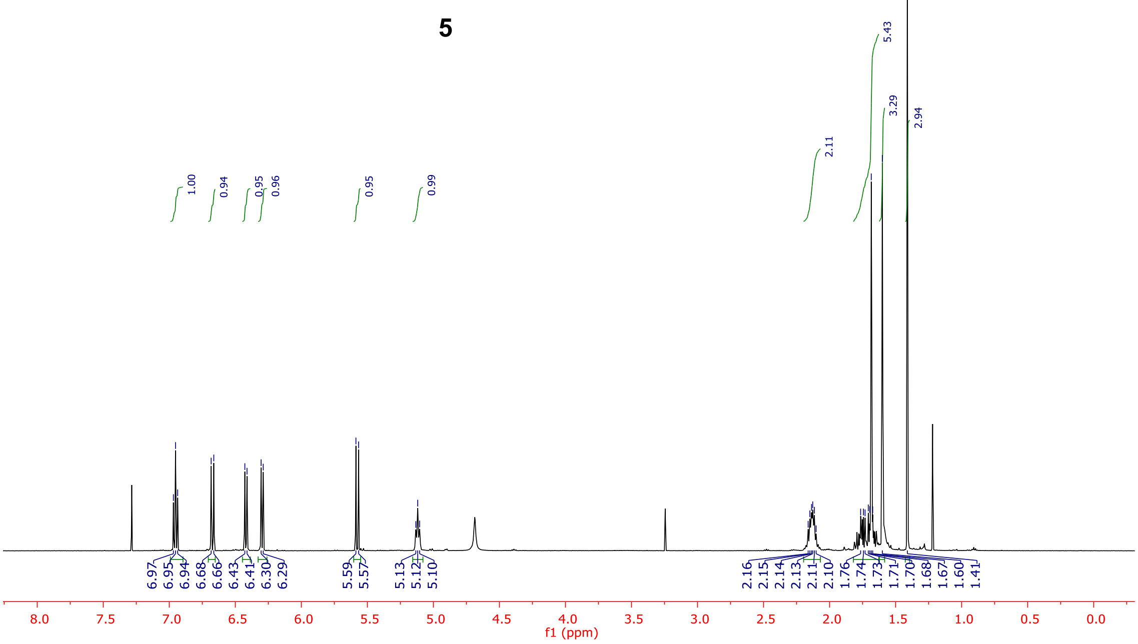

$^{13}C\{^{1}H\}$ NMR of **5** (126 MHz, $CDCl_3$)

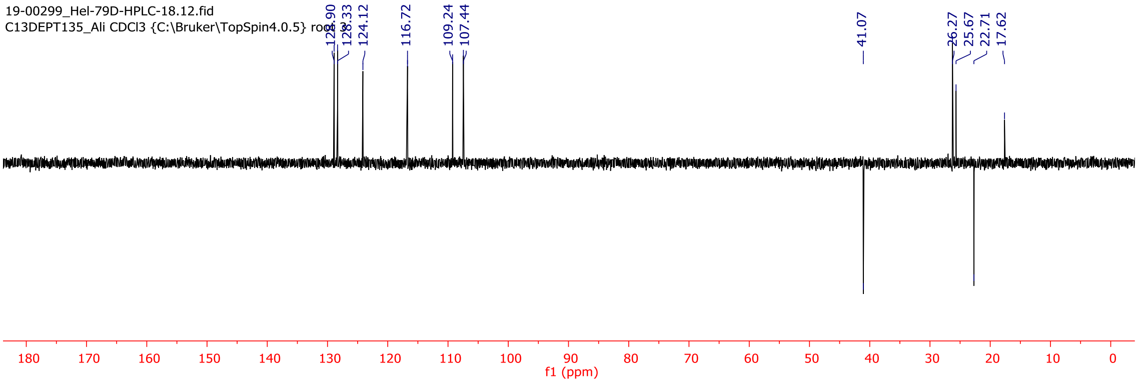

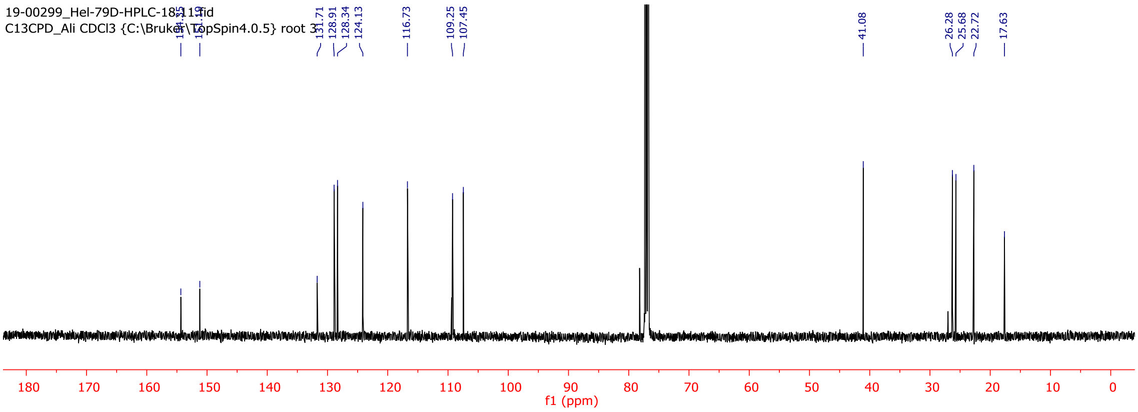

HSQC of 5 (500/126 MHz, $CDCl_3$)

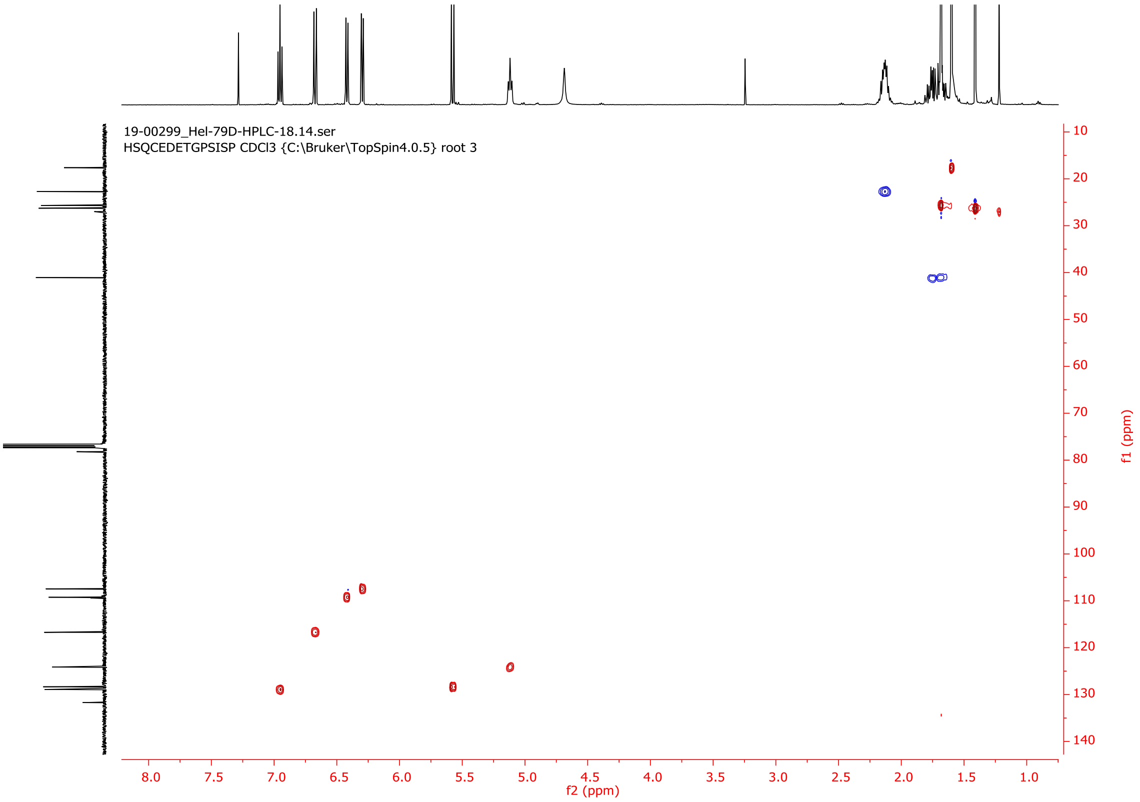

^{1}H NMR of **6** (500 MHz, $CDCl_3$)

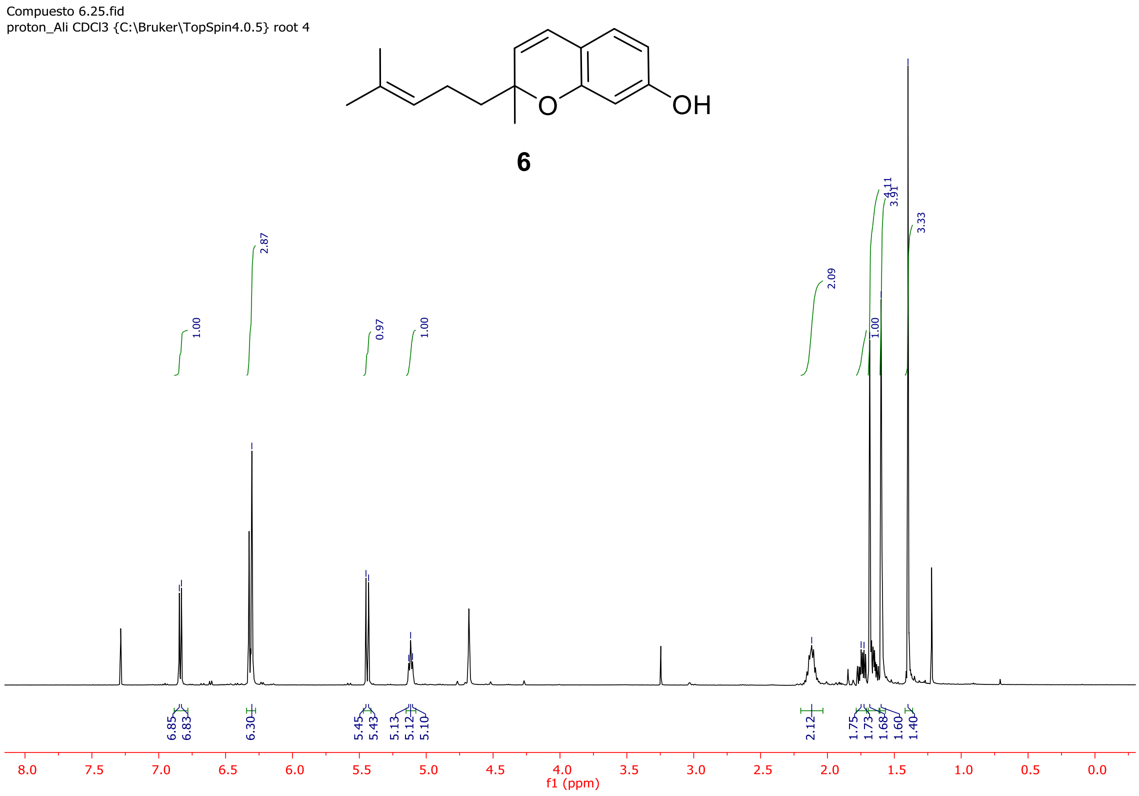

^{13}C{^{1}H} NMR of **6** (126 MHz, $CDCl_3$)

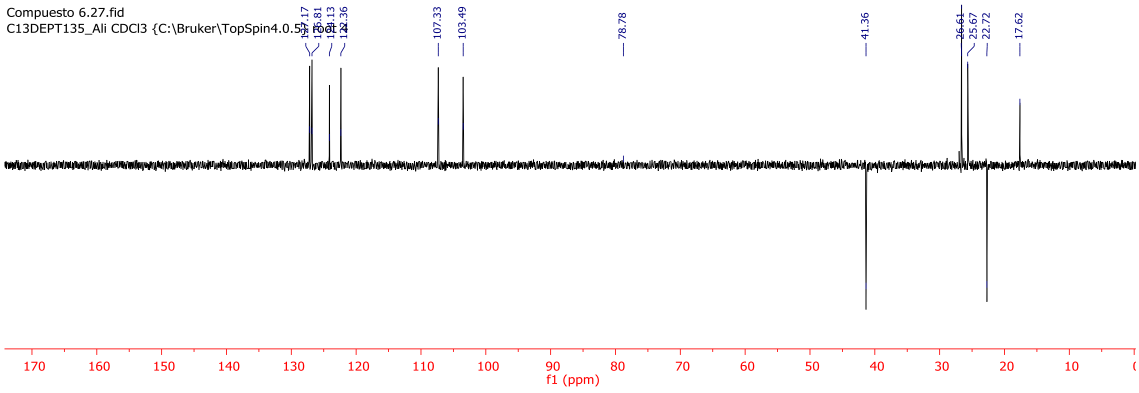

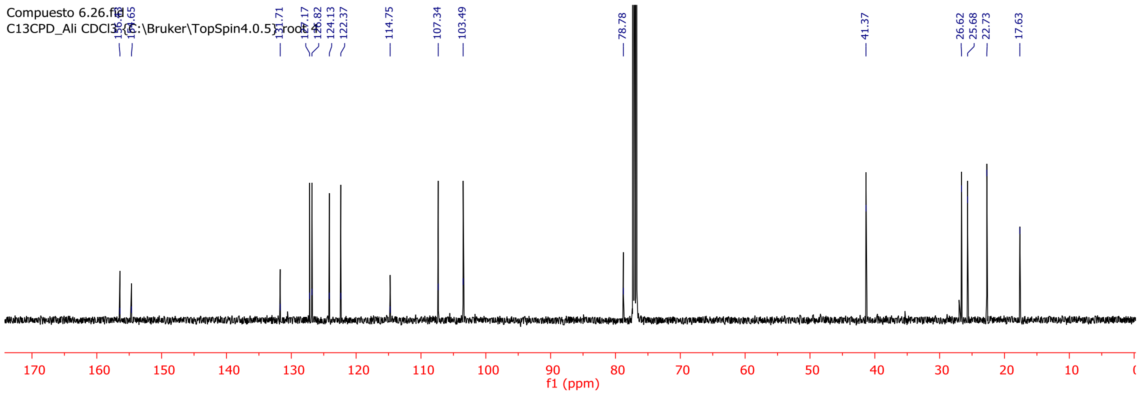

HSQC of 6 (500/126 MHz, $CDCl_3$)

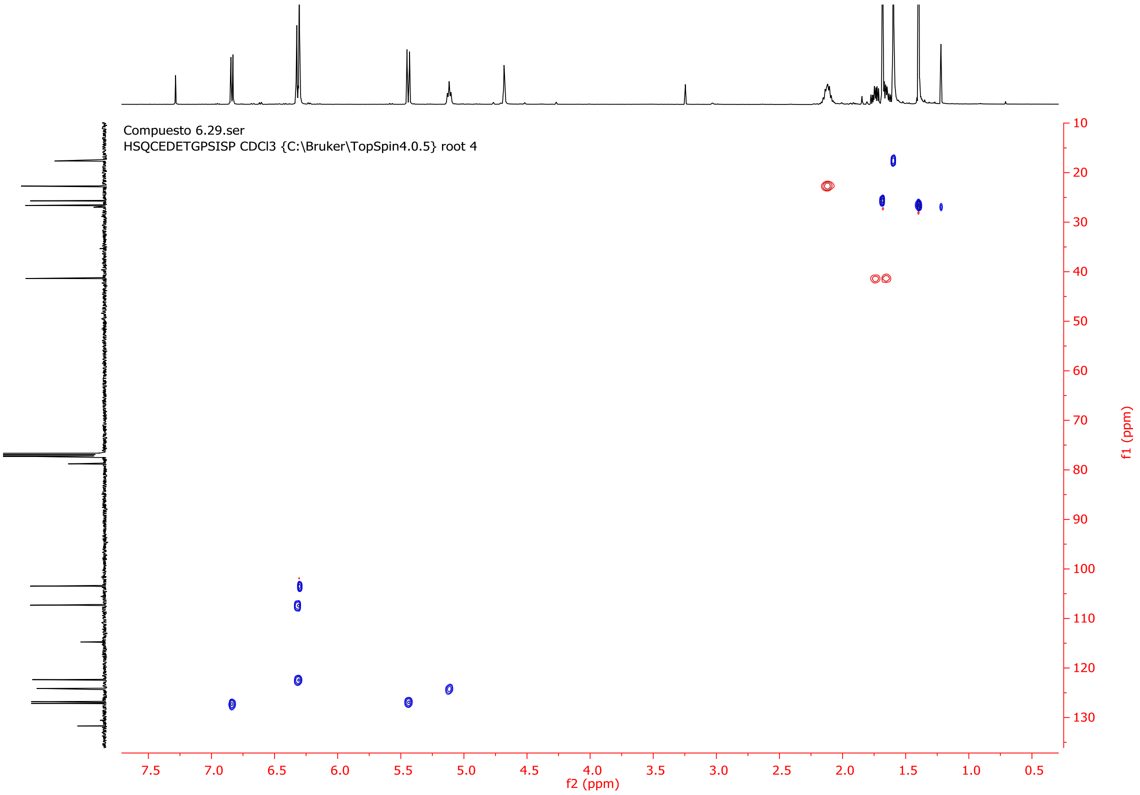

^{1}H NMR of **7** (500 MHz, $CDCl_3$)

AGP-25-A-HPLC3-6.10.fid
PROTON CDCl3 {C:\Bruker\TopSpin4.0.7} root 2

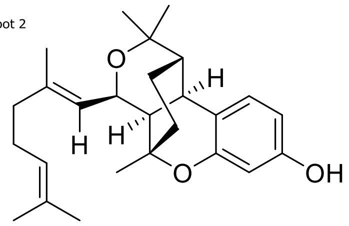

7

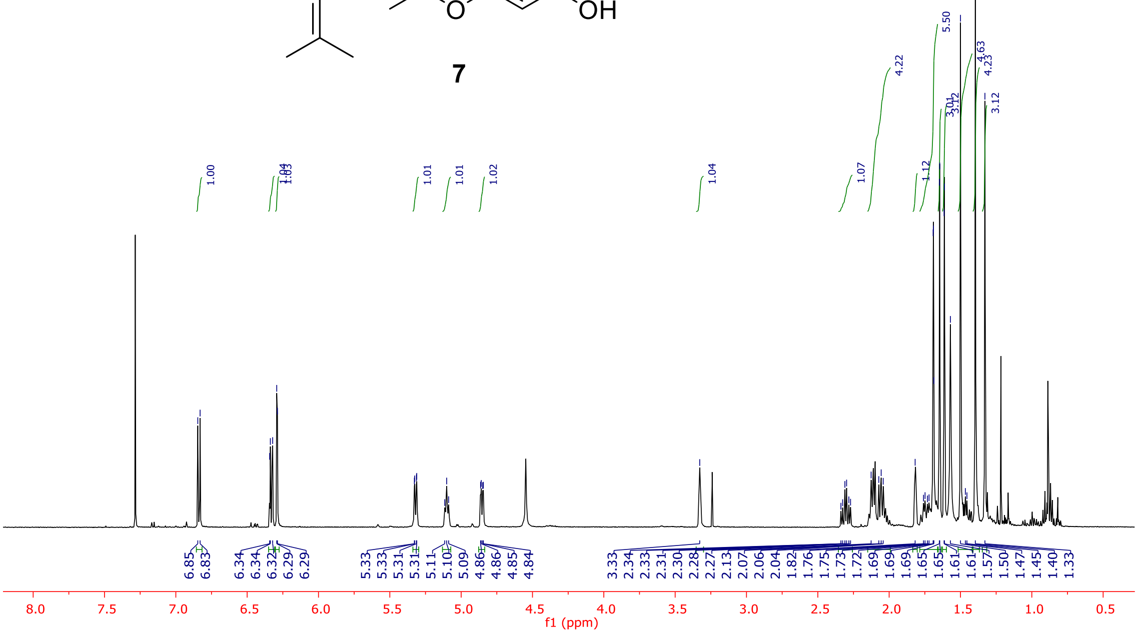

$^{13}C\{^{1}H\}$ NMR of **7** (126 MHz, $CDCl_3$)

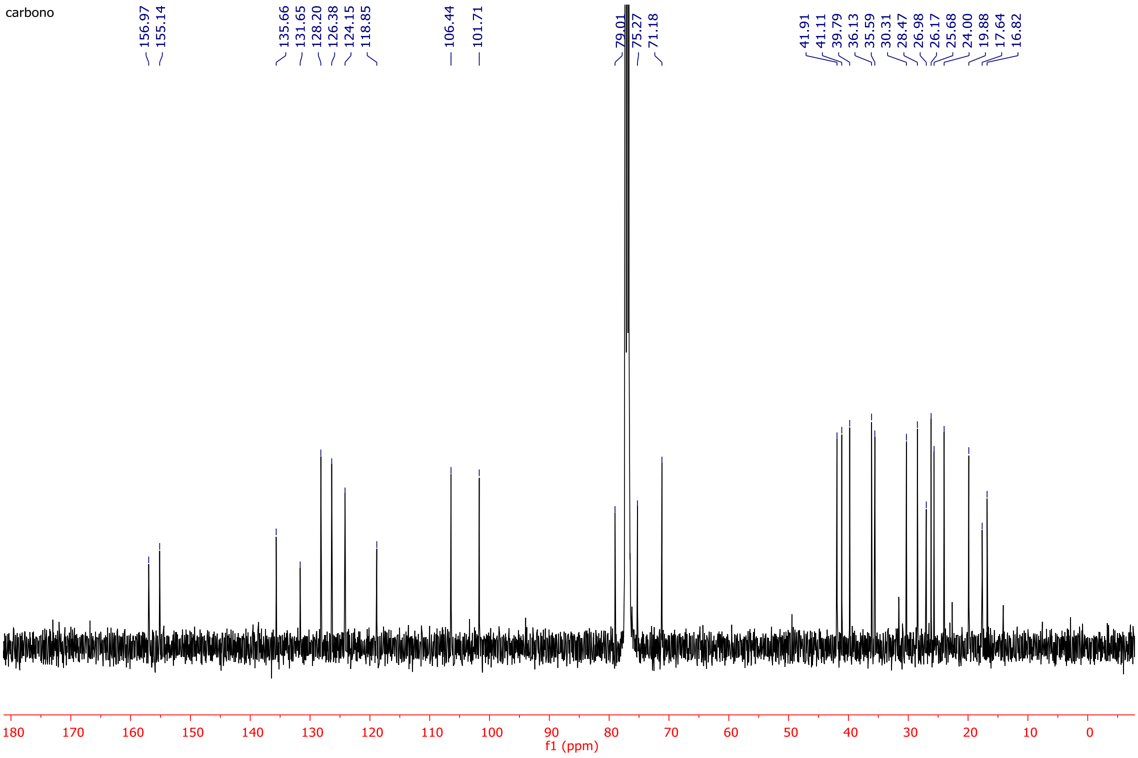

COSY of 7 (500 MHz, $CDCl_3$)

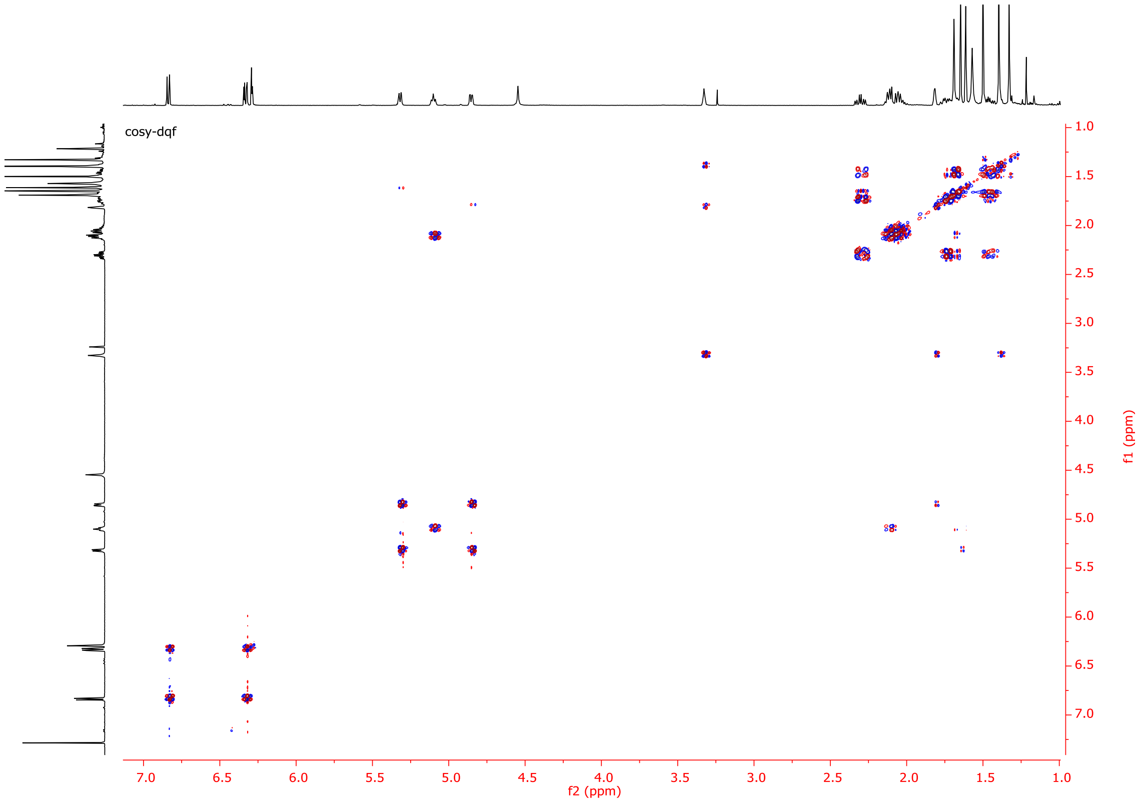

HSQC of 7 (500/126 MHz, $CDCl_3$)

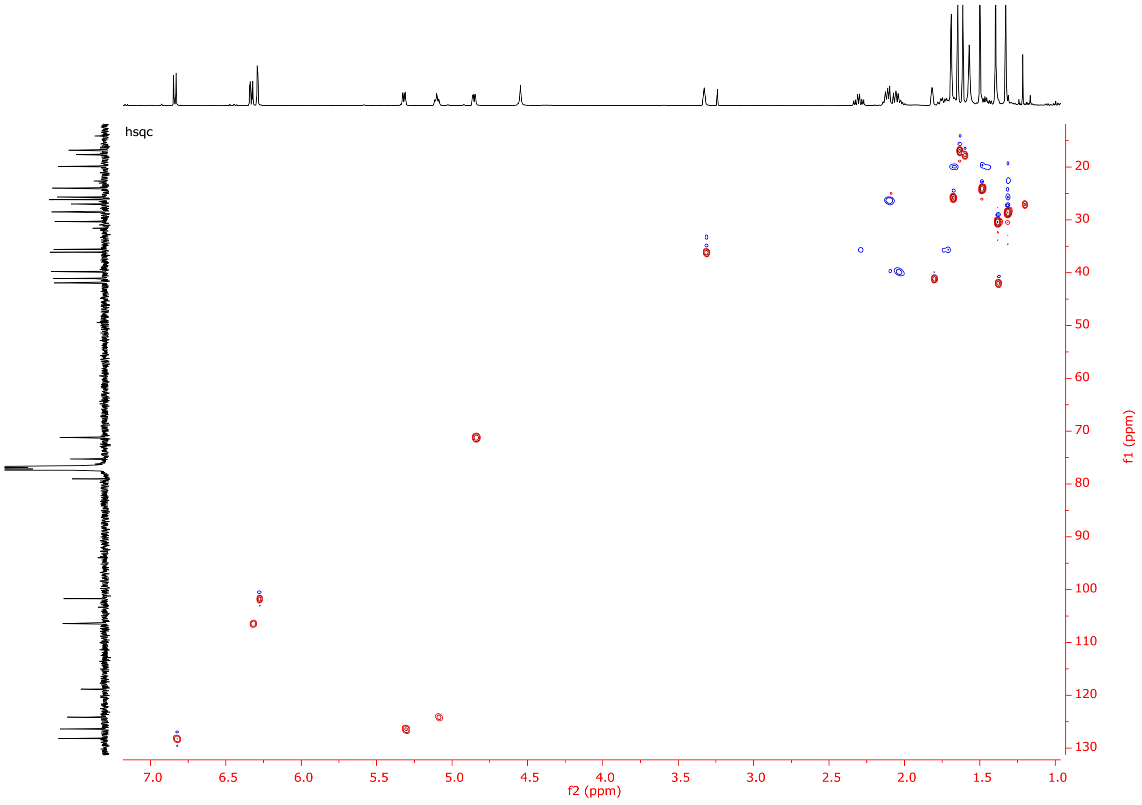

HMBC of 7 (500/126 MHz, $CDCl_3$)

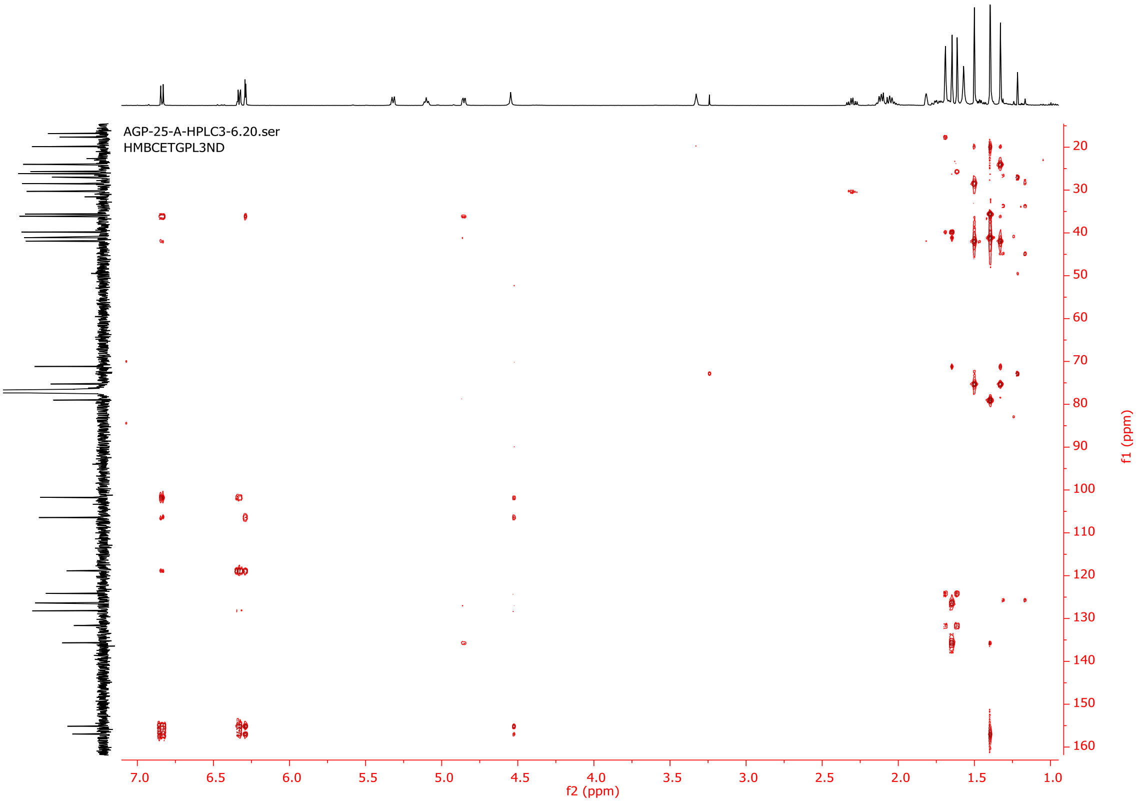

1D TOCSY of 7 (500 MHz, $CDCl_3$)

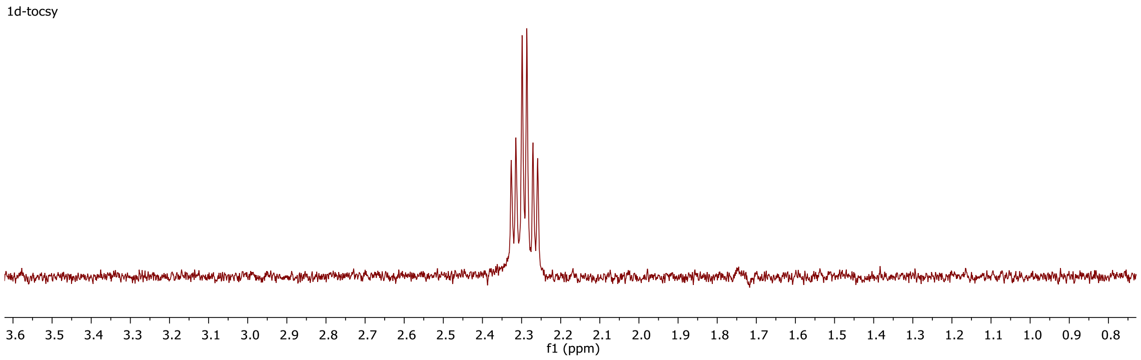

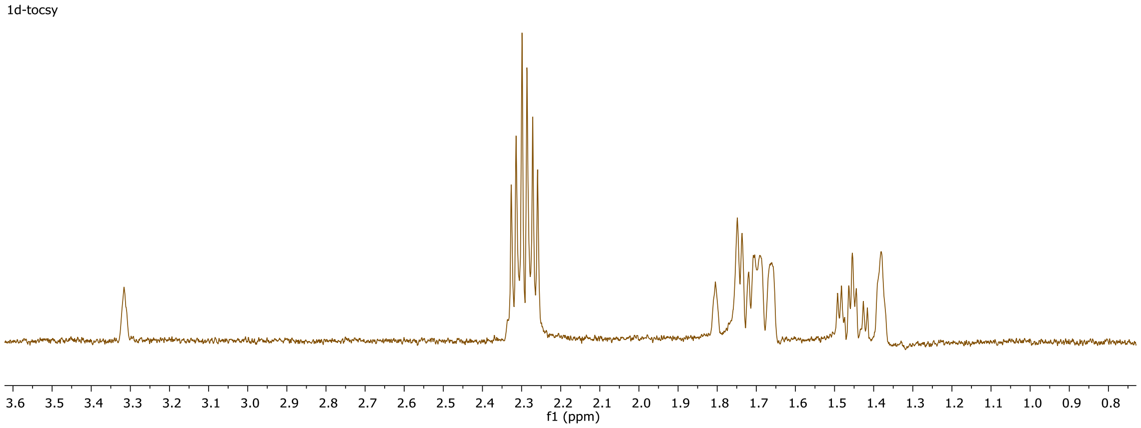

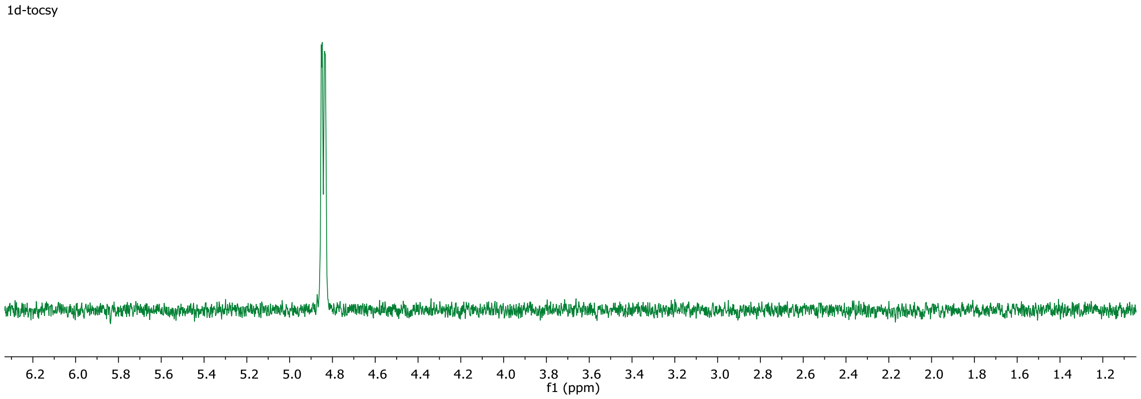

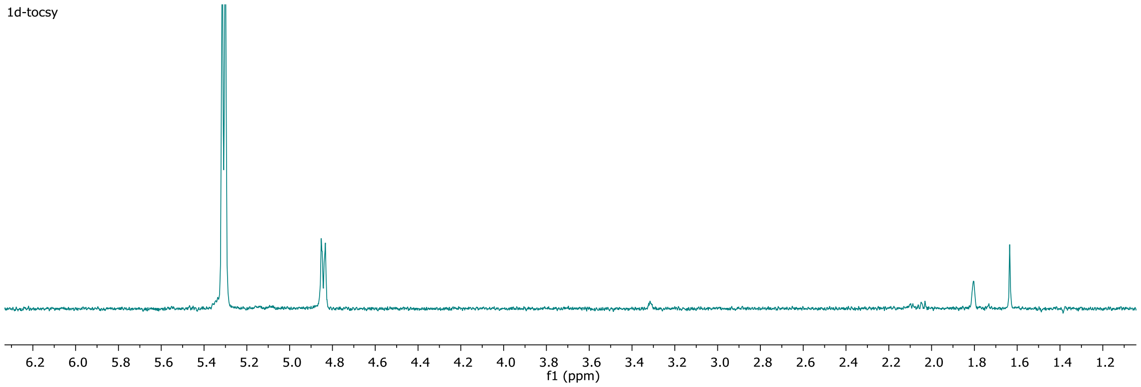

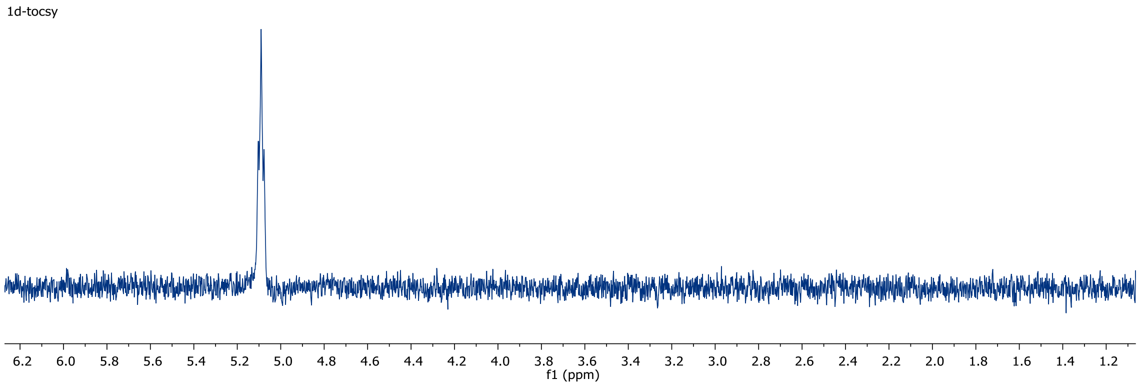
1d-tocsy
6.2 6.0 5.8 5.6 5.4 5.2 5.0 4.8 4.6 4.4 4.2 4.0 3.8 3.6 3.4 3.2 3.0 2.8 2.6 2.4 2.2 2.0 1.8 1.6 1.4 1.2
f1 (ppm)

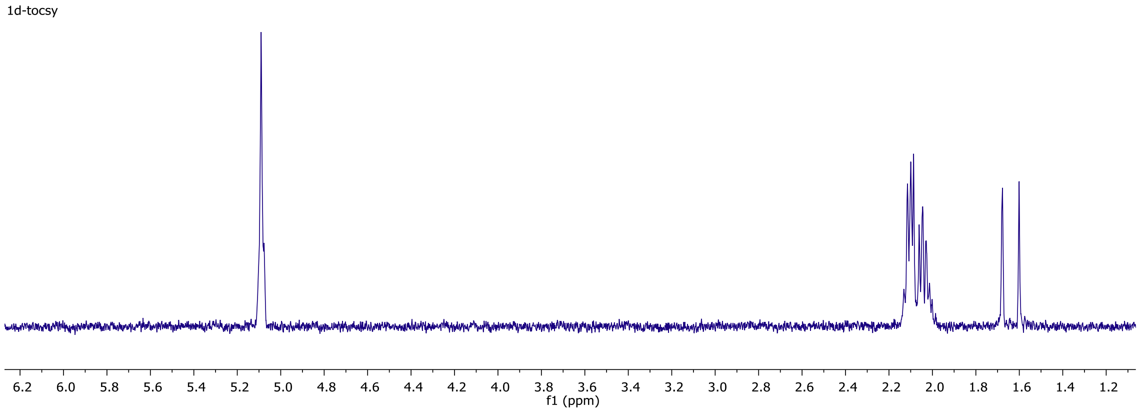
1d-tocsy
6.2 6.0 5.8 5.6 5.4 5.2 5.0 4.8 4.6 4.4 4.2 4.0 3.8 3.6 3.4 3.2 3.0 2.8 2.6 2.4 2.2 2.0 1.8 1.6 1.4 1.2
f1 (ppm)

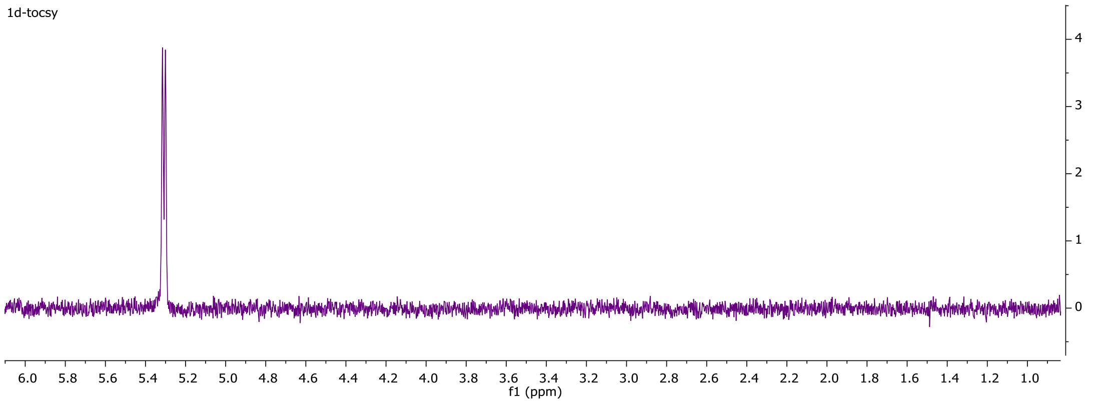
1d-tocsy
4 3 2 1 0
6.0 5.8 5.6 5.4 5.2 5.0 4.8 4.6 4.4 4.2 4.0 3.8 3.6 3.4 3.2 3.0 2.8 2.6 2.4 2.2 2.0 1.8 1.6 1.4 1.2 1.0
f1 (ppm)

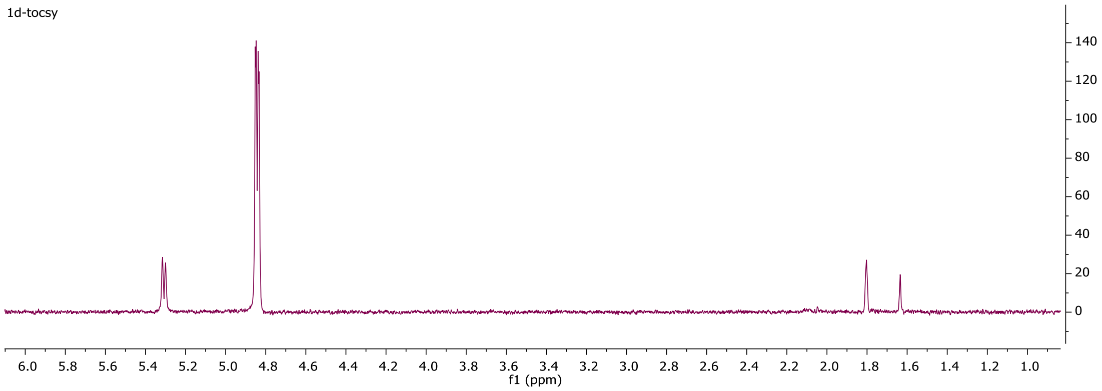
1d-tocsy
140 120 100 80 60 40 20 0
6.0 5.8 5.6 5.4 5.2 5.0 4.8 4.6 4.4 4.2 4.0 3.8 3.6 3.4 3.2 3.0 2.8 2.6 2.4 2.2 2.0 1.8 1.6 1.4 1.2 1.0
f1 (ppm)

1D NOESY of 7 (500 MHz, $CDCl_3$)

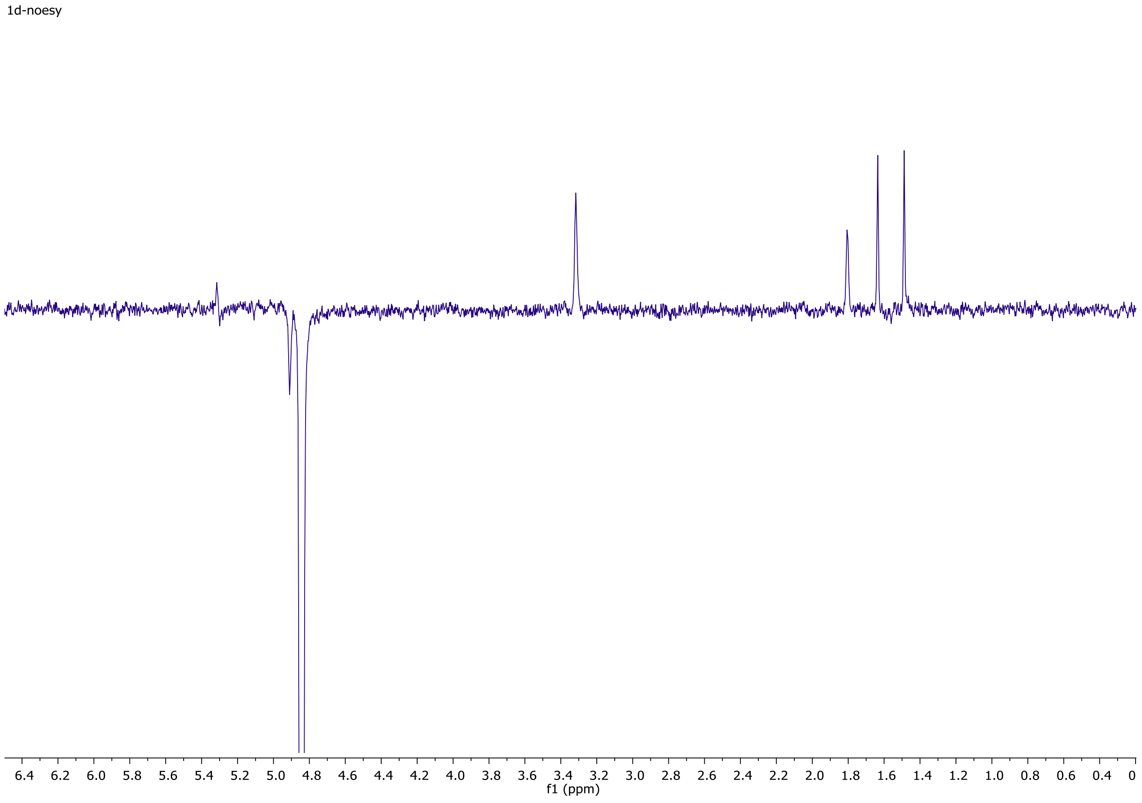

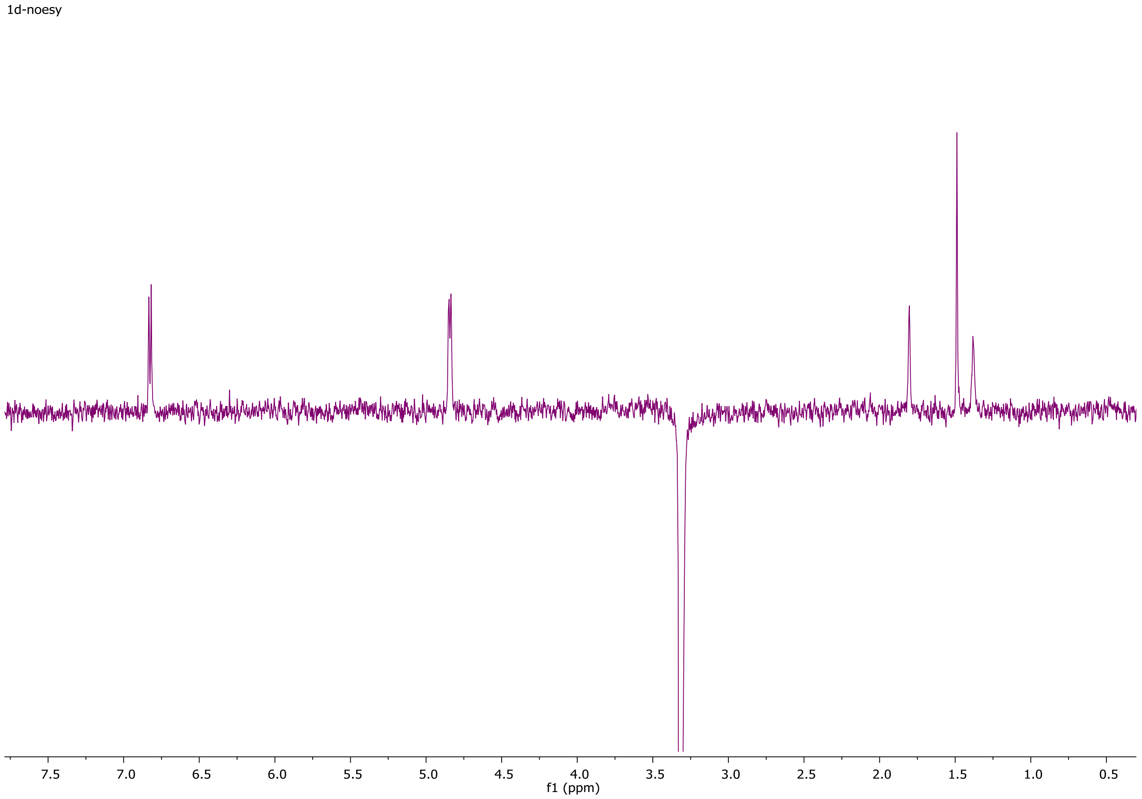

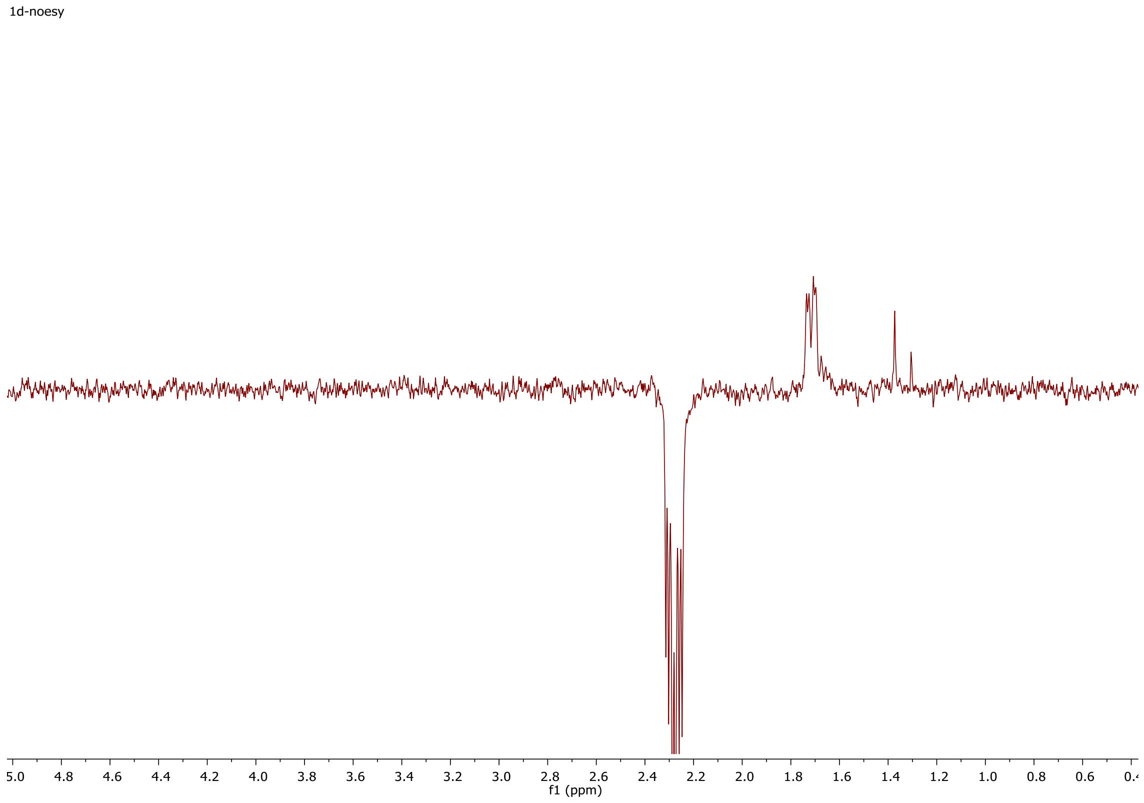
1d-noesy
5.0 4.8 4.6 4.4 4.2 4.0 3.8 3.6 3.4 3.2 3.0 2.8 2.6 2.4 2.2 2.0 1.8 1.6 1.4 1.2 1.0 0.8 0.6
f1 (ppm)

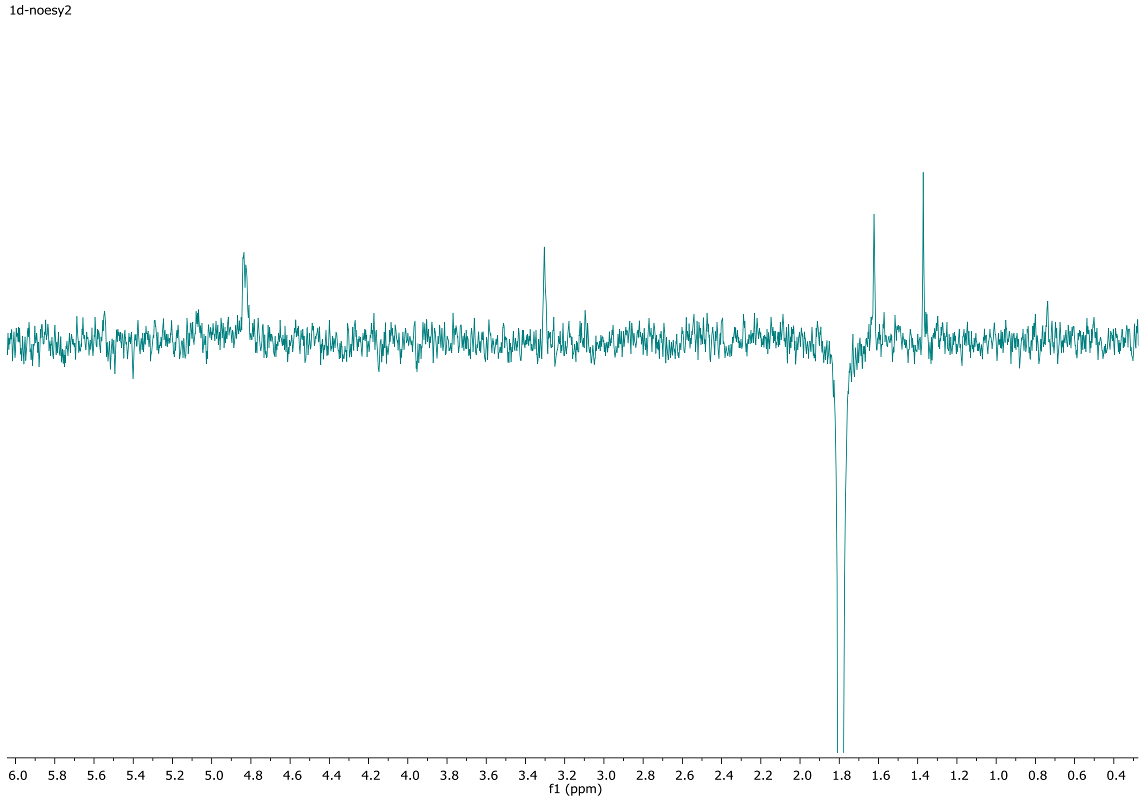
1d-noesy2
6.0 5.8 5.6 5.4 5.2 5.0 4.8 4.6 4.4 4.2 4.0 3.8 3.6 3.4 3.2 3.0 2.8 2.6 2.4 2.2 2.0 1.8 1.6 1.4 1.2 1.0 0.8 0.6 0.4
f1 (ppm)

^{1}H NMR of **7a** (500 MHz, $CDCl_3$)

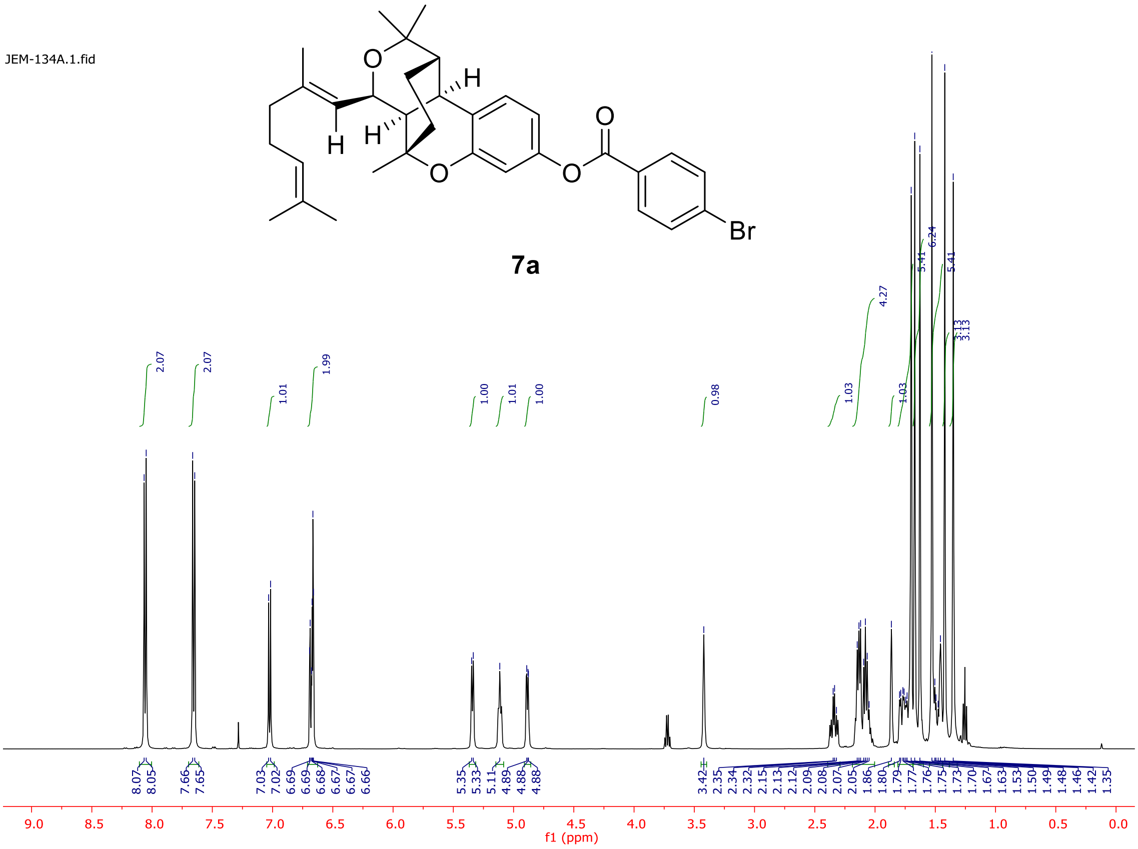

$^{13}C\{^1H\}$ NMR of **7a** (126 MHz, $CDCl_3$)

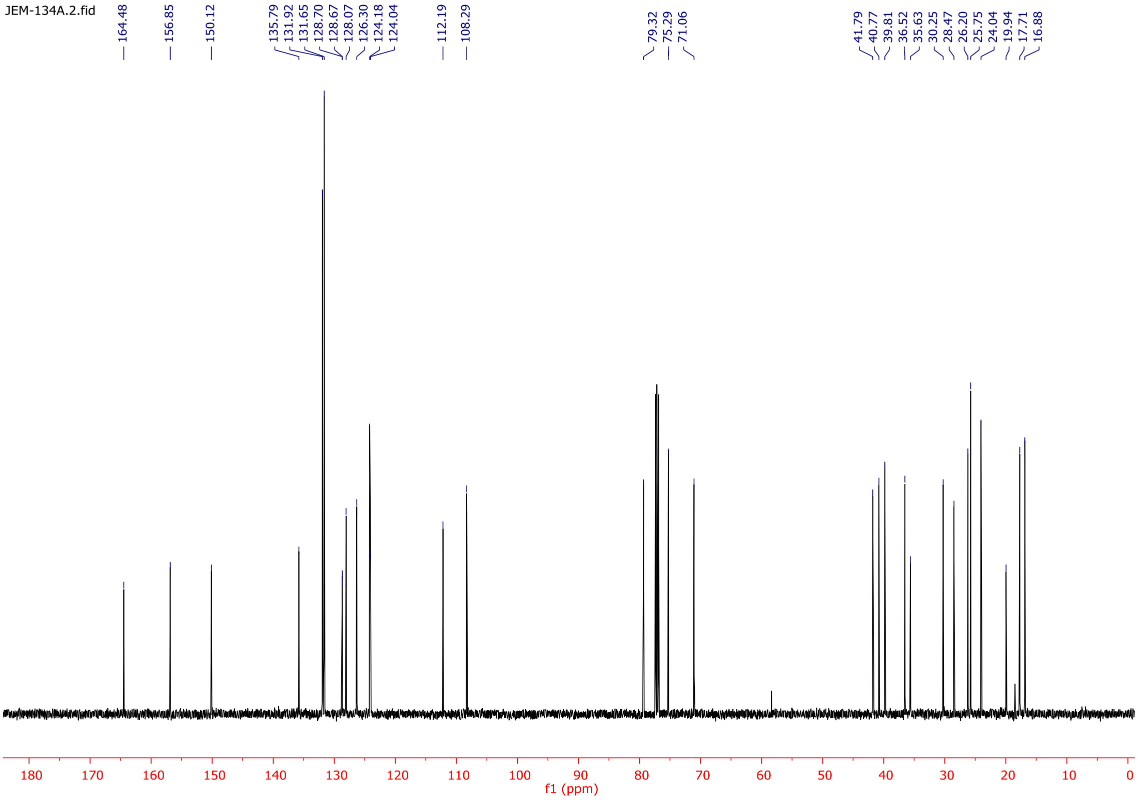

COSY of 7a (500 MHz, $CDCl_3$)

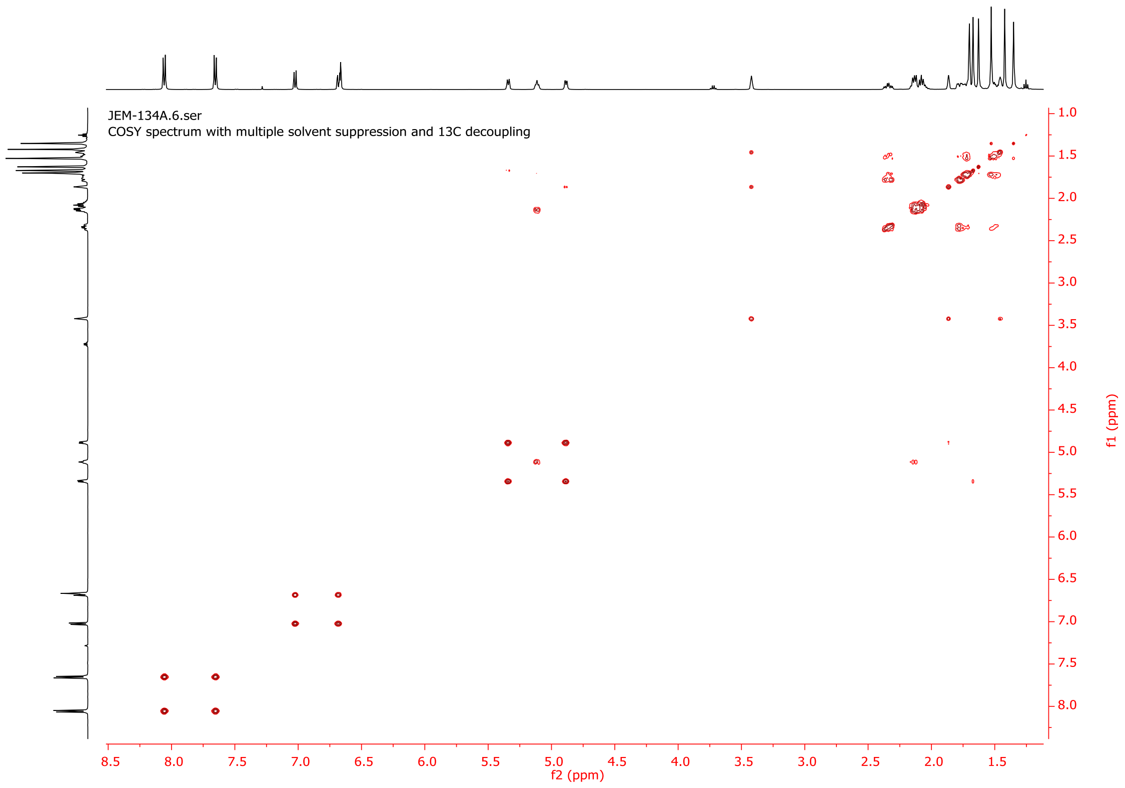

HSQC of 7a (500/126 MHz, $CDCl_3$)

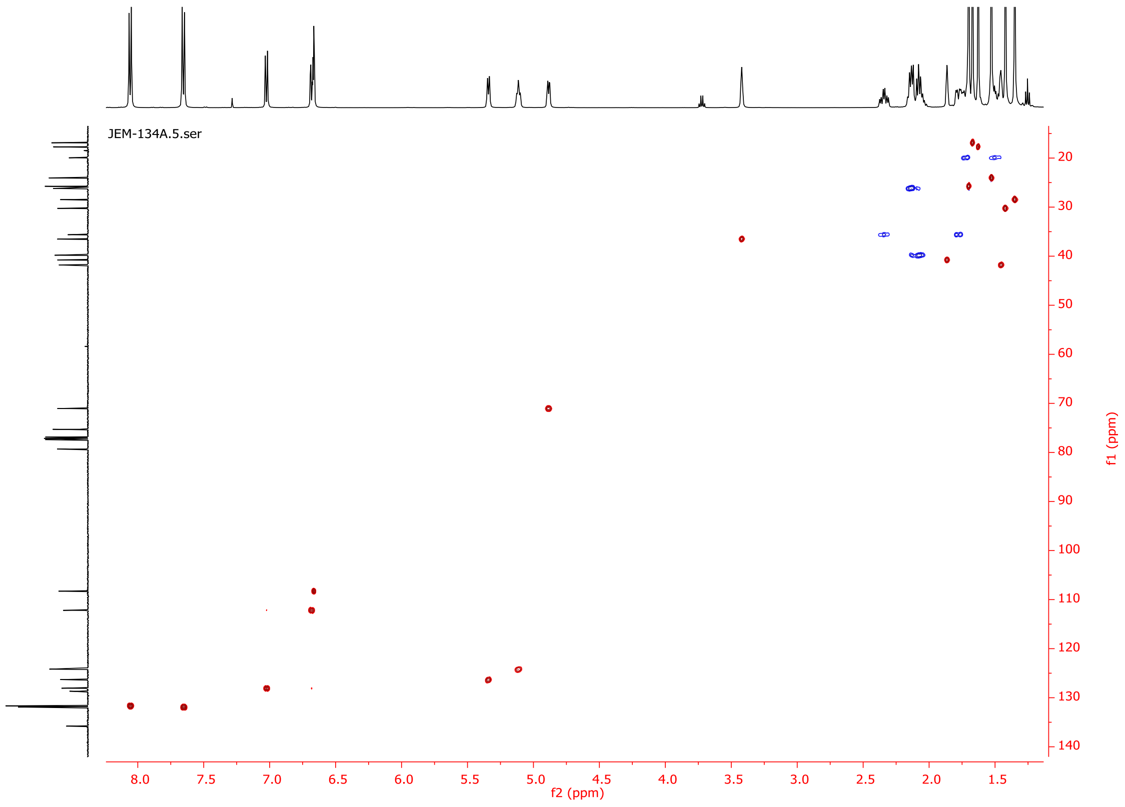

HMBC of 7a (500/126 MHz, $CDCl_3$)

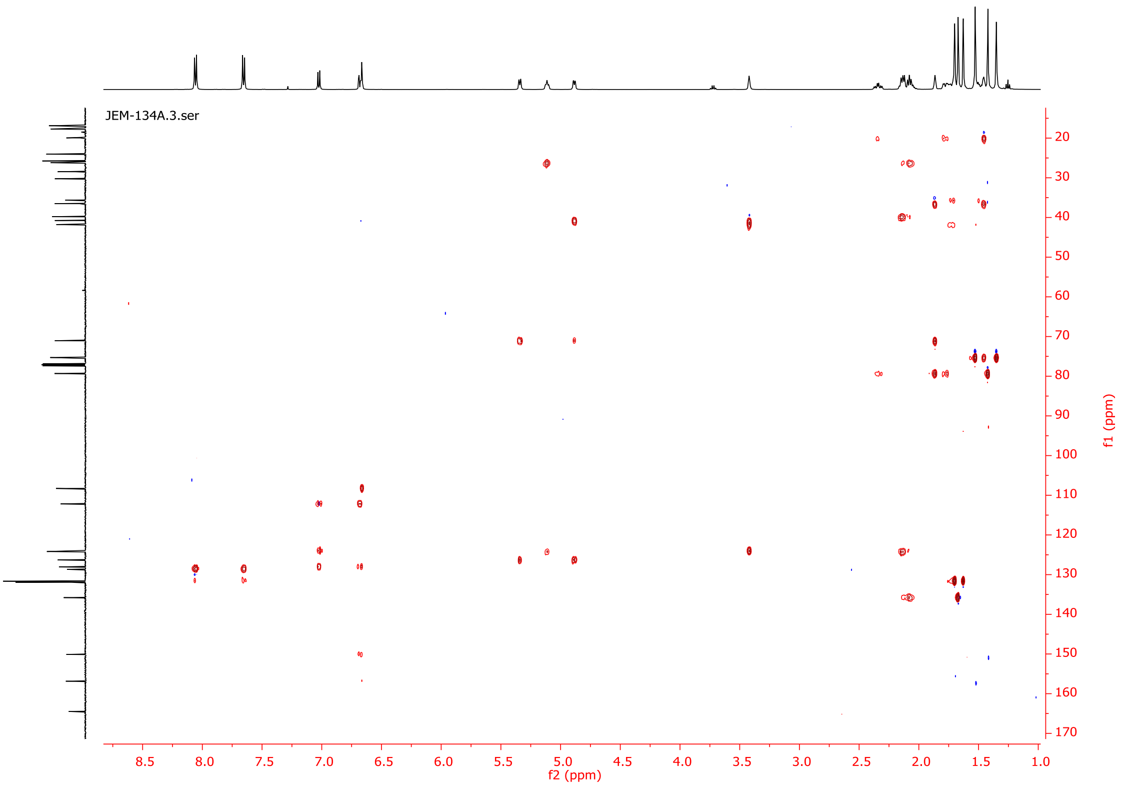

^{1}H NMR of **9** (600 MHz, $CDCl_3$)

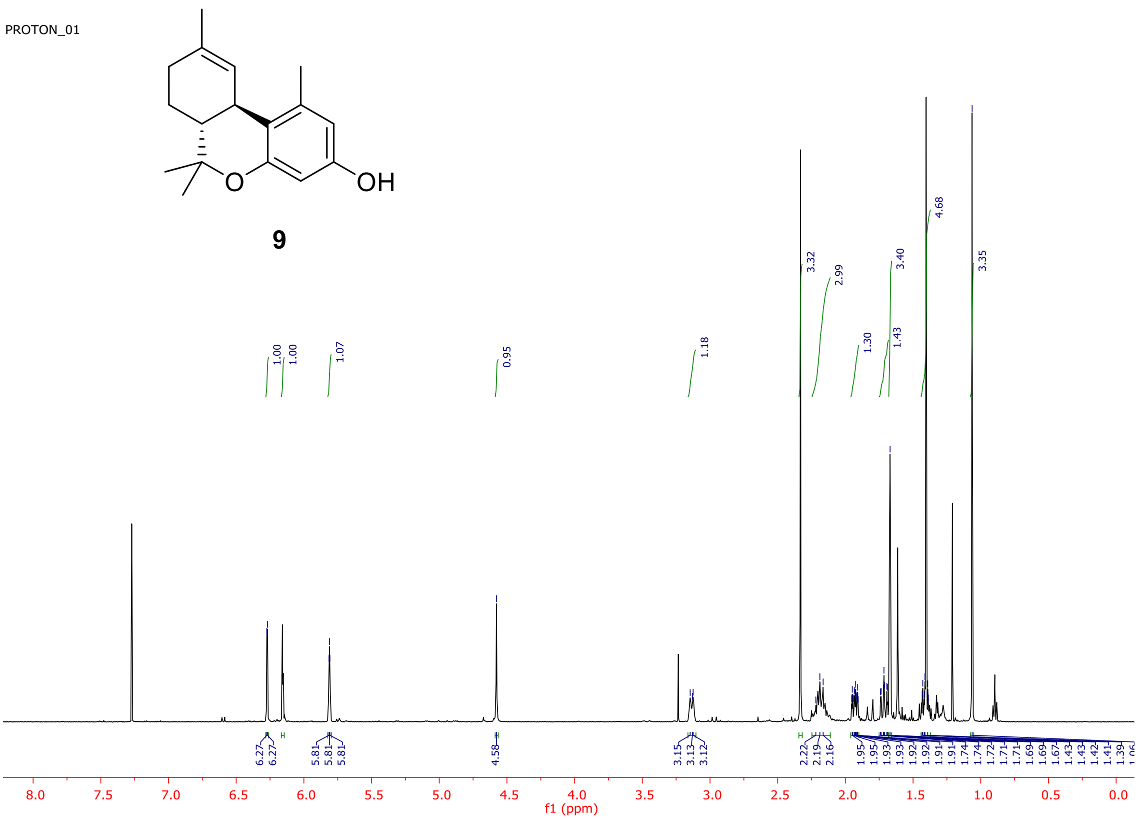

^{13}C{^{1}H} NMR of **9** (151 MHz, $CDCl_3$)

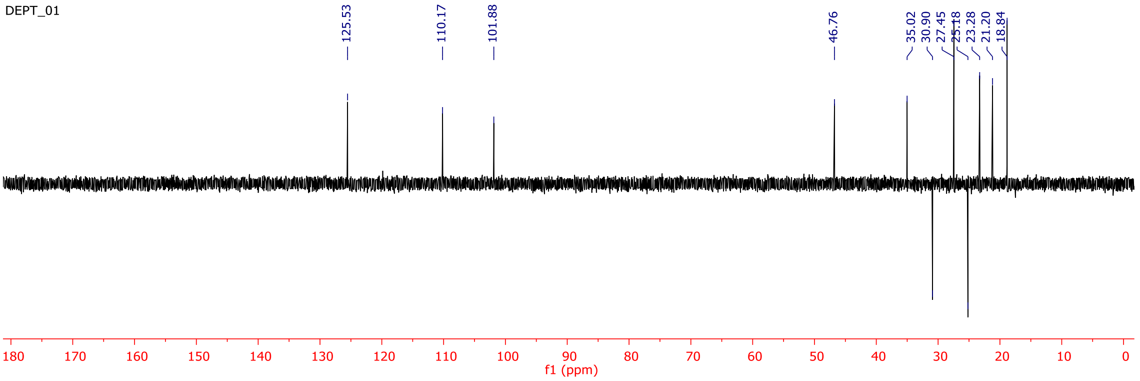

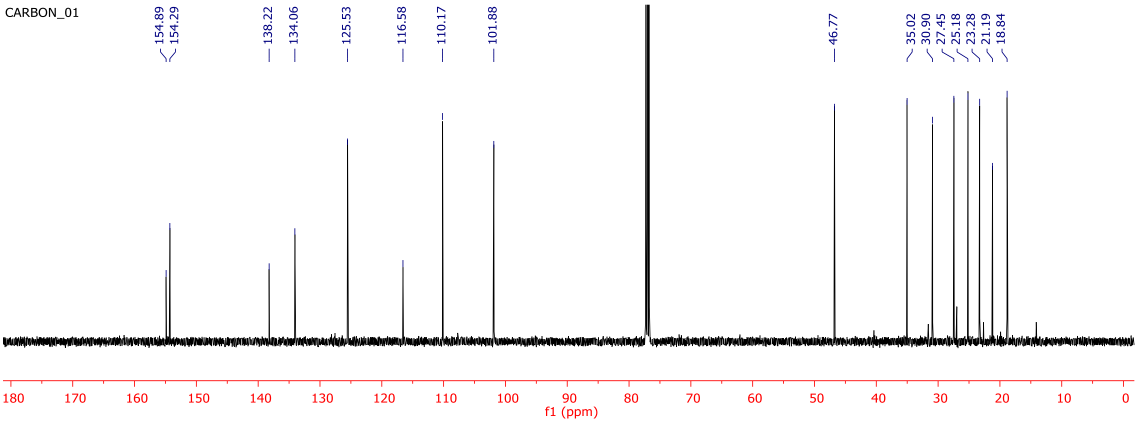

HSQC of 9 (600 MHz, $CDCl_3$)

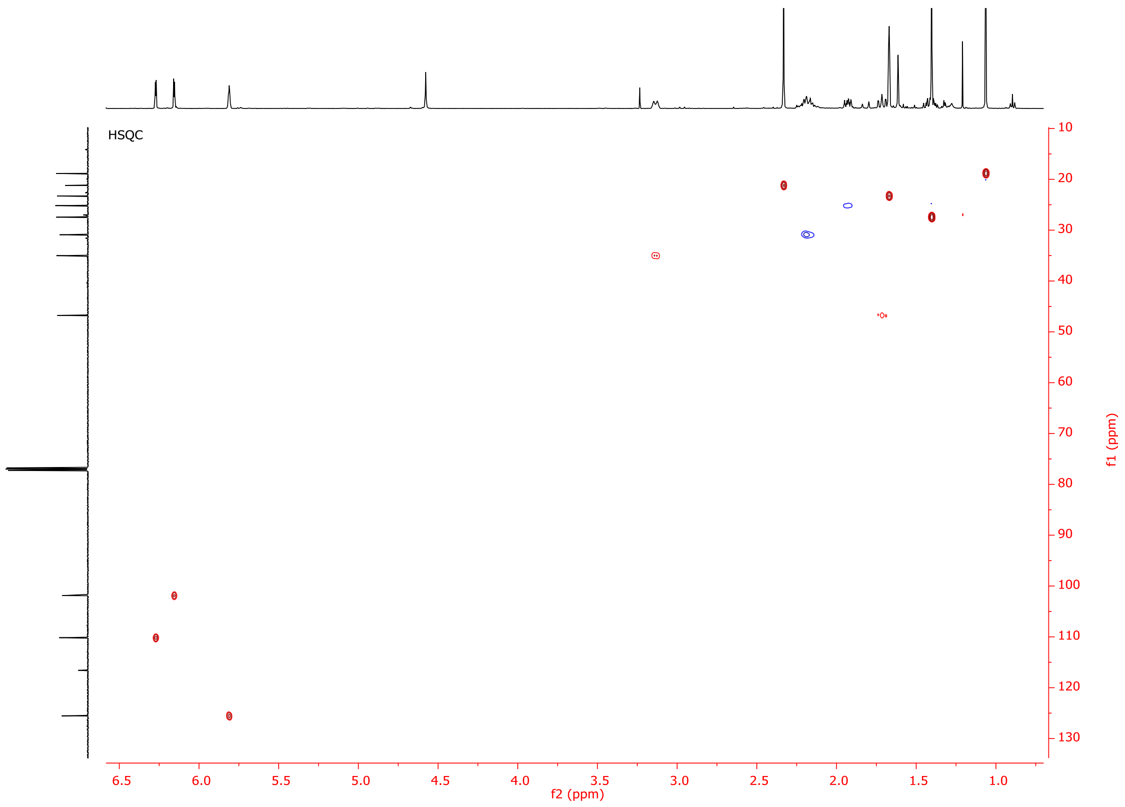

^{1}H NMR of **10** (600 MHz, $CDCl_3$)

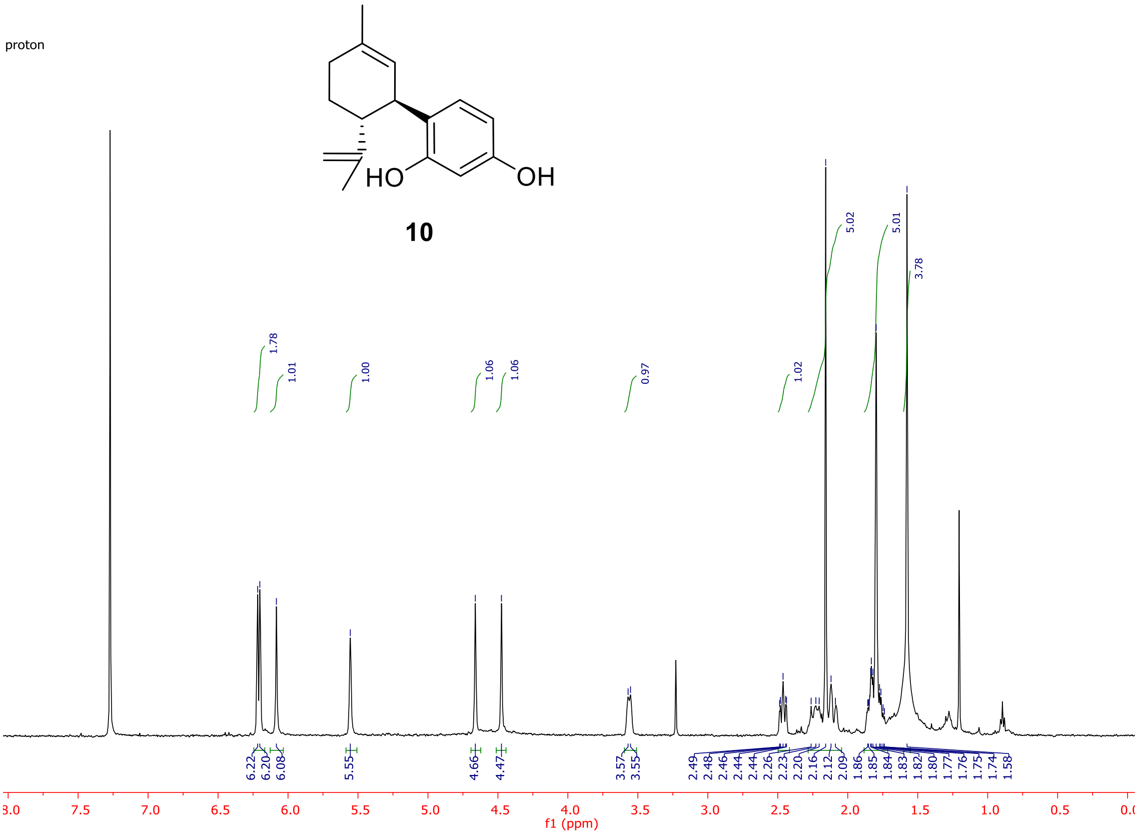

^{13}C{^{1}H} NMR of **10** (151 MHz, $CDCl_3$)

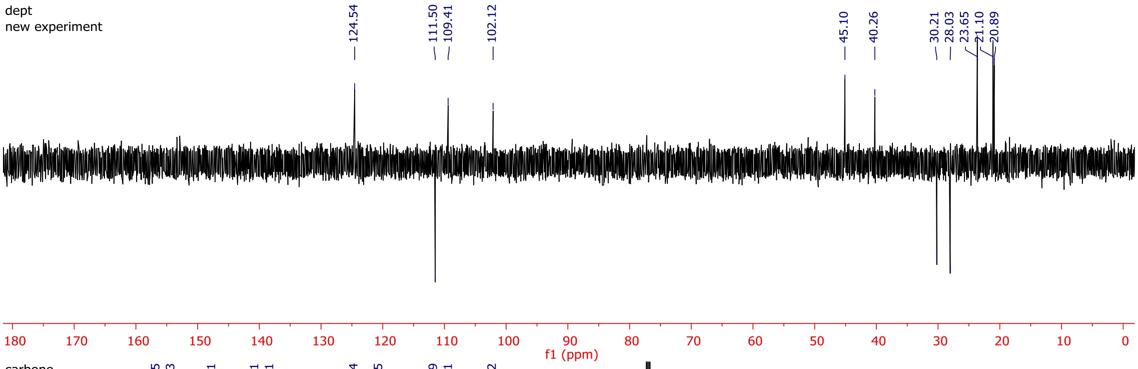

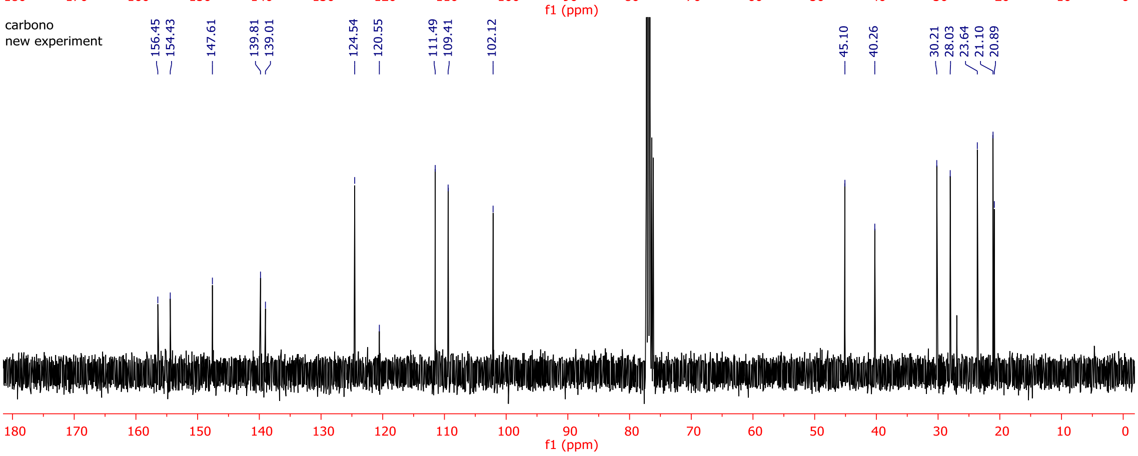

HSQC of 10 (600/151 MHz, $CDCl_3$)

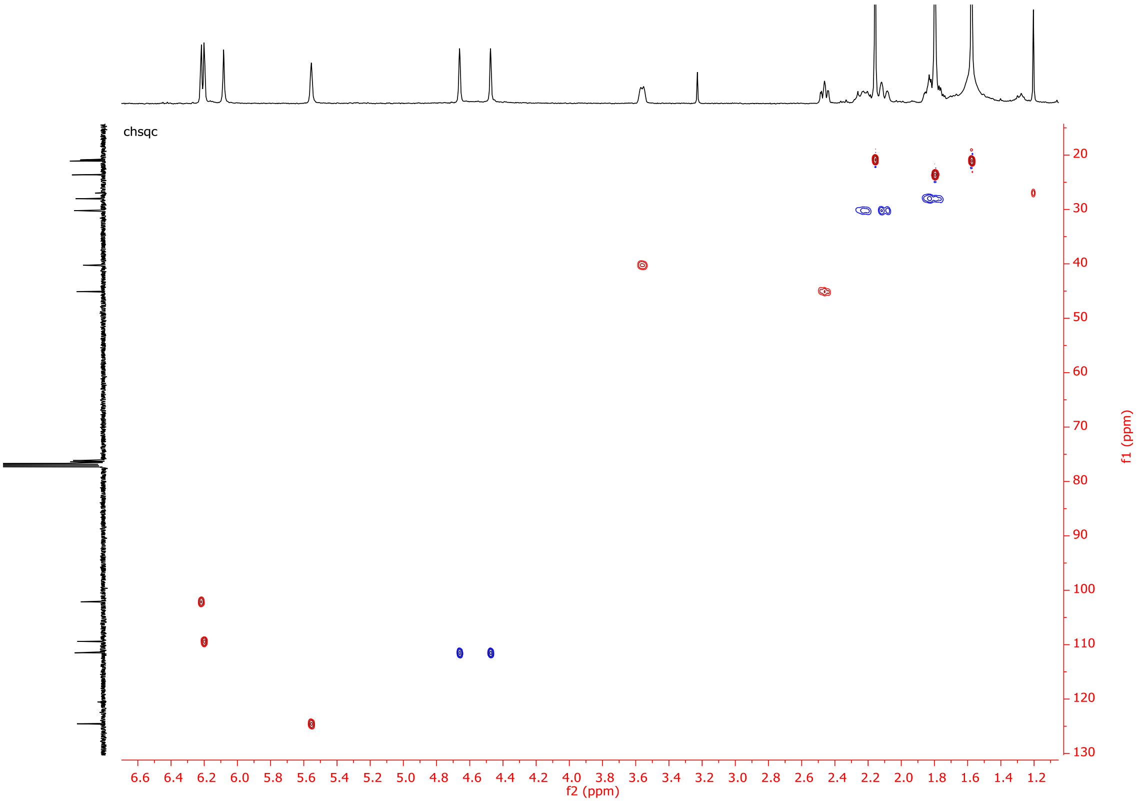

^{1}H NMR of **11** (300 MHz, $CDCl_3$)

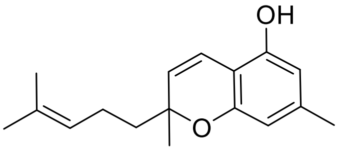

11

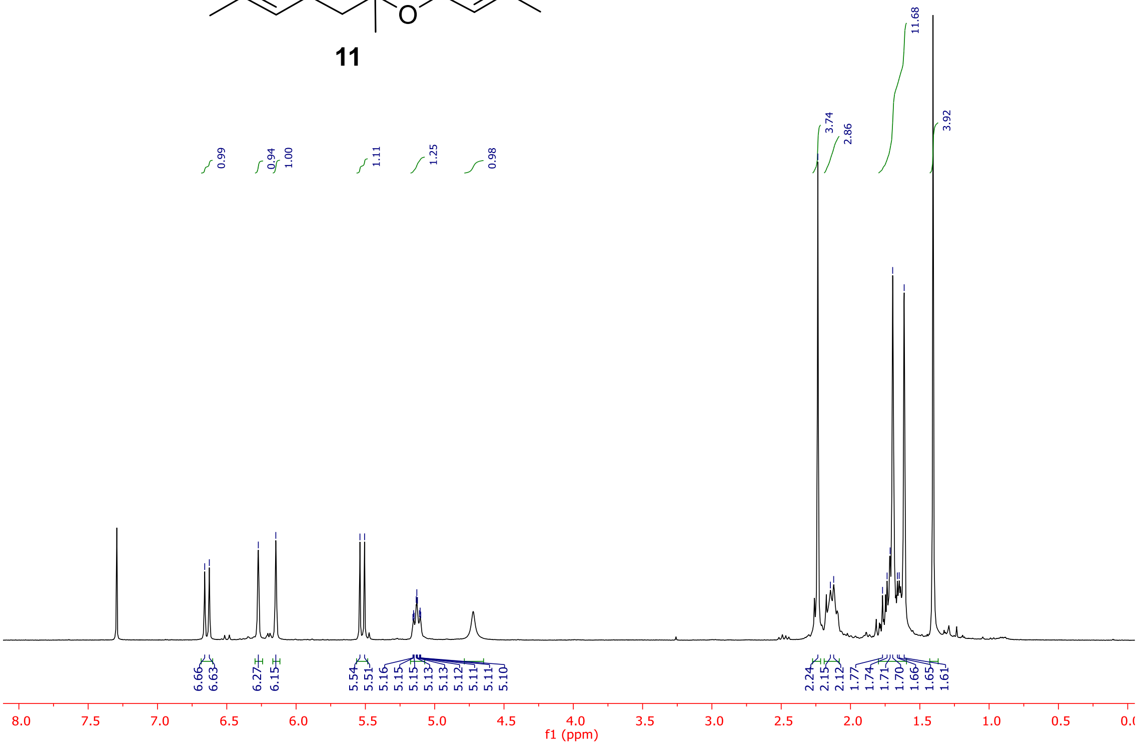

$^{13}C\{^{1}H\}$ NMR of **11** (75 MHz, $CDCl_3$)

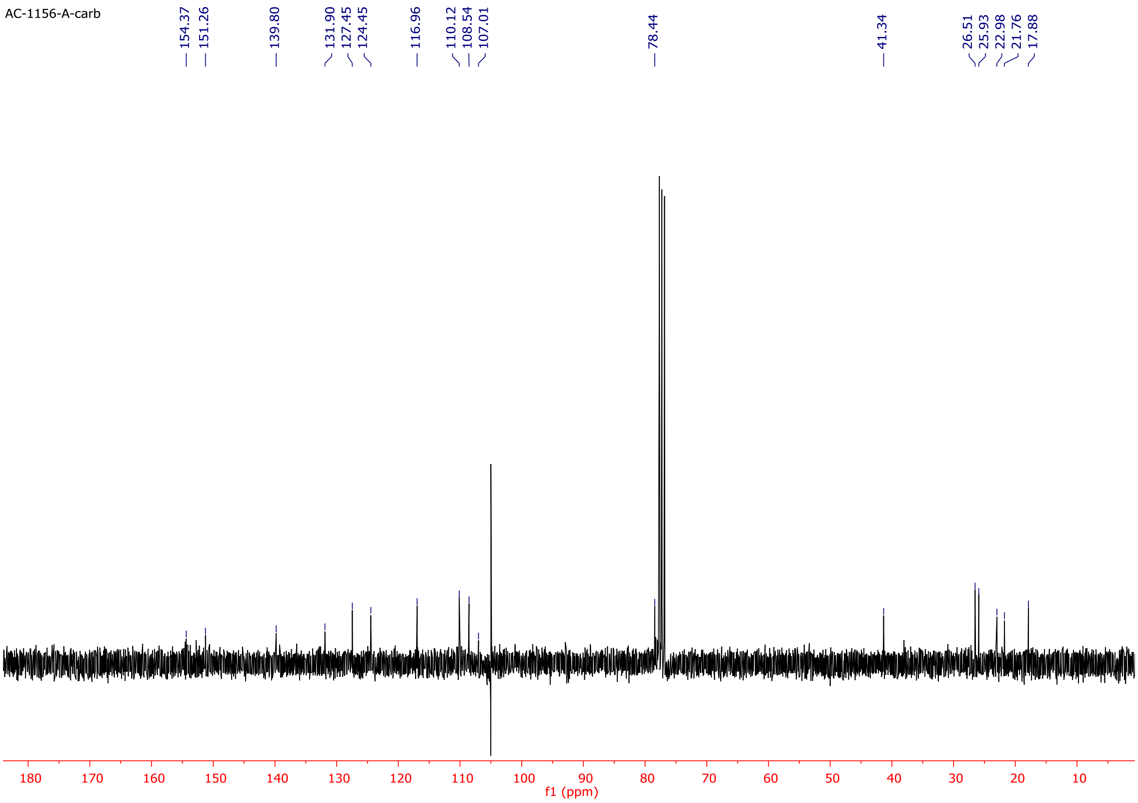

^{1}H NMR of **14** (600 MHz, $CDCl_3$)

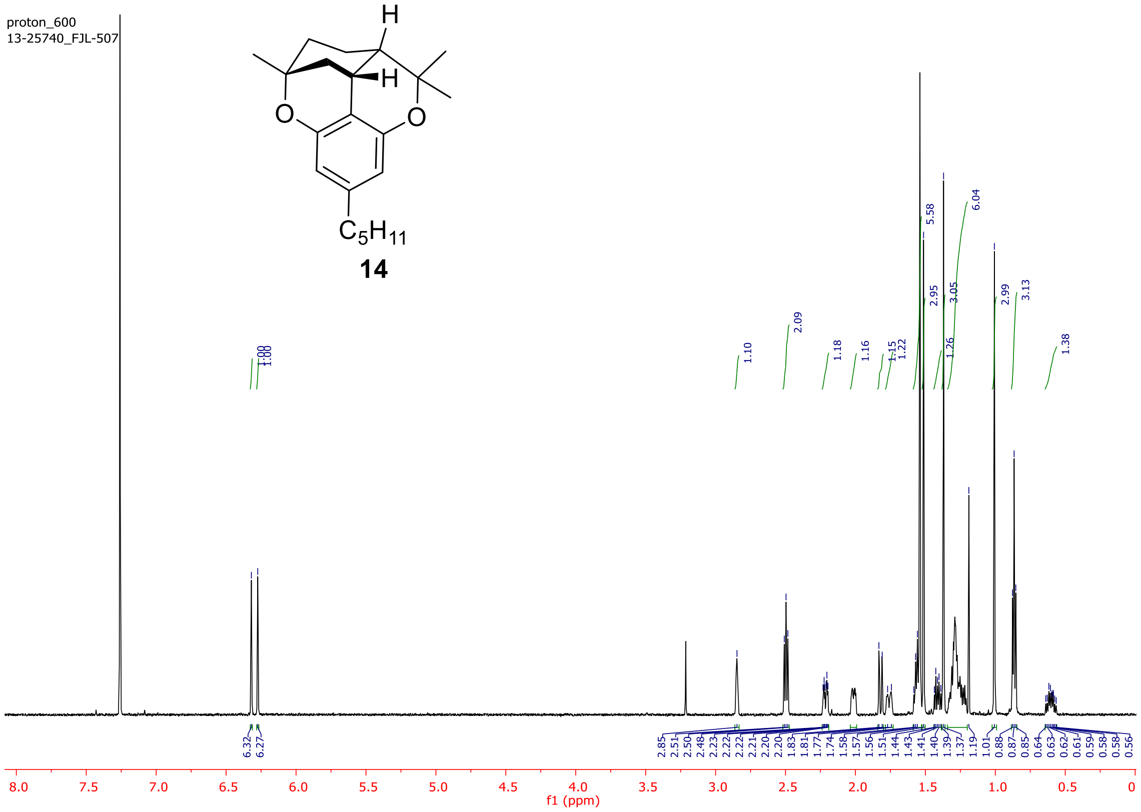

^{13}C{^{1}H} NMR of **14** (151 MHz, $CDCl_3$)

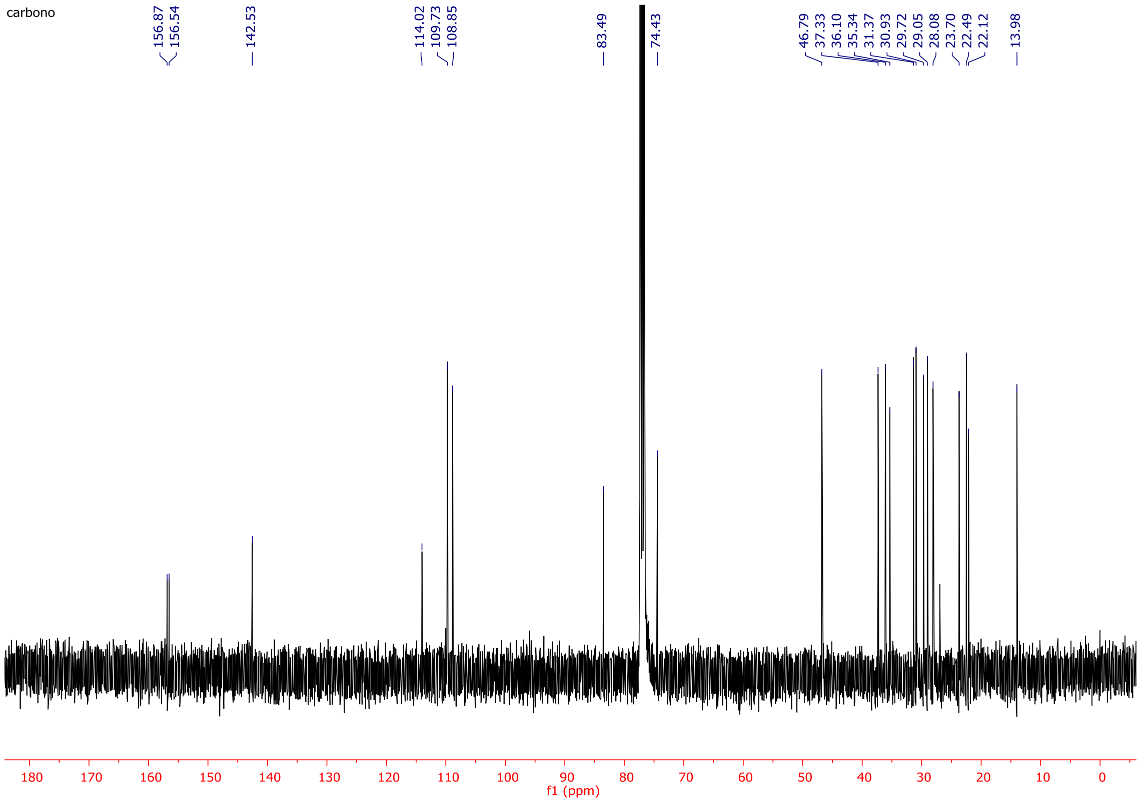

HSQC of 14 (600/151 MHz, $CDCl_3$)

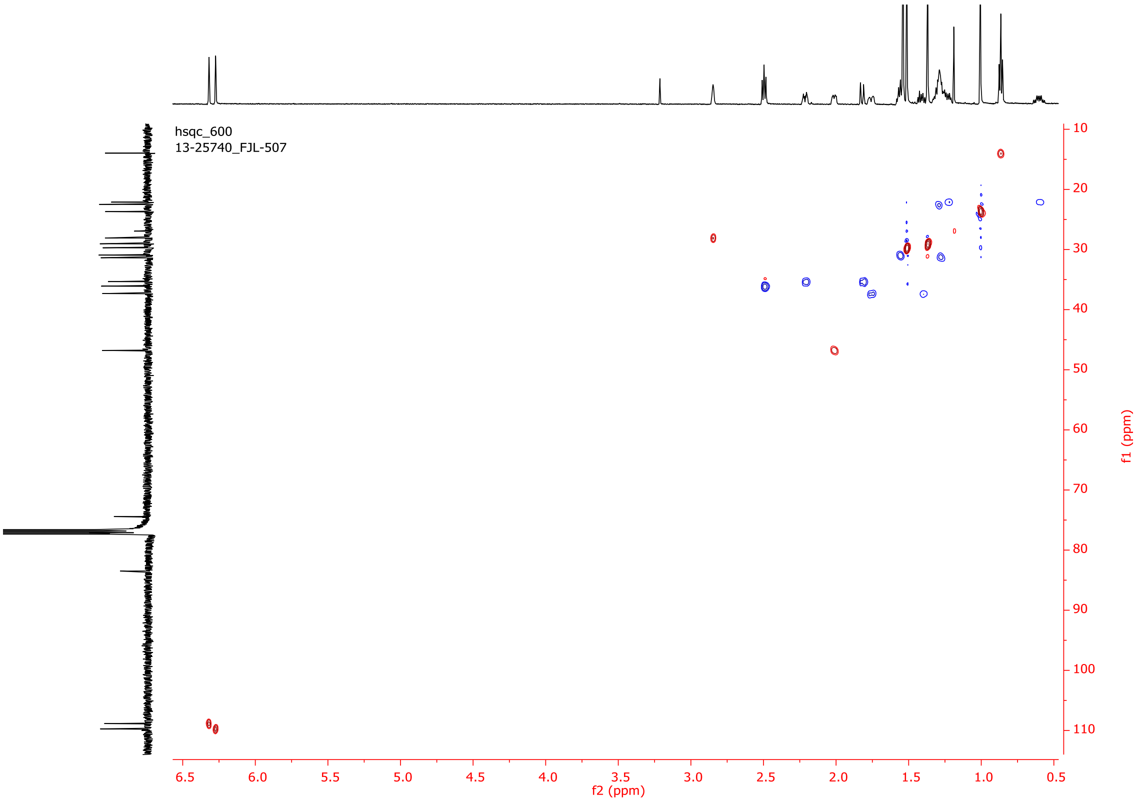

^{1}H NMR of **15** (300 MHz, $CDCl_3$)

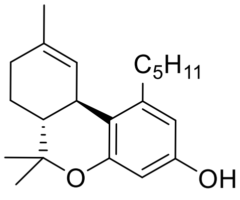

15

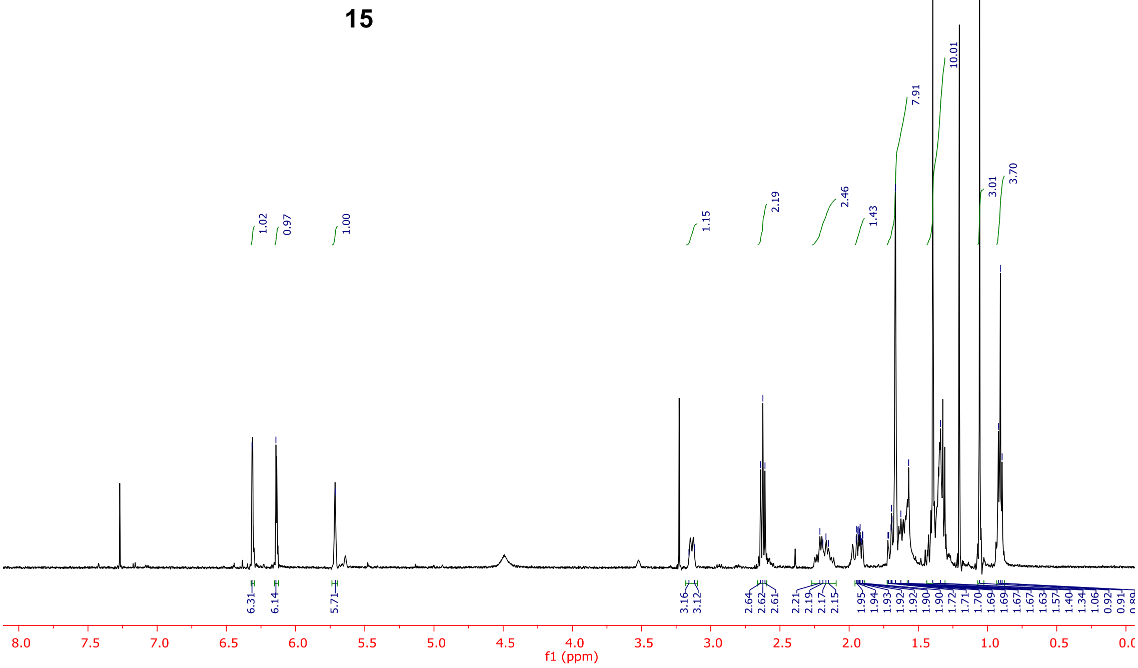

$^{13}C\{^{1}H\}$ NMR of **15** (75 MHz, $CDCl_3$)

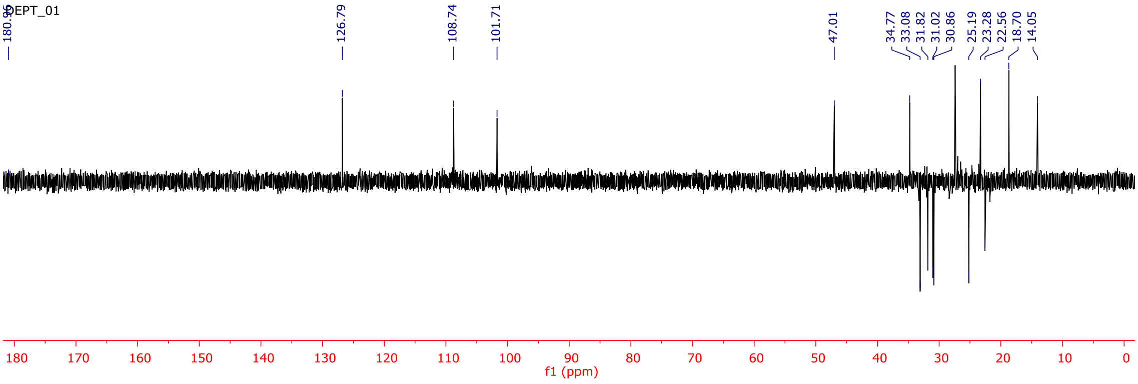

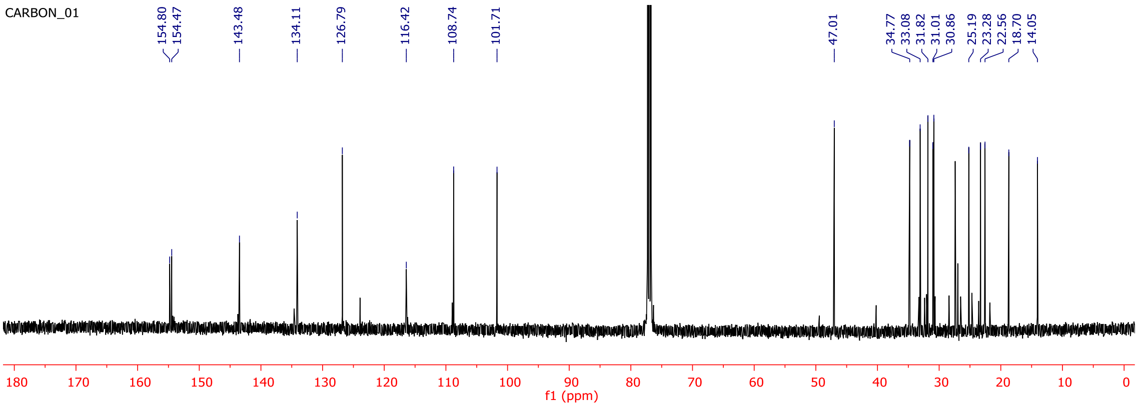

HSQC of 15 (300/75 MHz, $CDCl_3$)

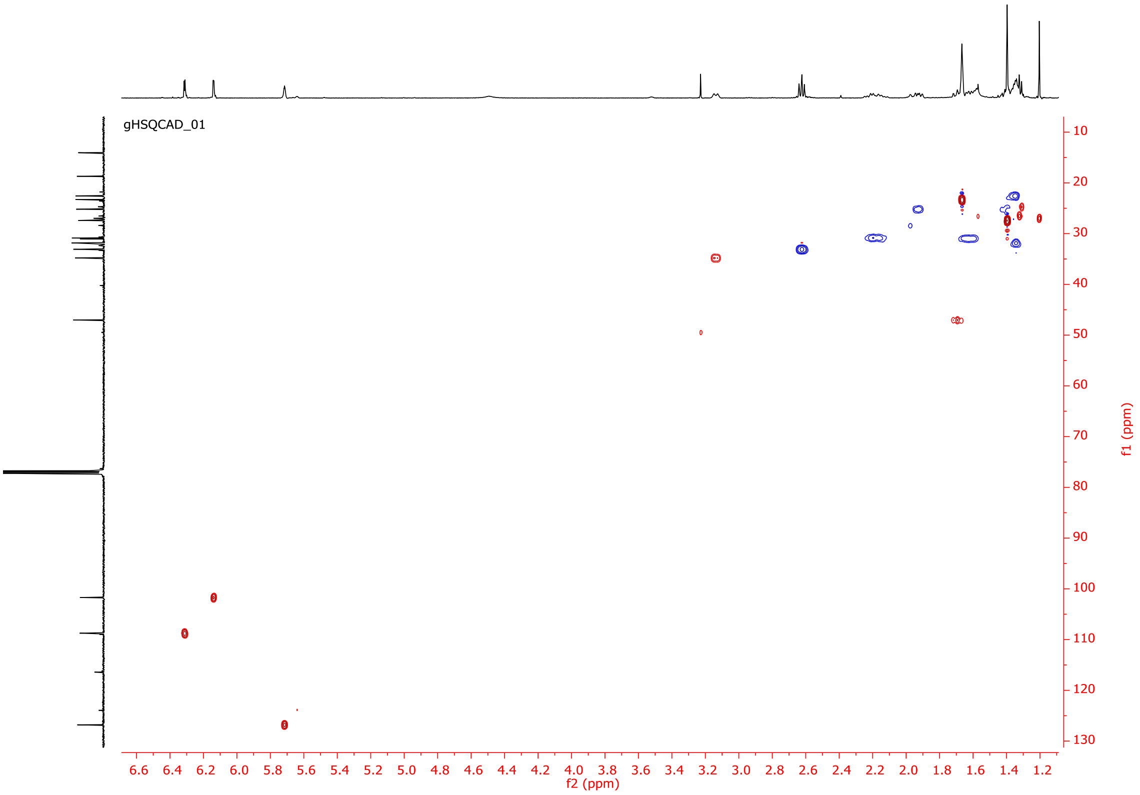

^{1}H NMR of **16** (300 MHz, $CDCl_3$)

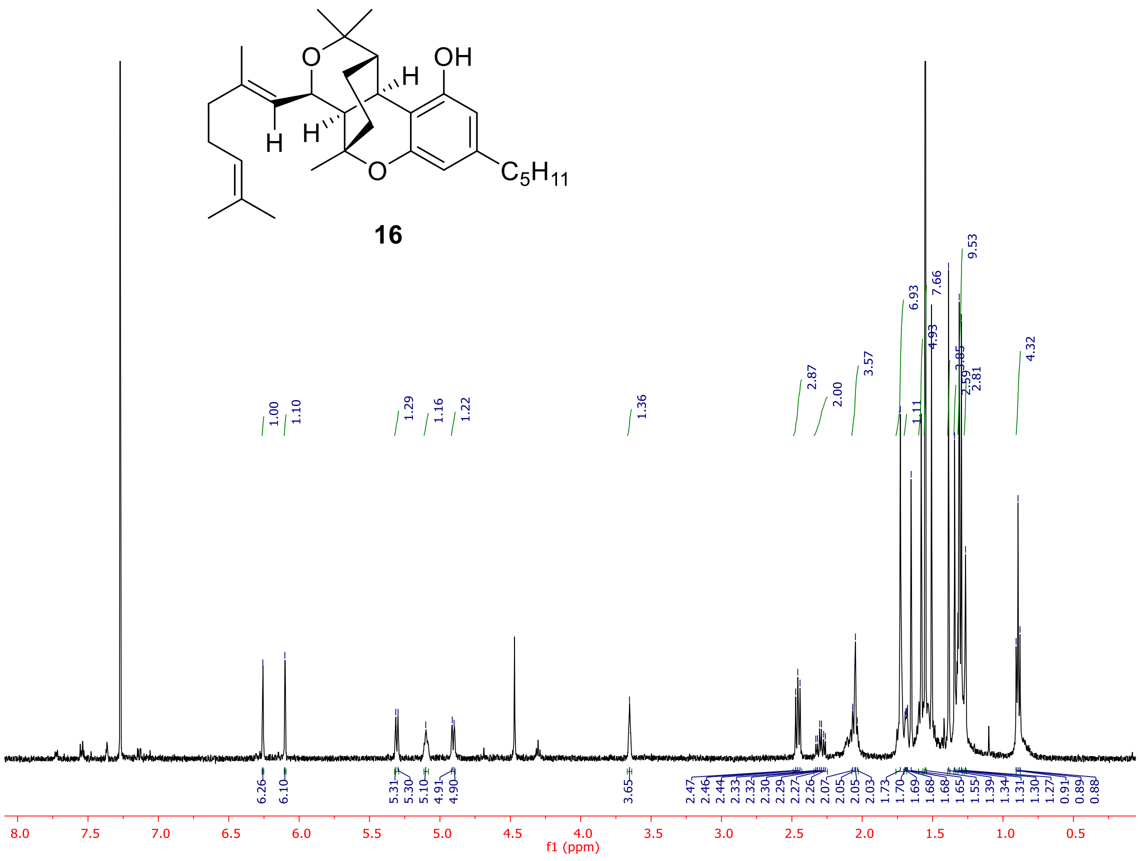

$^{13}C\{^1H\}$ NMR of **16** (75 MHz, $CDCl_3$)

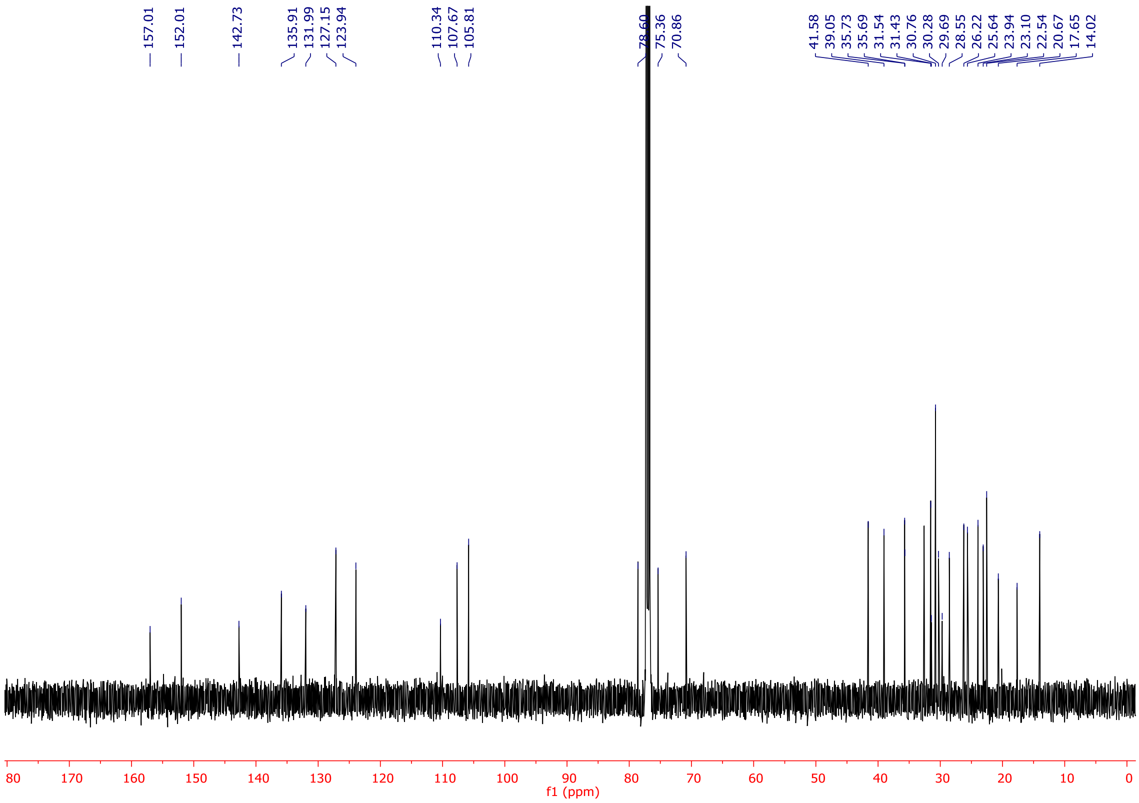

COSY of 16 (300 MHz, $CDCl_3$)

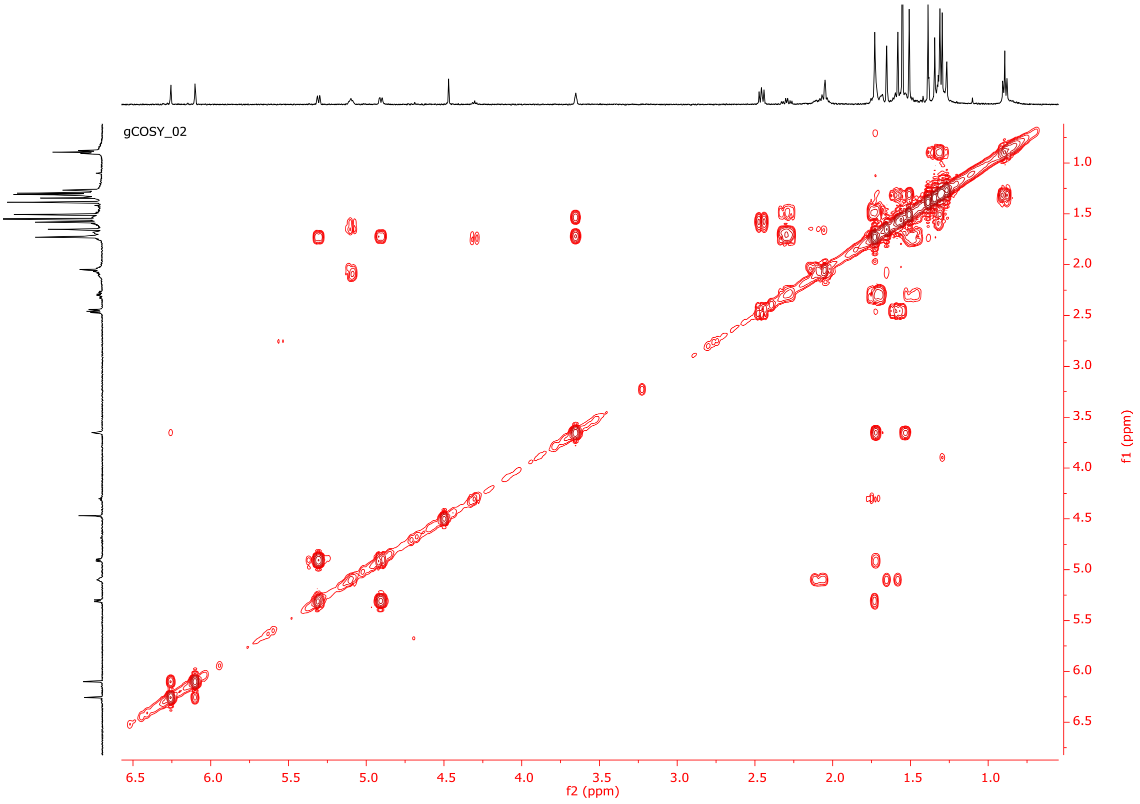

HSQC of 16 (300/75 MHz, $CDCl_3$)

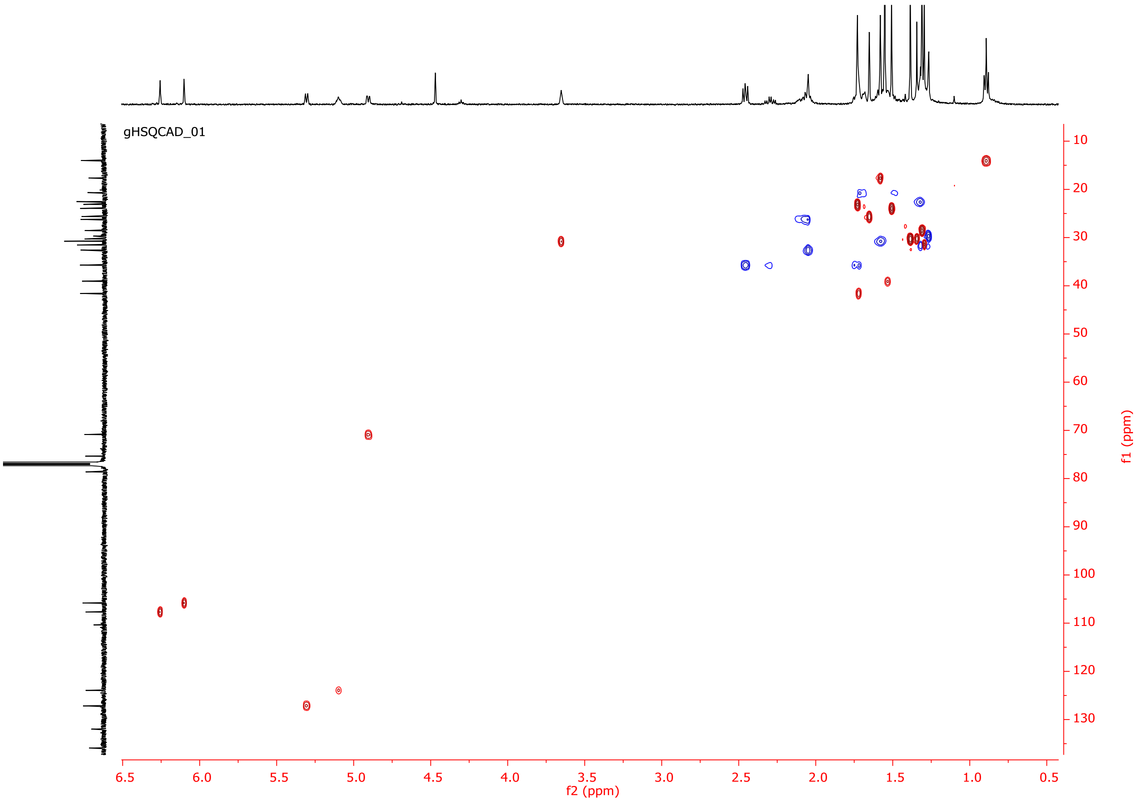

HMBC of 16 (300/75 MHz, $CDCl_3$)

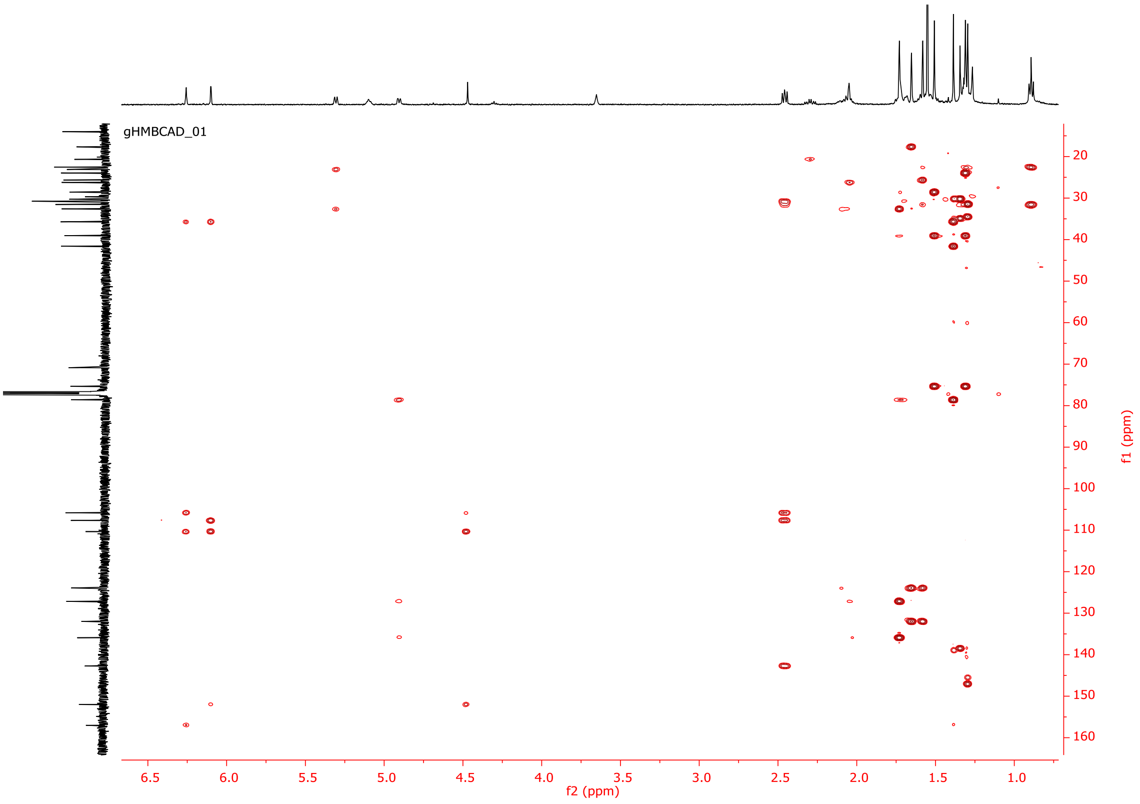

1D TOCSY of 16 (300 MHz, $CDCl_3$)

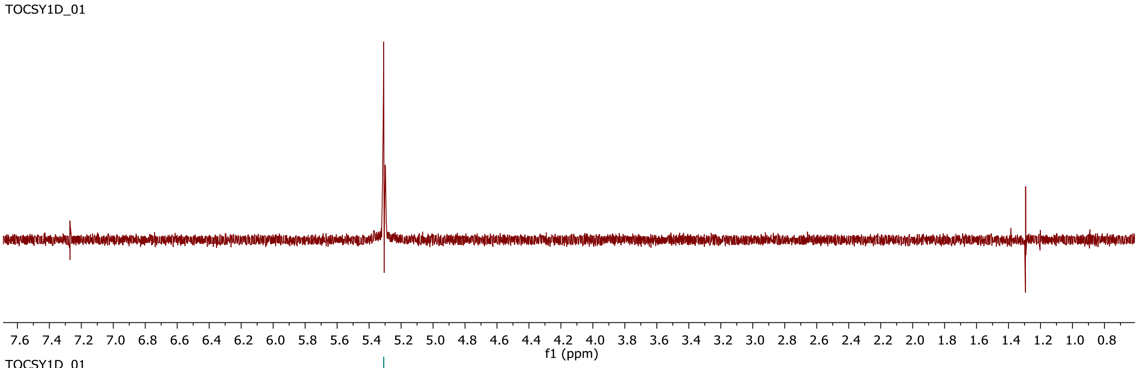

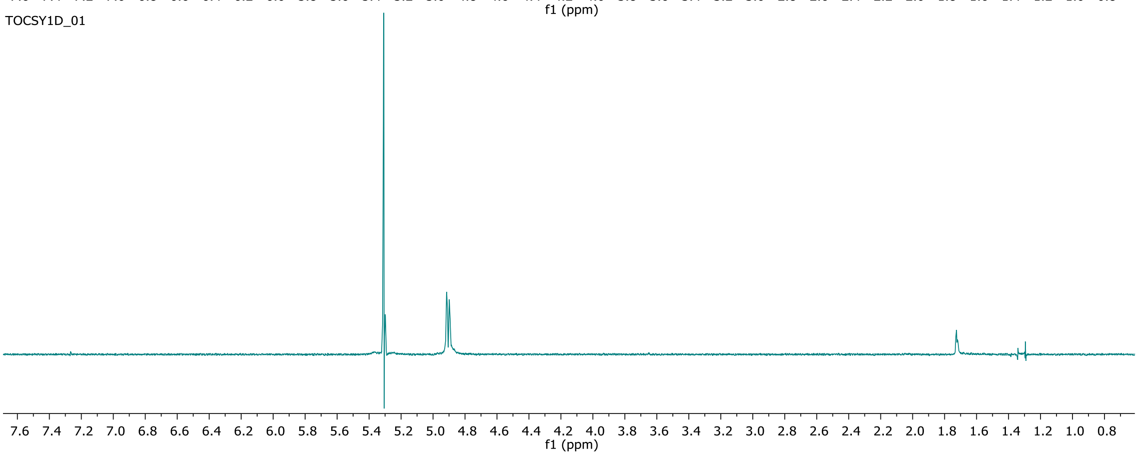

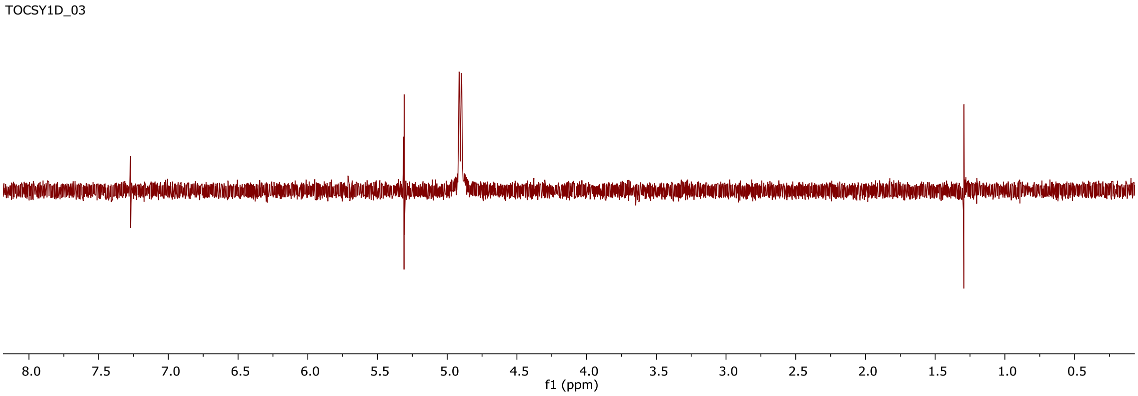

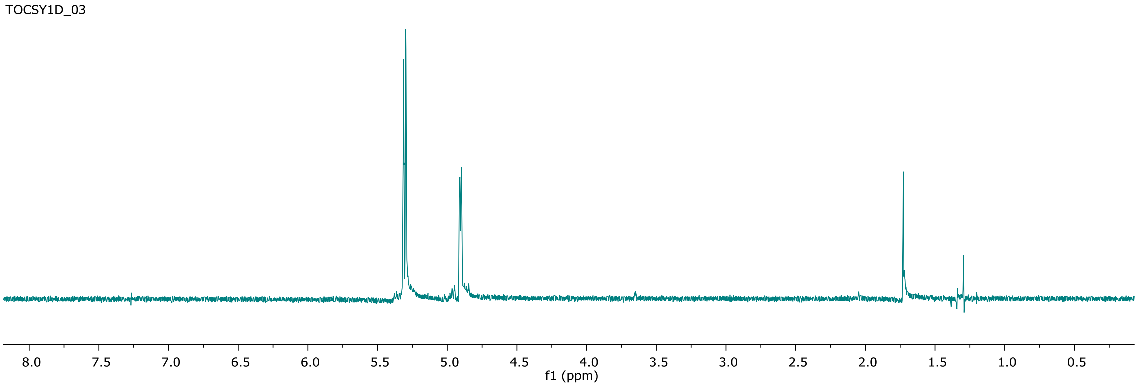

TOCSY1D_04

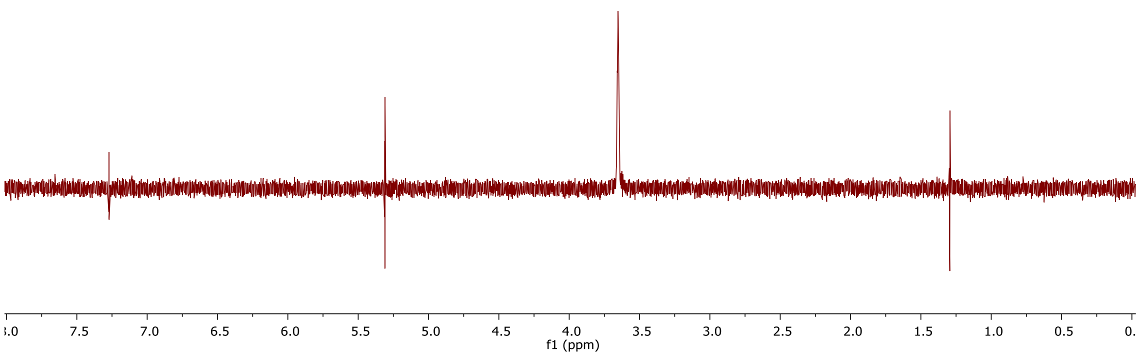

TOCSY1D_04

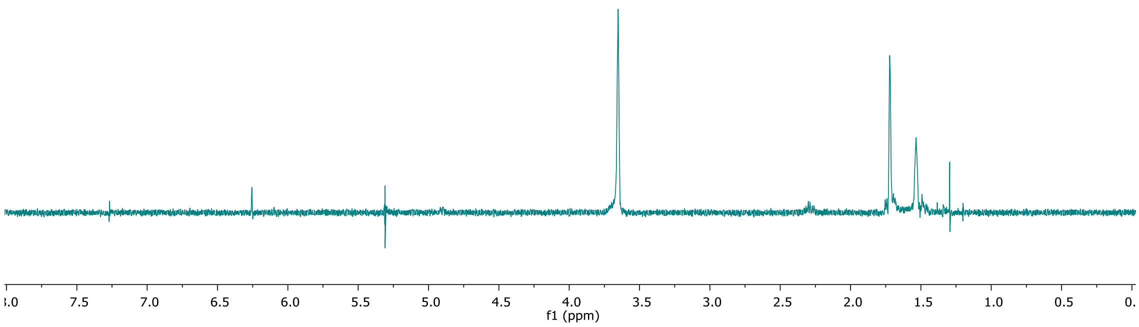

TOCSY1D_05

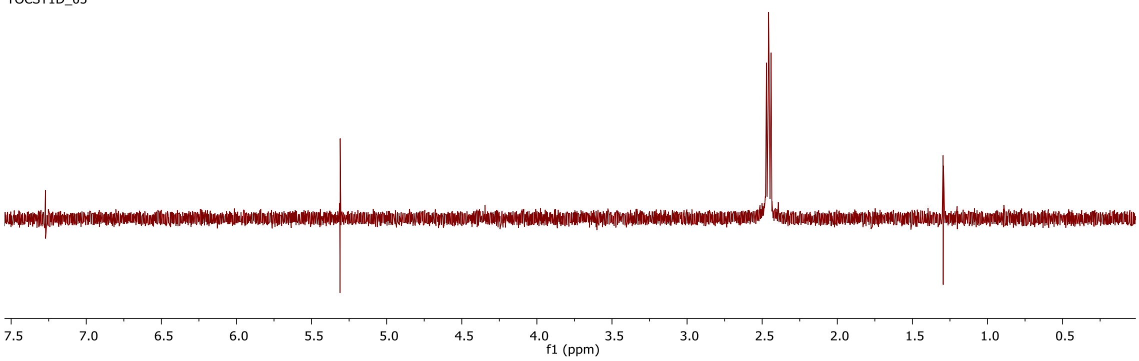

TOCSY1D_05

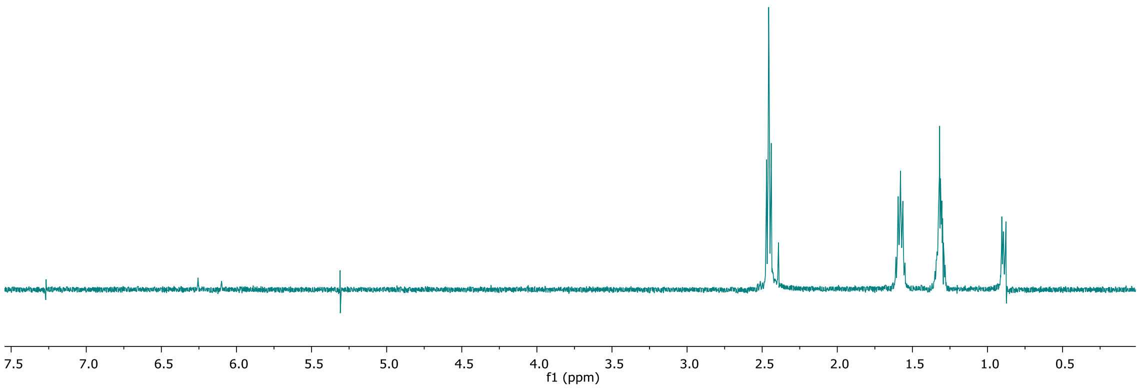

TOCSY1D_06

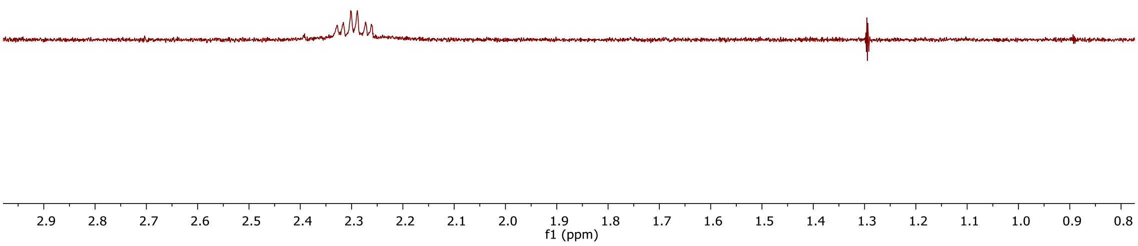

TOCSY1D_06

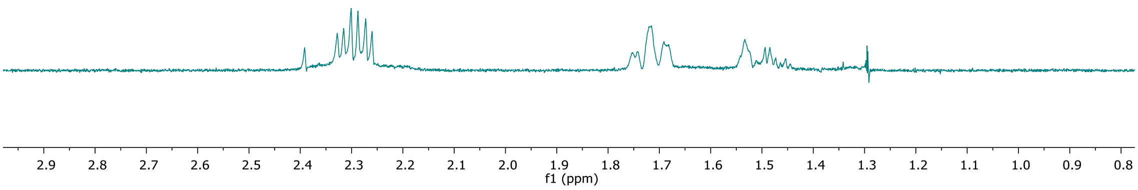

1D NOESY of 16 (300 MHz, $CDCl_3$)

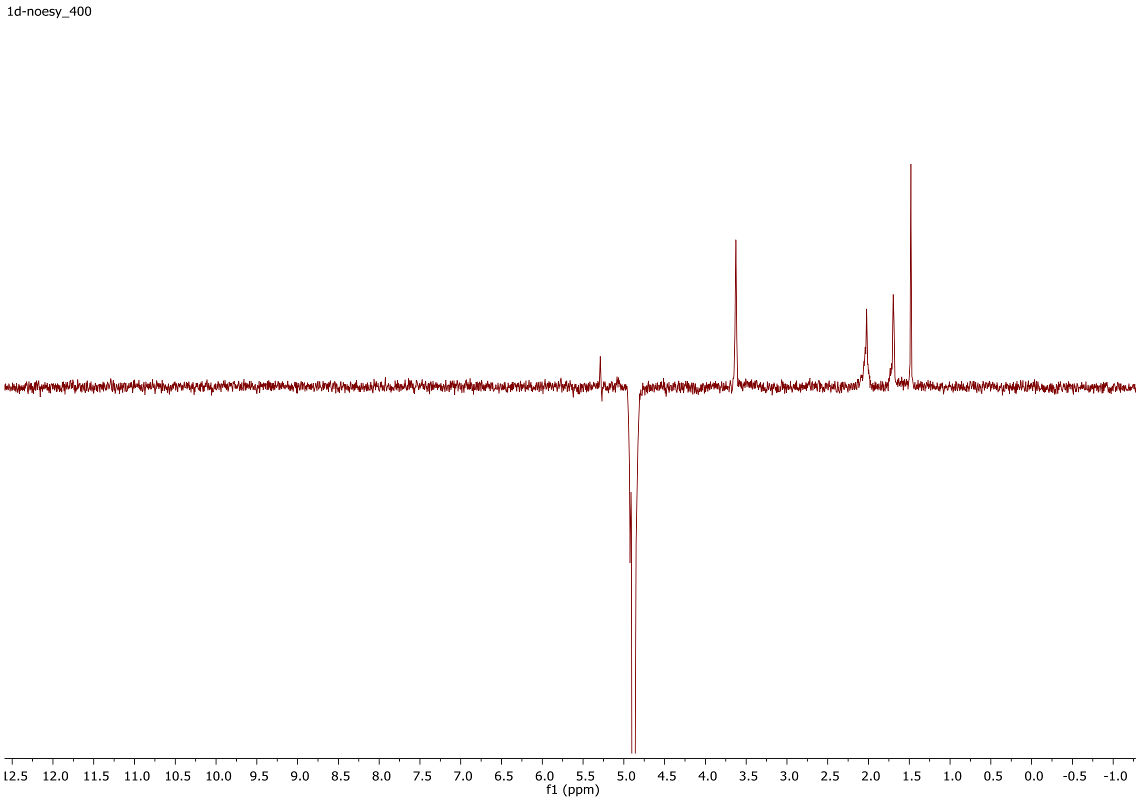

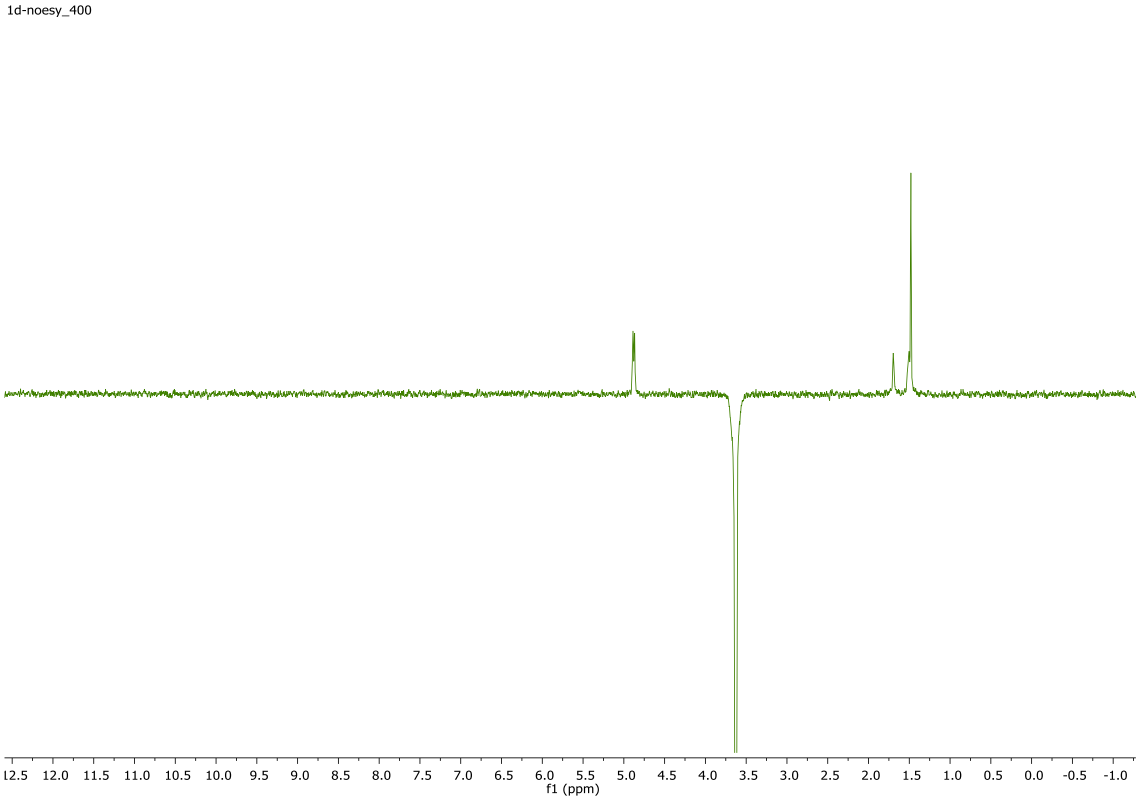

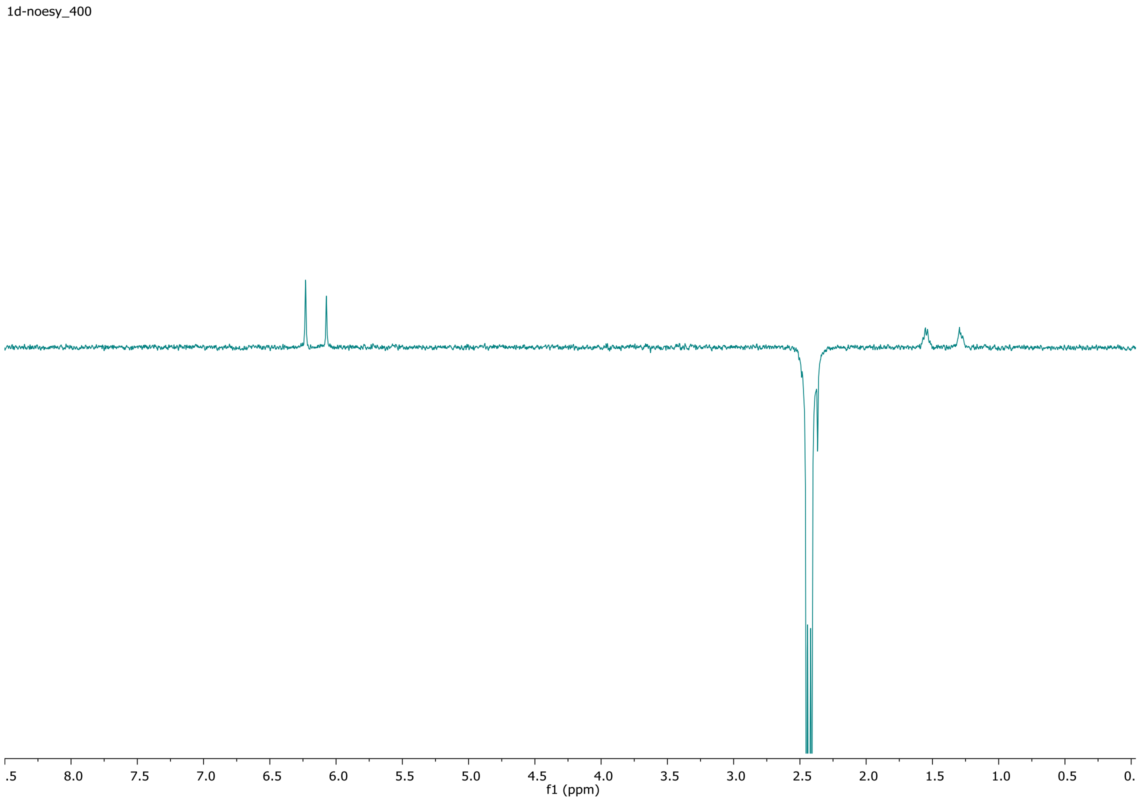
1d-noesy_400
.5
8.0
7.5
7.0
6.5
6.0
5.5
5.0
4.5
4.0
3.5
3.0
2.5
2.0
1.5
1.0
0.5
0.
f1 (ppm)

^{1}H NMR of **17** (500 MHz, $CDCl_3$)

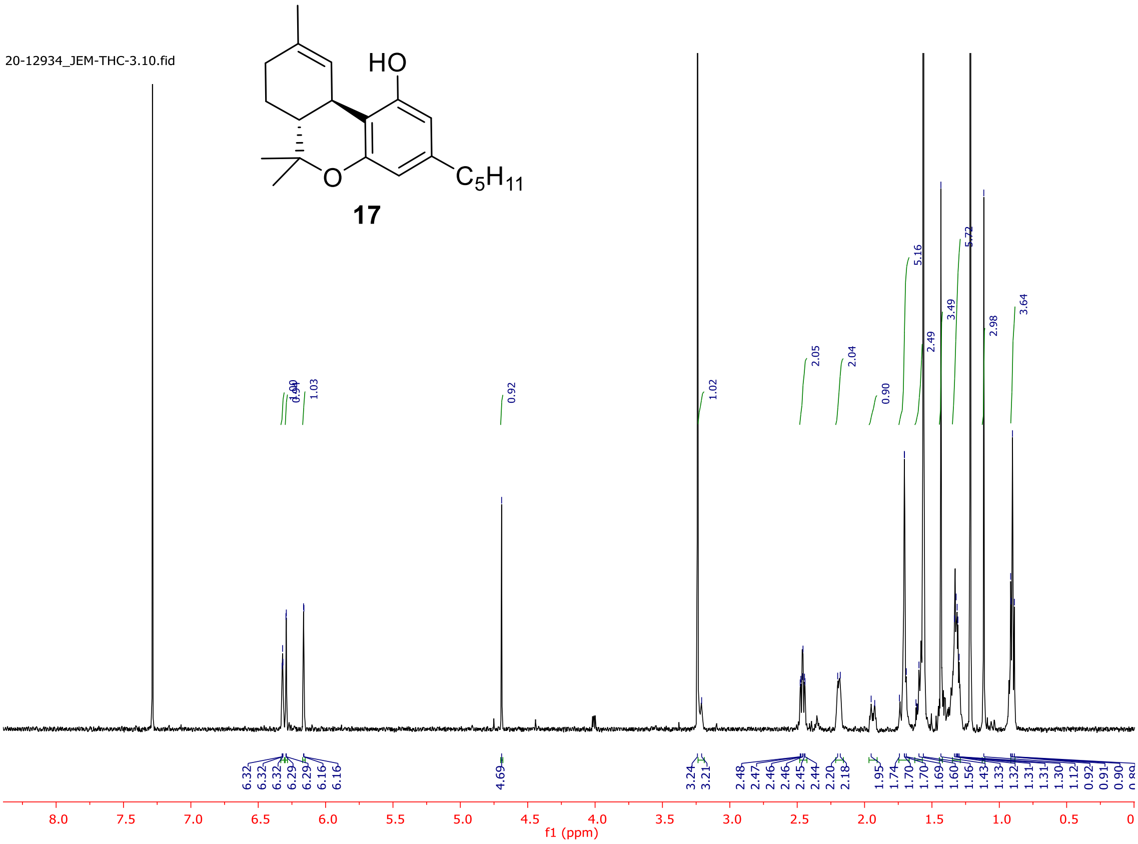

HSQC of 17 (500/126 MHz, $CDCl_3$)

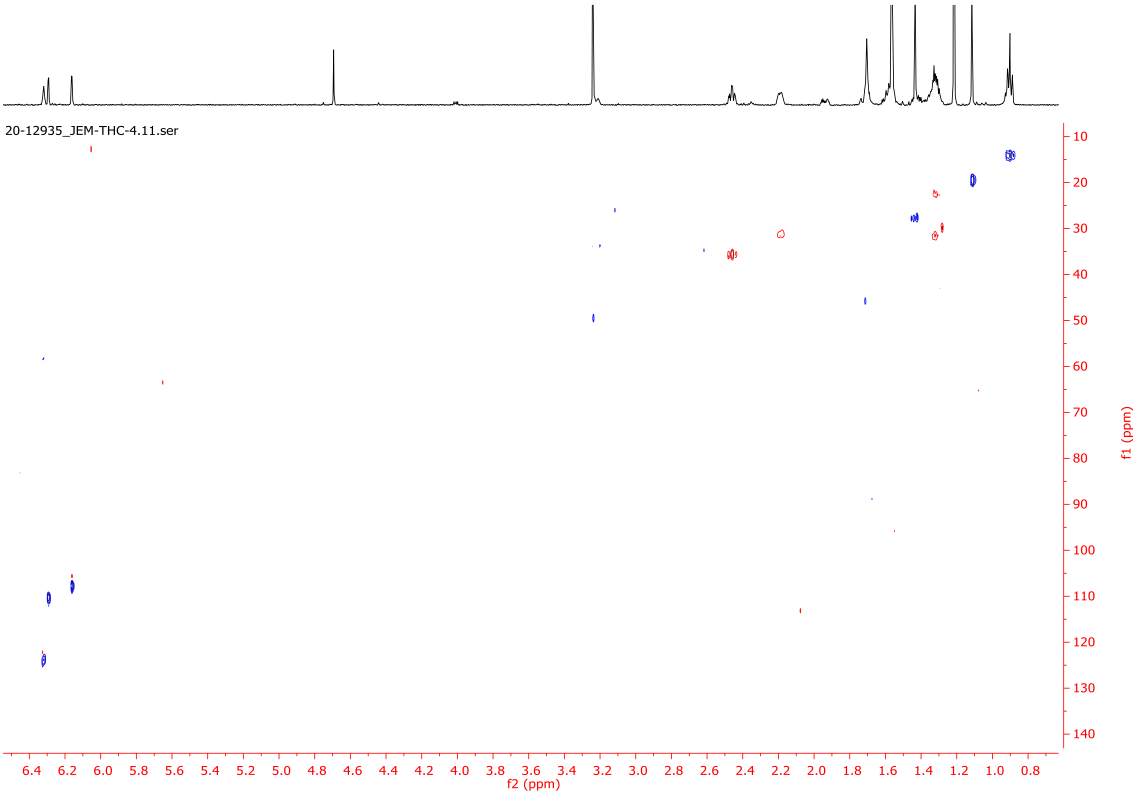

^{1}H NMR of **18** (400 MHz, $CDCl_3$)

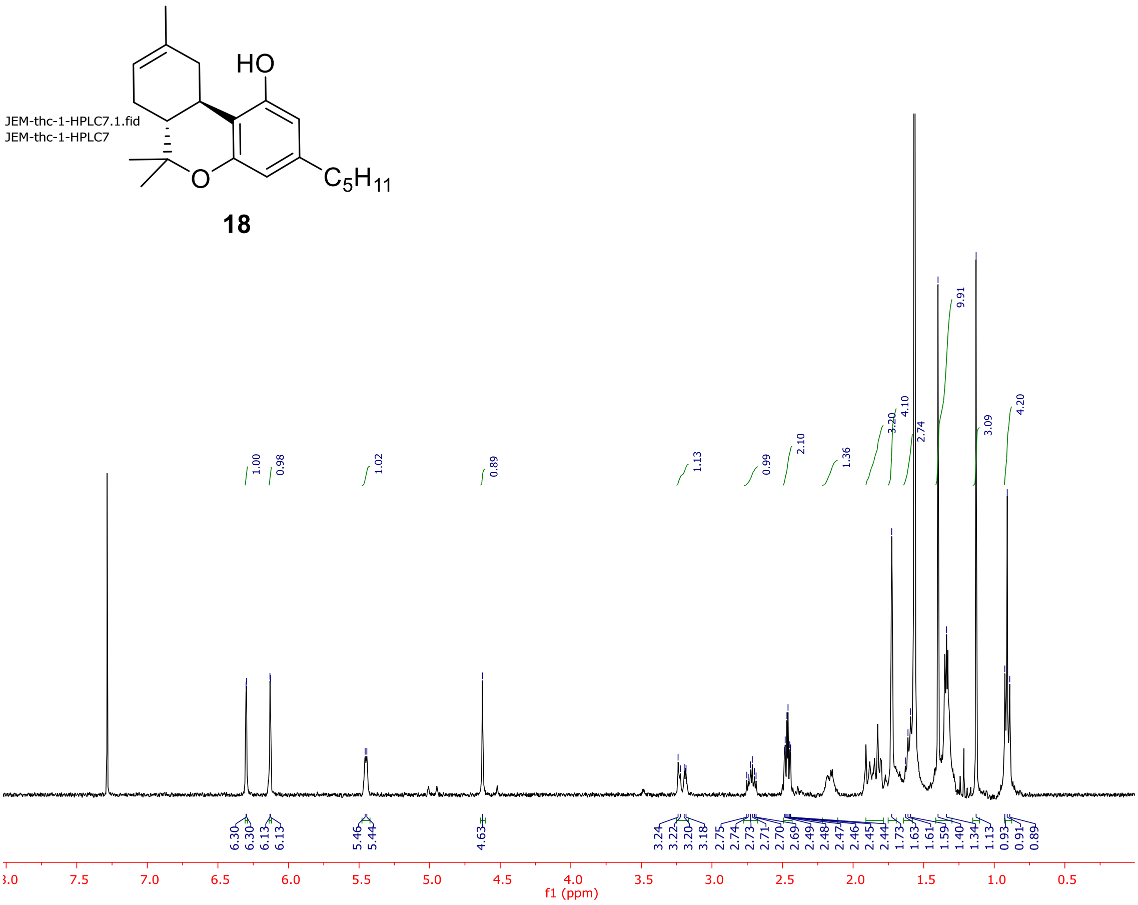

2. Diffusion experiments

PGSE NMR diffusion measurements were carried out using the stimulated echo sequence and monopolar pairs pulses (stegplsld) as described previously.[1] A smoothed rectangular shape was used for the gradient pulses and their strength varied automatically in the course of the experiments. The D values were determined from the slope of the regression line ln(I/I0) versus G2 (Figures S1-S4).

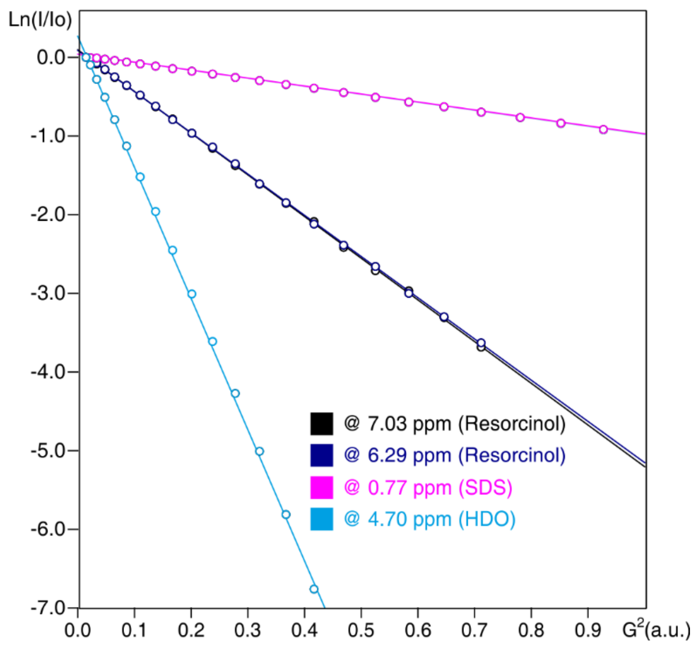

Figure S1. Stejskal-Tanner plots from ^{1}H PGSE NMR diffusion experiments in D2O at 294 K using the stimulated echo with bipolar pair pulses (stebpgp1s1d) sequence for compound citral (2 mM) in presence of SDS micelles (40 mM). The solid lines represent linear least-squares fits to the experimental data.

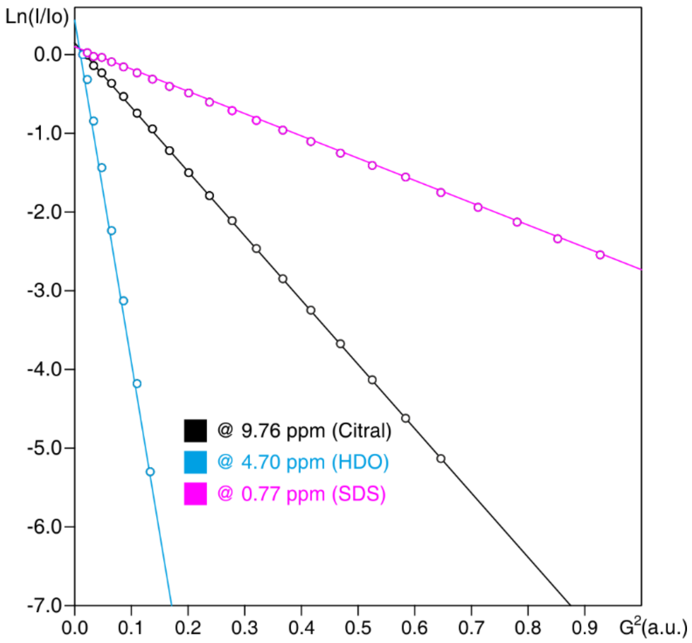

Figure S2. Stejskal-Tanner plots from ^{1}H PGSE NMR diffusion experiments in D_2O at 294 K using the stimulated echo with bipolar pair pulses (stebpgp1s1d) sequence for compound resorcinol (2 mM) in presence of SDS micelles (40 mM). The solid lines represent linear least-squares fits to the experimental data.

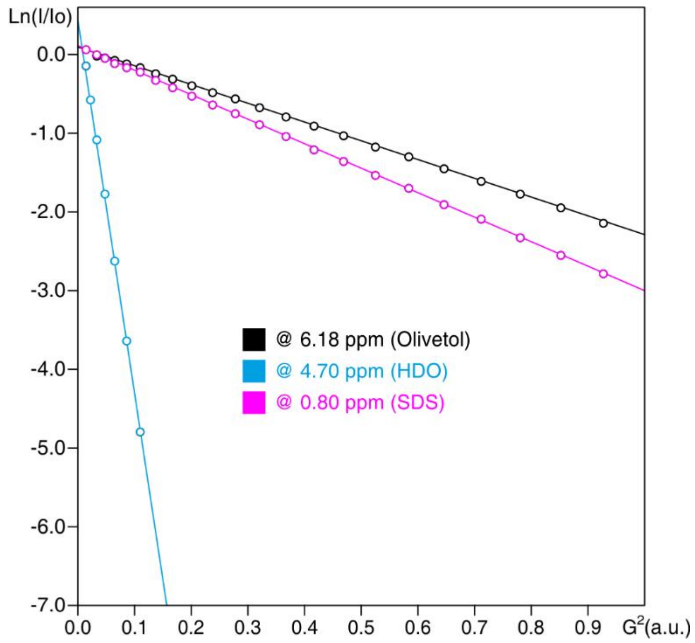

Figure S3. Stejskal-Tanner plots from ^{1}H PGSE NMR diffusion experiments in D_2O at 294 K using the stimulated echo with bipolar pair pulses (stebpgp1s1d) sequence for compound olivetol (2 mM) in presence of SDS micelles (40 mM). The solid lines represent linear least-squares fits to the experimental data.

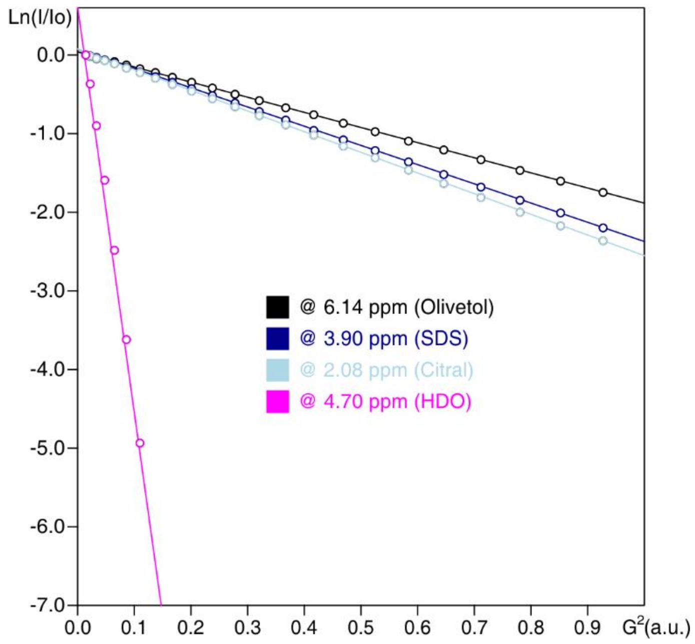

Figure S4. Stejskal-Tanner plots from ^{1}H PGSE NMR diffusion experiments in D_2O at 294 K using the stimulated echo with bipolar pair pulses (stebpgp1s1d) sequence for compound olivetol (2 mM) and citral (2 mM) in presence of SDS micelles (40 mM). The solid lines represent linear least-squares fits to the experimental data.

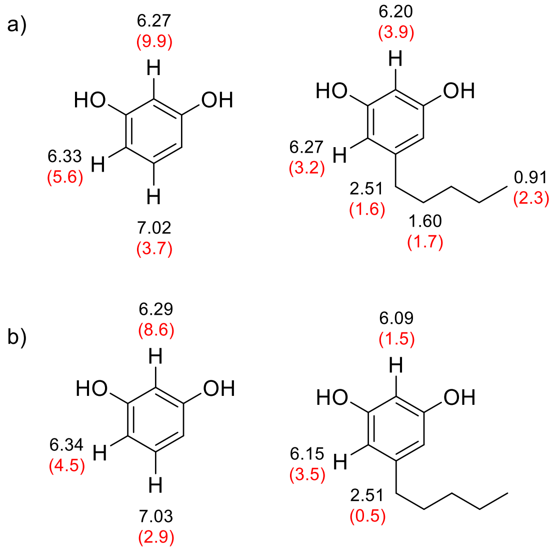

Figure S5. ^{1}H NMR Chemical Shifts and Relaxation T_1 Times (in Brackets) for the two Diphenols Under Study (Resorcinol Left and Olivetol Right) in a) Absence and b) Presence of Micelles of SDS at 40 Mm. The Concentration of Both Molecules Were in all the Cases 2 Mm.

3. Computational Details

Computational Chemistry methodology

Geometry optimizations and energy calculations were performed with GAUSSIAN 09[2] using DFT[3] at the MN15/6-31+g(d,p)[4] level of theory *in vacuo*. Intermediates and products and the saddle points of the reactions were located by means of GRRM[5] (Global Reaction Route Mapping) program, linked to GAUSSIAN 09, with the routines SCW[6] and 2PSHS.[7] Transition state structures were optimized as saddle points at the same level of calculation with the routine SADDLE implemented also in GRRM. A vibrational analysis was performed at the same level of theory *in vacuo* in order to determine the zero-point vibrational energy and to characterize each stationary point as a minimum or transition state structure. Transition states were identified by the presence of a single imaginary frequency that corresponds to the expected motion along the reaction coordinate. The reported energies are expressed in Kcal/mol. The same energies expressed in Kcal/mol as relative energies appear in the Plot of IRC, but do not include zero-point energy corrections. To verify that the TSs correspond to the expected reactant and product wells, intrinsic reaction coordinate (IRC)[8] calculations were performed at the same level MN15/6-31+g(d,p). Structural drawings were produced with Spartan08.[9]

Scheme S1. Proposed formation mechanism of compound 3 (hetero Diels-Alder).

Plot of IRC transformation of **IIIb** in to **3** (MN15/6-31+g(d,p)

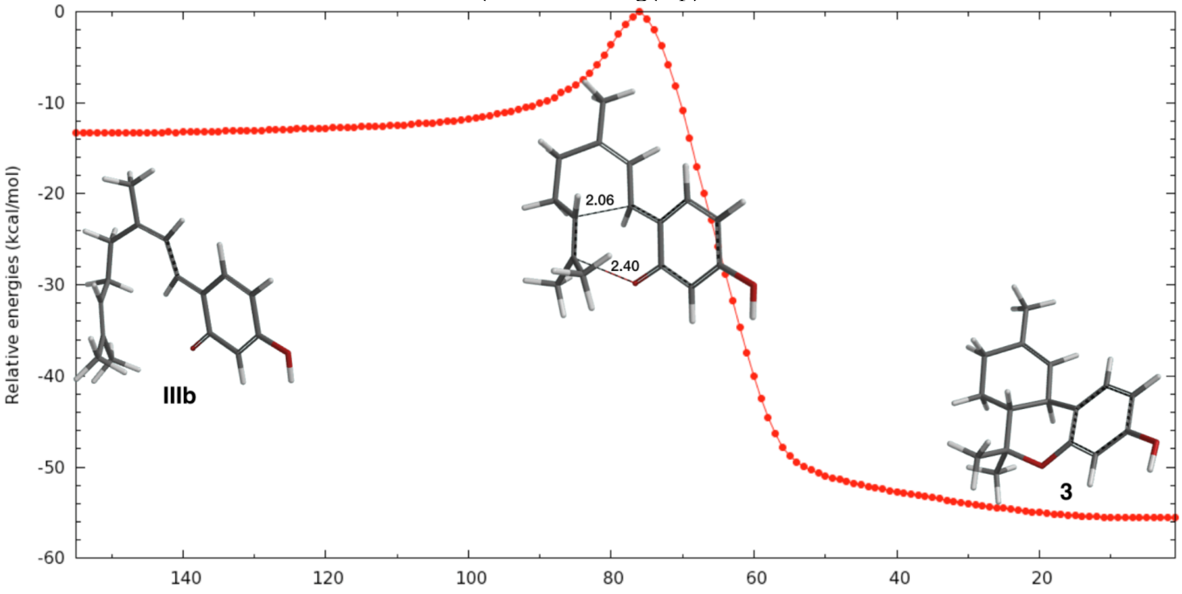

Cartesian Coordinates and Energies of **IIIb**.

#FREQ/MN15/6-31+g(d,p) //# MIN/MN15/6-31+g(d,p)

Charge = 0 Multiplicity = 1

----

1 C1	-1.116889375984	0.808510292075	-4.156459573465 C
2 C2	-0.664420045586	0.880428150230	-1.365295122630 C
3 C3	-1.465116491425	-0.293642543727	-3.435423086453 C
4 C4	-0.530232673178	1.983849311915	-3.544997976675 C
5 C5	-0.315531182652	2.005483304432	-2.208734204394 C
6 C6	-1.263715233376	-0.339001526873	-1.998016399432 C
7 H7	-1.903423220120	-1.172709446716	-3.903513892682 H
8 H8	-0.277789550935	2.817947640692	-4.192145989228 H
9 O9	-1.282044680819	0.909506811298	-5.497354151575 O
10 H10	-1.678070497696	0.099803230743	-5.852681700951 H
11 O11	-1.563346379619	-1.337397625062	-1.327110163802 O
12 C12	-0.456674609255	0.827319899582	-0.012906117979 C
13 H13	-0.782441165695	-0.100051122014	0.446094829910 H
14 C14	0.131782540139	1.861225300030	0.804257477142 C
15 H15	0.266968281149	2.833768106535	0.330420201928 H
16 C16	1.177037473138	-1.288663227304	1.425224402692 C
17 H17	2.020845103749	-0.637493509150	1.180863262954 H
18 C18	0.975670656179	-2.379965821095	0.670097916049 C
19 C19	1.846785474999	-2.686554258537	-0.517487738565 C
20 H20	2.655435295544	-1.956671338852	-0.627213085101 H
21 H21	2.286957287199	-3.689693582729	-0.442617860803 H
22 H22	1.237916678105	-2.669373452354	-1.432231838027 H
23 C23	-0.157630119460	-3.344380138692	0.897933935485 C
24 H24	-0.661843530080	-3.187479038266	1.855962540490 H
25 H25	-0.899628794411	-3.239243493978	0.096564008829 H
26 H26	0.213388661343	-4.377399971028	0.875887816855 H
27 C27	0.285913944500	-0.830668901298	2.557260853328 C
28 H28	-0.773689804174	-0.918207634816	2.280239777749 H
29 H29	0.410089043480	-1.487112204842	3.431109638637 H
30 C30	0.593964290375	0.592197319624	3.030895647714 C
31 H31	-0.068839737141	0.844322507501	3.873297585261 H

32 H32	1.605553556854	0.592176856425	3.469112291012 H
33 C33	0.564302395560	1.775967590625	2.089298091795 C
34 C34	1.140905815198	3.012008753053	2.733481201076 C
35 H35	1.047844184059	3.892662067388	2.092230936378 H
36 H36	0.636006024473	3.220320804231	3.685959516141 H
37 H37	2.203174518732	2.863662178392	2.969023791447 H
38 H38	0.130306537010	2.887311801029	-1.756532472522 H

Thermochemistry at 298.150 K, 1.000 Atm
E(el) = -483967.379650 kcal/mol
ZPVE = 201.714060 kcal/mol
Enthalpie(0K) = -483765.665590 kcal/mol
E(tr) = 0.888732 kcal/mol
E(rot) = 0.888732 kcal/mol
E(vib) = 212.066546 kcal/mol
H-E(el) = 214.436498 kcal/mol
Enthalpie = -483752.943153 kcal/mol
S(el) = 0.000000000000
S(tr) = 0.000067534655
S(rot) = 0.000053622490 (Symmetry number= 1)
S(vib) = 0.000113077623
G-E(el) = 170.613064 kcal/mol
Free Energy = -483796.766586 kcal/mol

Cartesian Coordinates and Energies of **Transition state IIIb - 3**

#FREQ/B3LYP/6-31+g(d,p) //# SADDLE/MN15/6-31+g(d,p)
Charge = 0 Multiplicity = 1
----

1 C1	-0.770285914601	0.519973156194	-3.947647715824 C
2 C2	-0.620981577127	0.678281263336	-1.147646996156 C
3 C3	-1.274474007806	-0.524095891774	-3.209048178256 C
4 C4	-0.145391371071	1.644940769730	-3.331727614075 C
5 C5	-0.060130817707	1.700777424721	-1.963463991503 C
6 C6	-1.215665850289	-0.491154694603	-1.774004660281 C
7 H7	-1.722828084871	-1.396755935959	-3.681568517021 H
8 H8	0.246070048176	2.431587471159	-3.969155917422 H
9 O9	-0.815212670890	0.549131450038	-5.309528713326 O
10 H10	-1.262247665744	-0.241049156427	-5.645326273868 H
11 O11	-1.569991902322	-1.464512336425	-1.044718633297 O
12 C12	-0.559245147067	0.627585150476	0.265943417927 C
13 H13	-1.339509599009	-0.000809655603	0.699070790459 H
14 C14	-0.103152372645	1.809294327522	1.037378538756 C
15 H15	-0.330386606748	2.772023485617	0.575501272986 H
16 C16	0.778265594052	-0.820021806663	0.877949573224 C
17 H17	1.634166699681	-0.250591246123	0.505798599678 H
18 C18	0.449725577674	-1.968662376489	0.161461520471 C
19 C19	1.126911634648	-2.206970453688	-1.150335535045 C
20 H20	1.243284768157	-1.257210412782	-1.694157276457 H
21 H21	2.137158812573	-2.609228024304	-0.989076868973 H
22 H22	0.560926887273	-2.902757347520	-1.776067071445 H
23 C23	-0.366906138979	-3.055422829541	0.788273055699 C
24 H24	0.221816023970	-3.579351549155	1.557412952589 H
25 H25	-1.255802100659	-2.636442502862	1.274016507804 H
26 H26	-0.703050907070	-3.775821197725	0.039544316543 H
27 C27	0.512750792454	-0.722487433002	2.366917285956 C

28 H28	-0.563431440998	-0.840324518788	2.559159281495	H
29 H29	1.004870661082	-1.553087559808	2.893256022525	H
30 C30	0.997060170735	0.599701694911	2.954115007419	C
31 H31	0.676891612532	0.681089869072	4.002798840168	H
32 H32	2.098851888093	0.614249788131	2.986281610378	H
33 C33	0.547369738491	1.831966296019	2.213500507142	C
34 C34	0.930032416199	3.133672256990	2.864462837444	C
35 H35	0.623432447332	3.994296251436	2.262732372629	H
36 H36	0.466049772042	3.222179741094	3.855494000930	H
37 H37	2.016030786612	3.187070737603	3.018353876652	H
38 H38	0.427193242530	2.552994586324	-1.494257390359	H

Imag. Freq. -465.76 cm^{-1}
Thermochemistry at 298.150 K, 1.000 Atm
E(el) = -483954.049033 kcal/mol
ZPVE = 201.713867 kcal/mol
Enthalpie(0K) = -483752.335166 kcal/mol
E(tr) = 0.888732 kcal/mol
E(rot) = 0.888732 kcal/mol
E(vib) = 210.915298 kcal/mol
H-E(el) = 213.285250 kcal/mol
Enthalpie = -483740.763782 kcal/mol
S(el) = 0.000000000000
S(tr) = 0.000067534655
S(rot) = 0.000052944490 (Symmetry number= 1)
S(vib) = 0.000087975190
G-E(el) = 174.285128 kcal/mol
Free Energy = -483779.764895 kcal/mol

Cartesian Coordinates and Energies of **3**.
#FREQ/MN15/6-31+g(d,p) //# MIN/MN15/6-31+g(d,p)

Charge = 0 Multiplicity = 1

1 C1	-0.730221088015	0.671821176578	-3.937662132056	C
2 C2	-0.658880618452	0.667368681472	-1.130117730328	C
3 C3	-0.774590507839	-0.542099782841	-3.247873943955	C
4 C4	-0.629082531560	1.877931794932	-3.242009260909	C
5 C5	-0.586710678636	1.862252153213	-1.846695478262	C
6 C6	-0.734875955957	-0.530343399294	-1.854238576869	C
7 H7	-0.841580094802	-1.495217745175	-3.768966781844	H
8 H8	-0.583050394859	2.807203951159	-3.801298250827	H
9 O9	-0.771598341126	0.726857826914	-5.302975599320	O
10 H10	-0.850654299303	-0.162615397701	-5.673977582049	H
11 O11	-0.844434989304	-1.717693437005	-1.191649600658	O
12 C12	-0.605511326674	0.490970596226	0.369651530110	C
13 H13	-1.593870860902	0.116377292221	0.698464548173	H
14 C14	-0.277546658505	1.739327531780	1.142507847270	C
15 H15	-0.839955639779	2.639392022354	0.888830206991	H
16 C16	0.423919048218	-0.620338023924	0.625130729791	C
17 H17	1.343395346375	-0.318482060639	0.094110474205	H
18 C18	-0.057644486670	-1.949429751596	0.007507630278	C
19 C19	1.136220287309	-2.803551735701	-0.410786688234	C
20 H20	1.703690940887	-2.286201380900	-1.193453853328	H
21 H21	1.802653437166	-2.985033675151	0.441477488872	H
22 H22	0.795423413505	-3.768174909343	-0.801767648140	H
23 C23	-0.991110683569	-2.731221280255	0.925090352545	C
24 H24	-0.449533091575	-3.131761117103	1.788695643594	H

25 H25	-1.813988194432	-2.101061930160	1.282031342648	H
26 H26	-1.419005507725	-3.566490185454	0.361440405872	H
27 C27	0.757797717892	-0.707889657513	2.113734528307	C
28 H28	-0.169628137524	-0.859149908949	2.684851812896	H
29 H29	1.412949470587	-1.564299197258	2.322880664817	H
30 C30	1.418190714892	0.592928135942	2.566324398199	C
31 H31	1.510179299728	0.613423367586	3.661339295576	H
32 H32	2.447214604344	0.654129669079	2.177750175378	H
33 C33	0.642502186078	1.809347051825	2.115946865530	C
34 C34	0.958208080267	3.093251930700	2.832839331271	C
35 H35	0.385907785722	3.934068379294	2.428816683330	H
36 H36	0.738876624335	3.007131979498	3.905276952782	H
37 H37	2.026795789397	3.331513002667	2.746731666041	H
38 H38	-0.498143911262	2.804631212338	-1.312028253724	H

Thermochemistry at 298.150 K, 1.000 Atm
E(el) = -484009.620652 kcal/mol
ZPVE = 205.107609 kcal/mol
Enthalpie(0K) = -483804.513043 kcal/mol
E(tr) = 0.888732 kcal/mol
E(rot) = 0.888732 kcal/mol
E(vib) = 213.631931 kcal/mol
H-E(el) = 216.001883 kcal/mol
Enthalpie = -483793.618769 kcal/mol
S(el) = 0.000000000000
S(tr) = 0.000067534655
S(rot) = 0.000052885658 (Symmetry number= 1)
S(vib) = 0.000079957024
G-E(el) = 178.512901 kcal/mol
Free Energy = -483831.107751 kcal/mol

Scheme S2. Proposed formation mechanism of compound 4 (hetero Diels-Alder).

Plot of IRC transformation of **IIIb** in to **4** (MN15/6-31+g(d,p)

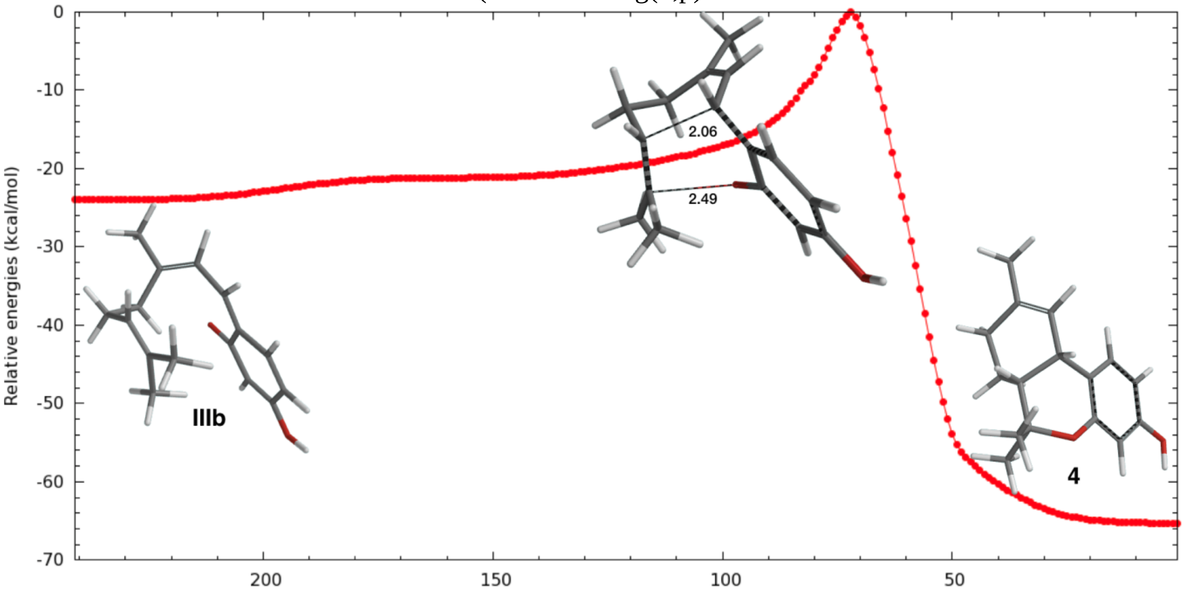

Cartesian Coordinates and Energies of **IIIb**.
#FREQ/MN15/6-31+g(d,p) //# MIN/MN15/6-31+g(d,p)
Charge = 0 Multiplicity = 1

----

1 C1	-0.545526209344	0.847183414319	0.953082503383 C
2 C2	-1.914257853559	1.303076223919	1.137124967965 C
3 C3	-2.465482069970	2.254202106431	0.335893897575 C
4 C4	-1.713240975481	2.886426690829	-0.730559130596 C
5 C5	-0.435377780077	2.504089427206	-0.958053931905 C
6 C6	0.215090690130	1.458118884530	-0.185273387514 C
7 C7	1.513187750550	1.161296134178	-0.512469739477 C
8 C8	2.484194966968	0.207600949091	0.000708640123 C
9 C9	2.365199698626	-1.128384996852	0.167272780146 C
10 C10	1.147242528005	-1.922374900172	-0.211230227205 C
11 C11	1.269253123977	-2.512668138462	-1.639542761945 C
12 C12	1.296477781152	-1.433920378784	-2.693131905883 C
13 C13	0.234984439062	-0.718087940027	-3.101774577040 C
14 C14	-1.163411252754	-0.920631662146	-2.581008553710 C
15 C15	0.380160986478	0.400083676968	-4.099718283846 C
16 C16	3.533485407430	-1.935905749945	0.659690454139 C
17 O17	-0.042785353064	0.002011597785	1.704332130561 O
18 H18	-2.487098410059	0.855787507829	1.943025317980 H
19 H19	-2.184045338919	3.667586579188	-1.325001720954 H
20 H20	1.937337935888	1.820938496788	-1.277838875124 H
21 H21	3.480509674153	0.636272582802	0.135461023065 H
22 H22	1.012422916458	-2.735712712968	0.514567071297 H
23 H23	0.251762234613	-1.301551754243	-0.166905249832 H
24 H24	2.171592167659	-3.132099560920	-1.716424135307 H
25 H25	0.418929177090	-3.188285101522	-1.798365124295 H
26 H26	2.271736950419	-1.174555627390	-3.109024020040 H
27 H27	-1.448502564749	-0.085610646160	-1.921457019372 H
28 H28	-1.279371867474	-1.847029300831	-2.011688207078 H
29 H29	-1.886100728261	-0.930917214999	-3.407061071865 H
30 H30	0.018099144787	1.345525592863	-3.669948902545 H
31 H31	1.421849725902	0.535806538041	-4.408621468701 H
32 H32	-0.226591110193	0.211884597335	-4.995532552852 H
33 H33	4.432477290404	-1.326584075298	0.790345714091 H

34 H34	3.279380952988	-2.392018945742	1.625514274875	H
35 H35	3.765122448925	-2.760160851826	-0.027709934684	H
36 O36	-3.750334007520	2.637712595481	0.544942902274	O
37 H37	-4.016206010006	3.319321350618	-0.087959587571	H
38 H38	0.142975027876	2.979670840487	-1.748821500661	H

Thermochemistry at 298.150 K, 1.000 Atm
E(el) = -483963.376376 kcal/mol
ZPVE = 201.598835 kcal/mol
Enthalpie(0K) = -483761.777541 kcal/mol
E(tr) = 0.888732 kcal/mol
E(rot) = 0.888732 kcal/mol
E(vib) = 211.762769 kcal/mol
H-E(el) = 214.132721 kcal/mol
Enthalpie = -483749.243655 kcal/mol
S(el) = 0.000000000000
S(tr) = 0.000067534655
S(rot) = 0.000052889350 (Symmetry number= 1)
S(vib) = 0.000103346934
G-E(el) = 172.266985 kcal/mol
Free Energy = -483791.109391 kcal/mol

Cartesian Coordinates and Energies of **Transition state IIIb - 4**

#FREQ/B3LYP/6-31+g(d,p) //# SADDLE/MN15/6-31+g(d,p)
Charge = 0 Multiplicity = 1

----

1 C1	-0.794344014536	0.628034861411	0.085708066364	C
2 C2	-1.998840308974	1.300235072234	0.494803637388	C
3 C3	-2.158685277783	2.645925504079	0.264629739698	C
4 C4	-1.167450230750	3.412511106931	-0.417496930118	C
5 C5	-0.003930620542	2.799084004956	-0.819902427969	C
6 C6	0.256165468765	1.439347068039	-0.510681220689	C
7 C7	1.480870112893	0.816029987590	-0.872205456274	C
8 C8	2.280942924785	0.087078676489	0.155163945206	C
9 C9	2.517371609613	-1.210424885847	0.371899306009	C
10 C10	1.998339579730	-2.287324669145	-0.534376535528	C
11 C11	2.018420291017	-1.854522774360	-2.005899675911	C
12 C12	1.096086819211	-0.696105365837	-2.321753353890	C
13 C13	-0.281829778697	-0.881277920041	-2.394374320040	C
14 C14	-0.972319138998	-2.199422657251	-2.210577938115	C
15 C15	-1.137290519944	0.226177431939	-2.907707862923	C
16 C16	3.326037709776	-1.665521010682	1.555458981419	C
17 O17	-0.667087375402	-0.629665566203	0.087558064333	O
18 H18	-2.786738689290	0.723093593612	0.969264596975	H
19 H19	-1.337292658440	4.471633310388	-0.603847072113	H
20 H20	2.113498110202	1.470086512424	-1.484286878313	H
21 H21	2.738218090747	0.805146012855	0.844174483802	H
22 H22	2.613283991876	-3.189384884083	-0.415307558277	H
23 H23	0.980927141501	-2.539080657881	-0.209060458173	H
24 H24	3.047213032119	-1.578159295336	-2.267270325514	H
25 H25	1.751169878359	-2.709507067284	-2.644679123329	H
26 H26	1.478946010885	0.057850289266	-3.011182676679	H
27 H27	-1.984126424091	-2.050989178846	-1.822371598683	H
28 H28	-0.443965060047	-2.873887703698	-1.537911485702	H
29 H29	-1.051770261619	-2.687206340423	-3.195466312170	H

30 H30	-1.752746618422	0.628555021720	-2.084665182741 H
31 H31	-0.555335833622	1.052860334953	-3.325078077172 H
32 H32	-1.835929217762	-0.150418917747	-3.665208289962 H
33 H33	3.651186046396	-0.824340960502	2.175571686133 H
34 H34	2.730652503799	-2.343027960878	2.181374943196 H
35 H35	4.212315696712	-2.227130288110	1.231875392985 H
36 O36	-3.315288897986	3.242657055070	0.676909384962 O
37 H37	-3.309674281464	4.182469842193	0.448993537366 H
38 H38	0.762550198339	3.376078589000	-1.336838357865 H

Imag. Freq. -449.53 cm^{-1}
Thermochemistry at 298.150 K, 1.000 Atm
E(el) = -483939.390252 kcal/mol
ZPVE = 201.175854 kcal/mol
Enthalpie(0K) = -483738.214398 kcal/mol
E(tr) = 0.888732 kcal/mol
E(rot) = 0.888732 kcal/mol
E(vib) = 210.504900 kcal/mol
H-E(el) = 212.874853 kcal/mol
Enthalpie = -483726.515399 kcal/mol
S(el) = 0.000000000000
S(tr) = 0.000067534655
S(rot) = 0.000052556264 (Symmetry number= 1)
S(vib) = 0.000089130951
G-E(el) = 173.731130 kcal/mol
Free Energy = -483765.659122 kcal/mol

Cartesian Coordinates and Energies of **4**.
#FREQ/MN15/6-31+g(d,p) //# MIN/MN15/6-31+g(d,p)
Charge = 0 Multiplicity = 1
----

1 C1	-0.917263966039	0.674996678205	-0.403755696858 C
2 C2	-2.000787209479	1.283931774877	0.217879969174 C
3 C3	-1.945636460572	2.657333694488	0.470942066246 C
4 C4	-0.833712121585	3.407193820068	0.078785561049 C
5 C5	0.242207907068	2.765468214884	-0.542646755748 C
6 C6	0.223507893292	1.394217077193	-0.783443856958 C
7 C7	1.422402887842	0.606410642417	-1.273551025936 C
8 C8	2.196679005634	0.258867831345	-0.013913661514 C
9 C9	2.439819719126	-0.972404127627	0.450354193760 C
10 C10	2.056979473340	-2.175628713462	-0.369094766923 C
11 C11	1.988203311875	-1.803085412354	-1.848338759573 C
12 C12	1.034833020170	-0.635580825942	-2.136041554756 C
13 C13	-0.479585171414	-0.966929126219	-2.021375751039 C
14 C14	-0.824037025458	-2.436982267810	-2.227490468835 C
15 C15	-1.271088752587	-0.143785984935	-3.048531632545 C
16 C16	3.099484587510	-1.225416914248	1.776645935959 C
17 O17	-0.965866656912	-0.661165162700	-0.691117075010 O
18 H18	-2.878074586812	0.705657322883	0.490166891949 H
19 H19	-0.805862619909	4.479768115424	0.263789781064 H
20 H20	2.046788020404	1.278726849313	-1.880422583856 H
21 H21	2.491777566283	1.122952855604	0.585192400502 H
22 H22	2.796908171200	-2.974005561301	-0.216442800274 H
23 H23	1.095598400146	-2.571791620196	-0.009844517508 H
24 H24	2.995637202981	-1.496776513374	-2.163999502635 H
25 H25	1.719874576353	-2.670705541716	-2.462018704570 H
26 H26	1.184369096008	-0.360426313929	-3.190041559387 H

27 H27	-1.912670542188	-2.551344493491	-2.194526285368	H
28 H28	-0.390179420711	-3.069702588511	-1.448775072214	H
29 H29	-0.467439719150	-2.781200879786	-3.204622219524	H
30 H30	-2.343712239429	-0.224907497649	-2.837281688327	H
31 H31	-0.992490945833	0.914926107605	-3.037797577584	H
32 H32	-1.078737268788	-0.536996762638	-4.053275792977	H
33 H33	3.325243167524	-0.291426741197	2.300790863457	H
34 H34	2.448150618166	-1.831002322781	2.420934311435	H
35 H35	4.033161053922	-1.789382651256	1.649662841734	H
36 O36	-3.025649176837	3.222698879010	1.088722413793	O
37 H37	-2.888290857608	4.173226118389	1.201986145339	H
38 H38	1.119374472947	3.344206274149	-0.829816507067	H

Thermochemistry at 298.150 K,   1.000 Atm
E(el)   = -484004.670528 kcal/mol
ZPVE   = 205.438799 kcal/mol
Enthalpie(0K)   = -483799.231729 kcal/mol
E(tr)   = 0.888732 kcal/mol
E(rot)   = 0.888732 kcal/mol
E(vib)   = 213.821696 kcal/mol
H-E(el)   = 216.191648 kcal/mol
Enthalpie   = -483788.478880 kcal/mol
S(el)   = 0.000000000000
S(tr)   = 0.000067534655
S(rot)   = 0.000052456069 (Symmetry number= 1)
S(vib)   = 0.000078372893
G-E(el)   = 179.079417 kcal/mol
Free Energy   = -483825.591111 kcal/mol

Scheme S3. Proposed formation mechanism of compound 3 (acid catalysis).

Plot of IRC transformation of **IIIb** in to **3 (acid catalysis)** (MN15/6-31+g(d,p)

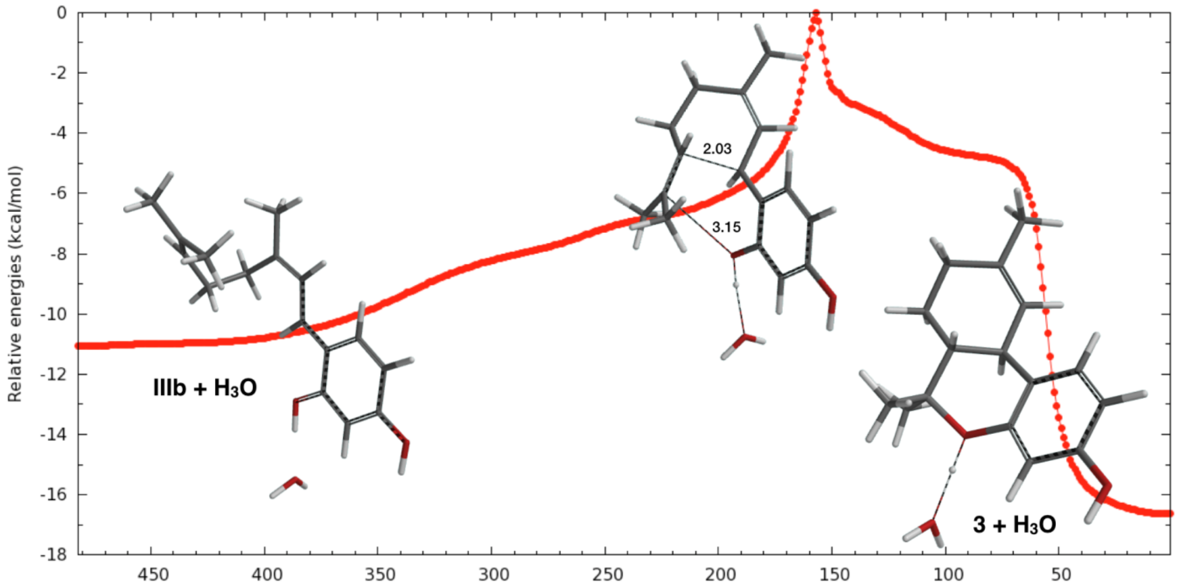

Cartesian Coordinates and Energies of **IIIb +H$_3$O**.
#FREQ/MN15/6-31+g(d,p) //# MIN/MN15/6-31+g(d,p)
Charge = 1 Multiplicity = 1

1 C1	-2.072641914451	0.156338808671	0.835350956322 C
2 C2	-3.373187636541	0.581782470130	0.566851964458 C
3 C3	-3.573183702739	1.676769231531	-0.276188689179 C
4 C4	-2.482683480223	2.372502266908	-0.871593999661 C
5 C5	-1.210777416870	1.954792337543	-0.607533227647 C
6 C6	-0.944350884389	0.837930674538	0.251054271654 C
7 C7	0.344038941864	0.380932143235	0.549454722403 C
8 C8	1.566919542684	0.918887808938	0.097857287469 C
9 C9	2.791030832808	0.360739159197	0.404380985175 C
10 C10	2.955838984121	-0.895462324575	1.208939669697 C
11 C11	2.716063290580	-2.174602012004	0.350230254963 C
12 C12	3.761823214994	-2.395573954358	-0.707968178499 C
13 C13	3.680510688827	-2.078481365563	-2.012329625334 C
14 C14	2.498122231452	-1.402081476220	-2.656457971263 C
15 C15	4.823772982643	-2.361266296363	-2.951163948429 C
16 C16	4.038446341625	0.975056514352	-0.127058193663 C
17 O17	-1.817442591148	-0.877417842244	1.628777439158 O
18 O18	-4.787399571820	2.137521512782	-0.576062450433 O
19 H19	-4.214746921100	0.058667541004	1.015895169416 H
20 H20	-5.499514971979	1.635516078753	-0.149240243131 H
21 H21	-2.699266485364	3.215338963890	-1.518796251188 H
22 H22	-0.376400128521	2.481032199210	-1.060384592232 H
23 H23	0.386888948344	-0.486458309120	1.206711276086 H
24 H24	1.563990631594	1.805011146712	-0.533072363766 H
25 H25	2.268279713393	-0.906503681345	2.061837001988 H
26 H26	3.974675323047	-0.929351500385	1.611885746976 H
27 H27	2.720371534394	-3.023266478875	1.043851652157 H
28 H28	1.709817998450	-2.124754231091	-0.083596525934 H
29 H29	4.686765104158	-2.858948907964	-0.358915136955 H
30 H30	2.761648616409	-0.370155900789	-2.932503854268 H

31 H31	1.612883664715	-1.361332430224	-2.015459334747	H
32 H32	2.225097774454	-1.912387495395	-3.587744013035	H
33 H33	5.163829456925	-1.438662222825	-3.440010937186	H
34 H34	5.674643142157	-2.813448039320	-2.433873503055	H
35 H35	4.505790633035	-3.040591866072	-3.751878591323	H
36 H36	3.852268460925	1.853451381198	-0.749188189820	H
37 H37	4.699491906304	1.251053141676	0.705222502966	H
38 H38	4.584892844772	0.215861060012	-0.706860853061	H
39 O39	-4.109976985805	-1.959659798657	2.607244604851	O
40 H40	-4.397020668944	-2.841377650646	2.329036612732	H
41 H41	-2.635430412084	-1.295596774941	1.999624831392	H
42 H42	-4.328049483596	-1.871165962127	3.546340073505	H

Thermochemistry at 298.150 K, 1.000 Atm
E(el) = -532144.055290 kcal/mol
ZPVE = 225.672371 kcal/mol
Enthalpie(0K) = -531918.382919 kcal/mol
E(tr) = 0.888732 kcal/mol
E(rot) = 0.888732 kcal/mol
E(vib) = 238.059926 kcal/mol
H-E(el) = 240.429878 kcal/mol
Enthalpie = -531903.625411 kcal/mol
S(el) = 0.000000000000
S(tr) = 0.000067890982
S(rot) = 0.000055037155 (Symmetry number= 1)
S(vib) = 0.000134731058
G-E(el) = 192.223924 kcal/mol
Free Energy = -531951.831366 kcal/mol

Cartesian Coordinates and Energies of **Transition state IIIb – 3 (in presence of H_3O)**

#FREQ/B3LYP/6-31+g(d,p) //# SADDLE/MN15/6-31+g(d,p)
Charge = 1 Multiplicity = 1

----

1 C1	-1.403739080255	-0.231711326155	0.184905550824	C
2 C2	-2.632217523989	0.268351499094	-0.254128726000	C
3 C3	-2.673747238315	1.498192615374	-0.915393523323	C
4 C4	-1.494939581911	2.236615581938	-1.150758349307	C
5 C5	-0.294850453509	1.743392071948	-0.686094497385	C
6 C6	-0.210162557271	0.513662285677	0.009229140428	C
7 C7	1.037148871654	0.010787292492	0.547304020653	C
8 C8	2.055287733643	1.023235188476	0.929324589264	C
9 C9	3.394659784198	0.904898820855	1.010599245020	C
10 C10	4.153003636754	-0.316605469404	0.574259454875	C
11 C11	3.273758765026	-1.493615159687	0.168413720126	C
12 C12	2.152526427360	-1.071529589237	-0.756167741676	C
13 C13	1.160472771899	-1.956563915440	-1.150762089339	C
14 C14	0.977235117556	-3.260605414526	-0.447283476149	C
15 C15	0.236480640359	-1.614014265183	-2.260702255944	C
16 C16	4.240604053965	2.051163019363	1.485933656271	C
17 O17	-1.291695180635	-1.439620162980	0.771322330845	O
18 O18	-3.820342655291	2.033974603535	-1.370394645466	O
19 H19	-3.538884761318	-0.310073796607	-0.086658774431	H
20 H20	-4.591928531261	1.485901061111	-1.163903683532	H
21 H21	-1.565817292408	3.179500209457	-1.682277555977	H
22 H22	0.617149100145	2.311002887754	-0.857696566371	H
23 H23	0.916628796325	-0.838133270375	1.222196861433	H

24 H24	1.619776273362	1.988465195037	1.189038908731	H
25 H25	4.836725341704	-0.619837310041	1.377795464524	H
26 H26	4.804986658517	-0.019360569346	-0.261523452581	H
27 H27	3.885471250584	-2.257289257450	-0.330016636661	H
28 H28	2.854974112607	-1.975260427130	1.062662099835	H
29 H29	2.358390392451	-0.241243813054	-1.435677418989	H
30 H30	0.090938596800	-3.792187982903	-0.799732815472	H
31 H31	0.881215213189	-3.107408484909	0.636041415548	H
32 H32	1.862537827570	-3.893818479949	-0.599653162622	H
33 H33	-0.803039170335	-1.644776068959	-1.903115524358	H
34 H34	0.441447093098	-0.633541031495	-2.697584424304	H
35 H35	0.311695605719	-2.381775453024	-3.043376986749	H
36 H36	3.639417755674	2.928925699582	1.735743824570	H
37 H37	4.811732459436	1.757686315576	2.375107145065	H
38 H38	4.973140619772	2.334501565142	0.719958931391	H
39 O39	-3.791819002004	-2.442756900117	1.358757153424	O
40 H40	-4.158372934421	-3.270801423065	1.017923997380	H
41 H41	-2.169383429704	-1.845125429099	0.967382403705	H
42 H42	-4.129889182663	-2.330926878728	2.258807456263	H

Imag. Freq. -296.09 cm^{-1}
Thermochemistry at 298.150 K, 1.000 Atm
E(el) = -532132.995427 kcal/mol
ZPVE = 225.045526 kcal/mol
Enthalpie(0K) = -531907.949901 kcal/mol
E(tr) = 0.888732 kcal/mol
E(rot) = 0.888732 kcal/mol
E(vib) = 236.816712 kcal/mol
H-E(el) = 239.186664 kcal/mol
Enthalpie = -531893.808763 kcal/mol
S(el) = 0.000000000000
S(tr) = 0.000067890982
S(rot) = 0.000053950192 (Symmetry number= 1)
S(vib) = 0.000119994284
G-E(el) = 193.941202 kcal/mol
Free Energy = -531939.054224 kcal/mol

Cartesian Coordinates and Energies of **3 + H_3O**.
#FREQ/MN15/6-31+g(d,p) //# MIN/MN15/6-31+g(d,p)
Charge = 1 Multiplicity = 1

----

1 C1	-1.204030655433	-0.057040212533	-0.086736358476	C
2 C2	-2.407698303113	0.325019889972	-0.661344581872	C
3 C3	-2.613447313871	1.699827783966	-0.836192014615	C
4 C4	-1.617530370187	2.609602295199	-0.458644612399	C
5 C5	-0.422476166898	2.162321632938	0.096718187837	C
6 C6	-0.191947955957	0.799172888553	0.315528070267	C
7 C7	1.071859997713	0.139082646975	0.826104123051	C
8 C8	2.208345126378	1.096869006975	1.064742491306	C
9 C9	3.456604124721	0.927598950912	0.604074534999	C
10 C10	3.855430143481	-0.301696061911	-0.177988280152	C
11 C11	2.863389906300	-1.447241724134	0.007479039876	C
12 C12	1.442354668545	-0.926573548593	-0.225454772647	C
13 C13	0.406805229014	-2.059203212972	-0.271094851233	C
14 C14	0.608232016967	-3.153191036770	0.758996331062	C
15 C15	0.190963617246	-2.583884675778	-1.677110228433	C
16 C16	4.544111093728	1.935432581718	0.846366957994	C

17 O17	-0.941509416093	-1.433559163759	0.161408783213	O
18 O18	-3.746316675715	2.208941383672	-1.371874388636	O
19 H19	-3.157992855650	-0.407219439978	-0.951484416144	H
20 H20	-4.390923180234	1.525793065597	-1.603126418678	H
21 H21	-1.801854996049	3.667245523395	-0.617937900037	H
22 H22	0.345012145318	2.883768174560	0.360706640146	H
23 H23	0.828499453531	-0.377440175720	1.773304710018	H
24 H24	1.981808272319	1.976571000256	1.667467509189	H
25 H25	4.855742781408	-0.619818591937	0.141613092534	H
26 H26	3.950135540175	-0.042481346769	-1.243376112116	H
27 H27	3.099095495907	-2.272122783217	-0.678111126401	H
28 H28	2.945886244058	-1.835288680002	1.032160293184	H
29 H29	1.427568794121	-0.421365964179	-1.204763953718	H
30 H30	-0.256507781722	-3.826244183412	0.777114486380	H
31 H31	0.761739137840	-2.738321872803	1.760136179863	H
32 H32	1.485781309417	-3.747101953216	0.486960770567	H
33 H33	-0.497874367396	-3.436465807286	-1.691629753628	H
34 H34	-0.183649891461	-1.787374079010	-2.330435384133	H
35 H35	1.151945055685	-2.923992820814	-2.078504606591	H
36 H36	4.172659633785	2.815715881503	1.378160622343	H
37 H37	5.358308958822	1.492818769890	1.433332920937	H
38 H38	4.981720797845	2.264879960844	-0.104359881515	H
39 O39	-2.924669353214	-3.044561321771	-0.065174628680	O
40 H40	-3.200998420812	-3.570308348473	-0.829914525105	H
41 H41	-1.734975722711	-2.065207057193	0.041367060897	H
42 H42	-3.461848360194	-3.302416617063	0.698891684759	H

Thermochemistry at 298.150 K, 1.000 Atm
E(el) = -532149.632697 kcal/mol
ZPVE = 227.908484 kcal/mol
Enthalpie(0K) = -531921.724213 kcal/mol
E(tr) = 0.888732 kcal/mol
E(rot) = 0.888732 kcal/mol
E(vib) = 238.781954 kcal/mol
H-E(el) = 241.151906 kcal/mol
Enthalpie = -531908.480791 kcal/mol
S(el) = 0.000000000000
S(tr) = 0.000067890982
S(rot) = 0.000053671995 (Symmetry number= 1)
S(vib) = 0.000106269603
G-E(el) = 198.526270 kcal/mol
Free Energy = -531951.106427 kcal/mol

Scheme S4. Proposed formation mechanism of compound 4 (acid catalysis).
Plot of IRC transformation of **IIIb** in to **4 (acid catalysis)** (MN15/6-31+g(d,p)

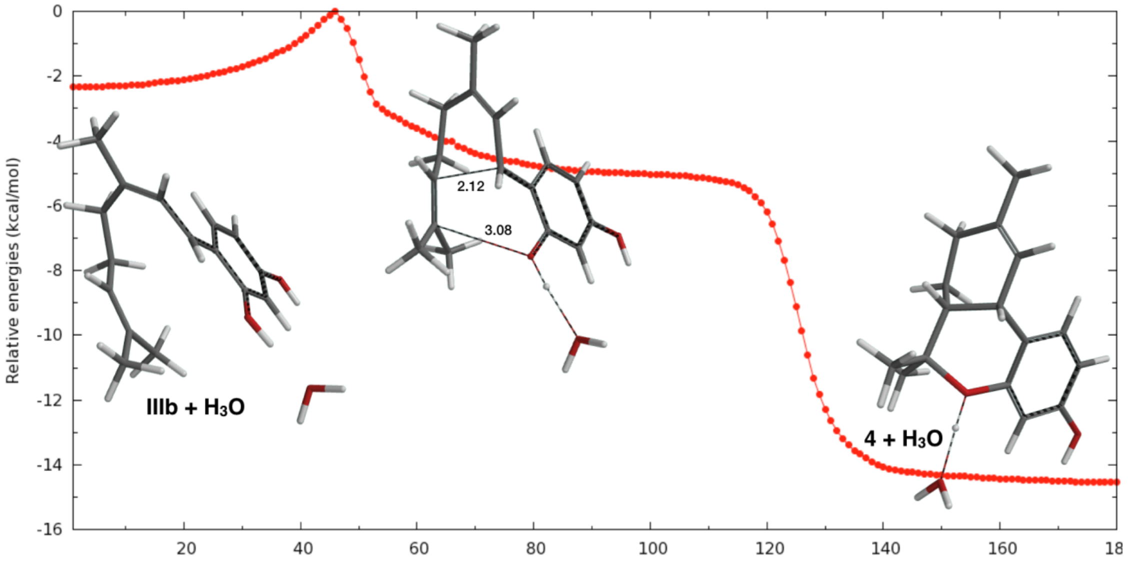

Cartesian Coordinates and Energies of **IIIb +H_3O**.
#FREQ/MN15/6-31+g(d,p) //# MIN/MN15/6-31+g(d,p)
Charge = 1 Multiplicity = 1

1	C1	-1.176216394583	0.201861753069	-1.182730370972 C
2	C2	-2.372665044920	0.743553452931	-0.716402942754 C
3	C3	-2.333635639463	1.849275178461	0.138596598849 C
4	C4	-1.105477855993	2.449998734902	0.536771033819 C
5	C5	0.065749141532	1.920095509545	0.076505340000 C
6	C6	0.087936706059	0.772915937874	-0.783718607657 C
7	C7	1.262099499645	0.236816423689	-1.306390323811 C
8	C8	2.612724842337	0.675513529245	-1.075561172630 C
9	C9	3.290590206504	0.887681541314	0.085621552292 C
10	C10	2.852003702041	0.532067297483	1.484982464181 C
11	C11	1.704019193344	-0.467055195507	1.704423153104 C
12	C12	1.744528110349	-1.648177535306	0.772719349932 C
13	C13	0.699926758135	-2.432422962859	0.435134569255 C
14	C14	-0.688634546301	-2.237575898098	0.983594585716 C
15	C15	0.869928123126	-3.613558417850	-0.480526364393 C
16	C16	4.703583583285	1.395535038182	0.012067259034 C
17	O17	-1.136432157521	-0.853214422299	-1.985514598516 O
18	O18	-3.438650378072	2.417914118519	0.619583682561 O
19	H19	-3.319951292630	0.300635318206	-1.016765695299 H
20	H20	-4.252203066648	1.991426498843	0.307067444047 H
21	H21	-1.142531601713	3.328635270130	1.171475122072 H
22	H22	1.014363011499	2.387240918523	0.327130421293 H
23	H23	1.130510018543	-0.491781816018	-2.107752833452 H
24	H24	3.197390592217	0.754797287823	-1.995067236285 H
25	H25	2.641702673821	1.466201676315	2.028798631521 H
26	H26	3.753961325724	0.136700876278	1.974551424496 H
27	H27	1.779536099551	-0.808776083845	2.745866778033 H
28	H28	0.732123193777	0.037496340832	1.640663064432 H
29	H29	2.723640710707	-1.908209562200	0.363623728854 H
30	H30	-1.444417776260	-2.504488334364	0.235185137787 H
31	H31	-0.881545005359	-1.214182052172	1.324808045744 H

32 H32	-0.845769234820	-2.899605454128	1.845587969131	H
33 H33	0.250795136880	-3.489808505872	-1.379509434420	H
34 H34	1.910800716771	-3.744632980581	-0.790296581062	H
35 H35	0.537987785896	-4.537366444256	0.010037510463	H
36 H36	4.963093889222	1.761816500285	-0.983884227558	H
37 H37	4.848846625129	2.206753161126	0.736142775261	H
38 H38	5.408645588313	0.601685939317	0.289918159310	H
39 O39	-3.636615232714	-1.649359188077	-2.686431682530	O
40 H40	-3.992268993322	-2.508910662130	-2.418295280527	H
41 H41	-2.035619727459	-1.173934113951	-2.252537234512	H
42 H42	-3.948733555871	-1.480828101717	-3.587464850322	H

Thermochemistry at 298.150 K, 1.000 Atm
E(el) = -532136.105584 kcal/mol
ZPVE = 225.296131 kcal/mol
Enthalpie(0K) = -531910.809452 kcal/mol
E(tr) = 0.888732 kcal/mol
E(rot) = 0.888732 kcal/mol
E(vib) = 237.568182 kcal/mol
H-E(el) = 239.938134 kcal/mol
Enthalpie = -531896.167449 kcal/mol
S(el) = 0.000000000000
S(tr) = 0.000067890982
S(rot) = 0.000053784938 (Symmetry number= 1)
S(vib) = 0.000128194535
G-E(el) = 193.189390 kcal/mol
Free Energy = -531942.916194 kcal/mol

Cartesian Coordinates and Energies of **Transition state IIIb – 4 (in presence of H_3O)**
#FREQ/B3LYP/6-31+g(d,p) //# SADDLE/MN15/6-31+g(d,p)
Charge = 1 Multiplicity = 1

----

1 C1	-1.229022191479	0.094571667054	-1.029441842926	C
2 C2	-2.413488433704	0.783532037160	-0.767091564425	C
3 C3	-2.368751566656	1.959792004674	-0.012769463043	C
4 C4	-1.146075924695	2.463950980334	0.483671658742	C
5 C5	0.017363088047	1.786091685936	0.203206439895	C
6 C6	0.023845573959	0.586595191886	-0.559096309008	C
7 C7	1.233941734055	-0.086196236124	-0.938114935470	C
8 C8	2.529995519278	0.634625968776	-0.907377857944	C
9 C9	3.335312959090	0.891712738259	0.137505180528	C
10 C10	3.119692171938	0.342905883283	1.525419175533	C
11 C11	1.925414059347	-0.600748863619	1.694776323662	C
12 C12	1.792975809402	-1.527844167525	0.514068614896	C
13 C13	0.717422607918	-2.367062677205	0.302824500860	C
14 C14	-0.521770707074	-2.220906341393	1.115639908368	C
15 C15	0.778254443524	-3.445528109527	-0.727477721059	C
16 C16	4.591226425412	1.700042239822	-0.037253254332	C
17 O17	-1.210565430981	-1.058172600709	-1.716269244492	O
18 O18	-3.469856594591	2.669289039800	0.277198858886	O
19 H19	-3.356848035658	0.395792163971	-1.146558591721	H
20 H20	-4.272064429676	2.285882809864	-0.108170976143	H
21 H21	-1.155411295653	3.387130625107	1.052917177056	H
22 H22	0.969857298626	2.186746145021	0.540521150360	H
23 H23	1.118226139104	-0.774788634331	-1.775848612858	H
24 H24	2.829302983464	0.989159380277	-1.896594595694	H

25 H25	3.043803345809	1.178661754385	2.234912143586	H
26 H26	4.045211466886	-0.182771850524	1.804343814147	H
27 H27	2.057806766057	-1.191308875275	2.611184742668	H
28 H28	0.993424674742	-0.035918334046	1.832063625609	H
29 H29	2.717934068976	-1.792155067848	-0.000993190884	H
30 H30	-1.327380276232	-2.868529352810	0.762693638772	H
31 H31	-0.869552678271	-1.175095032134	1.104922268715	H
32 H32	-0.307016791858	-2.451187846892	2.168391438207	H
33 H33	-0.057591346295	-3.347765516854	-1.429272192765	H
34 H34	1.721952793606	-3.439167127863	-1.279666743235	H
35 H35	0.669581503352	-4.421697226782	-0.235841121220	H
36 H36	4.705554312048	2.060188384364	-1.062815831713	H
37 H37	4.585157240722	2.566857139178	0.634806684586	H
38 H38	5.474274919753	1.104133787678	0.223845758474	H
39 O39	-3.683364169927	-1.572626827596	-2.795788758016	O
40 H40	-4.241794040121	-2.332352243111	-2.578252651512	H
41 H41	-2.095226085260	-1.287529493923	-2.091045952508	H
42 H42	-3.827766417647	-1.371249455889	-3.731539156255	H

Imag. Freq. -230.81 cm^{-1}
Thermochemistry at 298.150 K, 1.000 Atm
E(el) = -532133.777591 kcal/mol
ZPVE = 225.192403 kcal/mol
Enthalpie(0K) = -531908.585187 kcal/mol
E(tr) = 0.888732 kcal/mol
E(rot) = 0.888732 kcal/mol
E(vib) = 236.881613 kcal/mol
H-E(el) = 239.251565 kcal/mol
Enthalpie = -531894.526026 kcal/mol
S(el) = 0.000000000000
S(tr) = 0.000067890982
S(rot) = 0.000053763748 (Symmetry number= 1)
S(vib) = 0.000118258722
G-E(el) = 194.365695 kcal/mol
Free Energy = -531939.411896 kcal/mol

Cartesian Coordinates and Energies of **4 + H_3O**.
#FREQ/MN15/6-31+g(d,p) //# MIN/MN15/6-31+g(d,p)
Charge = 1 Multiplicity = 1

----

1 C1	-1.065390026904	0.198893416520	-0.733091801629	C
2 C2	-2.326388307068	0.699008157379	-0.441013749113	C
3 C3	-2.391728693352	2.048102678998	-0.068004208790	C
4 C4	-1.218167424514	2.810233768123	0.019070938947	C
5 C5	0.019309871722	2.241835387047	-0.266482976052	C
6 C6	0.120884598569	0.905480414589	-0.670981194906	C
7 C7	1.383318700678	0.123942326248	-0.971250558372	C
8 C8	2.647123425751	0.909687621840	-0.733419844576	C
9 C9	3.420940200371	0.765768520642	0.352441548782	C
10 C10	3.069272477119	-0.202204195226	1.455479473467	C
11 C11	1.648625064030	-0.760577407354	1.349019083331	C
12 C12	1.373819710728	-1.160394262313	-0.104771705133	C
13 C13	0.111675435835	-2.024414576899	-0.359728851156	C
14 C14	-0.647484200389	-2.477697960409	0.872713357782	C
15 C15	0.399985877241	-3.151174977268	-1.333443515206	C
16 C16	4.697794608353	1.535885051236	0.537447812479	C

17 O17	-0.895829838708	-1.151206316944	-1.144920784460	O
18 O18	-3.554962747385	2.669678887547	0.231575195835	O
19 H19	-3.216840997634	0.077819853712	-0.507211003111	H
20 H20	-4.323795958234	2.089895818263	0.139263617509	H
21 H21	-1.304232224506	3.849433625041	0.320365589980	H
22 H22	0.923952762590	2.837310875680	-0.176946691611	H
23 H23	1.351201142747	-0.179686508936	-2.030371330307	H
24 H24	2.930474076491	1.624945016868	-1.505460882083	H
25 H25	3.205356777991	0.292735333020	2.425687575323	H
26 H26	3.799600780056	-1.026308219754	1.441229227121	H
27 H27	1.545445884921	-1.612516043714	2.029203093043	H
28 H28	0.921181643519	0.000444053124	1.667093865737	H
29 H29	2.222333261127	-1.775249923502	-0.441151071577	H
30 H30	-1.535197863974	-3.053574645960	0.584266469271	H
31 H31	-0.948166262860	-1.625651107716	1.492082027793	H
32 H32	-0.013613644004	-3.133677085005	1.476211738099	H
33 H33	-0.514846034643	-3.682401160054	-1.620375937666	H
34 H34	0.895242851608	-2.766745828408	-2.231405075541	H
35 H35	1.070254643856	-3.868386291000	-0.849784644594	H
36 H36	4.911363126988	2.180428928038	-0.319615762050	H
37 H37	4.647518991781	2.159417608019	1.438531690688	H
38 H38	5.544104945798	0.850942146765	0.673467358920	H
39 O39	-3.055322568339	-2.373562294488	-1.811120490658	O
40 H40	-3.618295713119	-2.963785420751	-1.289260947504	H
41 H41	-1.757212600584	-1.648338938605	-1.377887046290	H
42 H42	-3.325497439629	-2.431361423526	-2.739956776828	H

Thermochemistry at 298.150 K, 1.000 Atm
E(el) = -532148.319104 kcal/mol
ZPVE = 227.937929 kcal/mol
Enthalpie(0K) = -531920.381175 kcal/mol
E(tr) = 0.888732 kcal/mol
E(rot) = 0.888732 kcal/mol
E(vib) = 238.812781 kcal/mol
H-E(el) = 241.182733 kcal/mol
Enthalpie = -531907.136371 kcal/mol
S(el) = 0.000000000000
S(tr) = 0.000067890982
S(rot) = 0.000053497471 (Symmetry number= 1)
S(vib) = 0.000107874586
G-E(el) = 198.289470 kcal/mol
Free Energy = -531950.029634 kcal/mol

Scheme S5. Proposed formation mechanism of compound 3 in presence of $GaCl_3$

Plot of IRC transformation of **IIIb** in to **3** in presence of $GaCl_3$ (MN15/6-31+g(d,p)

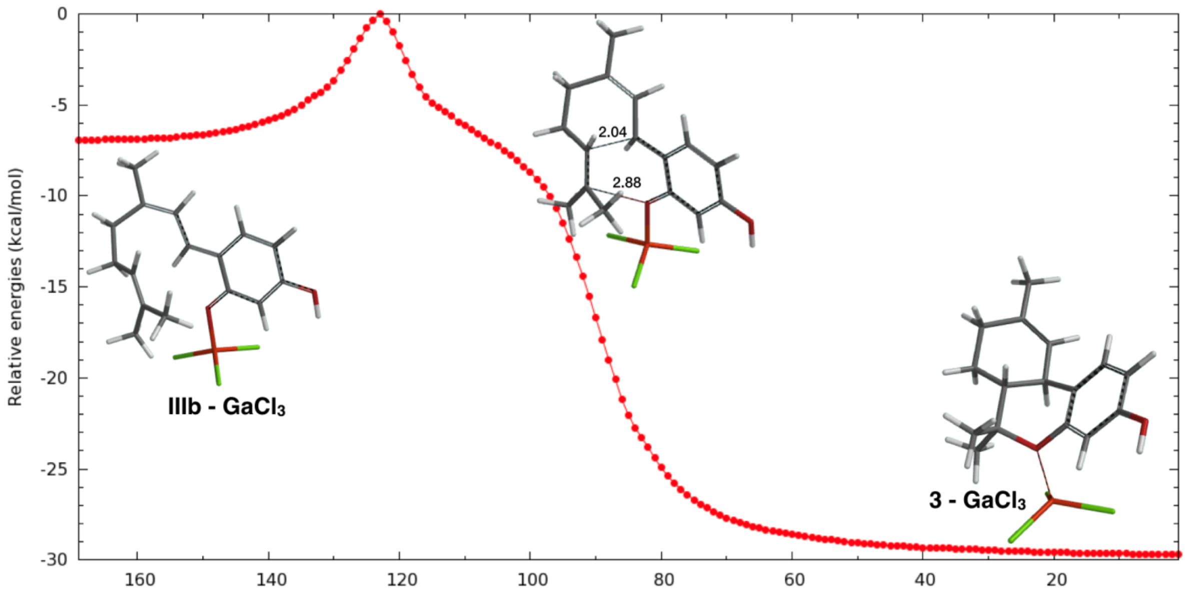

Cartesian Coordinates and Energies of **IIIb + $GaCl_3$**.
#FREQ/MN15/6-31+g(d,p) //# MIN/MN15/6-31+g(d,p)
Charge = 0 Multiplicity = 1

1	C1	-0.425821325846	0.488320823291	-4.202430620898 C
2	C2	-0.581810136026	0.679869332226	-1.409890114263 C
3	C3	-0.839012856808	-0.624225016279	-3.494206377269 C
4	C4	-0.081735213992	1.723047883761	-3.561012628947 C
5	C5	-0.159390309152	1.802141195768	-2.205714606327 C
6	C6	-0.947900193917	-0.559814489724	-2.089395343478 C
7	H7	-1.094107285208	-1.547069090541	-4.009294712304 H
8	H8	0.238839326042	2.553370025563	-4.181079758751 H
9	O9	-0.316176494735	0.486933875634	-5.538406225787 O
10	H10	-0.575163703563	-0.369658513172	-5.914155978949 H
11	O11	-1.344561971364	-1.556158919095	-1.368494995560 O
12	C12	-0.597160376396	0.674107425347	-0.026124757568 C
13	H13	-0.900014078772	-0.265371108786	0.424329603796 H
14	C14	-0.312373955726	1.799181188496	0.820670967616 C
15	H15	-0.376564442426	2.786654964358	0.364350014001 H
16	C16	1.406542008867	-0.906676955785	1.177465148087 C
17	H17	2.019609983474	-0.029669337871	0.955690039206 H
18	C18	1.466956766226	-1.963422964040	0.345455214966 C
19	C19	2.300334225721	-1.936737121408	-0.903850525805 C
20	H20	2.854702720330	-0.997741659070	-1.007549577132 H
21	H21	3.011863555413	-2.772284168626	-0.924915657314 H
22	H22	1.650397140348	-2.066708978957	-1.782499836892 H
23	C23	0.703326208648	-3.234448369142	0.615232836011 C
24	H24	1.192989808170	-3.815403545953	1.409092132352 H
25	H25	-0.324224375649	-3.040856227723	0.943415315881 H
26	H26	0.652097047081	-3.868747020600	-0.275455962593 H
27	C27	0.517713017281	-0.835937256660	2.395629675835 C
28	H28	-0.476152941824	-1.247237918935	2.166597440657 H
29	H29	0.907539961861	-1.484301138901	3.193819187236 H

30 C30	0.385211661542	0.570135797566	2.991797741693	C
31 H31	-0.354235532859	0.554580250957	3.806954425810	H
32 H32	1.333341955731	0.828116885539	3.490095023296	H
33 C33	0.063748348211	1.766169060166	2.128590401306	C
34 C34	0.286720412383	3.071659320850	2.843274625585	C
35 H35	-0.046294531428	3.928822797774	2.252642186395	H
36 H36	-0.247431299219	3.076266791563	3.801713671750	H
37 H37	1.351278723786	3.200326540018	3.078496190282	H
38 H38	0.119656756917	2.725516762486	-1.706777128662	H
39 Ga39	-2.020415352521	-3.226316870955	-1.983690676977	Ga
40 Cl40	-0.343801050359	-4.155237691826	-2.999489697711	Cl
41 Cl41	-3.607884735124	-2.721869483015	-3.362353221231	Cl
42 Cl42	-2.705686846092	-4.218547529945	-0.212555455986	Cl

Thermochemistry at 298.150 K, 1.000 Atm
E(el) = -2557190.964096 kcal/mol
ZPVE = 205.785084 kcal/mol
Enthalpie(0K) = -2556985.179012 kcal/mol
E(tr) = 0.888732 kcal/mol
E(rot) = 0.888732 kcal/mol
E(vib) = 220.296890 kcal/mol
H-E(el) = 222.666842 kcal/mol
Enthalpie = -2556968.297254 kcal/mol
S(el) = 0.000000000000
S(tr) = 0.000070088680
S(rot) = 0.000056762976 (Symmetry number= 1)
S(vib) = 0.000156874328
G-E(el) = 169.584001 kcal/mol
Free Energy = -2557021.380095 kcal/mol

Cartesian Coordinates and Energies of **Transition state IIIb–$GaCl_3$ in 3-$GaCl_3$**

#FREQ/B3LYP/6-31+g(d,p) //# SADDLE/MN15/6-31+g(d,p)
Charge = 0 Multiplicity = 1
----

1 C1	-0.304618181215	0.536270484322	-4.101668567878	C
2 C2	-0.394742716887	0.503311268351	-1.305712595728	C
3 C3	-0.829985757031	-0.579605355368	-3.463144686304	C
4 C4	0.201682044450	1.640379038329	-3.374165696798	C
5 C5	0.155968609844	1.608760695958	-1.998338739141	C
6 C6	-0.874573840307	-0.614772999865	-2.058553427966	C
7 H7	-1.199222850576	-1.428585857208	-4.035216592157	H
8 H8	0.610032852398	2.483197844358	-3.921894138977	H
9 O9	-0.238270423482	0.624840599299	-5.448776779866	O
10 H10	-0.621635868621	-0.160683602259	-5.867272850907	H
11 O11	-1.291160721362	-1.653015679696	-1.369100381538	O
12 C12	-0.453499922715	0.395145397015	0.122390300603	C
13 H13	-1.132518879649	-0.386728912885	0.465702075028	H
14 C14	-0.386671696896	1.638921293550	0.930523459010	C
15 H15	-0.789686954121	2.513321835568	0.417407477077	H
16 C16	1.140469298048	-0.615204105076	0.896576221434	C
17 H17	1.841993833276	0.171268940375	0.606533346365	H
18 C18	1.168726850107	-1.769539521419	0.124241066861	C
19 C19	1.859710113477	-1.769578212552	-1.191663418759	C
20 H20	2.143040625219	-0.762477483439	-1.515249080656	H
21 H21	2.773239430953	-2.377304208051	-1.106190021325	H

22 H22	1.241783422918	-2.260037045534	-1.954683723411 H
23 C23	0.550844568395	-3.036050252141	0.612580446768 C
24 H24	1.106742713711	-3.402734005984	1.488942593755 H
25 H25	-0.483778950331	-2.869542483080	0.940064558348 H
26 H26	0.540257789339	-3.812475055214	-0.157395084586 H
27 C27	0.715894888606	-0.651178326017	2.349118946704 C
28 H28	-0.293643360724	-1.078754656254	2.425279479719 H
29 H29	1.373076359278	-1.328385955999	2.911931808347 H
30 C30	0.744128143260	0.732999550808	2.987989590301 C
31 H31	0.278405320476	0.695091634079	3.982234700753 H
32 H32	1.784900692417	1.046978552799	3.167192702628 H
33 C33	0.078452124052	1.813705563274	2.179967224264 C
34 C34	0.022607394547	3.161862432541	2.843519325000 C
35 H35	-0.424944426242	3.918243329491	2.192952455087 H
36 H36	-0.565051223854	3.109657643609	3.768700265885 H
37 H37	1.029080013049	3.496358500797	3.126447125309 H
38 H38	0.550964587641	2.447403743467	-1.428865220687 H
39 Ga39	-2.041504528614	-3.256386706741	-2.007303993497 Ga
40 Cl40	-0.376647477087	-4.255359508070	-2.993514194153 Cl
41 Cl41	-3.630468379752	-2.763765155148	-3.380544071761 Cl
42 Cl42	-2.705491871918	-4.256340425584	-0.220699713315 Cl

Imag. Freq. -349.06 cm^{-1}
Thermochemistry at 298.150 K,   1.000 Atm
E(el)   = -2557184.045186 kcal/mol
ZPVE   = 205.470059 kcal/mol
Enthalpie(0K)   = -2556978.575127 kcal/mol
E(tr)   = 0.888732 kcal/mol
E(rot)   = 0.888732 kcal/mol
E(vib)   = 219.214080 kcal/mol
H-E(el)   = 221.584032 kcal/mol
Enthalpie   = -2556962.461154 kcal/mol
S(el)   = 0.000000000000
S(tr)   = 0.000070088680
S(rot)   = 0.000056647973 (Symmetry number= 1)
S(vib)   = 0.000143683086
G-E(el)   = 170.990683 kcal/mol
Free Energy   = -2557013.054503 kcal/mol

Cartesian Coordinates and Energies of **3-GaCl$_3$**
#FREQ/MN15/6-31+g(d,p) //# MIN/MN15/6-31+g(d,p)
Charge = 0 Multiplicity = 1
----

1 C1	-0.489742971055	0.752069532754	-4.012823076760 C
2 C2	-0.796406094518	0.461274972342	-1.229971727045 C
3 C3	-0.362893948931	-0.516516695633	-3.439047691412 C
4 C4	-0.754063780376	1.864528991147	-3.207480331498 C
5 C5	-0.883755813252	1.720912496835	-1.825853115202 C
6 C6	-0.540929559957	-0.616988682920	-2.068423656782 C
7 H7	-0.148833514407	-1.402379910373	-4.033887645517 H
8 H8	-0.841085233752	2.836919172733	-3.682568354262 H
9 O9	-0.358191437073	0.957889142478	-5.352007314106 O
10 H10	-0.295316856538	0.118015207368	-5.828126265113 H
11 O11	-0.458440605482	-1.866499058361	-1.432226877308 O
12 C12	-0.708706085989	0.125958112570	0.242418448719 C
13 H13	-1.512249759020	-0.593052106456	0.490647434950 H
14 C14	-0.799716168440	1.319282039280	1.151074197864 C

15 H15	-1.657502107062	1.977692138267	1.004484735502	H
16 C16	0.648347872771	-0.597416582129	0.372071404500	C
17 H17	1.386418248588	0.037427876900	-0.146134525936	H
18 C18	0.606378454996	-1.959519166128	-0.362078517231	C
19 C19	1.902768143324	-2.249153849682	-1.097452370703	C
20 H20	2.076388162691	-1.488305854213	-1.867755601419	H
21 H21	2.737780604388	-2.213216897000	-0.387248313791	H
22 H22	1.871493157717	-3.239159992880	-1.564165098276	H
23 C23	0.198387478080	-3.104633422256	0.544826250737	C
24 H24	1.016605186858	-3.301752998753	1.244555118143	H
25 H25	-0.706790541336	-2.874446729452	1.112781033220	H
26 H26	0.034486733939	-4.021501337625	-0.030780784180	H
27 C27	1.084256991399	-0.689207525563	1.834802078355	C
28 H28	0.313426487023	-1.209932797716	2.418760390631	H
29 H29	2.014643933162	-1.265132811222	1.930263944388	H
30 C30	1.274263757402	0.718987683708	2.395703586731	C
31 H31	1.431792071892	0.672702083185	3.481784174600	H
32 H32	2.185797958661	1.176442398228	1.979785640266	H
33 C33	0.092146208816	1.614962666341	2.107721239678	C
34 C34	-0.024223420617	2.846685695901	2.961935704951	C
35 H35	-0.877218557751	3.465225097096	2.667113962722	H
36 H36	-0.139331075488	2.577997969024	4.019979400039	H
37 H37	0.886250672259	3.455747003529	2.886986185748	H
38 H38	-1.044749161233	2.600354586664	-1.207968904188	H
39 Ga39	-1.795146310617	-3.253234934049	-1.939834465203	Ga
40 Cl40	-0.502167081705	-4.863499860792	-2.539130179541	Cl
41 Cl41	-2.927910589797	-2.456920568150	-3.574567532041	Cl
42 Cl42	-3.037058806168	-3.505332889022	-0.208702756354	Cl

Thermochemistry at 298.150 K, 1.000 Atm
E(el) = -2557213.711564 kcal/mol
ZPVE = 208.926834 kcal/mol
Enthalpie(0K) = -2557004.784730 kcal/mol
E(tr) = 0.888732 kcal/mol
E(rot) = 0.888732 kcal/mol
E(vib) = 221.865785 kcal/mol
H-E(el) = 224.235737 kcal/mol
Enthalpie = -2556989.475827 kcal/mol
S(el) = 0.000000000000
S(tr) = 0.000070088680
S(rot) = 0.000056307844 (Symmetry number= 1)
S(vib) = 0.000134055429
G-E(el) = 175.507280 kcal/mol
Free Energy = -2557038.204285 kcal/mol

Scheme S6. Proposed formation mechanism of compound 6

Plot of IRC transformation of **IVb** in to **6** (MN15/6-31+g(d,p)

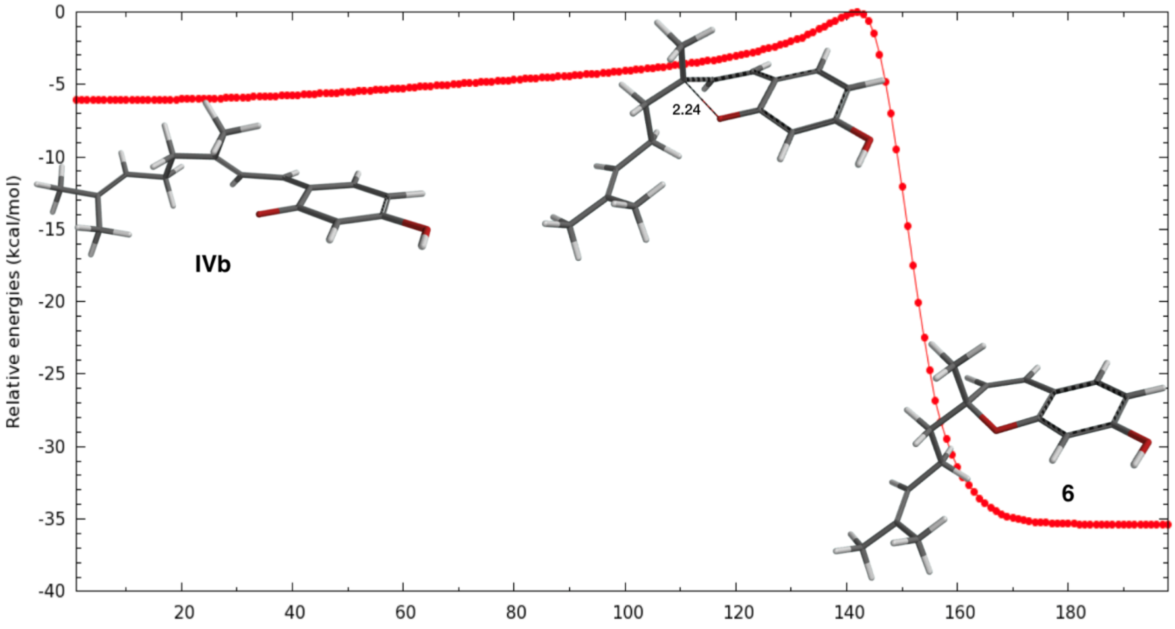

Cartesian Coordinates and Energies of **IVb**
#FREQ/MN15/6-31+g(d,p) //# MIN/MN15/6-31+g(d,p)
Charge = 0 Multiplicity = 1

----

1 C1	1.243536622611	-0.423336886778	1.505499280690 C
2 C2	0.889439328150	-0.704580735106	2.888168782236 C
3 C3	1.834311104334	-0.814105630904	3.862322215972 C
4 C4	3.247451805352	-0.678972445949	3.580361225858 C
5 C5	3.642641353433	-0.422333259131	2.313400199773 C
6 C6	2.702093514093	-0.241418368896	1.216833175402 C
7 C7	3.229618515002	-0.039366713051	-0.034152922952 C
8 C8	2.634472337902	0.243996738969	-1.323363124217 C
9 C9	1.676201131706	1.152850463425	-1.629934815623 C
10 C10	1.203715947778	1.356646192020	-3.048138311990 C
11 C11	1.769453852803	0.429897097157	-4.129444407709 C
12 C12	1.174989521460	0.733240343174	-5.482287846644 C
13 C13	0.017565445352	0.253520802283	-5.965639712726 C
14 C14	-0.876653591600	-0.701019651266	-5.217782997704 C
15 C15	-0.487879235358	0.652191159082	-7.327805749704 C
16 C16	1.027942666538	2.060699901708	-0.633703882282 C
17 O17	0.376786103880	-0.372655970024	0.623317265428 O
18 O18	1.547873144856	-1.069186296480	5.161907860928 O
19 H19	-0.170796265129	-0.827302038816	3.100097871318 H
20 H20	0.591409384551	-1.158782664649	5.287451508044 H
21 H21	3.947313314918	-0.798430594242	4.401220014151 H
22 H22	4.702484642640	-0.325742928923	2.083713367751 H
23 H23	4.317167126462	-0.154802074907	-0.080852140674 H
24 H24	3.135961368328	-0.258675678603	-2.150968468037 H
25 H25	0.105989831205	1.256621241668	-3.039149452830 H
26 H26	1.385036196762	2.404943536893	-3.332164206734 H
27 H27	1.582841960097	-0.613546489008	-3.844076693887 H
28 H28	2.859516552788	0.547854298679	-4.181515218434 H
29 H29	1.729017286615	1.438782669715	-6.104819302138 H

30 H30	-0.486460833966	-0.979878623431	-4.235334547277	H
31 H31	-1.031124757572	-1.619594905888	-5.799399371254	H
32 H32	-1.868685154136	-0.253430367829	-5.070683646641	H
33 H33	-0.611638666383	-0.229793713208	-7.970435219108	H
34 H34	-1.474197793108	1.129444602487	-7.254239573061	H
35 H35	0.195175161576	1.347548176774	-7.825624941300	H
36 H36	0.928910628882	3.070355322249	-1.051447270616	H
37 H37	0.024859172124	1.682376555447	-0.398371004543	H
38 H38	1.582162775889	2.112248480703	0.307944198735	H

Thermochemistry at 298.150 K, 1.000 Atm
E(el) = -483963.519872 kcal/mol
ZPVE = 201.740073 kcal/mol
Enthalpie(0K) = -483761.779798 kcal/mol
E(tr) = 0.888732 kcal/mol
E(rot) = 0.888732 kcal/mol
E(vib) = 211.958963 kcal/mol
H-E(el) = 214.328915 kcal/mol
Enthalpie = -483749.190957 kcal/mol
S(el) = 0.000000000000
S(tr) = 0.000067534655
S(rot) = 0.000054092284 (Symmetry number= 1)
S(vib) = 0.000109310231
G-E(el) = 171.122436 kcal/mol
Free Energy = -483792.397436 kcal/mol

Cartesian Coordinates and Energies of **Transition state IVb in 6**
#FREQ/ MN15/6-31+g(d,p) //# SADDLE/MN15/6-31+g(d,p)
Charge = 0 Multiplicity = 1
----

1 C1	1.463030649913	-0.087861515438	0.727120237199	C
2 C2	0.769941304351	-0.951894144073	1.644811257194	C
3 C3	1.299114880776	-1.278624361425	2.868284122166	C
4 C4	2.617263534661	-0.878795488764	3.248776556707	C
5 C5	3.360722227783	-0.167750418927	2.353516133524	C
6 C6	2.854809994734	0.251575000370	1.077419037704	C
7 C7	3.769219100258	0.631357503473	0.081352223709	C
8 C8	3.418262861235	1.029409091844	-1.215246006417	C
9 C9	2.213489352024	1.660922376022	-1.512895192544	C
10 C10	1.604957896972	1.573460406057	-2.882532800687	C
11 C11	1.892843628217	0.268585993472	-3.638300706731	C
12 C12	0.986798245886	0.094228541532	-4.831882083434	C
13 C13	-0.285196089268	-0.334306062694	-4.777742444891	C
14 C14	-0.985799584169	-0.720204395330	-3.499807974485	C
15 C15	-1.123244199550	-0.462214547914	-6.023059478729	C
16 C16	1.654466465065	2.746850059688	-0.643435375431	C
17 O17	0.882858966407	0.296558903568	-0.329332960728	O
18 O18	0.619526053602	-2.006028383568	3.792178357796	O
19 H19	-0.236973611095	-1.255756002610	1.364882346658	H
20 H20	-0.253407329957	-2.251781406030	3.452631325594	H
21 H21	2.997348403113	-1.181545046091	4.218884135714	H
22 H22	4.387884435491	0.099003159740	2.597275159630	H
23 H23	4.820883546045	0.418832555442	0.286472518071	H
24 H24	4.065722832759	0.717844619397	-2.035012007204	H
25 H25	0.519006786230	1.674170159761	-2.744848306232	H
26 H26	1.910394825074	2.450442076651	-3.475026582393	H

27 H27	1.767355688772	-0.558429872645	-2.926797964772	H
28 H28	2.939586622333	0.242359053301	-3.964596550565	H
29 H29	1.394292167006	0.363568045571	-5.808052175222	H
30 H30	-0.368969954203	-0.601489894530	-2.603813879204	H
31 H31	-1.322864339103	-1.764543593714	-3.551721018319	H
32 H32	-1.889309664051	-0.109182245756	-3.367678916173	H
33 H33	-1.471984240058	-1.495661775496	-6.152140666372	H
34 H34	-2.021067435164	0.166727284717	-5.956056857972	H
35 H35	-0.565336280255	-0.172672013823	-6.919224596942	H
36 H36	1.920948001813	3.709606113549	-1.104573966967	H
37 H37	0.563998329400	2.678020597904	-0.595486667134	H
38 H38	2.069027951727	2.722908771849	0.366723867681	H

Imag. Freq. -261.14 cm^{-1}
Thermochemistry at 298.150 K, 1.000 Atm
E(el) = -483957.419161 kcal/mol
ZPVE = 201.172859 kcal/mol
Enthalpie(0K) = -483756.246302 kcal/mol
E(tr) = 0.888732 kcal/mol
E(rot) = 0.888732 kcal/mol
E(vib) = 210.792563 kcal/mol
H-E(el) = 213.162515 kcal/mol
Enthalpie = -483744.256646 kcal/mol
S(el) = 0.000000000000
S(tr) = 0.000067534655
S(rot) = 0.000053782880 (Symmetry number= 1)
S(vib) = 0.000099211670
G-E(el) = 171.903282 kcal/mol
Free Energy = -483785.515879 kcal/mol

Cartesian Coordinates and Energies of **6**
#FREQ/MN15/6-31+g(d,p) //# MIN/MN15/6-31+g(d,p)
Charge = 0 Multiplicity = 1
----

1 C1	1.683506402484	-0.037143800307	0.714216832626	C
2 C2	0.809729481838	-0.636950068580	1.619019339414	C
3 C3	1.310461069139	-1.137943935767	2.822960377526	C
4 C4	2.673953333913	-1.045893808285	3.124319731038	C
5 C5	3.529629485529	-0.454711394094	2.201224941649	C
6 C6	3.060003855558	0.058834309098	0.985153694749	C
7 C7	3.913011728210	0.635490257729	-0.050046763962	C
8 C8	3.359186537855	1.267750327340	-1.094619921890	C
9 C9	1.861903836688	1.466037699672	-1.157971292862	C
10 C10	1.335292584417	1.415395527036	-2.591956015536	C
11 C11	1.688505108082	0.119942690760	-3.335237473852	C
12 C12	0.991840955216	0.025591674749	-4.669513933659	C
13 C13	-0.272024953203	-0.385561987520	-4.863158237705	C
14 C14	-1.183267181990	-0.838141714192	-3.750985121736	C
15 C15	-0.887194205053	-0.420746003100	-6.237841874874	C
16 C16	1.477383702954	2.782698166593	-0.479382588865	C
17 O17	1.177657757391	0.391147975007	-0.473596806602	O
18 O18	0.499738140379	-1.726366083190	3.748375910630	O
19 H19	-0.247644453969	-0.701049705853	1.370335791471	H
20 H20	-0.415035015138	-1.747802382921	3.434937511673	H
21 H21	3.034269532241	-1.443490624355	4.067640565203	H
22 H22	4.594390981121	-0.391958404872	2.417979262108	H

23 H23	4.991777413192	0.525176769520	0.043472099947	H
24 H24	3.958849499401	1.705936818224	-1.890598022055	H
25 H25	0.242554371572	1.526355030616	-2.549749557133	H
26 H26	1.724499267812	2.287609162187	-3.136032130444	H
27 H27	1.423885763264	-0.728193218664	-2.692482074743	H
28 H28	2.775293040921	0.064982939117	-3.478438699869	H
29 H29	1.558151327272	0.352130019294	-5.544055743890	H
30 H30	-0.715346501733	-0.787622963720	-2.763457155919	H
31 H31	-1.516317386175	-1.870682684826	-3.922589620496	H
32 H32	-2.089284933428	-0.217286675149	-3.728142876801	H
33 H33	-1.213615296278	-1.437992193259	-6.493040358305	H
34 H34	-1.779954626224	0.217392080415	-6.283939648999	H
35 H35	-0.182881044188	-0.083374921605	-7.004781079114	H
36 H36	1.978927106390	3.622875599842	-0.972567209748	H
37 H37	0.392609715425	2.930995353790	-0.534269731476	H
38 H38	1.785717903458	2.765547866280	0.572576718834	H

Thermochemistry at 298.150 K, 1.000 Atm
E(el) = -483992.803647 kcal/mol
ZPVE = 203.086217 kcal/mol
Enthalpie(0K) = -483789.717430 kcal/mol
E(tr) = 0.888732 kcal/mol
E(rot) = 0.888732 kcal/mol
E(vib) = 212.606735 kcal/mol
H-E(el) = 214.976687 kcal/mol
Enthalpie = -483777.826960 kcal/mol
S(el) = 0.000000000000
S(tr) = 0.000067534655
S(rot) = 0.000053692914 (Symmetry number= 1)
S(vib) = 0.000098921490
G-E(el) = 173.788576 kcal/mol
Free Energy = -483819.015071 kcal/mol

Scheme S7. Proposed formation mechanism of compound 7

Plot of IRC transformation of **6 + 2-GaCl₃** in to **7a** (MN15/6-31+g(d,p)

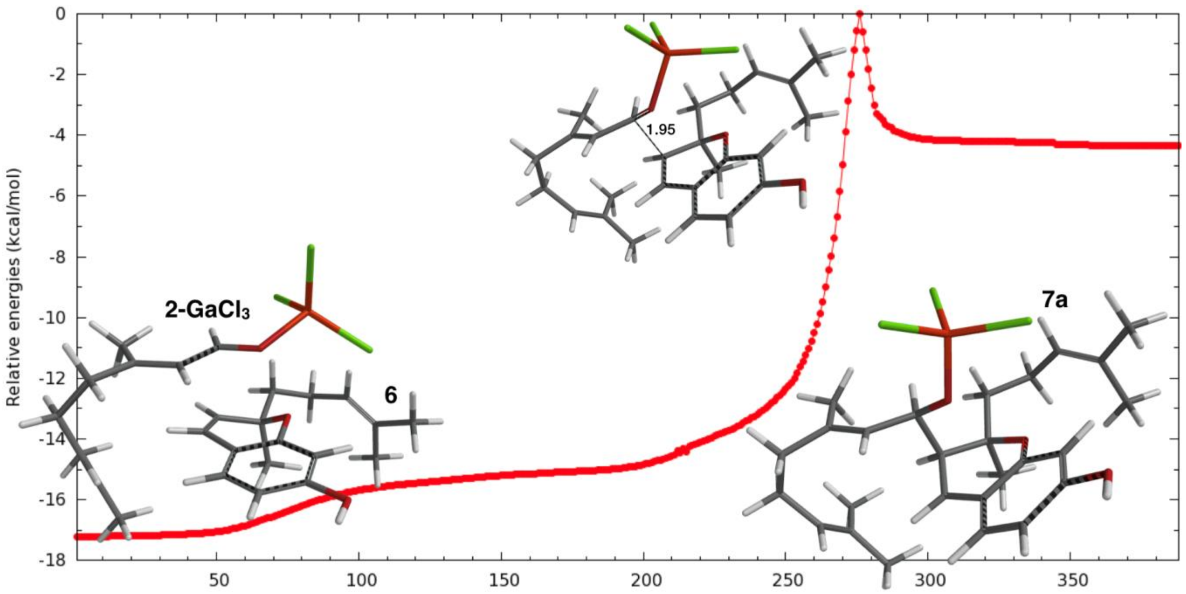

Cartesian Coordinates and Energies of **6 + 2-GaCl₃**
#FREQ/MN15/6-31+g(d,p) //# MIN/MN15/6-31+g(d,p)
Charge = 0 Multiplicity = 1

----

1 C1	-1.095191283506	1.757287030155	0.682288781648 C
2 O2	0.002726286555	1.636270338321	1.273576122676 O
3 C3	-2.335634417376	1.508215174487	1.357024695253 C
4 C4	-3.552433275338	1.639738865173	0.762524819374 C
5 C5	-3.767503967031	1.954694704180	-0.688435729226 C
6 C6	-4.804007503527	1.477337903595	1.584866993503 C
7 C7	-5.836150026625	0.498166112278	0.988828336644 C
8 C8	-5.312827581157	-0.911545787406	0.950623317099 C
9 C9	-5.173038579273	-1.730787805951	-0.106417596986 C
10 C10	-5.522094436023	-1.383064813123	-1.530585311103 C
11 C11	-4.613520526722	-3.120691221224	0.060560809672 C
12 C12	-0.734211313511	-2.070728978028	2.768454455183 C
13 C13	-1.512748774529	-1.780047952731	1.650681586143 C
14 C14	-0.925474224358	-1.463166647398	0.420860999966 C
15 C15	0.481777931774	-1.423243133145	0.347817174259 C
16 C16	1.279476791810	-1.711705279294	1.449154036358 C
17 C17	0.663888233502	-2.040229799745	2.658444341003 C
18 O18	1.079727176722	-1.013368235597	-0.801078594328 O
19 C19	-1.663130209293	-1.069286863033	-0.776368424165 C
20 C20	-1.024500474360	-0.987157287367	-1.957022881133 C
21 C21	0.433283354674	-1.398798953179	-2.033996847950 C
22 C22	0.546233512282	-2.918927230655	-2.191766574367 C
23 C23	1.157246386887	-0.647075254151	-3.153572183683 C
24 C24	2.581817545419	-1.122890941813	-3.481417838911 C
25 C25	3.519223311180	-1.057674235882	-2.306352886480 C
26 C26	4.315307385265	-2.027400369707	-1.828423846740 C
27 C27	4.439595221108	-3.415677967245	-2.403428599532 C
28 C28	5.166972937933	-1.793403241399	-0.606286875940 C
29 O29	1.475911370100	-2.332628448239	3.710757122710 O

30 Ga30	1.670536869347	2.420158793345	0.603763159558	Ga
31 Cl31	3.276257444933	1.116586713420	1.105770842553	Cl
32 Cl32	1.245019799914	2.724858454090	-1.494261936053	Cl
33 H33	-1.081255538616	2.088632087335	-0.363142122447	H
34 H34	-2.258211614546	1.227784438701	2.405741943679	H
35 H35	-4.552058046792	2.713166378123	-0.796657098185	H
36 H36	-2.874020512462	2.292364096826	-1.214937242627	H
37 H37	-4.127411126808	1.049476835409	-1.196950962604	H
38 H38	-4.536260638979	1.162015795857	2.600635002901	H
39 H39	-5.267810562360	2.472338982087	1.667564159607	H
40 H40	-6.730025154947	0.538856572224	1.625244056371	H
41 H41	-6.152933942346	0.841899623833	-0.001987208361	H
42 H42	-5.013370232013	-1.304698148677	1.927002889034	H
43 H43	-5.973353742837	-0.394171979064	-1.647395168804	H
44 H44	-4.621646993689	-1.431545340405	-2.161601233421	H
45 H45	-6.223940564855	-2.121413748584	-1.938179234838	H
46 H46	-3.667484829966	-3.226261016414	-0.490501709548	H
47 H47	-4.425289414525	-3.363336493568	1.111048765317	H
48 H48	-5.305582793287	-3.868325949482	-0.347474398523	H
49 H49	-1.199964736065	-2.323956831967	3.719273225011	H
50 H50	-2.599094996963	-1.780324057980	1.728126428040	H
51 H51	2.360766754354	-1.668094215780	1.367481875463	H
52 H52	-2.721791200222	-0.835847972472	-0.673872114991	H
53 H53	-1.531345188553	-0.682357869353	-2.871628163768	H
54 H54	-0.043299700982	-3.429628975611	-1.421942597884	H
55 H55	0.170627660831	-3.222044197715	-3.176398882528	H
56 H56	1.594055869601	-3.224818229849	-2.095249066544	H
57 H57	0.537904402263	-0.734910379873	-4.057637173592	H
58 H58	1.173910786632	0.412033726695	-2.872964121799	H
59 H59	2.548641925223	-2.130491017907	-3.912969787659	H
60 H60	2.957530639014	-0.466135060079	-4.279658315060	H
61 H61	3.504729382427	-0.106060382939	-1.768353179935	H
62 H62	3.813386452457	-3.585505667855	-3.283695842817	H
63 H63	5.480232398648	-3.618996493372	-2.690038362648	H
64 H64	4.173470483144	-4.165756857420	-1.644923147023	H
65 H65	6.226557034020	-1.990772988120	-0.819494526756	H
66 H66	5.071040619119	-0.768820981661	-0.236865890844	H
67 H67	4.876601286018	-2.478815472083	0.203479761332	H
68 H68	0.954159778339	-2.530288930167	4.500774828794	H
69 Cl69	1.621737631760	4.267967536446	1.695140226952	Cl

Thermochemistry at 298.150 K, 1.000 Atm
E(el) = -2849216.461514 kcal/mol
ZPVE = 356.541422 kcal/mol
Enthalpie(0K) = -2848859.920092 kcal/mol
E(tr) = 0.888732 kcal/mol
E(rot) = 0.888732 kcal/mol
E(vib) = 379.653406 kcal/mol
H-E(el) = 382.023358 kcal/mol
Enthalpie = -2848834.438156 kcal/mol
S(el) = 0.000000000000
S(tr) = 0.000071563058
S(rot) = 0.000059745507 (Symmetry number= 1)
S(vib) = 0.000255969982
G-E(el) = 309.566669 kcal/mol
Free Energy = -2848906.894845 kcal/mol

Cartesian Coordinates and Energies of **Transition state 6 + 2-GaCl$_3$ in 7a**
#FREQ/B3LYP/6-31+g(d,p) //# SADDLE/MN15/6-31+g(d,p)
Charge = 0 Multiplicity = 1
----

1 C1	-1.073326133619	1.102033106773	0.066736532017	C
2 O2	-0.068051498771	1.150413516074	0.912088939875	O
3 C3	-2.417403425008	1.486134871927	0.543530292833	C
4 C4	-3.401513883072	1.889042529779	-0.280561724933	C
5 C5	-3.215382980677	2.062708207309	-1.769861236871	C
6 C6	-4.806775979218	2.064584723016	0.228697665419	C
7 C7	-5.730158415592	0.976744485766	-0.368930088152	C
8 C8	-5.259324819141	-0.410400915304	-0.024490922896	C
9 C9	-4.902834840633	-1.408144927412	-0.852765062826	C
10 C10	-4.899092922511	-1.333206765663	-2.358704527902	C
11 C11	-4.471901715754	-2.749940517749	-0.315135172197	C
12 C12	0.554633653495	-2.357055293635	3.660890801567	C
13 C13	-0.557636701759	-2.039322137904	2.923647173807	C
14 C14	-0.450391752714	-1.692228080472	1.550040753024	C
15 C15	0.846223881945	-1.669528426189	0.948031603269	C
16 C16	1.979386094174	-1.985236129461	1.692984659255	C
17 C17	1.829891613942	-2.322093678615	3.034198124615	C
18 O18	1.023903105664	-1.334334310185	-0.329246171395	O
19 C19	-1.538670492920	-1.251356299744	0.783344688678	C
20 C20	-1.326376998217	-0.747638921619	-0.503039686769	C
21 C21	-0.101929121237	-1.286608799489	-1.234540799313	C
22 C22	-0.403073256910	-2.721135004228	-1.681848002872	C
23 C23	0.313761279576	-0.389906231485	-2.403731462661	C
24 C24	1.490086292201	-0.869672988287	-3.274519455537	C
25 C25	2.827310054081	-0.677547642216	-2.607534333544	C
26 C26	3.661010599613	-1.619730638530	-2.143062807057	C
27 C27	3.386605918431	-3.101499927662	-2.181551234784	C
28 C28	4.943624040075	-1.247169853930	-1.446862229029	C
29 O29	2.946898141795	-2.624647621992	3.720828271207	O
30 Ga30	1.465025523964	2.215336824450	0.634985671610	Ga
31 Cl31	3.255417984396	1.018046366315	0.724402806811	Cl
32 Cl32	1.176068638682	3.113431942579	-1.336856762876	Cl
33 H33	-0.859510541851	1.425167094407	-0.958213074902	H
34 H34	-2.593720789249	1.382470610024	1.614724523801	H
35 H35	-3.996411505865	2.713107371531	-2.179489255756	H
36 H36	-2.245814360459	2.504452091440	-2.023230768360	H
37 H37	-3.291601554107	1.099755096995	-2.298495830987	H
38 H38	-4.818361212122	1.996223343777	1.324100405187	H
39 H39	-5.195186994677	3.054253115864	-0.047768598383	H
40 H40	-6.743862550122	1.127400904493	0.026218805130	H
41 H41	-5.799236059286	1.113167090630	-1.454423065481	H
42 H42	-5.202779840197	-0.614668723978	1.050182785479	H
43 H43	-5.234487035867	-0.370713799783	-2.752147989291	H
44 H44	-3.886444658353	-1.526891314921	-2.746498779851	H
45 H45	-5.543903824337	-2.115026804698	-2.780453545895	H
46 H46	-3.454813010218	-3.001074056175	-0.653306671174	H
47 H47	-4.489320903763	-2.777448865055	0.779819441572	H
48 H48	-5.130132182502	-3.546154821551	-0.686587550164	H
49 H49	0.478053037073	-2.618953144768	4.713584994923	H
50 H50	-1.543526483238	-2.037562598938	3.382949625010	H
51 H51	2.962365320998	-1.933289708398	1.236303510911	H
52 H52	-2.521751752710	-1.156479715361	1.246274920802	H
53 H53	-2.204192049078	-0.593089031054	-1.133074787703	H
54 H54	-0.736323416385	-3.327237608896	-0.831045029893	H

55 H55	-1.197685170813	-2.718817027303	-2.439206116080	H
56 H56	0.491319150126	-3.185843669207	-2.105285237903	H
57 H57	-0.584996272403	-0.257336810863	-3.027738137535	H
58 H58	0.584078949238	0.596551760379	-2.010179559452	H
59 H59	1.338898062272	-1.903736529420	-3.604991279592	H
60 H60	1.468740715564	-0.258046323014	-4.186551016295	H
61 H61	3.107479756677	0.367348299490	-2.449934115830	H
62 H62	2.629234996193	-3.385951581793	-2.918035688740	H
63 H63	4.301867584268	-3.661243831545	-2.410657820209	H
64 H64	3.041636215361	-3.442992717025	-1.192528943597	H
65 H65	5.807492759836	-1.710490747513	-1.942502434302	H
66 H66	5.089357490528	-0.164208010272	-1.415968055232	H
67 H67	4.939793531319	-1.613147800229	-0.408981573867	H
68 H68	2.754214034942	-2.810761129994	4.651370903217	H
69 Cl69	1.382628679066	3.671198130630	2.217198708188	Cl

Imag. Freq. -378.89 cm^{-1}
Thermochemistry at 298.150 K, 1.000 Atm
E(el) = -2849199.254054 kcal/mol
ZPVE = 356.574373 kcal/mol
Enthalpie(0K) = -2848842.679681 kcal/mol
E(tr) = 0.888732 kcal/mol
E(rot) = 0.888732 kcal/mol
E(vib) = 378.732312 kcal/mol
H-E(el) = 381.102265 kcal/mol
Enthalpie = -2848818.151790 kcal/mol
S(el) = 0.000000000000
S(tr) = 0.000071563058
S(rot) = 0.000059447597 (Symmetry number= 1)
S(vib) = 0.000239566604
G-E(el) = 311.770252 kcal/mol
Free Energy = -2848887.483803 kcal/mol

Cartesian Coordinates and Energies of 7a
#FREQ/MN15/6-31+g(d,p) //# MIN/MN15/6-31+g(d,p)
Charge = 0 Multiplicity = 1

----

1 C1	-0.947180460324	0.724270265383	-0.251765436176	C
2 O2	0.229605507884	0.675991057191	0.458874605146	O
3 C3	-2.112127981265	1.315076298738	0.491770506744	C
4 C4	-3.147367649032	1.921304393681	-0.106875364355	C
5 C5	-3.179634730211	2.210095856120	-1.591677804066	C
6 C6	-4.413687306545	2.219049734726	0.650143029277	C
7 C7	-5.551637784318	1.290267437245	0.160963793669	C
8 C8	-5.204189364564	-0.164579619029	0.324959768940	C
9 C9	-5.083061445465	-1.112502468447	-0.621367982700	C
10 C10	-5.287625628176	-0.900992214616	-2.099992020952	C
11 C11	-4.728336741920	-2.534532963228	-0.262033859945	C
12 C12	0.603361664827	-2.165418547248	3.517212251375	C
13 C13	-0.533684082401	-2.054177071479	2.784166332940	C
14 C14	-0.465769594008	-1.843681647311	1.360764786114	C
15 C15	0.838012537606	-1.839411429270	0.721744245478	C
16 C16	2.000294862334	-1.979552439494	1.479897155580	C
17 C17	1.880630960164	-2.105506193559	2.853153142969	C
18 O18	0.961156999093	-1.797508163233	-0.581378130917	O
19 C19	-1.538338128354	-1.436712878066	0.619966709684	C
20 C20	-1.290472345405	-0.780021876161	-0.671997615011	C

21 C21	-0.153786856184	-1.466262733334	-1.457816015543	C
22 C22	-0.656648860205	-2.797435525547	-2.016689208656	C
23 C23	0.438395556141	-0.558351689584	-2.540587567528	C
24 C24	1.735797397022	-1.066933490505	-3.199099414375	C
25 C25	2.971751451830	-0.692123433203	-2.420392641482	C
26 C26	3.867425330401	-1.513927486455	-1.850928926801	C
27 C27	3.762968523007	-3.017480970606	-1.834034679247	C
28 C28	5.081283244148	-0.970809218575	-1.141954640265	C
29 O29	3.006587481680	-2.189125145210	3.566042736803	O
30 Ga30	1.372154389839	2.120427232204	0.556042473383	Ga
31 Cl31	3.296015984063	1.267020358618	1.085862579441	Cl
32 Cl32	1.344487963729	3.035723286038	-1.431052205389	Cl
33 H33	-0.832050525626	1.249637182820	-1.213476171932	H
34 H34	-2.115232023688	1.153158382152	1.572558282578	H
35 H35	-3.961382468679	2.941814713474	-1.825985967073	H
36 H36	-2.225548683335	2.616615130180	-1.946167129661	H
37 H37	-3.395675359197	1.306022601282	-2.180444461372	H
38 H38	-4.249442130379	2.066790055483	1.724861974000	H
39 H39	-4.721288044390	3.263593026429	0.504621439492	H
40 H40	-6.459370291233	1.514953737993	0.737366569406	H
41 H41	-5.784771127336	1.522770771393	-0.884803806041	H
42 H42	-5.017258654628	-0.471021492191	1.360224998247	H
43 H43	-5.557858467997	0.124233064952	-2.362414729135	H
44 H44	-4.372301907588	-1.163191889313	-2.652784239858	H
45 H45	-6.075671357241	-1.567392907622	-2.474522074879	H
46 H46	-3.786195551471	-2.840587100646	-0.745761006405	H
47 H47	-4.624031643713	-2.671252149700	0.820123705277	H
48 H48	-5.497988705135	-3.231340624582	-0.618667997391	H
49 H49	0.573351634811	-2.274850460390	4.598697021210	H
50 H50	-1.512021330539	-2.061088472697	3.258056550196	H
51 H51	2.972592505669	-1.907817582957	1.004737404675	H
52 H52	-2.532842866700	-1.387338762731	1.063275882393	H
53 H53	-2.196458685434	-0.744751001097	-1.292513969012	H
54 H54	-1.050148885849	-3.430798393881	-1.211946089315	H
55 H55	-1.459282621388	-2.618216900189	-2.742207314494	H
56 H56	0.153687503071	-3.339232333270	-2.513161773289	H
57 H57	-0.350297712634	-0.410109448518	-3.293013103290	H
58 H58	0.667757368263	0.420603562270	-2.107765361746	H
59 H59	1.691439526671	-2.144005869431	-3.395935232658	H
60 H60	1.801470355422	-0.588023693382	-4.185250435178	H
61 H61	3.133796280533	0.384759456164	-2.318523902214	H
62 H62	2.832945041887	-3.395559718007	-2.267827646110	H
63 H63	4.604966147055	-3.475067981698	-2.370662943241	H
64 H64	3.818477061375	-3.385863278009	-0.798936727391	H
65 H65	6.001340359731	-1.387564572044	-1.574470753778	H
66 H66	5.126780771793	0.120129231930	-1.193750365454	H
67 H67	5.075633727509	-1.250602991390	-0.077988095896	H
68 H68	2.837053461392	-2.207816868636	4.520035722875	H
69 Cl69	0.620753435811	3.470520324055	2.073828772842	Cl

Thermochemistry at 298.150 K, 1.000 Atm
E(el) = -2849203.595369 kcal/mol
ZPVE = 357.558170 kcal/mol
Enthalpie(0K) = -2848846.037199 kcal/mol
E(tr) = 0.888732 kcal/mol
E(rot) = 0.888732 kcal/mol
E(vib) = 379.935242 kcal/mol
H-E(el) = 382.305194 kcal/mol

Enthalpie = -2848821.290175 kcal/mol
S(el) = 0.000000000000
S(tr) = 0.000071563058
S(rot) = 0.000059306639 (Symmetry number= 1)
S(vib) = 0.000247273893
G-E(el) = 311.557582 kcal/mol
Free Energy = -2848892.037787 kcal/mol

Plot of IRC transformation of **7a** in to **7** (MN15/6-31+g(d,p)

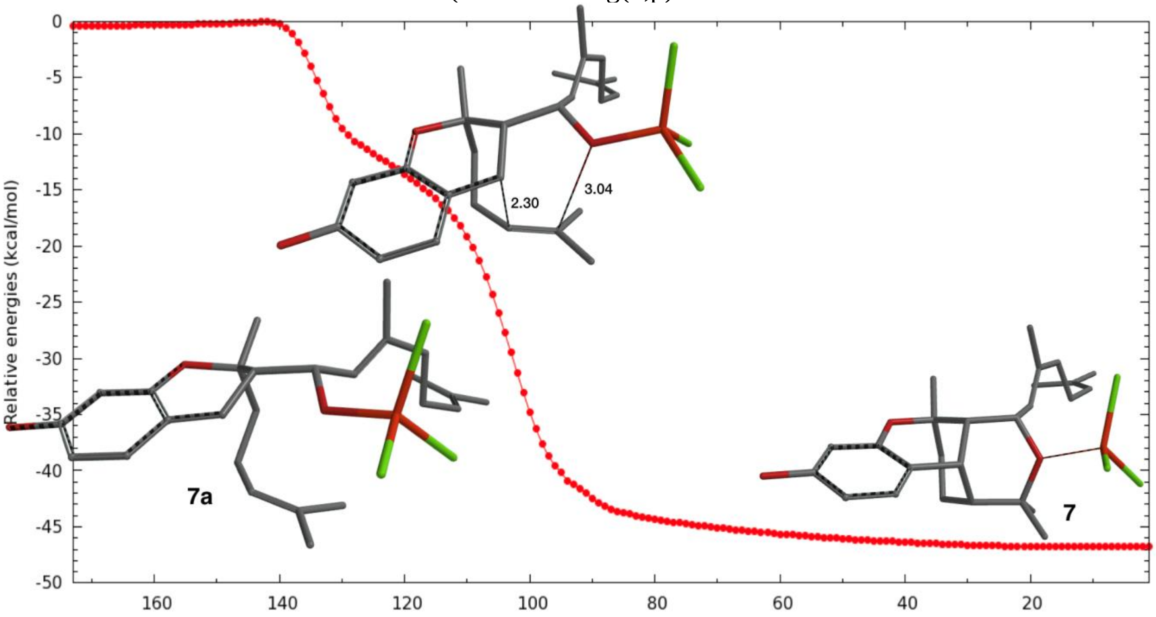

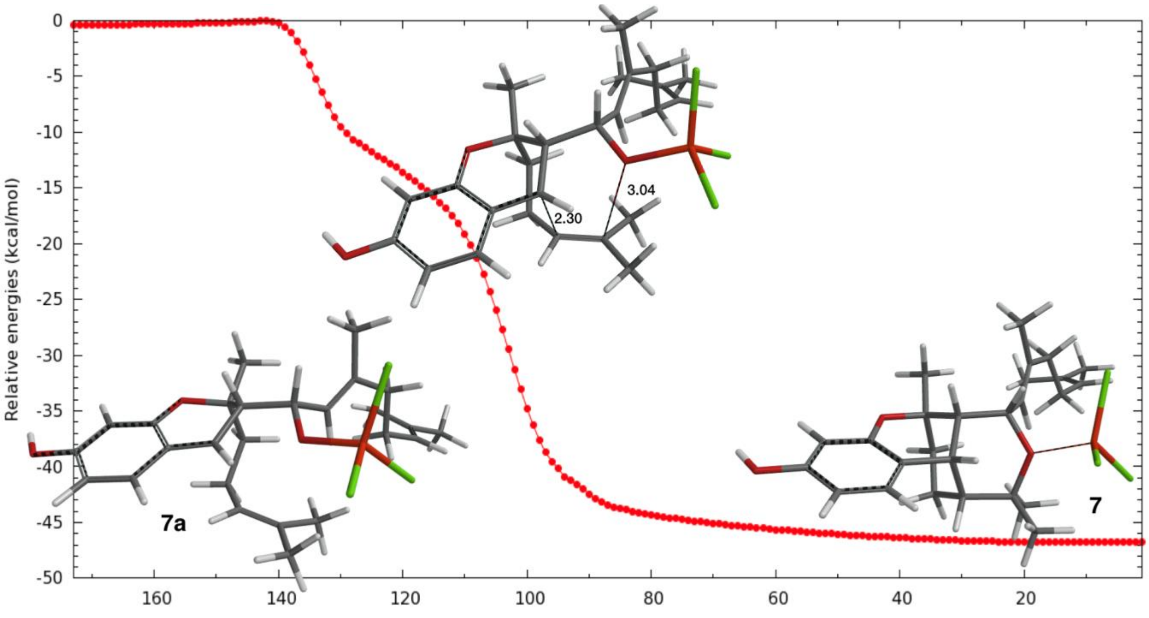

Cartesian Coordinates and Energies of **7a**
#FREQ/MN15/6-31+g(d,p) //# MIN/MN15/6-31+g(d,p)
Charge = 0 Multiplicity = 1
----

1 C1	-4.831819184652	-1.535262729985	2.496860336645	C
2 C2	-5.666083765704	-1.404939539478	1.349680971465	C
3 C3	-5.166603258558	-1.013706905697	0.109222702678	C
4 C4	-3.803294021923	-0.736317594889	-0.013093668263	C

5 C5	-2.949866914072	-0.812493493619	1.132263470848 C
6 C6	-3.500915282090	-1.249400699910	2.376287125880 C
7 O7	-3.339527114691	-0.403930269254	-1.210040573260 O
8 C8	-1.888595560900	-0.336059871252	-1.484239731703 C
9 C9	-1.212526868437	0.275066073042	-0.246219400790 C
10 C10	-1.619366963249	-0.440277447138	0.983317036118 C
11 C11	0.314757739121	0.661043581405	-0.262144825632 C
12 O12	0.848578435931	0.206454090344	0.944050231041 O
13 C13	0.480545304786	-3.024221296534	0.566278100218 C
14 C14	-0.853792672185	-2.887544480437	0.372281473676 C
15 C15	1.176643359628	0.157629199013	-1.391943967228 C
16 H16	-1.744439348339	1.241191083022	-0.113903479246 H
17 H17	1.454501599777	-0.887344594867	-1.283664924155 H
18 C18	1.796510218529	0.902693804466	-2.323607278494 C
19 C19	1.554921030846	2.371518500188	-2.556209733768 C
20 C20	1.065161960821	-2.960252866657	1.946346261768 C
21 C21	1.476195681861	-3.310726157994	-0.518014636149 C
22 C22	-1.607888905086	-2.927799124052	-0.939637463464 C
23 C23	-1.430901080381	-1.738018270296	-1.904932881361 C
24 H24	-0.904885940965	-0.454359379330	1.808105218933 H
25 C25	-1.806471368599	0.614455011763	-2.671249164962 C
26 O26	-6.961575881862	-1.696770238001	1.535539442562 O
27 C27	2.844345593457	0.320522710407	-3.243819810588 C
28 C28	3.030702442600	-1.200572698686	-3.212265120684 C
29 C29	4.051483131758	-1.658090004584	-4.222490805487 C
30 C30	3.826256081703	-1.918102344262	-5.520493406168 C
31 C31	2.475548607828	-1.803088972254	-6.179423693249 C
32 C32	4.939251435486	-2.346784716495	-6.441078771302 C
33 H33	-5.282512699216	-1.851794213015	3.431110665714 H
34 H34	-5.804811390577	-0.943736890925	-0.767671252923 H
35 H35	-2.832715111419	-1.326945584076	3.231191281337 H
36 H36	1.185327558207	2.531712961594	-3.579152932752 H
37 H37	2.498069746468	2.923252801328	-2.460550823662 H
38 H38	0.840550546618	2.818739642441	-1.860864059381 H
39 H39	1.628769401447	-3.873664781265	2.176255502421 H
40 H40	0.298335841033	-2.812536759654	2.714820993029 H
41 H41	1.775886775357	-2.125672869811	2.008572367104 H
42 H42	2.336379614227	-2.635124455235	-0.431119918078 H
43 H43	1.063756104145	-3.249343635843	-1.529849252313 H
44 H44	1.866816989324	-4.327394311716	-0.371269742036 H
45 H45	-2.677849309236	-3.059913029948	-0.726126307032 H
46 H46	-1.316573573111	-3.824721195040	-1.503692288209 H
47 H47	-0.378082848293	-1.641393502977	-2.196974513849 H
48 H48	-1.988060763261	-1.967943855239	-2.823799714295 H
49 H49	-2.465995920994	0.258181471858	-3.469075658173 H
50 H50	-0.780330210292	0.657541265186	-3.044876849895 H
51 H51	-2.120990143481	1.621959707351	-2.376423744986 H
52 H52	-7.479722650906	-1.585273937527	0.723782081829 H
53 H53	3.803529949194	0.803253615227	-2.997165422560 H
54 H54	2.620253424260	0.630960699156	-4.277500567603 H
55 H55	2.059962013689	-1.689089269163	-3.390479909967 H
56 H56	3.356396006157	-1.498625898914	-2.209080747217 H
57 H57	5.076570401075	-1.749617801763	-3.858708062465 H
58 H58	2.181650575542	-2.760776650826	-6.630269678572 H
59 H59	2.513603259238	-1.070734038361	-6.997281219075 H
60 H60	1.686699202364	-1.492611368419	-5.488011109601 H
61 H61	4.724100336821	-3.328675013530	-6.884290950775 H
62 H62	5.896183817935	-2.409957211953	-5.913992336971 H

63 H63	5.051028232847	-1.641499899591	-7.275684132559	H
64 H64	0.332663926957	1.760964082794	-0.291253982939	H
65 H65	-1.457344799234	-2.887208347727	1.277575707802	H
66 Ga66	2.554522657428	0.704674766463	1.435928768632	Ga
67 Cl67	2.830743157314	2.768525522390	0.796971593336	Cl
68 Cl68	4.041817847511	-0.608545757289	0.507041022647	Cl
69 Cl69	2.553127317126	0.419704282517	3.583648568742	Cl

Thermochemistry at 298.150 K, 1.000 Atm
E(el) = -2849193.818019 kcal/mol
ZPVE = 357.748391 kcal/mol
Enthalpie(0K) = -2848836.069628 kcal/mol
E(tr) = 0.888732 kcal/mol
E(rot) = 0.888732 kcal/mol
E(vib) = 380.110928 kcal/mol
H-E(el) = 382.480880 kcal/mol
Enthalpie = -2848811.337139 kcal/mol
S(el) = 0.000000000000
S(tr) = 0.000071563058
S(rot) = 0.000060194190 (Symmetry number= 1)
S(vib) = 0.000243642129
G-E(el) = 312.246688 kcal/mol
Free Energy = -2848881.571331 kcal/mol

Cartesian Coordinates and Energies of **Transition state 7a in 7**
#FREQ/B3LYP/6-31+g(d,p) //# SADDLE/MN15/6-31+g(d,p)
Charge = 0 Multiplicity = 1

----

1 C1	-4.864657613162	-1.502121731094	2.510795510180	C
2 C2	-5.698347038107	-1.320450714555	1.378747999138	C
3 C3	-5.178481128562	-0.963870419498	0.136624861526	C
4 C4	-3.799686364046	-0.777429227693	0.002591950302	C
5 C5	-2.946317210952	-0.922315736329	1.127784808101	C
6 C6	-3.512653804148	-1.309877854267	2.370810955605	C
7 O7	-3.335116667156	-0.459511619068	-1.209294081090	O
8 C8	-1.894102505113	-0.394766867222	-1.485817054357	C
9 C9	-1.193996092915	0.149714755946	-0.232635917056	C
10 C10	-1.564715712000	-0.665888072999	0.957286427445	C
11 C11	0.319502046309	0.571458372353	-0.278872870706	C
12 O12	0.899992220747	0.085744869255	0.898649463803	O
13 C13	0.358766003449	-2.906567517508	0.725260146315	C
14 C14	-0.979068135603	-2.831621598287	0.426450230515	C
15 C15	1.176315575283	0.128477904367	-1.436585484772	C
16 H16	-1.738423600653	1.090819973707	-0.015401327646	H
17 H17	1.472577181976	-0.912217993120	-1.372822563957	H
18 C18	1.791083548350	0.905832184370	-2.343860227435	C
19 C19	1.533077108051	2.375862169501	-2.541374913456	C
20 C20	0.812224638318	-2.867373145073	2.149628027063	C
21 C21	1.445431582444	-3.117433502388	-0.275238889007	C
22 C22	-1.626001397757	-2.952804518962	-0.938539762696	C
23 C23	-1.431541246446	-1.786870440169	-1.924389232324	C
24 H24	-0.879928673304	-0.640667535079	1.804113788424	H
25 C25	-1.807046607529	0.586963276984	-2.646213196641	C
26 O26	-7.016303123770	-1.520560803234	1.572305758516	O
27 C27	2.849832336644	0.346155377848	-3.266029884203	C
28 C28	3.044358330599	-1.175411967245	-3.256263751675	C
29 C29	4.095116113394	-1.608009988694	-4.246477243532	C

30 C30	3.906771041799	-1.854606560994	-5.552910475942	C
31 C31	2.572722639241	-1.747855048092	-6.246034608654	C
32 C32	5.049121462016	-2.257971168308	-6.448663675578	C
33 H33	-5.320265617399	-1.779643753794	3.455117293639	H
34 H34	-5.816017975758	-0.840247946098	-0.735178993817	H
35 H35	-2.845474568352	-1.433022855693	3.222071629536	H
36 H36	1.156147820163	2.554724270253	-3.558510211563	H
37 H37	2.469070385223	2.937818815949	-2.436425338686	H
38 H38	0.815589732081	2.795067507170	-1.831632584319	H
39 H39	1.457742176303	-3.725330570903	2.374176803819	H
40 H40	-0.026188991750	-2.854771048927	2.854647561758	H
41 H41	1.419836271560	-1.965444733012	2.312160781007	H
42 H42	2.260380058732	-2.397630587551	-0.120024961202	H
43 H43	1.108374691597	-3.085762867635	-1.314889046051	H
44 H44	1.876459578248	-4.111429470366	-0.086878780288	H
45 H45	-2.702336835811	-3.109658620205	-0.787095614945	H
46 H46	-1.264786056354	-3.863646591115	-1.435730707406	H
47 H47	-0.376775603880	-1.689283763585	-2.208755431947	H
48 H48	-1.982458546136	-2.026730213718	-2.843601010454	H
49 H49	-2.496202835073	0.271428205110	-3.436333031206	H
50 H50	-0.790251407538	0.616212297855	-3.044882847916	H
51 H51	-2.089148495473	1.592844901524	-2.314616833988	H
52 H52	-7.524335000636	-1.369791939493	0.761309256106	H
53 H53	3.804418690938	0.828560844393	-3.001362846292	H
54 H54	2.635060171611	0.671282683076	-4.296996576051	H
55 H55	2.081391202539	-1.665767526852	-3.469751247764	H
56 H56	3.346134962352	-1.491293547525	-2.250027448251	H
57 H57	5.111728706230	-1.690384666286	-3.857557072673	H
58 H58	2.302124591942	-2.703630616709	-6.715074033505	H
59 H59	2.624045754614	-1.006117497275	-7.054557919697	H
60 H60	1.762181401303	-1.453906442035	-5.572882646442	H
61 H61	4.857885786708	-3.236525864069	-6.909790436703	H
62 H62	5.992462357206	-2.316346329114	-5.897128514246	H
63 H63	5.174150610210	-1.540740496348	-7.271060369271	H
64 H64	0.310329217602	1.671885043863	-0.271232531375	H
65 H65	-1.631198563666	-3.015656750788	1.276978486753	H
66 Ga66	2.574956724150	0.679351529815	1.391520050733	Ga
67 Cl67	2.745050773517	2.766465536673	0.804933417173	Cl
68 Cl68	4.121868546883	-0.543867199948	0.436883567555	Cl
69 Cl69	2.610645378716	0.353465408916	3.536053421786	Cl

Imag. Freq. –137.08 cm^{-1}
Thermochemistry at 298.150 K, 1.000 Atm
E(el) = -2849193.443949 kcal/mol
ZPVE = 357.806330 kcal/mol
Enthalpie(0K) = -2848835.637620 kcal/mol
E(tr) = 0.888732 kcal/mol
E(rot) = 0.888732 kcal/mol
E(vib) = 379.584019 kcal/mol
H-E(el) = 381.953971 kcal/mol
Enthalpie = -2848811.489979 kcal/mol
S(el) = 0.000000000000
S(tr) = 0.000071563058
S(rot) = 0.000060191641 (Symmetry number= 1)
S(vib) = 0.000235566275
G-E(el) = 313.231183 kcal/mol
Free Energy = -2848880.212766 kcal/mol

Cartesian Coordinates and Energies of **7**
#FREQ/MN15/6-31+g(d,p) //# MIN/MN15/6-31+g(d,p)
Charge = 0 Multiplicity = 1
----

1 C1	-4.954299052280	-1.005914632863	2.623617328226 C
2 C2	-5.770230521288	-0.977525398872	1.485433649766 C
3 C3	-5.211572706886	-0.884099325341	0.212382331636 C
4 C4	-3.820760260675	-0.823830402701	0.075888931026 C
5 C5	-2.985725140821	-0.843368954138	1.194533585109 C
6 C6	-3.575751053621	-0.936882396155	2.460014544984 C
7 O7	-3.336027338876	-0.736293288520	-1.192538631030 O
8 C8	-1.916082310357	-0.709909564410	-1.477848591767 C
9 C9	-1.182439226359	-0.025550488672	-0.308097075578 C
10 C10	-1.501370801643	-0.784998455851	0.988721856052 C
11 C11	0.361155992170	0.144743766676	-0.393105827121 C
12 O12	1.034100385602	-0.639509070108	0.657897414824 O
13 C13	0.669596464781	-2.055723803223	0.943182024447 C
14 C14	-0.875830197098	-2.188584414649	0.860650161141 C
15 C15	1.173693761895	-0.072498100582	-1.642523669899 C
16 H16	-1.604957220204	0.987186079873	-0.237994987731 H
17 H17	1.654778682355	-1.044438623099	-1.704407861788 H
18 C18	1.491920403470	0.872810653338	-2.538315212699 C
19 C19	0.886859742565	2.249222076097	-2.541712553153 C
20 C20	1.088506228937	-2.282495833421	2.391892523033 C
21 C21	1.450409079261	-3.000829450430	0.035379393874 C
22 C22	-1.461246044821	-2.933904763866	-0.350088098247 C
23 C23	-1.420749330063	-2.142424688617	-1.658070089585 C
24 H24	-1.040020117006	-0.265726072611	1.841929488107 H
25 C25	-1.862252605078	0.066376838571	-2.788052992775 C
26 O26	-7.118844981725	-1.042702972924	1.678352332586 O
27 C27	2.503135910453	0.624681990626	-3.629723989171 C
28 C28	3.270064035837	-0.701198400786	-3.561158713350 C
29 C29	4.331281224108	-0.782990431949	-4.628479596144 C
30 C30	4.164307669475	-1.219041318243	-5.887337351923 C
31 C31	2.856548568690	-1.724823408443	-6.439326819237 C
32 C32	5.305621591125	-1.228583049756	-6.870686301198 C
33 H33	-5.414172510200	-1.072635318752	3.604215262523 H
34 H34	-5.830087163142	-0.859581246927	-0.682431625011 H
35 H35	-2.928302919602	-0.946666905776	3.336320987896 H
36 H36	0.345483278832	2.416702288815	-3.483159320514 H
37 H37	1.673189408310	3.010540196317	-2.474386153361 H
38 H38	0.179708233960	2.414307540821	-1.722089701931 H
39 H39	0.881024664250	-3.326654218635	2.647435113449 H
40 H40	0.525952875795	-1.631234024361	3.068028525653 H
41 H41	2.159731930060	-2.110013062494	2.543562418687 H
42 H42	2.521177663076	-2.789267764521	0.105983100461 H
43 H43	1.144228343524	-2.952102399505	-1.011658113121 H
44 H44	1.280186848443	-4.027124288966	0.380577156441 H
45 H45	-2.508189886279	-3.159602766636	-0.109384514199 H
46 H46	-0.964999487185	-3.903213342681	-0.476074526490 H
47 H47	-0.402382136583	-2.088093876519	-2.065872254266 H
48 H48	-2.041916078922	-2.629811194824	-2.419842659110 H
49 H49	-2.606356500169	-0.361638710192	-3.468078921857 H
50 H50	-0.877219061579	0.004154271836	-3.254416401251 H
51 H51	-2.116865195460	1.119174352027	-2.618274417976 H
52 H52	-7.586895857953	-1.003644395597	0.832835047742 H
53 H53	3.225554057356	1.455227394937	-3.601270773387 H
54 H54	2.009347852263	0.709029157482	-4.611708242934 H

55 H55	2.566770551000	-1.540839990328	-3.651694880303	H
56 H56	3.738066482492	-0.789666788419	-2.570578938948	H
57 H57	5.319997060559	-0.416177858923	-4.346703267868	H
58 H58	2.963672741323	-2.755441500030	-6.803148395682	H
59 H59	2.550433061667	-1.118790384727	-7.302527455153	H
60 H60	2.042920436778	-1.703071426698	-5.708743549125	H
61 H61	5.494294918984	-2.246193704047	-7.238208349107	H
62 H62	6.228557756346	-0.848663372756	-6.422075066796	H
63 H63	5.070002491580	-0.614068620923	-7.749813914413	H
64 H64	0.545827866304	1.168464625736	-0.048852729631	H
65 H65	-1.169804601191	-2.763714232487	1.749720393693	H
66 Ga66	2.615114357363	0.291875821769	1.387725962893	Ga
67 Cl67	2.959037335987	2.110666168930	0.281265586596	Cl
68 Cl68	4.273139084792	-1.060223987210	1.168880347285	Cl
69 Cl69	1.959240017027	0.802273793538	3.371649960765	Cl

Thermochemistry at 298.150 K, 1.000 Atm
E(el) = -2849240.227796 kcal/mol
ZPVE = 361.792762 kcal/mol
Enthalpie(0K) = -2848878.435034 kcal/mol
E(tr) = 0.888732 kcal/mol
E(rot) = 0.888732 kcal/mol
E(vib) = 382.619838 kcal/mol
H-E(el) = 384.989790 kcal/mol
Enthalpie = -2848855.238005 kcal/mol
S(el) = 0.000000000000
S(tr) = 0.000071563058
S(rot) = 0.000060155113 (Symmetry number= 1)
S(vib) = 0.000226070184
G-E(el) = 318.050478 kcal/mol
Free Energy = -2848922.177318 kcal/mol

Scheme S8. Proposed formation mechanism of Cannabicitrans 14 (*cis* union)

Plot of IRC transformation of **CBC quinone** in to **Cannabicitrans (14S)** (*cis* union) (MN15/6-31+g(d,p)

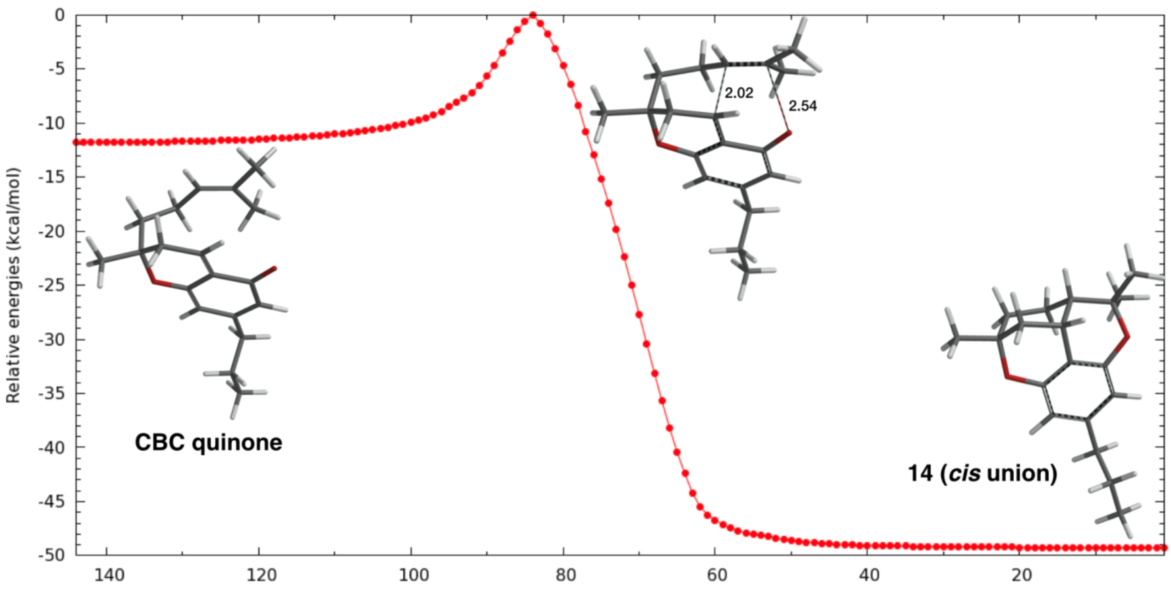

Cartesian Coordinates and Energies of **CBC quinone**
#FREQ/MN15/6-31+g(d,p) //# MIN/MN15/6-31+g(d,p)
Charge = 0 Multiplicity = 1

1 C1	0.078706729049	0.934125999294	1.575095549009 C
2 C2	0.963133715742	1.743720169168	2.213340399101 C
3 C3	0.986456175554	3.165828055634	1.927116524729 C
4 C4	0.151614932210	3.739197214901	1.010584826339 C
5 C5	-0.816790983357	2.946138862855	0.272537422758 C
6 C6	-0.859926855325	1.487191581259	0.610546514951 C
7 C7	-1.759657914743	0.685588241071	0.008302652074 C
8 C8	-1.856594879285	-0.763745129919	0.341474566210 C
9 C9	-0.571833637546	-1.334349526236	0.960106611635 C
10 O10	0.034346000096	-0.388316091243	1.868904861051 O
11 O11	-1.564238579476	3.410215337204	-0.596580140750 O
12 C12	0.035914935078	0.930490146447	-2.691246927660 C
13 C13	0.097069302440	-0.289843761909	-2.130390116601 C
14 C14	0.471100988456	-1.779389741338	-0.080455617066 C
15 C15	1.046425396408	-0.738640252481	-1.050608660524 C
16 H16	-0.599102674758	-1.052523696846	-2.494699342236 H
17 H17	-2.454173636829	1.134205036222	-0.701639762333 H
18 C18	-0.920161680965	-2.530836159543	1.839104677520 C
19 C19	1.963644303691	4.001987518570	2.712502706523 C
20 C20	1.543701396593	4.152434829454	4.182820403218 C
21 C21	2.544972812688	4.986204562141	4.979262877286 C
22 H22	1.658753974052	1.322994382104	2.935197030451 H
23 H23	0.192458031807	4.803747138729	0.793432425055 H
24 H24	-2.694037242464	-0.894179105882	1.045995421947 H
25 H25	-2.115527598626	-1.350212332728	-0.549209017469 H
26 H26	1.299210255677	-2.215428756125	0.493536466966 H
27 H27	0.019787883238	-2.602489358709	-0.656678608001 H
28 H28	1.431630720989	0.118416704831	-0.483661530368 H
29 H29	1.930455679951	-1.198939268013	-1.517372773128 H

30 H30	-1.413939054675	-3.305774311881	1.241795221214	H
31 H31	-0.013195143774	-2.952561844676	2.284194439869	H
32 H32	-1.593660963652	-2.221092221126	2.644710604583	H
33 H33	2.958545371593	3.533575424740	2.673615428434	H
34 H34	2.053086973808	4.995121369444	2.254729182246	H
35 H35	0.548734573202	4.614884633318	4.219000045743	H
36 H36	1.438812089500	3.157128360144	4.635041845795	H
37 H37	2.236876501049	5.094346643834	6.024227071485	H
38 H38	3.538267091858	4.521305747756	4.967589195146	H
39 H39	2.644275386135	5.991431636614	4.552484761090	H
40 C40	-0.992390351188	1.261491363752	-3.739774436166	C
41 H41	-1.664005725247	2.043902150396	-3.359446017084	H
42 H42	-1.590150863663	0.386580990964	-4.016746321495	H
43 H43	-0.519678261021	1.660229107947	-4.647061705986	H
44 C44	0.960662766129	2.066661993052	-2.335121410854	C
45 H45	1.391687690952	1.973717233585	-1.331562356127	H
46 H46	0.429863672315	3.023506956016	-2.384500545898	H
47 H47	1.795154762686	2.112478451252	-3.048456158223	H

Thermochemistry at 298.150 K, 1.000 Atm
E(el) = -557898.529523 kcal/mol
ZPVE = 256.155200 kcal/mol
Enthalpie(0K) = -557642.374324 kcal/mol
E(tr) = 0.888732 kcal/mol
E(rot) = 0.888732 kcal/mol
E(vib) = 268.206388 kcal/mol
H-E(el) = 270.576340 kcal/mol
Enthalpie = -557627.953183 kcal/mol
S(el) = 0.000000000000
S(tr) = 0.000068289466
S(rot) = 0.000054530142 (Symmetry number= 1)
S(vib) = 0.000124255665
G-E(el) = 224.350552 kcal/mol
Free Energy = -557674.178971 kcal/mol

Cartesian Coordinates and Energies of **Transition state CBC in 14 (*cis* union)**
#FREQ/B3LYP/6-31+g(d,p) //# SADDLE/MN15/6-31+g(d,p)
Charge = 0 Multiplicity = 1

----

1 C1	0.103904068856	0.857615789896	1.633430110867	C
2 C2	0.941745014821	1.685279926463	2.344010434126	C
3 C3	1.069292226055	3.056066438872	1.960341854188	C
4 C4	0.400425616718	3.567498548322	0.866596093591	C
5 C5	-0.499959940299	2.741263110239	0.108319375274	C
6 C6	-0.692928487319	1.383513478765	0.580834289345	C
7 C7	-1.515807044829	0.550868008635	-0.192255781412	C
8 C8	-1.809141893397	-0.799916081888	0.401542995898	C
9 C9	-0.530197371267	-1.388323191861	0.997724301988	C
10 O10	0.038666489544	-0.470645858358	1.950526463112	O
11 O11	-1.061856547909	3.074667705528	-0.975476070479	O
12 C12	-0.242881923511	1.225659162737	-2.513814988717	C
13 C13	-0.358458527358	0.047240137670	-1.775385588953	C
14 C14	0.476061403649	-1.713253195256	-0.118435170733	C
15 C15	0.834487583985	-0.569321549652	-1.074054987877	C
16 H16	-1.123464066848	-0.650491612032	-2.130469048978	H
17 H17	-2.285985863896	1.052032824359	-0.777294282368	H
18 C18	-0.830843117494	-2.642827668636	1.804810036105	C

19 C19	1.961924656777	3.941069305254	2.796752368965 C
20 C20	1.317407997485	4.306547575376	4.141619835594 C
21 C21	2.223290528102	5.195249865048	4.991174298225 C
22 H22	1.529349622011	1.284395434548	3.166719280785 H
23 H23	0.542532973993	4.599067127350	0.551581628727 H
24 H24	-2.558667849354	-0.673942473817	1.196821089577 H
25 H25	-2.225393190693	-1.499744335554	-0.333807875931 H
26 H26	1.390752918994	-2.091008192998	0.355604299698 H
27 H27	0.046795785146	-2.546251779163	-0.697009964110 H
28 H28	1.375419664066	0.211142989613	-0.522430923492 H
29 H29	1.541411023587	-0.953680398403	-1.824266545579 H
30 H30	-1.284824220798	-3.409012013805	1.166512490414 H
31 H31	0.093379929361	-3.042870631513	2.234947614615 H
32 H32	-1.519946950131	-2.405190659068	2.621661942149 H
33 H33	2.916982396104	3.429631773471	2.988665838657 H
34 H34	2.194080441430	4.860745366370	2.243648426556 H
35 H35	0.361105478162	4.810436910248	3.948530605709 H
36 H36	1.075276258748	3.383691338403	4.686016190239 H
37 H37	1.755457679334	5.452604495684	5.947272643444 H
38 H38	3.174100724954	4.692264816930	5.206698797754 H
39 H39	2.455314415304	6.131042200347	4.468169205857 H
40 C40	-1.317501467437	1.628875353729	-3.472502605955 C
41 H41	-1.528075456792	2.697378238429	-3.369311423100 H
42 H42	-2.240978874955	1.064564973615	-3.301562812416 H
43 H43	-0.988795996843	1.438941675889	-4.504716538653 H
44 C44	1.034061593939	2.002791049366	-2.502712826876 C
45 H45	1.373621364612	2.192775460734	-1.473776050348 H
46 H46	0.919674663029	2.966513575914	-3.004336134084 H
47 H47	1.830272128191	1.423291401436	-2.995185948899 H

Imag. Freq. -449.04 cm^{-1}
Thermochemistry at 298.150 K, 1.000 Atm
E(el) = -557886.764769 kcal/mol
ZPVE = 256.324208 kcal/mol
Enthalpie(0K) = -557630.440561 kcal/mol
E(tr) = 0.888732 kcal/mol
E(rot) = 0.888732 kcal/mol
E(vib) = 267.376640 kcal/mol
H-E(el) = 269.746592 kcal/mol
Enthalpie = -557617.018177 kcal/mol
S(el) = 0.000000000000
S(tr) = 0.000068289466
S(rot) = 0.000054363297 (Symmetry number= 1)
S(vib) = 0.000108530771
G-E(el) = 226.494019 kcal/mol
Free Energy = -557660.270749 kcal/mol

Cartesian Coordinates and Energies of **14 (*cis* union)**
#FREQ/MN15/6-31+g(d,p) //# MIN/MN15/6-31+g(d,p)
Charge = 0 Multiplicity = 1
----

1 C1	0.006165745791	0.734419752356	1.741492808351 C
2 C2	0.853709873326	1.544221363572	2.506139799618 C
3 C3	1.145067649856	2.845453059624	2.080235497792 C
4 C4	0.647121422111	3.312017264282	0.850195726048 C
5 C5	-0.212306611692	2.491147598399	0.127529994789 C
6 C6	-0.646867899625	1.266502169366	0.634682382794 C

7 C7	-1.467652595672	0.439734859022	-0.301506263697 C
8 C8	-1.813939562188	-0.892225896039	0.346471621623 C
9 C9	-0.536368320001	-1.458009081415	0.959730088958 C
10 O10	-0.105265785255	-0.595228286491	2.037119756502 O
11 O11	-0.628695887425	2.787476472431	-1.143055569571 O
12 C12	-0.400098694324	1.738960145260	-2.138831090007 C
13 C13	-0.537177326408	0.309023100058	-1.521752929720 C
14 C14	0.524813526033	-1.590116275355	-0.153243408496 C
15 C15	0.770996754807	-0.362354950928	-1.054283921689 C
16 H16	-1.009025129385	-0.329878398380	-2.286726111824 H
17 H17	-2.374876118378	0.987983336545	-0.590271144358 H
18 C18	-0.768343709735	-2.802755695466	1.629705610904 C
19 C19	2.009725632845	3.745630112465	2.929613621349 C
20 C20	1.174102958292	4.644726629746	3.851919370374 C
21 C21	2.044912023819	5.559721698910	4.709991343390 C
22 H22	1.325757669280	1.130559257188	3.394586389423 H
23 H23	0.953938048741	4.274546087614	0.446949128534 H
24 H24	-2.565733448741	-0.761069318581	1.135499945553 H
25 H25	-2.212701075328	-1.600065348927	-0.393321474618 H
26 H26	1.470598703307	-1.911595307744	0.301635710954 H
27 H27	0.177180913664	-2.423345187678	-0.783352929749 H
28 H28	1.392607367534	0.363121919514	-0.515379869335 H
29 H29	1.360156756645	-0.682789904306	-1.923086280869 H
30 H30	-1.129473083532	-3.536389909841	0.900833909300 H
31 H31	0.164167345592	-3.170746089615	2.071353375453 H
32 H32	-1.512213581414	-2.697233567583	2.426291710437 H
33 H33	2.692532842731	3.139136610530	3.540591393608 H
34 H34	2.634722843790	4.378901578477	2.284105600267 H
35 H35	0.486415877965	5.241097317911	3.236948262289 H
36 H36	0.544704950207	4.009584827988	4.489811943265 H
37 H37	1.440330461291	6.196635096557	5.364101747645 H
38 H38	2.722017180630	4.973988537808	5.343771005358 H
39 H39	2.662656309795	6.213418967434	4.081985317235 H
40 C40	-1.501302541681	1.959317373025	-3.168741566385 C
41 H41	-1.508232516941	3.008506652583	-3.481364488983 H
42 H42	-2.482872044018	1.718532276209	-2.744603635228 H
43 H43	-1.331291664215	1.326759793615	-4.047247706721 H
44 C44	0.970031549822	2.006202807608	-2.754480201675 C
45 H45	1.747390761157	2.022119622538	-1.982546050164 H
46 H46	0.959776476140	2.981901142292	-3.251522890916 H
47 H47	1.224978831846	1.236987148998	-3.493290986994 H

Thermochemistry at 298.150 K, 1.000 Atm
E(el) = -557936.018552 kcal/mol
ZPVE = 259.817280 kcal/mol
Enthalpie(0K) = -557676.201271 kcal/mol
E(tr) = 0.888732 kcal/mol
E(rot) = 0.888732 kcal/mol
E(vib) = 270.106576 kcal/mol
H-E(el) = 272.476528 kcal/mol
Enthalpie = -557663.542024 kcal/mol
S(el) = 0.000000000000
S(tr) = 0.000068289466
S(rot) = 0.000054266343 (Symmetry number= 1)
S(vib) = 0.000100009875
G-E(el) = 230.836285 kcal/mol
Free Energy = -557705.182266 kcal/mol

Scheme S9. . Proposed formation mechanism of Cannabicitrans 14 (*trans* union)

Plot of IRC transformation of **CBC quinone** in to **Cannabicitrans (14R)** (*trans* union) (MN15/6-31+g(d,p)

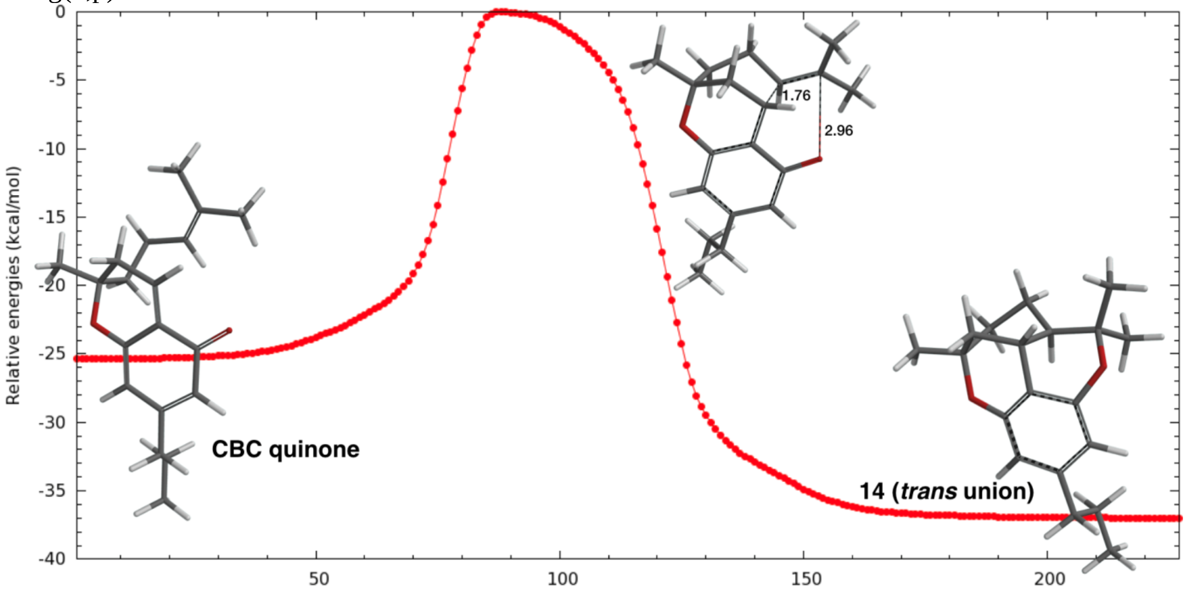

Cartesian Coordinates and Energies of **CBC quinone**
#FREQ/MN15/6-31+g(d,p) //# MIN/MN15/6-31+g(d,p)
Charge = 0 Multiplicity = 1

----

1 C1	-0.561170842255	-1.102357983308	-0.468395344679 C
2 C2	-1.864216350752	-1.472763272779	-0.416300931056 C
3 C3	-2.909925459445	-0.523391411758	-0.758805277516 C
4 C4	-2.632810253172	0.764474255226	-1.112322292590 C
5 C5	-1.265241434319	1.260566132377	-1.156538996737 C
6 C6	-0.193336690891	0.247109699248	-0.889994286595 C
7 C7	1.103510685506	0.556621640064	-1.096298498480 C
8 C8	2.164111798050	-0.459935795428	-0.830940476126 C
9 C9	1.695354918746	-1.455689339298	0.233241306348 C
10 O10	0.423597233616	-1.998577135403	-0.193845871963 O
11 O11	-0.971947704155	2.436233438371	-1.395009866657 O
12 C12	2.989066445707	2.433531562884	0.895039667779 C
13 C13	2.219616871338	1.568104339979	1.576016084702 C
14 C14	1.494932587935	-0.843843050478	1.636254128304 C
15 C15	2.548300824584	0.187325550298	2.081732363972 C
16 H16	1.191439181416	1.880587363575	1.780593411375 H
17 H17	1.350120275868	1.547504897554	-1.475939933428 H
18 C18	2.638877440526	-2.646189799586	0.297303699662 C
19 C19	-4.332278843163	-1.009008747646	-0.650154923847 C
20 C20	-4.765706429288	-1.223763359697	0.808127600835 C
21 C21	-6.201796844565	-1.733796520775	0.906150233515 C
22 H22	-2.125745896465	-2.487782291123	-0.127427501284 H
23 H23	-3.424650538353	1.472174594283	-1.345109427037 H
24 H24	2.376712574341	-1.017171569892	-1.757182264056 H
25 H25	3.103878858344	0.014049914255	-0.526896026936 H
26 H26	0.506865063301	-0.365409747826	1.674768971401 H
27 H27	1.457364098833	-1.682729586088	2.342572723989 H
28 H28	2.557100319895	0.205881050914	3.180163367545 H
29 H29	3.554167456804	-0.134403897313	1.778924656853 H
30 H30	3.616695825862	-2.327772207154	0.677831577593 H

31 H31	2.232326805524	-3.409396371963	0.968486779539 H
32 H32	2.773647473503	-3.086759110009	-0.695959954772 H
33 H33	-5.006474444744	-0.287389697158	-1.128272967949 H
34 H34	-4.433712851915	-1.960117799800	-1.194181719573 H
35 H35	-4.081221613416	-1.933621386393	1.291648817741 H
36 H36	-4.660664262705	-0.274766279756	1.349987003820 H
37 H37	-6.506924456881	-1.879243830712	1.947463833256 H
38 H38	-6.900967494566	-1.024735154871	0.446990656671 H
39 H39	-6.312633731110	-2.692866350051	0.385827600796 H
40 C40	4.419784365743	2.185859679881	0.494950160508 C
41 H41	4.514535522537	2.195547118734	-0.600952274945 H
42 H42	4.820920672258	1.238561145909	0.865978305093 H
43 H43	5.064910458252	2.991931629895	0.867773713857 H
44 C44	2.428988928582	3.750687512614	0.422969103806 C
45 H45	1.373403343318	3.861294864808	0.688487909595 H
46 H46	2.507193137602	3.832038819919	-0.670479139937 H
47 H47	2.992270006587	4.594319532931	0.843513218575 H

Thermochemistry at 298.150 K, 1.000 Atm
E(el) = -557897.847781 kcal/mol
ZPVE = 256.319362 kcal/mol
Enthalpie(0K) = -557641.528418 kcal/mol
E(tr) = 0.888732 kcal/mol
E(rot) = 0.888732 kcal/mol
E(vib) = 268.372907 kcal/mol
H-E(el) = 270.742859 kcal/mol
Enthalpie = -557627.104922 kcal/mol
S(el) = 0.000000000000
S(tr) = 0.000068289466
S(rot) = 0.000054823891 (Symmetry number= 1)
S(vib) = 0.000126137640
G-E(el) = 224.110010 kcal/mol
Free Energy = -557673.737771 kcal/mol

Cartesian Coordinates and Energies of **Transition state CBC in 14 (*trans* union)**
#FREQ/B3LYP/6-31+g(d,p) //# SADDLE/MN15/6-31+g(d,p)
Charge = 0 Multiplicity = 1

----

1 C1	-0.389606777641	-1.165037470721	-0.426030498485 C
2 C2	-1.744931101066	-1.428168518203	-0.422367168842 C
3 C3	-2.662949150899	-0.365990845326	-0.670482024185 C
4 C4	-2.230459298353	0.927846125477	-0.890204021628 C
5 C5	-0.829079097937	1.243009110474	-0.911244824195 C
6 C6	0.080087087965	0.130380834529	-0.728151760698 C
7 C7	1.483596265601	0.460011995888	-0.698139632142 C
8 C8	2.360611713097	-0.772728358619	-0.760962845387 C
9 C9	1.815733018211	-1.792835023464	0.238370321607 C
10 O10	0.497991360879	-2.184230238555	-0.178474386693 O
11 O11	-0.335635048399	2.407202947678	-1.023852679390 O
12 C12	2.147039020431	2.524082131821	0.585690287466 C
13 C13	1.635761608354	1.232026021878	0.880151250488 C
14 C14	1.752489329512	-1.155235949948	1.651364518782 C
15 C15	2.416865372007	0.222948953963	1.733469955932 C
16 H16	0.613779354612	1.301154332735	1.266511749610 H
17 H17	1.731179273629	1.230762452075	-1.429500531045 H
18 C18	2.635205054568	-3.071597954567	0.235204036156 C
19 C19	-4.140171987641	-0.679807367066	-0.619249710466 C

20 C20	-4.642534841600	-0.911436447223	0.812739561856	C
21 C21	-6.135116835495	-1.232269695963	0.855160115728	C
22 H22	-2.098936687680	-2.439563048215	-0.236120695911	H
23 H23	-2.941515244278	1.735598184384	-1.051400456760	H
24 H24	2.299226468649	-1.195528347139	-1.772126205693	H
25 H25	3.418107238233	-0.560083446141	-0.561460087356	H
26 H26	0.695195369934	-1.053601351297	1.932528714941	H
27 H27	2.211810639085	-1.830575351985	2.383061400768	H
28 H28	2.396930830541	0.574898518903	2.771214956809	H
29 H29	3.473007898786	0.174997905789	1.438579974187	H
30 H30	3.654622150229	-2.864670073042	0.582352168914	H
31 H31	2.178507339592	-3.809016083435	0.903754719461	H
32 H32	2.680971289882	-3.494200277399	-0.773950873040	H
33 H33	-4.707069341780	0.144627167164	-1.071774735843	H
34 H34	-4.348886072406	-1.579106181958	-1.217605106995	H
35 H35	-4.067694691731	-1.729606666879	1.267665866799	H
36 H36	-4.428530201454	-0.014768858033	1.409863379253	H
37 H37	-6.486512664149	-1.393657449503	1.879906297592	H
38 H38	-6.723290970750	-0.413290604833	0.423315748062	H
39 H39	-6.356442560665	-2.138053554759	0.277220583355	H
40 C40	3.451042207348	2.668239388383	-0.117161097117	C
41 H41	3.242090603846	2.934341803641	-1.167395813328	H
42 H42	4.049965022701	1.753091764425	-0.111910292368	H
43 H43	4.033797241227	3.498096557136	0.299042538024	H
44 C44	1.390746837693	3.779194608231	0.837589842252	C
45 H45	0.417242950654	3.588837801099	1.291454269025	H
46 H46	1.217516086740	4.312758245970	-0.106558954809	H
47 H47	1.997343939929	4.437252312695	1.479612145314	H

Imag. Freq. -119.36 cm^{-1}

Thermochemistry at 298.150 K, 1.000 Atm
E(el) = -557872.461389 kcal/mol
ZPVE = 256.584152 kcal/mol
Enthalpie(0K) = -557615.877237 kcal/mol
E(tr) = 0.888732 kcal/mol
E(rot) = 0.888732 kcal/mol
E(vib) = 267.516367 kcal/mol
H-E(el) = 269.886319 kcal/mol
Enthalpie = -557602.575070 kcal/mol
S(el) = 0.000000000000
S(tr) = 0.000068289466
S(rot) = 0.000054490234 (Symmetry number= 1)
S(vib) = 0.000106986148
G-E(el) = 226.898984 kcal/mol
Free Energy = -557645.562405 kcal/mol

Cartesian Coordinates and Energies of **14 (*trans* union)**
#FREQ/MN15/6-31+g(d,p) //# MIN/MN15/6-31+g(d,p)
Charge = 0 Multiplicity = 1

----

1 C1	-0.229225582309	-1.272904615763	-0.523878762753	C
2 C2	-1.617431480022	-1.449062908711	-0.461617553346	C
3 C3	-2.463278136648	-0.360137938703	-0.717940950813	C
4 C4	-1.930371256553	0.929809539093	-0.920666789708	C
5 C5	-0.548617307816	1.063222753465	-1.003471882343	C
6 C6	0.267596440535	-0.074416031804	-1.027590831408	C
7 C7	1.698624626183	0.229329302144	-0.810261056395	C

8 C8	2.539135496919	-1.032880306391	-0.816367499624	C
9 C9	2.016785261416	-1.890026816307	0.317166280709	C
10 O10	0.609482446829	-2.190279554000	0.058804342451	O
11 O11	0.126387389115	2.261627361916	-0.878281068077	O
12 C12	1.067524294383	2.343628103074	0.271016640830	C
13 C13	1.529678444385	0.906938947153	0.576118561642	C
14 C14	2.278550565526	-1.117078885541	1.661855621000	C
15 C15	2.605928354268	0.402704238025	1.524141091937	C
16 H16	0.629239377798	0.465680928668	1.031798218053	H
17 H17	2.066679117095	0.956441361844	-1.542858533725	H
18 C18	2.661151923492	-3.266489031609	0.373952214374	C
19 C19	-3.960715552693	-0.531406408706	-0.593248763851	C
20 C20	-4.480714692570	-0.146421834185	0.799077732569	C
21 C21	-5.992971857169	-0.319589702177	0.920744843631	C
22 H22	-2.029747901618	-2.402460665358	-0.137468031123	H
23 H23	-2.577071996962	1.804759812080	-0.940013828252	H
24 H24	2.438596606840	-1.557187435954	-1.775451675024	H
25 H25	3.603352043237	-0.820122023536	-0.656157078235	H
26 H26	1.382624148845	-1.227857926075	2.287477519091	H
27 H27	3.097653036067	-1.626341264513	2.184960358359	H
28 H28	2.550976620939	0.871393154253	2.514884127768	H
29 H29	3.621755977404	0.565320340924	1.138421591309	H
30 H30	3.744115448799	-3.171239992995	0.510975707516	H
31 H31	2.249836906475	-3.841505556256	1.211096153803	H
32 H32	2.462964774138	-3.811808052744	-0.554321042459	H
33 H33	-4.469801094264	0.084361442073	-1.347749829316	H
34 H34	-4.232373352303	-1.575168921502	-0.801309205364	H
35 H35	-3.965005623561	-0.760508566310	1.550049973850	H
36 H36	-4.200326078622	0.895273025160	1.008126534410	H
37 H37	-6.353713623335	-0.043261157718	1.917062963460	H
38 H38	-6.518074058346	0.305151069080	0.187871857781	H
39 H39	-6.283294584843	-1.360930461441	0.735220676794	H
40 C40	2.180844699105	3.278255956091	-0.168900917063	C
41 H41	1.755188255350	4.230424818402	-0.501900013367	H
42 H42	2.762661949272	2.855364502740	-0.994079902916	H
43 H43	2.855644481248	3.469808234479	0.674111373500	H
44 C44	0.284692961543	2.923838937121	1.441779570901	C
45 H45	-0.602330162839	2.307991800104	1.634691133487	H
46 H46	-0.040329783969	3.945149252874	1.217753286376	H
47 H47	0.904028286964	2.940565366957	2.347688596679	H

Thermochemistry at 298.150 K, 1.000 Atm
E(el) = -557909.443127 kcal/mol
ZPVE = 259.599329 kcal/mol
Enthalpie(0K) = -557649.843798 kcal/mol
E(tr) = 0.888732 kcal/mol
E(rot) = 0.888732 kcal/mol
E(vib) = 269.969548 kcal/mol
H-E(el) = 272.339500 kcal/mol
Enthalpie = -557637.103626 kcal/mol
S(el) = 0.000000000000
S(tr) = 0.000068289466
S(rot) = 0.000054171682 (Symmetry number= 1)
S(vib) = 0.000101257145
G-E(el) = 230.483614 kcal/mol
Free Energy = -557678.959512 kcal/mol

----

4. Single Crystal X-ray Diffraction.

X-ray data collection of suitable single crystals of compound **7a** was done at 100(2) K on a Bruker VENTURE area detector equipped with graphite monochromated Mo-Kα radiation (λ = 0.71073 Å) by applying the ω-scan method. The data reduction was performed with the APEX2[10] software and corrected for absorption using SADABS.[11] Crystal structures were solved by direct methods using the SIR97 program[12] and refined by full-matrix least-squares on F^2 including all reflections using anisotropic displacement parameters by means of the WINGX crystallographic package.[13] All hydrogen atoms were included as fixed contributions riding on attached atoms with isotropic thermal displacement parameters 1.2 times or 1.5 times those of their parent atoms for the organic ligands. Details of the structure determination and refinement of compounds are summarized in Table S1. Crystallographic data (excluding structure factors) for the structures reported in this paper have been deposited with the Cambridge Crystallographic Data Center as supplementary publication no. CCDC 2021364. Copies of the data can be obtained free of charge on application to the Director, CCDC, 12 Union Road, Cambridge, CB2 1EZ, U.K. (Fax: +44-1223-335033; e-mail: deposit@ccdc.cam.ac.uk or http://www.ccdc.cam.ac.uk).

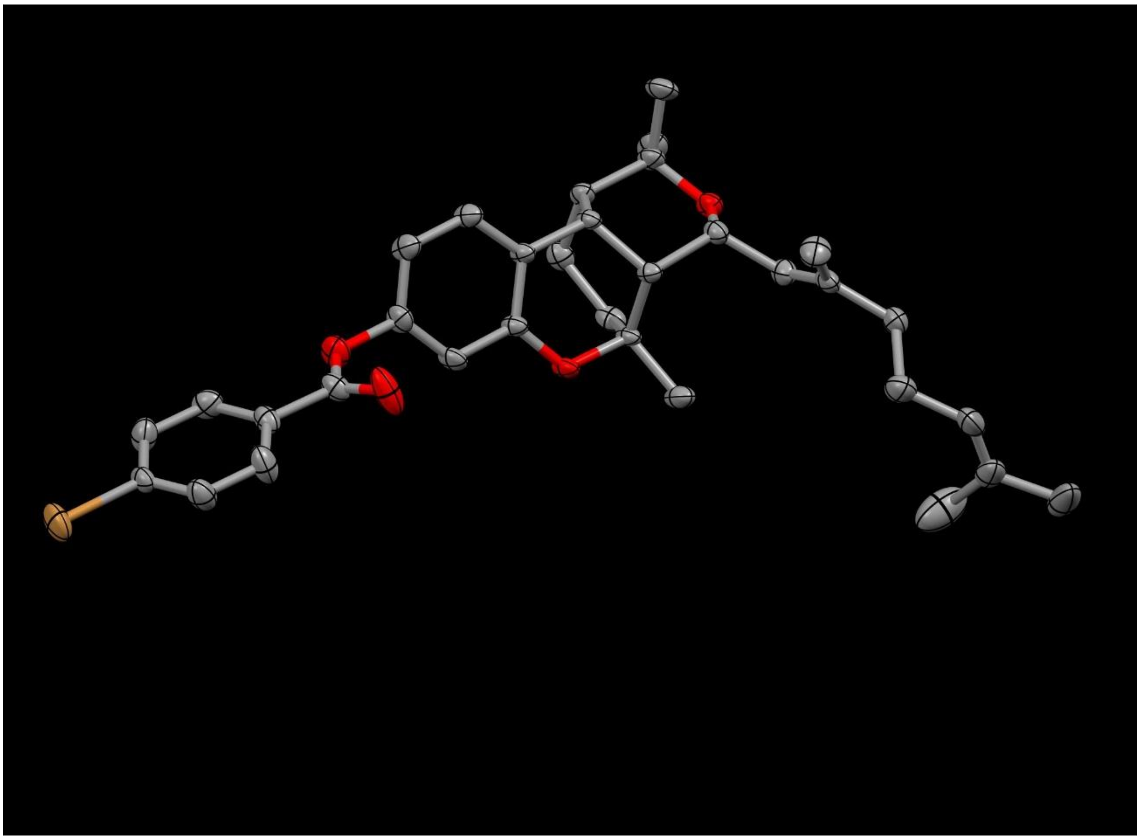

Figure S6. Mercury perspective of compound **7a**. Thermal ellipsoids are drawn at the 50% probability level. Colour code: grey, carbon; red, oxygen; tan, bromine. Hydrogen atoms have been omitted for clarity

Table S1. Crystallographic data and structure refinement details of all compounds.

**Compound**	**7a**
Chem. form.	$C_{33}H_{38}BrO_4$
CCDC	2021364
Form. weight	578.54
Cryst. system	Triclinic
Space group	*P*-1
a (Å)	10.5014(7)
b (Å)	10.5314(6)
c (Å)	14.5299(9)
α (°)	84.151(3)
β (°)	80.170(3)
γ (°)	65.279(3)
V (Å^3)	1437.39(16)
Z	2
GOF [a]	1.069
R_{int}	0.0667
R_1 [b] / wR^2 [c] [I>2σ(I)]	0.0741 / 0.1814
R_1 [b] / wR^2 [c] (all data)	0.1007 / 0.2004

[a] $S = [\sum w(F_0^2 - F_c^2)^2 / (N_{obs} - N_{param})]^{1/2}$ [b] $R_1 = \sum||F_0|-|F_c|| / \sum|F_0|$ [c] $wR_2 = [\sum w(F_0^2 - F_c^2)^2 / \sum wF_0^2]^{1/2}$
$w = 1/[\sigma^2(F_0^2) + (aP)^2 + bP]$ where $P = (\max(F_0^2,0) + 2Fc^2)/3$

5. References

[1] Álvarez, J. M.; Raya-Barón, A.; Nieto, P. M.; Cuca, L. E.; Carrasco-Pancorbo, A.; Fernández-Gutiérrez, A.; Fernández, I. Flavonoid Glycosides from Persea caerulea. Unraveling Their Interactions with SDS-Micelles Through Matrix-Assisted DOSY, PGSE, Mass Spectrometry, and NOESY. *Magn. Reson. Chem.* **2016**, *54*, 718–728.

[2] Frisch, M. J.; Trucks, G. W.; Schlegel, H. B.; Scuseria, G. E.; Robb, M. A.; Cheeseman, J. R.; Scalmani, G.; Barone, V.; Mennucci, B.; Petersson, G. A.; Nakatsuji, H.; Caricato, M.; Li, X.; Hratchian, H. P.; Izmaylov, A. F.; Bloino, J.; Zheng, G.; Sonnenberg, J. L.; Hada, M.; Ehara, M.; Toyota, K.; Fukuda, R.; Hasegawa, J.; Ishida, M.; Nakajima, T.; Honda, Y.; Kitao, O.; Nakai, H.; Vreven, T.; Montgomery, J. A., Jr.; Peralta, J. E.; Ogliaro, F.; Bearpark, M.; Heyd, J. J.; Brothers, E.; Kudin, K. N.; Staroverov, V. N.; Kobayashi, R.; Normand, J.; Raghavachari, K.; Rendell, A.; Burant, J. C.; Iyengar, S. S.; Tomasi, J.; Cossi, M.; Rega, N.; Millam, J. M.; Klene, M.; Knox, J. E.; Cross, J. B.; Bakken, V.; Adamo, C.; Jaramillo, J.; Gomperts, R.; Stratmann, R. E.; Yazyev, O.; Austin, A. J.; Cammi, R.; Pomelli, C.; Ochterski, J. W.; Martin, R. L.; Morokuma, K.; Zakrzewski, V. G.; Voth, G. A.; Salvador, P.; Dannenberg, J. J.; Dapprich, S.; Daniels, A. D.; Farkas, O.; Foresman, J. B.; Ortiz, J. V.; Cioslowski, J.; Fox, D. J.; Revision D.01 ed.; Gaussian, Inc.: Wallingford, CT.

[3] Lynch, B. J.; Zhao, Y.; Truhlar, D. G. J. Phys. Chem. A 2003, 107, 1384. (b) Koch, W.; Holthausen, M. C. A Chemist’s Guide to Density Functional Theory; 2nd.; Wiley-VCH: Weinheim, Germany, 2000. (c) Parr, R. G.; Yang, W. Density Functional Theory of Atoms and Molecules; Clarendon Press: Oxford, UK, 1989.

[4] (a) Yu, H. S.; He, X.; Li, S. L.; Truhlar, D. G. A Kohn–Sham Global-Hybrid Exchange–Correlation Density Functional with Broad Accuracy for Multi-Reference and

Single-Reference Systems and Noncovalent Interactions. *Chem. Sci.* **2016**, *7* (8), 5032–5051. (b) Stephens, P. J.; Devlin, F. J.; Chabalowski, C. F.; Frisch, M. J. Ab Initio Calculation of Vibrational Absorption and Circular Dichroism Spectra Using Density Functional Force Fields. *J. Phys. Chem.* **1994**, *98*, 11623–11627. (b) Becke, A. D. J. Density-Functional Thermochemistry. III. The Role of Exact Exchange. *Chem. Phys.* **1993**, *98*, 5648–5652. (c) Becke, A. D. A New Mixing of Hartree-Fock and Local Density-functional Theories. *J. Chem. Phys.* **1993**, *98*, 1372–1377. (d) Lee, C.; Yang, W.; Parr, R. G. Development of the Colle-Salvetti Correlation-Energy Formula into a Functional of the Electron Density. *Physical Review B* **1988**, *37*, 785–789. (e) Schuchardt, K. L.; Didler, B. T.; Elsethagen, T.; Sun, L.; Gurumoorthi, V.; Chase, J.; Li, J.; Windus, T. L. Basis Set Exchange: A Community Database for Computational Sciences. *J. Chem. Inf. Model.* **2007**, *47*, 1045–1052. (f) Feller, D. J. The Role of Databases in Support of Computational Chemistry Calculations. *Comput. Chem.* **1996**, *17*, 1571–1586.

[5] Ohno, K.; Maeda, S. A Scaled Hypersphere Search Method for the Topography of Reaction Pathways on the Potential Energy Surface. *Chem. Phys. Lett.* **2004**, *384*, 277–282.

[6] Maeda, S.; Ohno, K. Global Mapping of Equilibrium and Transition Structures on Potential Energy Surfaces by the Scaled Hypersphere Search Method: Applications to ab Initio Surfaces of Formaldehyde and Propyne Molecules. *J. Phys. Chem. A* **2005**, *109*, 5742–5753.

[7] Ohno, K.; Maeda, S. Global Reaction Route Mapping on Potential Energy Surfaces of Formaldehyde, Formic Acid, and Their Metal-Substituted Analogues. *J. Phys. Chem. A* **2006**, *110*, 8933–8941.

[8] (a) González, C.; Schlegel, H. B. An Improved Algorithm for Reaction Path Following. *J. Chem. Phys.* **1989**, *90*, 2154–2161. (b) González, C.; Schlegel, H. B. Improved Algorithms for Reaction Path Following: Higher-Order Implicit Algorithms. *J. Chem. Phys.* **1991**, *95*, 5853–5860. (c) Hratchian, H. P.; Schlegel, H. B. Accurate Reaction Paths Using a Hessian Based Predictor–Corrector Integrator. *J. Chem. Phys.* **2004**, *120*, 9918–9924. (d) Hratchian, H. P.; Schlegel, H. B. Using Hessian Updating To Increase the Efficiency of a Hessian Based Predictor-Corrector Reaction Path Following Method. *J. Chem. Theory Comput.* **2005**, *1*, 61–69.

[9] Spartan08.Wavefunction, Inc., Irvine, CA.

[10] Bruker Apex2, Bruker AXS Inc., Madison, Wisconsin, USA, 2004.

[11] Sheldrick, G.M. SADABS, Program for Empirical Adsorption Correction, Institute for Inorganic Chemistry, University of Gottingen: Germany, 1996.

[12] Altomare, A.; Burla, M. C.; Camilla, M.; Cascarano, G. L.; Giacovazzo, C.; Guagliardi, A.; Moliterni, A. G. G.; Polidori, G.; Spagna, R. SIR97: a New Tool for Crystal Structure Determination and Refinement *J. Appl. Crystallogr.* **1999**, *32*, 115–119.

[13] (a) Sheldrick, G. M. SHELX-2014, Program for Crystal Structure Refinement; University of Göttingen, Göttingen, Germany, 2014. (b) Farrugia, L. J. WinGX Suite for Small-Molecule Single-Crystal Crystallography *J. Appl. Cryst.* **1999**, *32*, 837–838.
